# Formation of macrocyclic ring systems by carbonylation of trifunctional P/B/B frustrated Lewis pairs†
†Electronic supplementary information (ESI) available: additional experimental details, further spectral and crystallographic data, additional data from the solid state NMR and theoretical studies. CCDC 1549697–1549702. For ESI and crystallographic data in CIF or other electronic format see DOI: 10.1039/c7sc04394e


**DOI:** 10.1039/c7sc04394e

**Published:** 2017-12-14

**Authors:** Long Wang, Shunxi Dong, Constantin G. Daniliuc, Lei Liu, Stefan Grimme, Robert Knitsch, Hellmut Eckert, Michael Ryan Hansen, Gerald Kehr, Gerhard Erker

**Affiliations:** a Organisch-Chemisches Institut , Westfälische Wilhelms-Universität Münster , Corrensstr. 40 , 48149 Münster , Germany . Email: erker@uni-muenster.de; b Mulliken Center for Theoretical Chemistry , Institut für Physikalische und Theoretische Chemie , Universität Bonn , Beringstr. 4 , 53115 Bonn , Germany; c Institut für Physikalische Chemie , Westfälische Wilhelms-Universität Münster , Corrensstr. 28/30 , 48149 Münster , Germany; d Institute of Physics in Sao Carlos , University of Sao Paulo , CEP 369 , Sao Carlos , SP 13566-590 , Brazil

## Abstract

The trifunctional P/B/B frustrated Lewis pairs **11a–c** featuring bulky aryl groups at phosphorus [Dmesp (**a**), Tipp (**b**), Mes* (**c**)] react with H_2_ by heterolytic hydrogen splitting followed by cleavage of HB(C_6_F_5_)_2_ to give the zwitterionic six-membered heterocyclic PH phosphonium/borate products **14a–c**. Compounds **11a,b** react with carbon monoxide by means of a Lewis acid induced CO insertion/rearrangement sequence that eventually results in the formation of the macrocyclic dimers **17a,b**. The respective carbonylation reaction of the Mes*P/B/B FLP gives the macrocyclic trimer **18c**. The new products were characterized spectroscopically and by X-ray diffraction and the reaction mechanism was analyzed by DFT calculations.

## Abstract 2

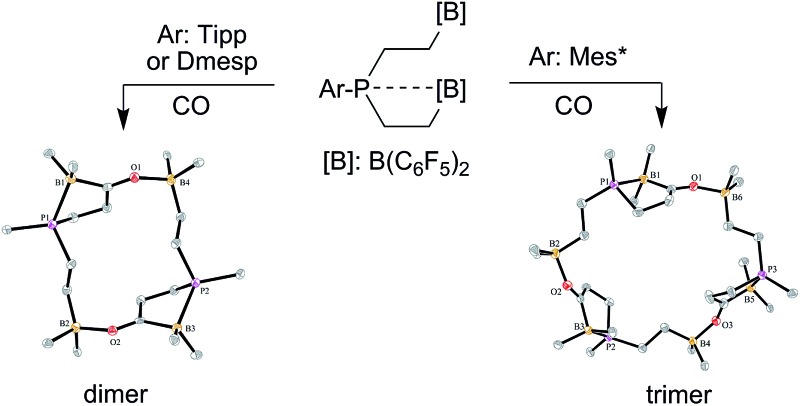
The trifunctional P/B/B frustrated Lewis pairs **11a–c** featuring bulky aryl groups at phosphorus [Dmesp (**a**), Tipp (**b**), Mes* (**c**)] were synthesized. Compounds **11a,b** react with carbon monoxide and form the macrocyclic dimers **17a,b**, while the carbonylation reaction of the Mes*P/B/B FLP **11c** gives the macrocyclic trimer **18c**.

## Introduction

Macrocyclic compounds have very interesting structural features.^1^,^2^ Many such systems play significant roles in medicine and biology^3^–^7^ and many serve as important chemical reagents.^8^–^12^ Macrocyclic ring closure is often difficult to achieve selectively since ring closure principally competes with entropically favoured formation of the linear oligomers. Chemical synthesis of macrocycles, therefore, has relied on a variety of specific measures in order to achieve the required chemoselectivity; high dilution is one important and often used principle^13^–^15^ as is template directed synthesis. Conformational features as well as electrostatic effects may play a role.^16^–^19^ There are some reactions that seem to bear a “natural” tendency for macrocyclic ring formation.^20^–^28^

We have now found that Lewis pair formation might favor intermolecular cyclooligomerization in cases where the direct internal interaction of the Lewis acid and base functionalities is effectively precluded by specific geometric restrictions. We have found that this may selectively lead to cyclodimeric and even cyclotrimeric ring systems in a rather simple experimental procedure. First examples will be presented and discussed in this account.

## Results and discussion

**Scheme 1:**
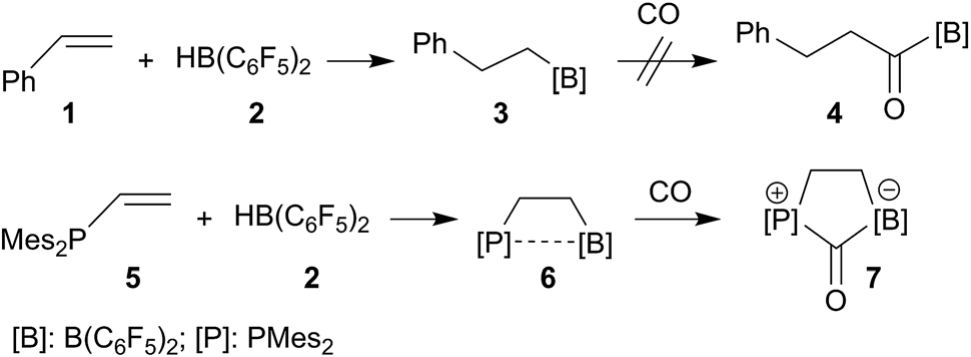
Behavior of strongly electrophilic pentafluorophenyl containing boranes toward carbon monoxide.

Alkyl boranes are important building blocks in organic synthesis. Many such systems are readily available by convenient hydroboration routes.^29^–^31^ Many alkyl boranes insert carbon monoxide into the boron–carbon bond. This reaction type has been used for the preparation of CO derived ketones, aldehydes or alcohols.^32^ Hydroboration of alkenes with Piers' borane [HB(C_6_F_5_)_2_] (**2**) occurs readily.^33^,^34^ The resulting products, such as the respective styrene (**1**) hydroboration compound PhCH_2_CH_2_B(C_6_F_5_)_2_ (**3**), however, do not readily insert CO at ambient conditions (r.t., 1.5 bar CO pressure, see Scheme 1, see the ESI† for details). CO insertion was not observed even in the presence of additional B(C_6_F_5_)_3_. The difference is even more pronounced with vicinal P/B frustrated Lewis pairs (FLPs), such as compound **6**, which readily reacts with CO, but does not form the CO insertion product into the [B]–CH_2_– linkage but rather undergoes cooperative 1,1-P/B addition to the carbon atom of carbon monoxide to yield the CO-bridged product **7**. A number of related P/B FLPs show a similar behavior.^35^–^40^

We reasoned that this behavior might originate from the very special properties of the strong B(C_6_F_5_)_2_ Lewis acid unit making the alkyl migration step to carbon monoxide unfavorable. Introduction of a second B(C_6_F_5_)_2_ group into the system might potentially provide a way out of this behavior: specifically located it could function as an activator for the P/B bonded carbonyl unit and thus initiate the otherwise unfavorable CO insertion reaction in such systems. This turned out to be a successful concept and, in addition, it opened an easy pathway to several rather unusually structured macrocyclic ring systems. Three such examples with some of their remarkable characteristic features will be reported in this account.

**Scheme 2:**
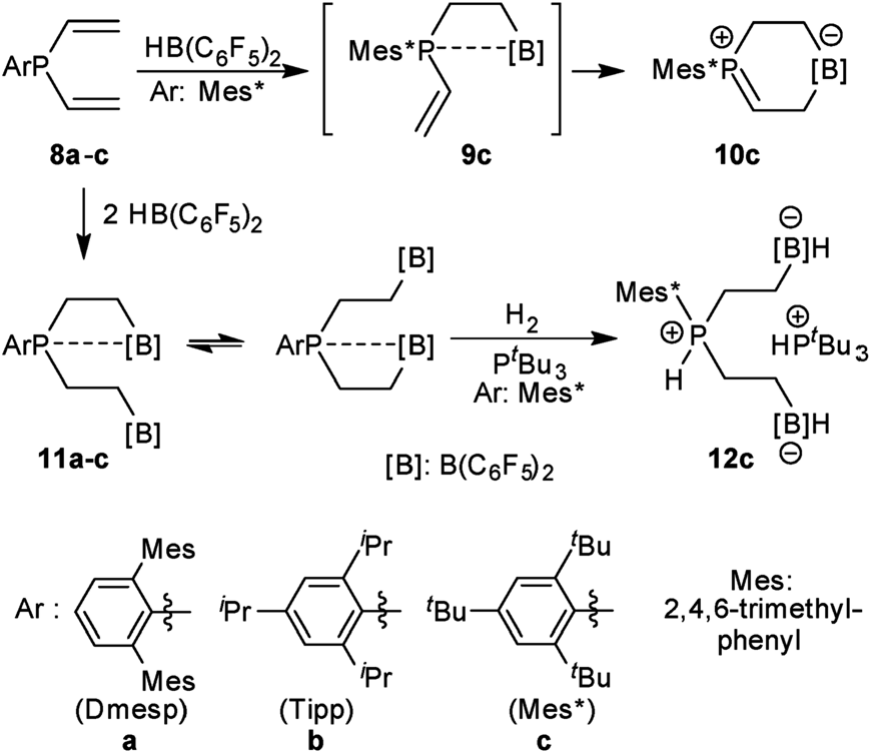
Formation and some previously reported reactions of the P/B/B FLP systems **11**.

We have prepared the aryldivinylphosphanes **8a–c** by treatment of the respective ArPCl_2_ precursors^41^–^47^ with two molar equiv. of vinyl magnesium bromide. We had reported the reaction of compound **8c** with one molar equiv. of HB(C_6_F_5_)_2_ which had given the unique zwitterionic methylene phosphonium product **10c***via* internal B(C_6_F_5_)_2_ addition to the adjacent vinyl phosphane (see Scheme 2).^48^ Addition of a second equiv. of Piers' borane had given the P/B/B49a–e system **11c**, for which we had observed a dynamic equilibrium of the P···B/B coordination by dynamic ^19^F NMR spectroscopy (see Scheme 2).49*f* We have now also generated the P/B/B systems **11a,b** featuring the bulky 2,6-dimesitylphenyl (Dmesp) and 2,4,6-triisopropylphenyl (Tipp) aryl groups at phosphorus, respectively. Compound **11a** also features an equilibrating dynamic structure in solution analogous to the previously described behavior of **11c**. We had shown that the P/B/B system **11c** splits dihydrogen in the presence of the external base ^*t*^Bu_3_P to give **12c**.49*f*

**Fig. 1:**
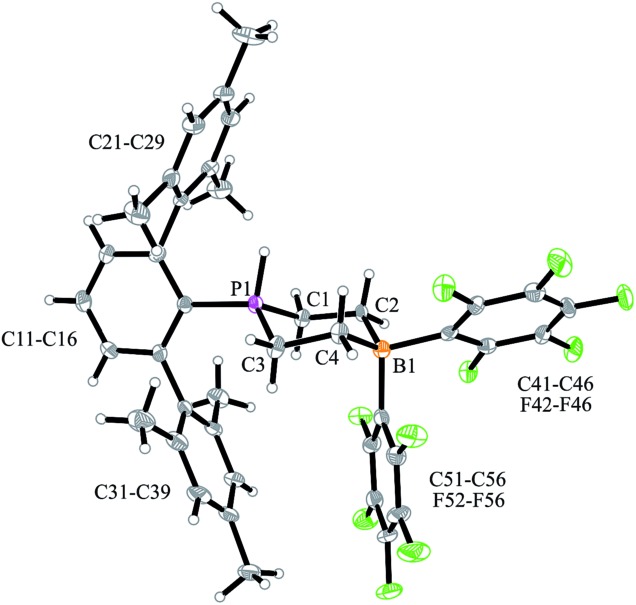
A view of the molecular structure of the P/B/B hydrogenation product **14a** (thermal ellipsoids are shown with 50% probability). Selected bond lengths (Å) and angles (°): P1–C1 1.786(3), P1–C3 1.786(3), C1–C2 1.540(4), C3–C4 1.546(5), C2–B1 1.648(5), C4–B1 1.652(5), C1–P1–C3 106.7(2), C2–B1–C4 107.5(3), ΣP1^CCC^ 339.2(5).

**Scheme 3:**
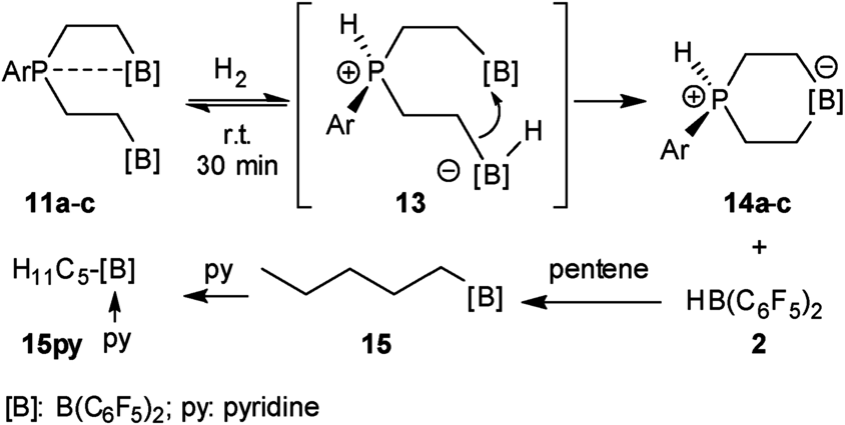
Reaction of the P/B/B FLPs **11** with dihydrogen.

We have now exposed the small series of P/B/B FLPs **11a–c** to dihydrogen in the absence of the external base and found a markedly different behavior which indicated a surprising mode of participation of the extra –B(C_6_F_5_)_2_ Lewis acid. Typically, the *in situ* generated system **11a** was exposed to a H_2_ atmosphere (1.5 bar) in dichloromethane solution for 30 min at r.t. to give a mixture of the zwitterionic heterocyclic phosphonium/borate product **14a** (Ar: Dmesp) and HB(C_6_F_5_)_2_. The latter was removed from the mixture by the hydroboration reaction with 1-pentene converting it to pentane soluble pentyl-B(C_6_F_5_)_2_. It was isolated and identified as its pyridine adduct **15py** (for details see the ESI†). The heterocycle **14a** was eventually isolated as a white solid in 78% yield. Its X-ray crystal structure analysis (see Fig. 1) showed the presence of the chair-shaped 1,4-P/B heterocycle with tetracoordinated boron and the PH(Dmesp) phosphonium unit being part of it. In solution, compound **14a** shows a typical borate ^11^B NMR feature at *δ* –14.6 and a phosphonium ^31^P NMR signal at *δ* 9.0 (^1^H: *δ* 5.56, ^1^*J*_PH_ = 463.0 Hz). The ^13^C NMR spectrum shows signals of the six-membered core unit at *δ* 19.0 (PCH_2_, ^1^*J*_PC_ = 43.1 Hz) and *δ* 20.8 (broad, BCH_2_), respectively (see Scheme 3, also see the ESI† for details).

The P/B/B compounds **11b,c** reacted analogously with dihydrogen with formation of the PH/B products **14b,c**. We isolated them both in *ca.* 70% yield; both were characterized by spectroscopy and by X-ray diffraction (see the ESI† for details). We assume a reaction pathway (see Scheme 3) that is initiated by heterolytic splitting of dihydrogen by using a P/B pair^50^–^58^ of the P/B/B FLP **11** to give the PH^+^/BH^–^/B intermediates **13**. We assume that the additional –B(C_6_F_5_)_2_ Lewis acid becomes actively involved and forms the six-membered P/B heterocycles **14** by a σ-bond metathesis type reaction^59^–^61^ with concomitant formation of HB(C_6_F_5_)_2_. The formation of the products **14** and HB(C_6_F_5_)_2_ (**2**) gave us a strong indication of the active role of the additional-B(C_6_F_5_)_2_ Lewis acid in the compounds **11**. This we used advantageously in the reaction of the P/B/B FLPs **11a–c** with carbon monoxide.

We generated the P/B/B system **11b** (Ar: Tipp) *in situ* (24 h, r.t., see Scheme 2) and then exposed the solution to a carbon monoxide atmosphere (1.5 bar, r.t.). After 30 min reaction time a white precipitate of the cyclodimeric CO insertion product **17b** had formed. It was isolated as a white solid in 54% yield (for details see the ESI†). Compound **17b** is thermally quite stable in solution. However, it lost carbon monoxide upon heating for 6 h at 80 °C in benzene-d_6_ solution to re-form the starting material **11b**.

**Scheme 4:**
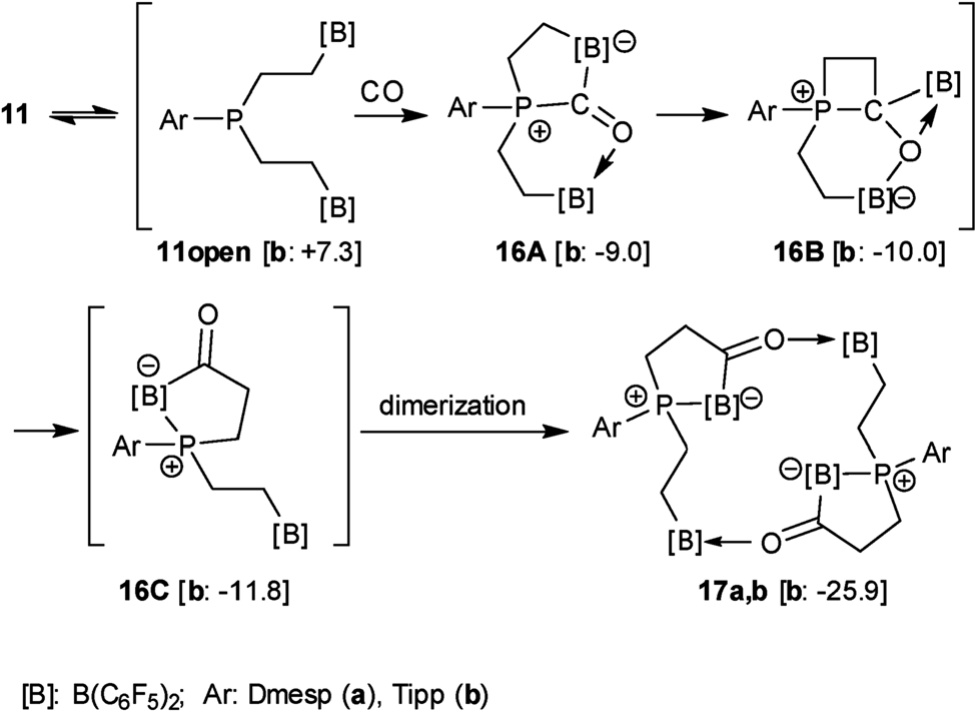
Carbonylation reaction of the P/B/B FLPs **11a, **with computed reaction Gibbs free energies at PW6B95-D3/def2-TZVP (COSMO-RS, toluene)//PBEh-3c level of the theory. The energy of **11b** + CO is selected as the reference and all values are given in kcal mol^–1^ at 298 K. The value of the dimer **17b** refers to the monomeric subunit for the purpose of comparability.

We assume a reaction pathway as it is depicted in Scheme 4, which was supported by the results of DFT calculations^69^ (for details see the ESI†). All structures were optimized with a composite DFT method PBEh-3c,^69^ followed by single point energy calculations at the PW6B95-D3 level of theory^65^,^67^,^68^ with a Gaussian AO def2-TZVP basis set.^64^,^66^ The COSMO-RS (conductor-like screening model for real solvents) solvation model^62^,^63^ (with toluene as the solvent) was used to compute solvation free energies. Endergonic opening of the P···B linkage of **11b** yields the reactive P/B/B intermediate **11open**, which may undergo the typical 1,1-P/B FLP addition reaction to carbon monoxide.^35^,^36^ Carbonyl activation by the remaining pendent –B(C_6_F_5_)_2_ functionality^70^,^71^ *via***16A** might initiate the kinetically facile and thermodynamically feasible formation of the CO insertion product^32^**16B**. Isomerization forms the P/B Lewis pair. In **16C** the direct internal interaction of the carbonyl oxygen with the pendent borane Lewis acid is geometrically precluded; the system may serve as an active C=O/B frustrated Lewis pair. This leads to dimerization giving the observed macrocyclic reaction product **17b**.

**Fig. 2:**
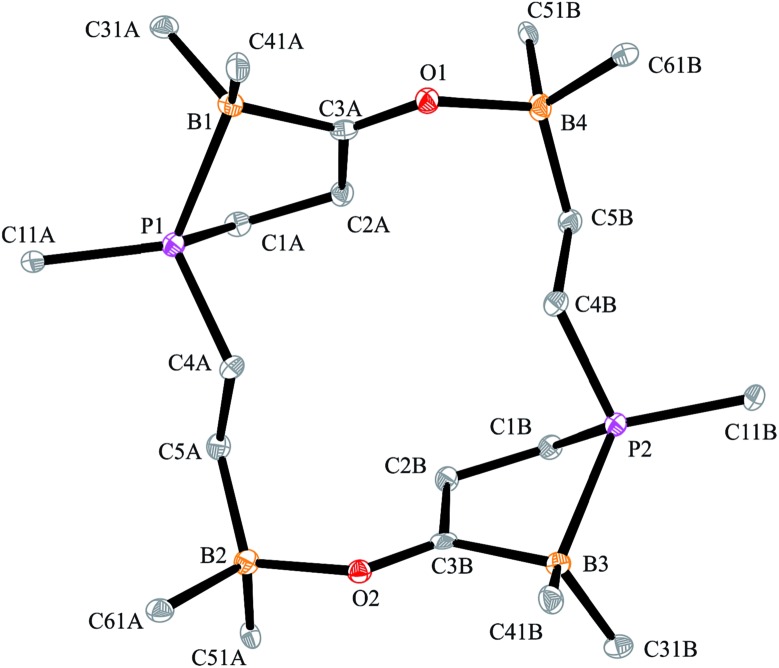
A view of the structure of the framework of macrocyclic dimer **17b** (the bulky substituents at boron and phosphorus were omitted for clarity; thermal ellipsoids are shown with 50% probability). Selected bond lengths (Å) and angles (°): P1–B1 2.063(2), B1–C3A 1.632(3), C3A–O1 1.254(2), O1–B4 1.609(2), P2–B3 2.067(2), B3–C3B 1.648(3), C3B–O2 1.255(2), O2–B2 1.618(2), B1–C3A–O1 120.8(2), C3A–O1–B4 129.6(1), B3–C3B–O2 122.7(2), C3B–O2–B2 130.8(1).

The X-ray crystal structure analysis confirmed the sixteen-membered cyclodimeric heterocyclic ring structure. The monomeric units are connected by a pair of carbonyl C=O···borane interactions. The remaining boron atoms form Lewis pair interactions with their adjacent phosphane Lewis bases. In this situation two diastereoisomers are possible due to the phosphorus chirality;^72^,^73^ we found the near to *C*_2_-symmetric *rac*-structure in the crystal (see Fig. 2, also see the ESI† for details).

In solution, compound **17b** shows the NMR features of the symmetry-equivalent monomeric subunits. We monitored eight separate ^1^H NMR sp^3^-CH signals of the methylene groups, the ^19^F NMR signals of four different C_6_F_5_ substituents at boron and a single ^31^P NMR signal at *δ* 21.0. From a ^13^C labelled sample we located the ^13^C NMR carbonyl resonance at *δ* 269.0.^70^,^71^ Compound **17b** shows a *ν̂*(CO) = 1588 cm^–1^ [*ν̂*(^13^CO) = 1547 cm^–1^] carbonyl stretching band.

The Dmesp substituted P/B/B system **11a** reacts analogously with CO. We isolated the *C*_2_-symmetrical dimer **17a** in 61% yield and characterized it by C,H-elemental analysis, by NMR (^10^B: *δ* 7.8, –10.0; ^31^P: *δ* 19.1) and IR spectroscopy (*ν̂*(CO) = 1579 cm^–1^) and by X-ray diffraction (for details see the ESI†). It is thermally slightly less stable than **17b**. Compound **17a** lost CO upon heating to 50 °C (12 h) in dichloromethane solution to give the starting material **11a**.

**Scheme 5:**
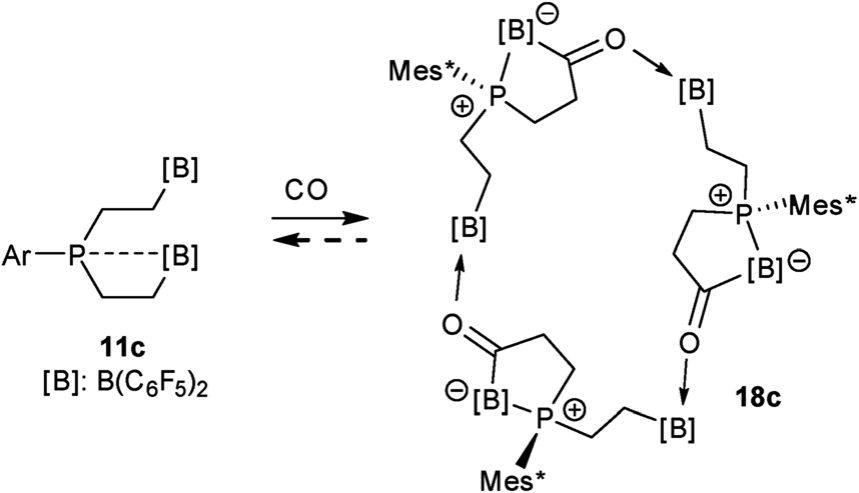
Formation of the cyclotrimeric carbonylation product **18c**.

**Fig. 3:**
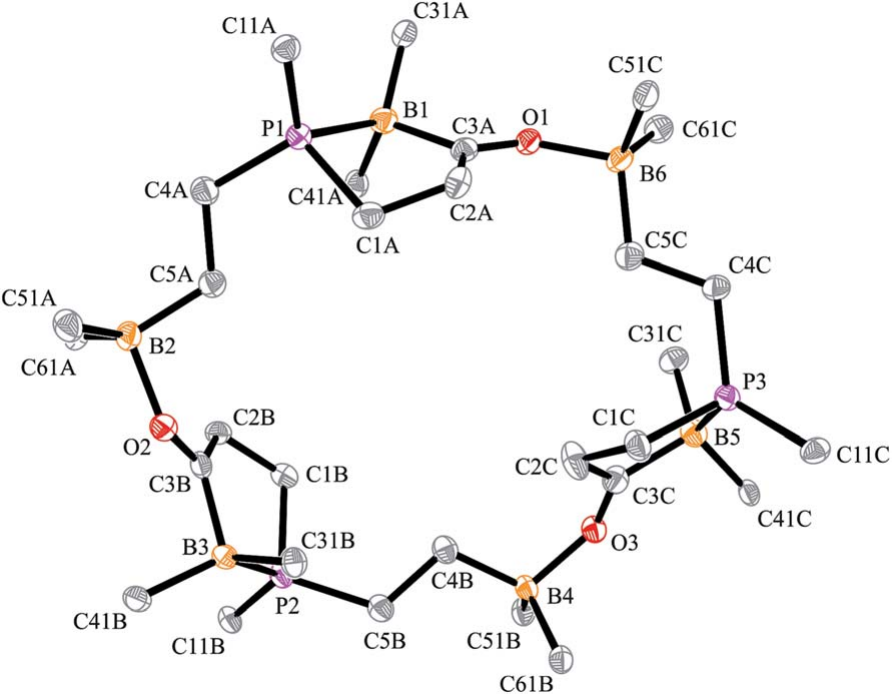
A view of the core structure of the macrocyclic P/B/B carbonylation trimer **18c** (the substituents at boron and phosphorus are omitted for clarity; thermal ellipsoids are shown with 15% probability). Selected bond lengths (Å) and angles (°): P1–B1 2.096(5), C3A–O1 1.253(5), O1–B6 1.590(6), P3–B5 2.110(5), C3C–O3 1.245(5), O3–B4 1.603(6), P2–B3 2.094(5), C3B–O2 1.239(5), O2–B2 1.625(6), B1–C3A–O1 117.7(4), C3A–O1–B6 133.7(3), B5–C3C–O3 119.6(4), C3C–O3–B4 133.4(3), B3–C3B–O2 118.3(4), C3B–O2–B2 131.9(4).

The carbonylation reaction of the Mes*P/B/B system **11c** took a slightly different course. Exposure of the *in situ* generated Mes*P/B/B system **11c** to CO (1.5 bar, r.t. 30 min) gave compound **18c** (81% isolated) (see Scheme 5, for details see the ESI†). The compound was stable in the solid state but lost carbon monoxide with re-formation of the starting material **11c** in solution (CD_2_Cl_2_). Therefore, the solution NMR data were monitored using *in situ* generated samples at low temperature (for details see the ESI†). Single crystals of compound **18c** for the X-ray crystal structure analysis were obtained from a toluene solution in a carbon monoxide atmosphere at –5 °C. Compound **18c** shows a macrocyclic twenty four-membered core structure. It is composed of three monomeric subunits that were probably formed by a CO insertion/rearrangement sequence analogous to the one described in Scheme 4; this was supported by DFT calculations (for details see the ESI†). The presence of the three phosphorus chirality centers would principally allow for two diastereoisomers, an all-*cis*-(of averaged *C*_3_-symmetry) and a *cis*-, *trans*-, *trans*-isomer. The latter structural situation is found in the crystal of compound **18c**. Each of the three symmetry inequivalent but chemically closely related subunits features a five-membered P/B containing heterocyclic carbonyl moiety. The C=O group is used for bridging to the pendent –B(C_6_F_5_)_2_ Lewis acid of the next monomeric subunit (see Fig. 3).

**Fig. 4:**
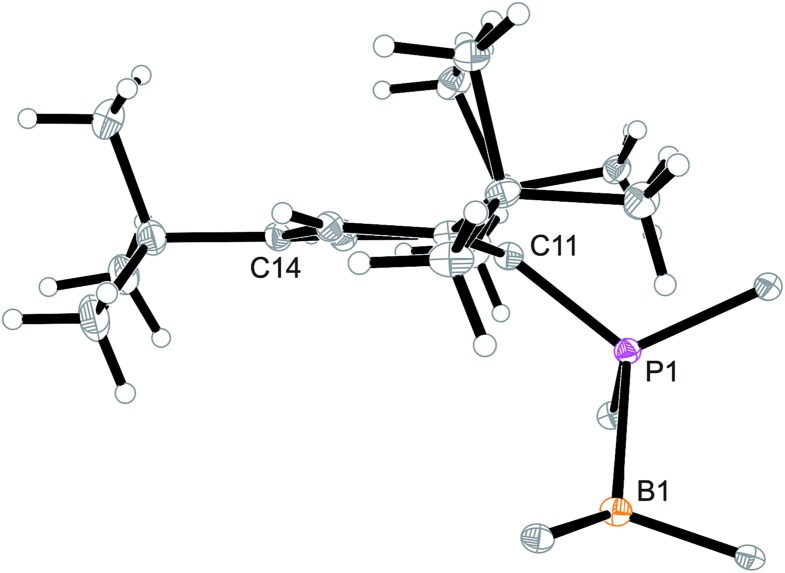
Side view of a 2,4,6-tri-^t^butylphenyl-P (*i.e.* Mes*-P) unit of the macrocyclic trimer **18c**. The P1–C11–C14 angle of this unit amounts to 144.1°.

Arylphosphanes usually have the C(aryl)-P vector oriented in line with the aryl plane. Very bulky arylphosphanes may deviate from this behavior (which may be expressed by the P1–C11–C14 angle as schematically shown in Fig. 4 for one of the three Mes*-P units of the trimer **18c**). We note an almost co-linear arrangement for the pair of Tipp-P units in the dimer **17b**. The respective P1–C11–C14 angles for the pair of crystallographically independent Tipp-P subunits were found at 174.4° and 173.1°. We find a slightly bent structure for the more bulky Dmesp-P groups in compound **17a** with P1–C11–C14 angles of 171.0° and 166.2°, respectively, but we note a rather extreme bending of the Mes*-P moiety. In the Mes*-PCl_2_ reagent the P1–C11–C14 type angle amounts to 156°.^46^ In the three Mes*-P subunits in our trimer **18c** we find this distortion of the (aryl)C–P moieties being increased further by *ca.* 10° to P1–C11–C14 values of 144.1°, 147.9°, and 146.1°, respectively. Fig. 4 shows a side-on projection of one out of the three Mes*-P moieties of compound **18c**, which visualizes this strong distortional effect. This might actually have created an overall conformational situation that may have contributed to determining the observed chemistry of this system to a considerable extent, especially the specific association behaviour of the monomeric subunits forming the observed cyclotrimer **18c**.

**Fig. 5:**
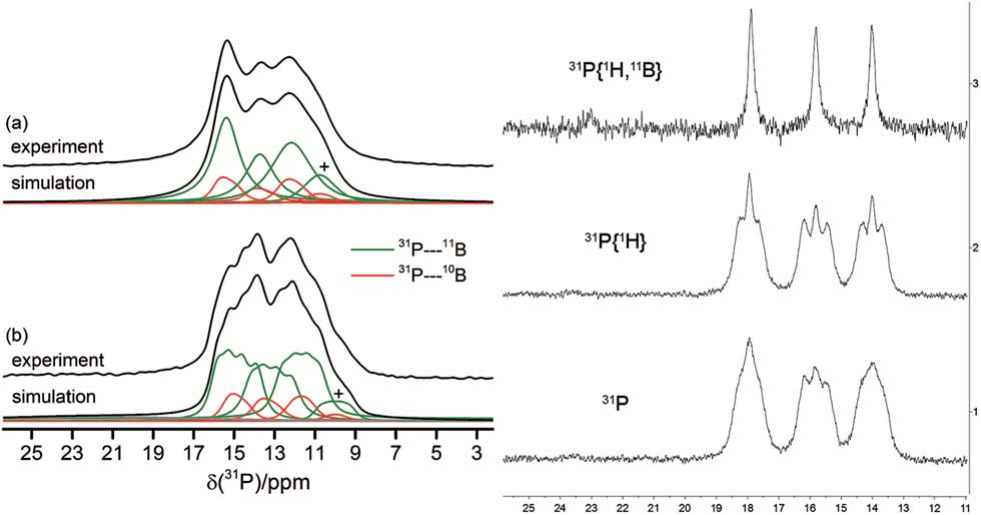
^31^P MAS NMR spectra obtained for the *C*_1_-symmetric macrocyclic trimer **18c**. Left: (a) ^1^H → ^31^P{^1^H, ^11^B} CP/MAS NMR spectrum of **18c**, (b) ^31^P{^1^H} MAS NMR spectrum and corresponding simulations, on the indirect ^31^P–^11^B and/or ^31^P–^10^B spin–spin interactions. The symbol + marks a suspected impurity. Right: ^31^P, ^31^P{^1^H}, and ^31^P{^1^H, ^11^B} NMR spectra of compound **18c** in solution (CD_2_Cl_2_, 203 K).

The solid-state ^31^P MAS NMR spectra confirmed the asymmetric (*C*_1_) structure of the cyclotrimer **18c** containing three different phosphorus atoms. Consequently, three ^31^P NMR signals at 12, 13 and 15 ppm were observed which are broadened and additionally split by the indirect ^31^P–^11^B spin–spin coupling (^1^J(^31^P–^11^B) ∼ 80 Hz). As illustrated in Fig. 5(a), simultaneous ^11^B and ^1^H decoupling enhances the resolution (see Fig. 5, left, for further details including the ^11^B{^31^P} REDOR and 2D-INEPT experiments see the ESI†). Compound **18c** also showed three equal intensity ^31^P NMR resonances in solution (Fig. 5, right). It showed 22 different ^1^H NMR signals (two with relative intensity two, all others with intensity one at 243 K) of the 12 pairs of diastereotopic CH_2_ hydrogen atoms as well as 12 methylene ^13^C NMR signals of the core ring carbons. There are nine separate ^1^H NMR *t*-Bu singlets and the ^19^F NMR signals of 12 C_6_F_5_ groups at the boron atoms of compound **18c**. The ^13^CO derived isotopologue showed three ^13^C NMR carbonyl signals [*δ* 273.3 (d, ^2^*J*_PC_ = 23.6 Hz), 272.0 (d, ^2^*J*_PC_ = 29.9 Hz) and 271.9 (d, ^2^*J*_PC_ = 29.9 Hz)] (for details see the ESI†).

## Conclusions

It seems that the presence of the additional B(C_6_F_5_)_2_ Lewis acid function influences the reaction of the internal ethylene-bridged P/B FLP functionality of the compounds **11** in two decisive ways: activation of the carbonyl group at the stage of the conventional cooperative P/B CO addition intermediate **16A**^35^,^36^ by the reactive boron Lewis acid probably makes the CO insertion reaction into the adjacent –CH_2_–B(C_6_F_5_)_2_ group feasible. Our DFT analysis of the CO insertion step of the Mes*P/B/B system revealed an exergonic (Δ*G ca.* –4 kcal mol^–1^) formation of the intermediate **16Bc** (the intermediate analogous to **16Bb** in the Tipp system shown in Scheme 4). In contrast the hypothetical CO insertion reaction from compound **7** (see Scheme 1) was computed by the DFT calculations as markedly endergonic [Δ*G ca.* +9 kcal mol^–1^, rel. Δ*G*(**7**) = 0]. Once the carbonyl compound is formed by C–C bond formation it is prone to rearrangement generating a monomeric intermediate **16C** featuring both an organic carbonyl function and a remote free –B(C_6_F_5_)_2_ Lewis acid, a combination which paves the way to formation of the unique macrocyclic oligomers **17** and **18** by Lewis adduct formation between these pairs of functional groups.

Why are the macrocyclic dimers and even a cyclotrimer formed in our examples instead of the alternative linear oligomers? Actually, we do not know for sure, but we may speculate that this has to do with the special properties encountered in phosphane/borane frustrated Lewis pair chemistry. This chemistry is governed by van der Waals interactions between the bulky protagonists and it becomes increasingly apparent that conformational features strongly determine frustrated Lewis pair behavior.^35^,^36^,^50^ In our case it might be a combination of both factors that serves to tip the balance toward cyclooligomer formation. The conformational influence is probably indicated by the different behavior of the (Dmesp)P and (Tipp)P containing FLP pairs **11a,b***vs.* the Mes*P derived system **11c** in the carbonylation/cyclooligomerization reaction. The former systems feature rather normal steric features of the bulky aryl-P linkage, whereas the latter shows the special conformational feature of the uncommon strongly bent P-aryl moiety.^46^ Our DFT analysis points to an energetic difference in the formation of the observed dimer (**17b**) in the Tipp substituted system *vs.* the cyclotrimer (**18c**) in the case of the Mes* containing system: in the Tipp containing system we find an energetic preference of the formation of the cyclodimer of *ca.* 5 kcal mol^–1^ over the trimer, whereas in the case of the more bulky Mes* system this becomes reversed and the cyclotrimer is favored by *ca.* 10 kcal mol^–1^ over the dimer (see the ESI† for details). The favored formation of the unusual macrocyclic trimer **18c** might indeed point to a marked influence of specific conformational features introduced by the very bulky aryl Mes* substituent into this chemistry.

The formation of the macrocyclic dimers and trimers from our carbonylated P/B/B FLP systems may place some frustrated Lewis pair reactions into the group of macrocyclic ring closure procedures that show a “natural” tendency of favoring the internal bond formation in cases of a suitable general design.^20^–^26^ This unique behavior of the carbonylation chemistry of the P/B/B systems **11** emphasizes the potential that frustrated Lewis pair chemistry has for discovering surprisingly facile pathways to unusual products formed under mild reaction conditions.

## Conflicts of interest

There are no conflicts to declare.

## Supplementary Material

Supplementary information

Crystal structure data

## References

[cit1] Gloe K., Macrocyclic Chemistry: Current Trends and Future Perspective, Springer, 2005.

[cit2] Yudin A. K. (2015). Chem. Sci..

[cit3] Driggers E. M., Hale S. P., Lee J., Terrett N. K. (2008). Nat. Rev. Drug Discovery.

[cit4] Marsault E., Peterson M. L. (2011). J. Med. Chem..

[cit5] Mallinson J., Collins I. (2012). Future Med. Chem..

[cit6] Villar E. A., Beglov D., Chennamadhavuni S., Porco J. A., Kozakov D., Vajda S., Whitty A. (2014). Nat. Chem. Biol..

[cit7] Allen S. E., Dokholyan N. V., Bowers A. A. (2016). ACS Chem. Biol..

[cit8] Kellogg R. M. (1984). Angew. Chem., Int. Ed. Engl..

[cit9] Pedersen C. J. (1988). Angew. Chem., Int. Ed. Engl..

[cit10] Davis F. and Higson S., Macrocycles: Construction, Chemistry and Nanotechnology Applications, Wiley-VCH Verlag GmbH, 2011.

[cit11] Li J., Yim D., Jang W.-D., Yoon J. (2017). Chem. Soc. Rev..

[cit12] Liu Z., Nalluri S. K. M., Stoddart J. F. (2017). Chem. Soc. Rev..

[cit13] Rossa L., Vögtle F. (1983). Top. Curr. Chem..

[cit14] Rodgers S. J., Ng C. Y., Raymond K. N. (1985). J. Am. Chem. Soc..

[cit15] Malesevic M., Strijowski U., Bächle D., Sewald N. (2004). J. Biotechnol..

[cit16] Cort A. D., Ercolani G., Mandolini L., Mencarelli P. (1993). J. Chem. Soc., Chem. Commun..

[cit17] Gerbeleu N. V., Arion V. B. and Burgess J. P., Template Synthesis of Macrocyclic Compounds, Wiley-VCH Verlag GmbH, 1999.

[cit18] Collins J. C., James K. (2012). Med. Chem. Commun..

[cit19] Martí-Centelles V., Pandey M. D., Burguete M. I., Luis S. V. (2015). Chem. Rev..

[cit20] Fürstner A., Langemann K. (1996). J. Org. Chem..

[cit21] Fürstner A., Thiel O. R., Blanda G. (2000). Org. Lett..

[cit22] Fürstner A., Thiel O. R., Ackermann L. (2001). Org. Lett..

[cit23] Lee C. W., Grubbs R. H. (2001). J. Org. Chem..

[cit24] Gradillas A., Pérez-Castells J. (2006). Angew. Chem., Int. Ed..

[cit25] Marx V. M., Herbert M. B., Keitz B. K., Grubbs R. H. (2013). J. Am. Chem. Soc..

[cit26] Shen X., Nguyen T. T., Koh M. J., Xu D., Speed A. W. H., Schrock R. R., Hoveyda A. H. (2017). Nature.

[cit27] Zhang W., Moore J. S. (2006). Angew. Chem., Int. Ed..

[cit28] White C. J., Yudin A. K. (2011). Nat. Chem..

[cit29] Brown H. C. (1961). Tetrahedron.

[cit30] Brown H. C., Singaram B. (1987). Pure Appl. Chem..

[cit31] Brown H. C., Ramachandran P. V. (1991). Pure Appl. Chem..

[cit32] Brown H. C., Acc. Chem. Res., 1969, 2 , 65 –72 , . See also: Hair G. S. Jones R. A. Cowley A. H. Lynch V., Organometallics, 2001, 20 , 177 –181 .

[cit33] Parks D. J., Spence R. E. v. H., Piers W. E. (1995). Angew. Chem., Int. Ed. Engl..

[cit34] Parks D. J., Piers W. E., Yap G. P. A. (1998). Organometallics.

[cit35] Sajid M., Lawzer A., Dong W., Rosorius C., Sander W., Schirmer B., Grimme S., Daniliuc C. G., Kehr G., Erker G. (2013). J. Am. Chem. Soc..

[cit36] Elmer L.-M., Kehr G., Daniliuc C. G., Siedow M., Eckert H., Tesch M., Studer A., Williams K., Warren T. H., Erker G. (2017). Chem.–Eur. J..

[cit37] Cardenas A. J. P., Culotta B. J., Warren T. H., Grimme S., Stute A., Fröhlich R., Kehr G., Erker G. (2011). Angew. Chem., Int. Ed..

[cit38] Sajid M., Stute A., Cardenas A. J. P., Culotta B. J., Hepperle J. A. M., Warren T. H., Schirmer B., Grimme S., Studer A., Daniliuc C. G., Fröhlich R., Petersen J. L., Kehr G., Erker G. (2012). J. Am. Chem. Soc..

[cit39] Ekkert O., Miera G. G., Wiegand T., Eckert H., Schirmer B., Petersen J. L., Daniliuc C. G., Frohlich R., Grimme S., Kehr G., Erker G. (2013). Chem. Sci..

[cit40] Wang L., Samigullin K., Wagner M., McQuilken A. C., Warren T. H., Daniliuc C. G., Kehr G., Erker G. (2016). Chem.–Eur. J..

[cit41] Saednya A., Hart H. (1996). Synthesis.

[cit42] Chandrasekhar V., Sasikumar P., Boomishankar R., Anantharaman G. (2006). Inorg. Chem..

[cit43] Yakhvarov D. G., Hey-Hawkins E., Kagirov R. M., Budnikova Y. H., Ganushevich Y. S., Sinyashin O. G. (2007). Russ. Chem. Bull..

[cit44] Overländer C., Tirrée J. J., Nieger M., Niecke E., Moser C., Spirk S., Pietschnig R. (2007). Appl. Organomet. Chem..

[cit45] Rivard E., Power P. P. (2007). Inorg. Chem..

[cit46] Ito S., Miyake H., Yoshifuji M. (2009). Phosphorus, Sulfur, and Silicon and the Related Elements.

[cit47] Möbus J., Bonnin Q., Ueda K., Fröhlich R., Itami K., Kehr G., Erker G. (2012). Angew. Chem., Int. Ed..

[cit48] Hasegawa Y., Kehr G., Ehrlich S., Grimme S., Daniliuc C. G., Erker G. (2014). Chem. Sci..

[cit49] Zhao X., Lough A. J., Stephan D. W. (2011). Chem.–Eur. J..

[cit50] Spies P., Erker G., Kehr G., Bergander K., Frohlich R., Grimme S., Stephan D. W. (2007). Chem. Commun..

[cit51] Stephan D. W., Erker G. (2013). Top. Curr. Chem..

[cit52] Stephan D. W., Erker G. (2013). Top. Curr. Chem..

[cit53] Stephan D. W., Erker G. (2015). Angew. Chem., Int. Ed..

[cit54] Stephan D. W. (2016). Science.

[cit55] Axenov K. V., Mömming C. M., Kehr G., Fröhlich R., Erker G. (2010). Chem.–Eur. J..

[cit56] Chernichenko K., Madarász Á., Pápai I., Nieger M., Leskelä M., Repo T. (2013). Nat. Chem..

[cit57] Sajid M., Kehr G., Wiegand T., Eckert H., Schwickert C., Pöttgen R., Cardenas A. J. P., Warren T. H., Fröhlich R., Daniliuc C. G., Erker G. (2013). J. Am. Chem. Soc..

[cit58] Chen G.-Q., Kehr G., Daniliuc C. G., Mück-Lichtenfeld C., Erker G. (2016). Angew. Chem., Int. Ed..

[cit59] Perutz R. N., Sabo-Etienne S. (2007). Angew. Chem., Int. Ed..

[cit60] Waterman R. (2013). Organometallics.

[cit61] Wang Y., Chen W., Lu Z., Li Z. H., Wang H. (2013). Angew. Chem., Int. Ed..

[cit62] Klamt A., Schüürmann G. (1993). J. Chem. Soc., Perkin Trans. 2.

[cit63] Klamt A. (1995). J. Phys. Chem..

[cit64] Weigend F., Ahlrichs R. (2005). Phys. Chem. Chem. Phys..

[cit65] Zhao Y., Truhlar D. G. (2005). J. Phys. Chem. A.

[cit66] Weigend F. (2006). Phys. Chem. Chem. Phys..

[cit67] Grimme S., Antony J., Ehrlich S., Krieg H. (2010). J. Chem. Phys..

[cit68] Grimme S., Ehrlich S., Goerigk L. (2011). J. Comput. Chem..

[cit69] Grimme S., Brandenburg J. G., Bannwarth C., Hansen A. (2015). J. Chem. Phys..

[cit70] Jacobsen H., Berke H., Döring S., Kehr G., Erker G., Fröhlich R., Meyer O. (1999). Organometallics.

[cit71] Vagedes D., Fröhlich R., Erker G. (1999). Angew. Chem., Int. Ed..

[cit72] Dutartre M., Bayardon J., Juge S. (2016). Chem. Soc. Rev..

[cit73] Verkade J. P. and Qinn L. D., Phosphorus-31 NMR Spectroscopy in Stereochemical Analysis, Organic Compounds and Metal Complexes, Wiley-VCH Verlag GmbH, 1987.

