# Supplementary material for: Formation of macrocyclic ring systems by carbonylation of trifunctional P/B/B frustrated Lewis pairs
Source: Chem Sci. 2017 Dec 14;9(6):1544–50. doi: 10.1039/c7sc04394e (PMC5890323; doi:10.1039/c7sc04394e)
Supplement: Supplementary file 1 [file SC-009-C7SC04394E-s001.pdf]

Electronic Supplementary Material (ESI) for Chemical Science.
This journal is © The Royal Society of Chemistry 2018

Formation of Macrocyclic Ring Systems by Carbonylation of Trifunctional P/B/B Frustrated Lewis Pairs

Long Wang,[a] Shunxi Dong,[a] Constantin G. Daniliuc,[a] Lei Liu,[b] Stefan Grimme,[b] Robert Knitsch,[c] Hellmut Eckert,[c,d] Michael Ryan Hansen,[c] Gerald Kehr,[a] Gerhard Erker[a]

Supplementary Information

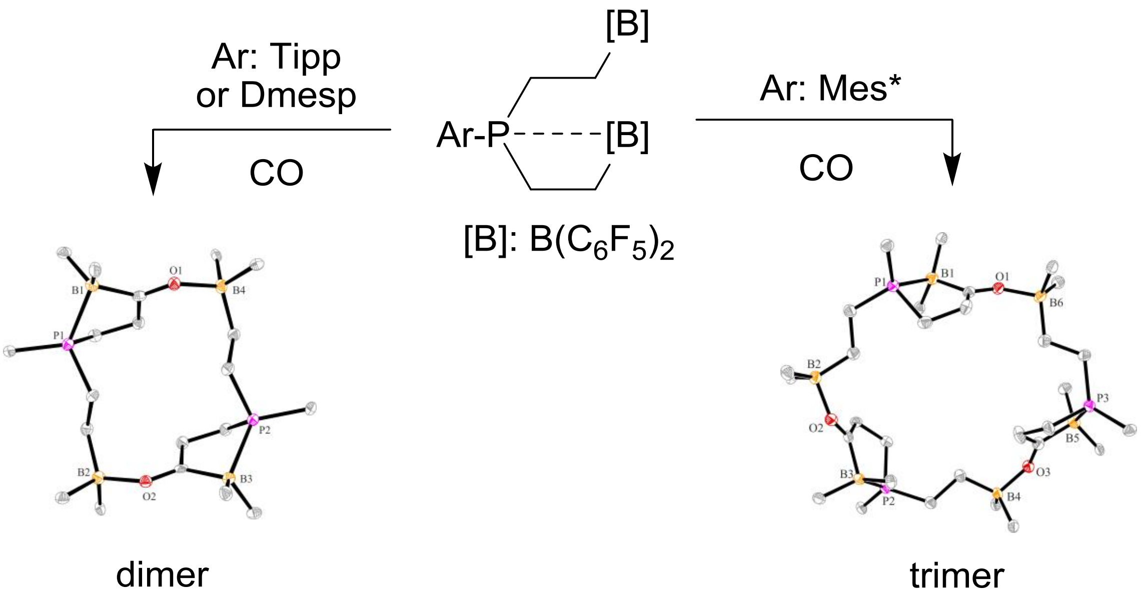

Table of Contents

**Part 1. Experiment and Analytical Details)**	S3
General information	S3
Preparation of compound **3**	S4
Reaction of compound **3** with CO gas	S4
Preparation of Compound **8a**	S6
In situ generation of compound **11a**	S8
Synthesis of compound **14a**	S11
Characterization of compound **15py**	S15
Synthesis of compound **14b**	S18
In situ reaction of compound **14b** (*in situ* generated) with H_2	S22
Synthesis of compound **14c**	S24
Preparation of compound **17a**	S28
Preparation of ^{13}C labeled compound **17a**	S34
Preparation of compound **17b**	S37
Preparation of ^{13}C labeled compound **17b**	S43
In situ generation of compound **18c**	S46
In situ generation of ^{13}C labeled compound **18c**	S52
Preparation of compound **18c**	S54
Stability of compound **18c** in dichloromethane-d_2 solution	S57
Reaction of compound **18c** with pyridine	S60
Stability of compound **17a** in dichloromethane-d_2 solution at 50 ^oC	S63
Stability of compound **17b** in benzene-d_6 at 80 ^oC	S66
**Part 2. Computational Details**	S67
**Part 3. Solid State NMR Experiments**	S95
Experimental Procedures	S95
Results and Discussion	S96
**References**	S108

Part 1: Experimental and Analytical Details

Formation of Macrocyclic Ring Systems by Carbonylation of Trifunctional P/B/B Frustrated Lewis Pairs

Long Wang,[a] Shunxi Dong,[a] Constantin G. Daniliuc,[a] Gerald Kehr,[a] Gerhard Erker[a]

[a] Organisch-Chemisches Institut, Westfälische Wilhelms-Universität Münster, Correns-strasse 40, 48149 Münster, Germany

General Information

All experiments were carried out in a dry argon atmosphere using an MBraun glove box and/or standard Schlenk techniques. All solvents were dried and stored under an argon atmosphere before use. NMR spectra were measured on a Varian INOVA 500 MHz spectrometer (^{1}H 500 MHz; ^{13}C, 126 Hz; ^{11}B, 160 MHz; ^{19}F, 470 Hz) or a Varian UNITY plus 600 MHz spectrometer (^{1}H 600 MHz; ^{13}C, 151 Hz; ^{11}B, 192 MHz; ^{19}F, 564 Hz). Chemical shifts are given relative to $SiMe_4$ and referenced to the respective residual solvent signal (^{1}H NMR and ^{13}C NMR) or an external standard [δ ($BF_3{\cdot}OEt_2$) = 0 for ^{11}B NMR, δ ($CFCl_3$) = 0 for ^{19}F NMR]. NMR assignments were supported by additional 1D and 2D NMR experiments. IR were measured on Varian 1300 FT-IR and melting points were measured on TA-instruments DSC Q-20.
Unless otherwise noted, all chemicals were purchased from commercially available sources. Compound **2** were prepared according to the literature.[1,2] We prepared the aryldivinylphosphanes **8a-c** by treatment of the respective $ArPCl_2$ precursors with two molar equiv. of vinyl magnesium bromide.[3-9] We had shown that the P/B/B system **11c** splits dihydrogen in the presence of the external base $^{t}Bu_3P$ to give **12c**.[10]

X-Ray diffraction: For compounds **14c** and **18c** data sets were collected with a Nonius Kappa CCD diffractometer. Programs used: data collection, COLLECT (R. W. W. Hooft, Bruker AXS, **2008**, Delft, The Netherlands); data reduction Denzo-SMN (Z. Otwinowski, W. Minor, *Methods Enzymol.* **1997**, *276*, 307-326); absorption correction, Denzo (Z. Otwinowski, D. Borek, W. Majewski, W. Minor, *Acta Crystallogr*. **2003**, *A59*, 228-234); structure solution SHELXS-97 (G. M. Sheldrick, *Acta Crystallogr*. **1990**, *A46*, 467-473); structure refinement SHELXL-97 (G. M. Sheldrick, *Acta Crystallogr*. **2008**, *A64*, 112-122). For compound **14b** data sets were collected with a Bruker APEX II CCD diffractometer. Data sets for the compounds **14a**, **17a** and **17b** were collected with a D8 Venture Dual Source 100 CMOS diffractometer. Programs used for compound **14a**: data collection: APEX3 V2016.1-0 (Bruker AXS Inc., **2016**); cell refinement: SAINT V8.37A (Bruker AXS Inc., **2015**); data reduction: SAINT V8.37A (Bruker AXS Inc., **2015**); absorption correction, SADABS V2014/7 (Bruker AXS Inc., **2014**); structure solution SHELXT-2015 (Sheldrick, **2015**); structure refinement SHELXL-2015 (Sheldrick, **2015**) and graphics, XP (Bruker AXS Inc., **2015**). *R*-values are given for observed reflections, and *w*R2 values are given for all reflections. *Exceptions and special features*: For compound **14a** one and a half badly disordered dichloromethane molecules, for compound **17a** a badly disordered dichloromethane molecule and for compound **18c** one toluene molecule were found in the asymmetrical unit and could not be satisfactorily refined. The program SQUEEZE (Spek, A.L. (**2015**). Acta Cryst. C71, 9-18) was therefore used to remove mathematically the effect of the solvent. The quoted formula and derived parameters are not included the squeezed solvent molecules.
CCDC deposition numbers are 1549697 to 1549702.

Preparation of compound 3

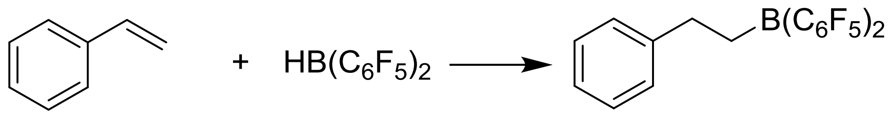

Scheme S1.

A solution of styrene **1** (145.8 mg, 1.4 mmol, 1.4 eq.) in pentane (6 mL) was added to a suspension of $HB(C_6F_5)_2$ **2** (345.9 mg, 1.0 mmol, 1 eq.) in pentane (2 mL) at room temperature. While stirring the reaction suspension became a clear solution and then a suspension. The precipitate was collected by filtration, then it was washed with cold pentane (2 mL×2, -30 °C) to give compound **3** as a white solid (320.0 mg, 0.71 mmol, yield 71 %).[11]

Reaction of compound 3 with CO gas

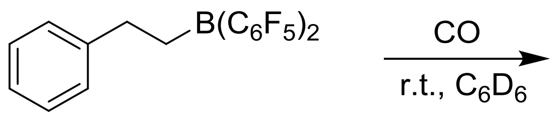

Scheme S2.

A solution of compound **3** (22.5 mg, 0.05 mmol) in C_6D_6 (1 mL) was filled in a J-Young tube, which was cooled to -78 °C (dry ice / isopropanol bath) and then evacuated carefully. Then the reaction mixture was exposed to CO gas (1.5 bar) at room temperature. After storing for 24 h at room temperature, the mixture was characterized by NMR experiments.

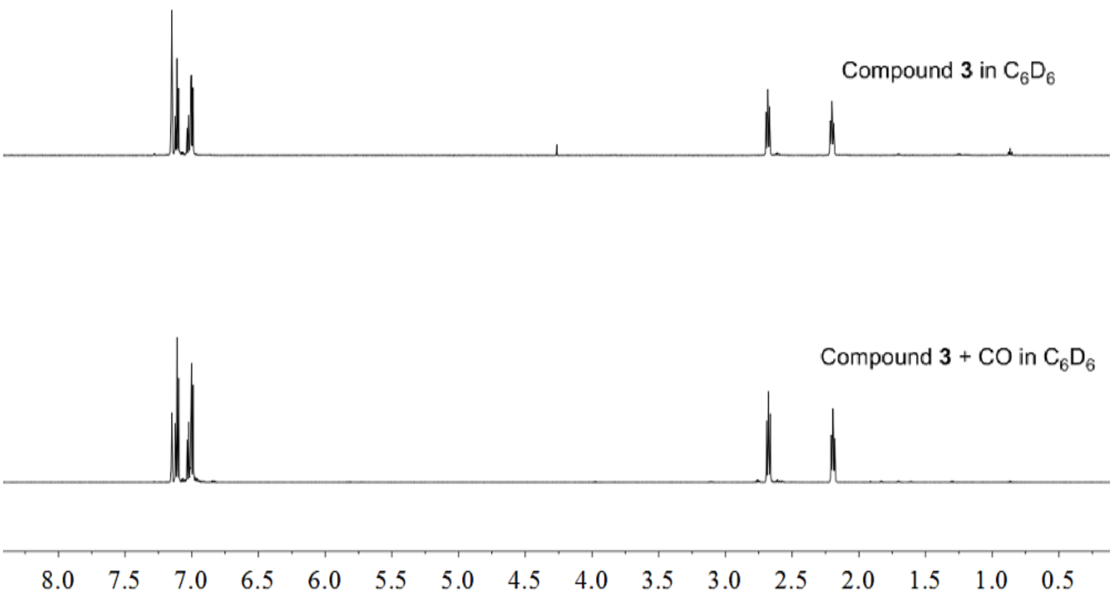

Figure S1. **^{1}H NMR** (600 MHz, 299 K, C_6D_6) spectra of compound **3** (top) and compound **3** + CO (bottom)

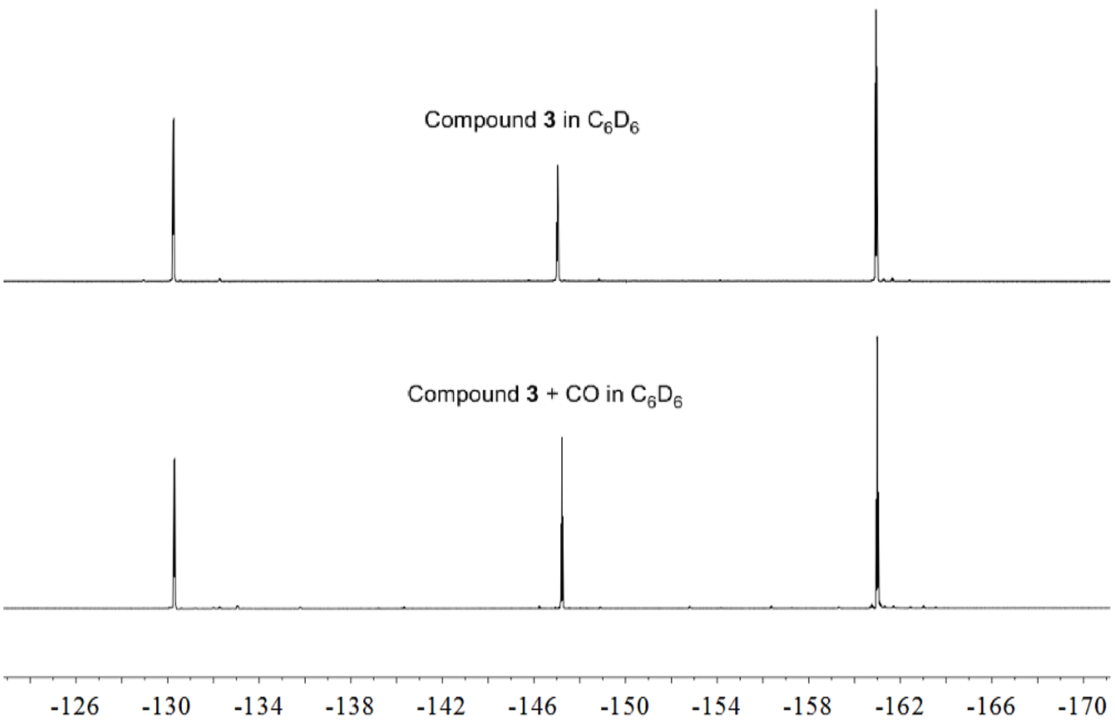

Figure S2. **^{19}F NMR** (564 MHz, 299 K,C_6D_6) spectra of compound **3** (top) and compound **3** + CO (bottom)

Preparation of Compound 8a[4]

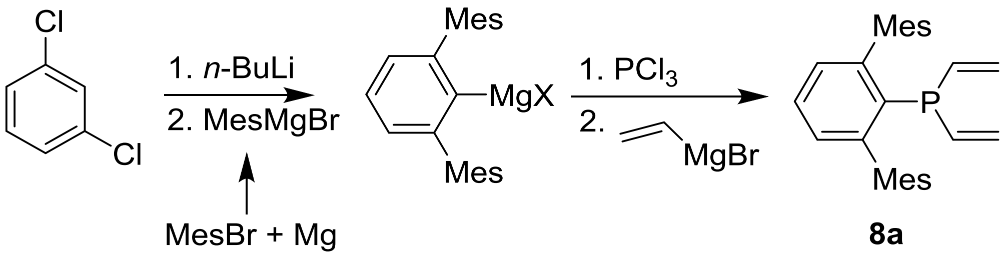

Scheme S3.

In a flame dried Schlenk flask, a solution of 1,3-dichlorobenzene (2.35 g, 16 mmol, 1.0 eq.) in THF (30 mL) was cooled to -78 ^{o}C in a dry ice / isopropanol bath. *n*-BuLi (10 mL of a 1.6 M solution in *n*-hexane, 16 mmol, 1.0 eq.) was added dropwise and stirred at -78 ^{o}C for 2 h. A solution of MesMgBr (32 mmol, 2.0 eq.) in THF (40 mL), freshly prepared from magnesium (0.78 g, 32 mmol, 2.0 eq.) and mesityl bromide (6.37 g, 32 mmol, 2.0 eq.), was then added dropwise and stirred at -78 ^{o}C for 2 h . The solution was allowed to warm to room temperature and stirring continued for 12 h. The resulting solution was heated to reflux and stirred for 2 h. After cooling the mixture to -78 ^{o}C, PCl_3 (1.4 mL, 16 mmol, 1.0 eq.) was added and the reaction mixture was allowed to warm to room temperature and stirred for 24 h. After cooling the mixture to -78 ^{o}C again, vinylmagnesium bromide (32 mL of 1 M solution in THF, 32 mmol, 2.0 eq.) was added. The mixture was allowed to warm to room temperature and stirred at room temperature for 48 h. After removal of all volatiles in vacuo, the resulting mixture was extracted with pentane (100 mL × 3). The combined extraction was concentrated and purified by silica gel chromatography (pentane : dichloromethane : triethylamine = 200:10:1) to give compound **8a** as a white solid (3.57 g, 8.96 mmol, 56 %).
HRMS: m/z calc. for $C_{28}H_{31}P$+[H^+] 399.2236; found 399.2236.
NMR data from a solution of the white solid in dichloromethane-d_2.
^{1}H NMR (500 MHz, 299 K, dichloromethane-d_2) δ = 7.41 (t, $^3J_{HH}$ = 7.5 Hz, 1H, *p*-C_6H_3), 6.96 (dd, $^3J_{HH}$ = 7.5 Hz, $^4J_{PH}$ = 2.2 Hz, 2H, *m*- C_6H_3), 6.88 (s, 4H, *m*-Mes), 5.77 (ddd, $^3J_{HH}{}^{trans}$ = 18.3 Hz, $^3J_{HH}{}^{cis}$ = 11.6 Hz, $^2J_{PH}$ = 7.7 Hz, 1H, =CH), 5.32 (ddd, $^3J_{PH}$ = 35.5 Hz, $^3J_{HH}{}^{cis}$ = 11.6 Hz, $^2J_{HH}$ = 2.2 Hz, 1H, =$CH_2{}^E$), 5.21 (ddd, $^3J_{HH}{}^{trans}$ = 18.3 Hz, $^3J_{PH}$ = 15.0 Hz, $^2J_{HH}$ = 2.2 Hz, 1H, =$CH_2{}^Z$), 2.33 (s, 6H, *p*-$CH_3{}^{Mes}$), 1.96 (s, 12H, *o*-$CH_3{}^{Mes}$).
^{13}C{^{1}H} NMR (126 MHz, 299 K, dichloromethane-d_2) δ = 147.1 (d, $^2J_{PC}$ = 16.4 Hz, *o*-C_6H_3), 140.1 (d, $^3J_{PC}$ = 4.7 Hz, *i*-Mes), 137.1 (*p*-Mes), 136.3 (d, J_{PC} = 1.1 Hz, *o*-Mes), 136.0 (d, $^1J_{PC}$ = 13.6 Hz, =CH), 134.9 (d, $^1J_{PC}$ = 19.1 Hz, *i*-C_6H_3), 129.80 (*p*-C_6H_3), 129.77 (*m*-C_6H_3), 128.0 (*m*-Mes), 126.9 (d, $^2J_{PC}$ = 31.8 Hz, =CH_2), 21.3 (d, J_{PC} = 2.0 Hz, *o*-$CH_3{}^{Mes}$), 21.2 (*p*-$CH_3{}^{Mes}$).
^{31}P{^{1}H} NMR (202 MHz, 299 K, dichloromethane-d_2) δ = -19.1 ($\nu_{1/2}$ ~ 1 Hz).

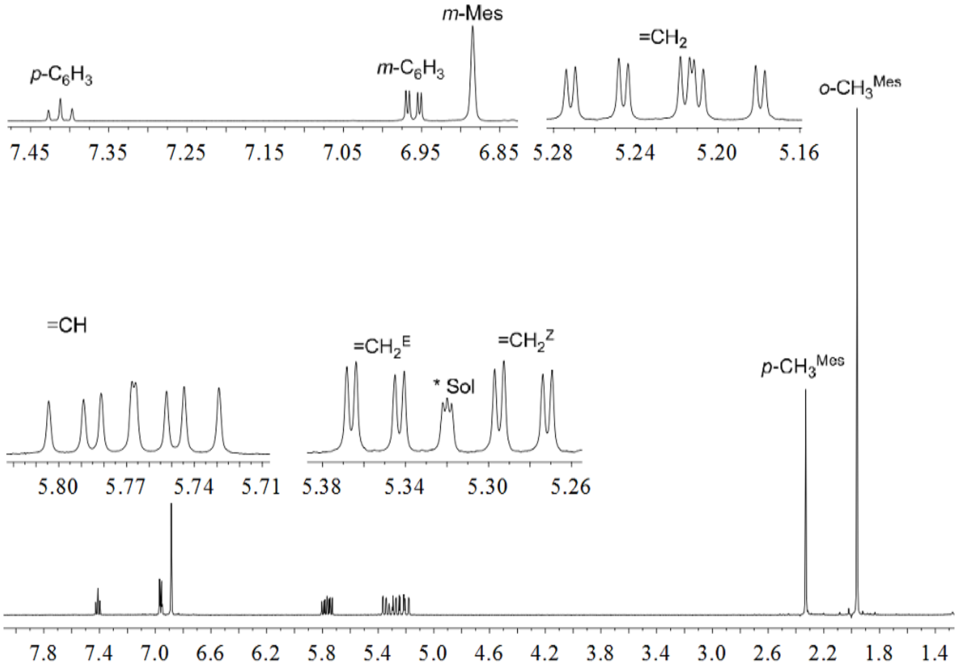

Figure S3. ^{1}H NMR (500 MHz, 299 K, dichloromethane-d_2) spectrum of compound **8a**

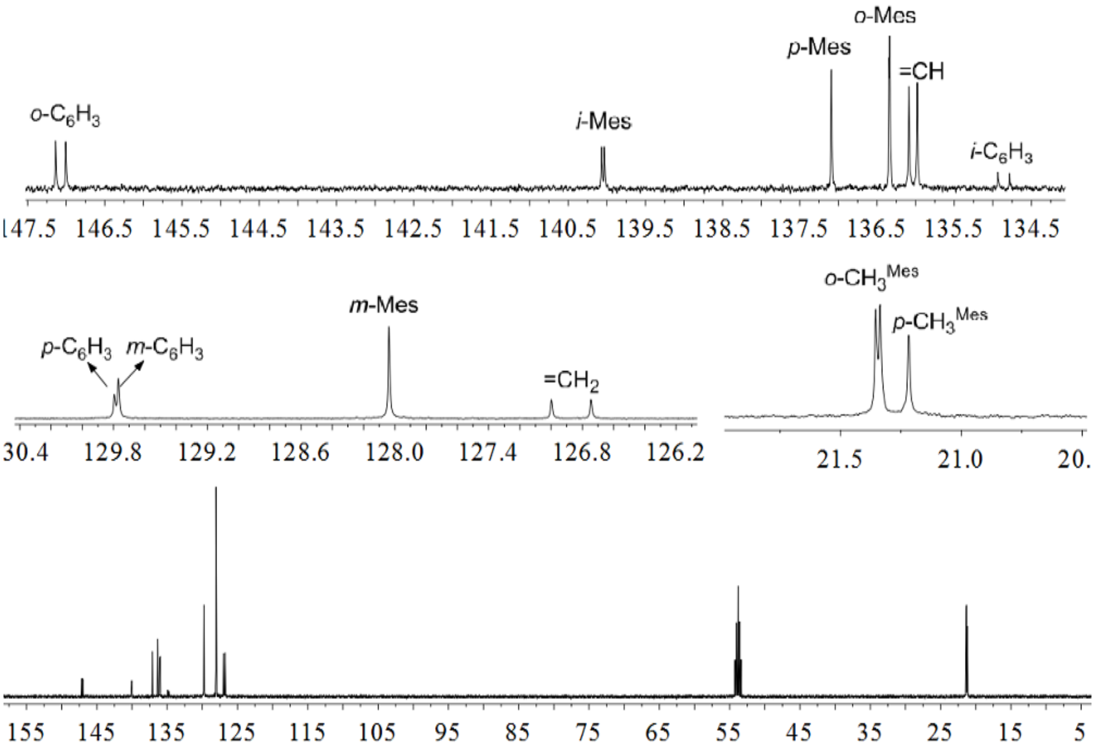

Figure S4. **$^{13}C\{^1H\}$ NMR** (126 MHz, 299 K, dichloromethane-d_2) spectrum of compound **8a**

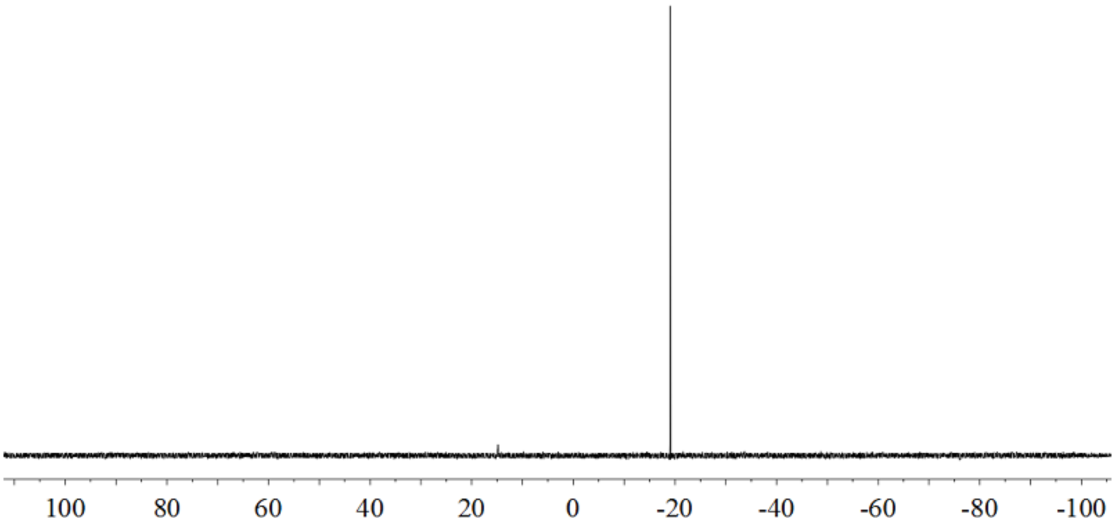

Figure S5. **$^{31}P\{^1H\}$ NMR** (202 MHz, 299 K, dichloromethane-d_2) spectrum of compound **8a**

In situ generation of compound 11a

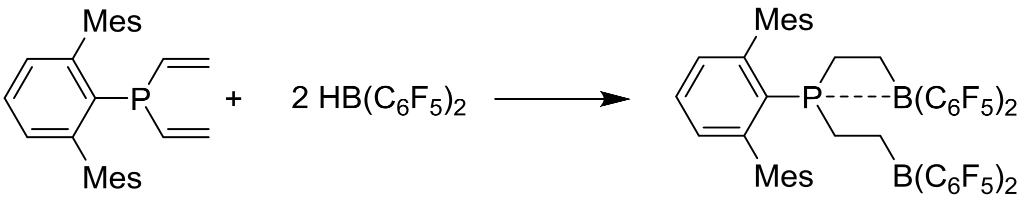

8a **2** **11a**

Scheme S4.

In the glove box, compound **8a** (19.9 mg, 0.05 mmol, 1.0 eq.) and compound **2** (34.6 mg, 0.10 mmol, 2.0 eq.) were mixed with dichloromethane-d_2 (1 mL) to give a yellow solution. The yellow solution was characterized by NMR experiments.

^{1}H NMR (600 MHz, 299 K, dichloromethane-d_2) δ = 7.55 (t, $^3J_{HH}$ = 7.5 Hz, 1H, *p*-C_6H_3), 7.03 (m, 2H, *m*-C_6H_3), 6.85 (s, 4H, *m*-Mes), 2.29 (s, 6H, *p*-CH_3^{Mes}), 2.00/1.69 (each m, each 2H, PCH_2), 1.94/1.61 (each m, each 2H, BCH_2), 1.85 (s, 12H, *o*-CH_3^{Mes}),.
^{13}C{^{1}H} NMR (151 MHz, 299 K, dichloromethane-d_2) δ = 147.3 (dm, $^1J_{FC}$ ~ 245 Hz, C_6F_5), 147.0 (d, $^2J_{PC}$ = 8.3 Hz, *o*-C_6H_3), 142.2 (dm, $^1J_{FC}$ ~ 255 Hz, C_6F_5), 138.4 (*p*-Mes), 137.61 (dm, $^1J_{FC}$ ~ 250 Hz, C_6F_5), 137.57 (d, $^3J_{PC}$ = 8.3 Hz, *i*-Mes), 136.4 (*o*-Mes), 131.9 (d, $^4J_{PC}$ = 2.4 Hz, *p*-C_6H_3), 131.8 (d, $^3J_{PC}$ = 2.4 Hz, *m*-C_6H_3), 130.2 (*i*-C_6H_3), 128.6 (*m*-Mes), 116.2 (br m, i-C_6F_5), 22.9 (br, BCH_2), 21.2 (*o*-CH_3^{Mes}), 20.9 (*p*-CH_3^{Mes}), 20.5 (d, $^1J_{PC}$ = 22.4 Hz, PCH_2).
^{11}B{^{1}H} NMR (192 MHz, 299 K, dichloromethane-d_2) δ = 41.2 ($\nu_{1/2}$ ~ 1000 Hz).
^{19}F NMR (564 MHz, 299 K, dichloromethane-d_2) δ = -129.5 (m, 2F, *o*-C_6F_5), -152.7 (m, 1F, *p*-C_6F_5), -163.0 (m, 2F, *m*-C_6F_5), [$\Delta\delta^{19}F_{m,p}$ = 10.3].
^{31}P{^{1}H} NMR (243 MHz, 299 K, dichloromethane-d_2) δ = 14.0 ($\nu_{1/2}$ ~ 20 Hz).

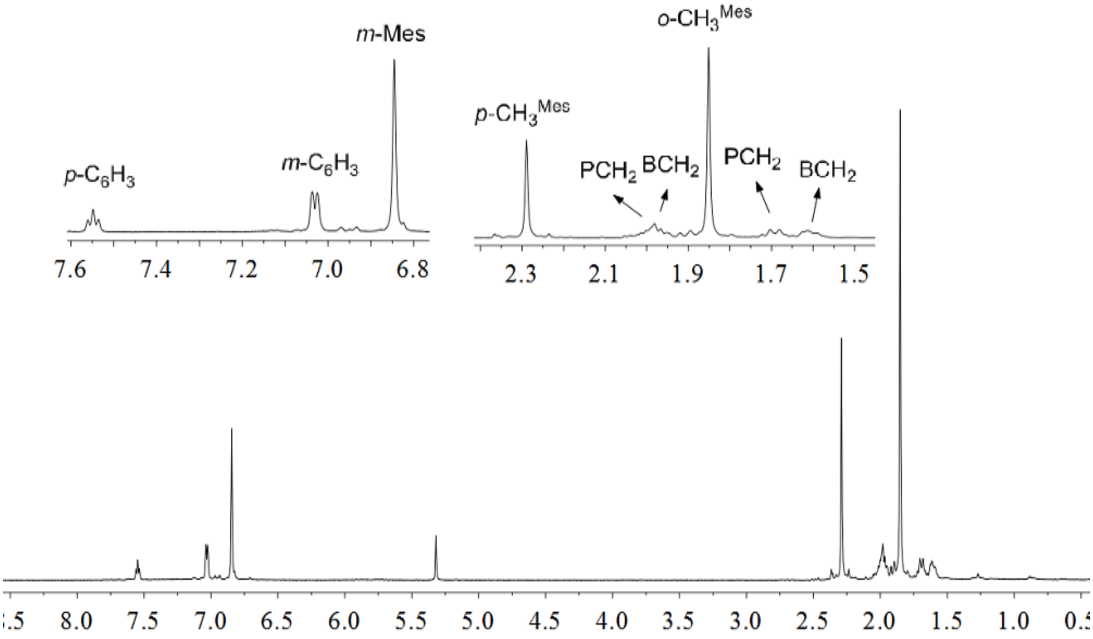

Figure S6. **^{1}H NMR** (600 MHz, 299 K, dichloromethane-d_2) spectrum of compound **11a**

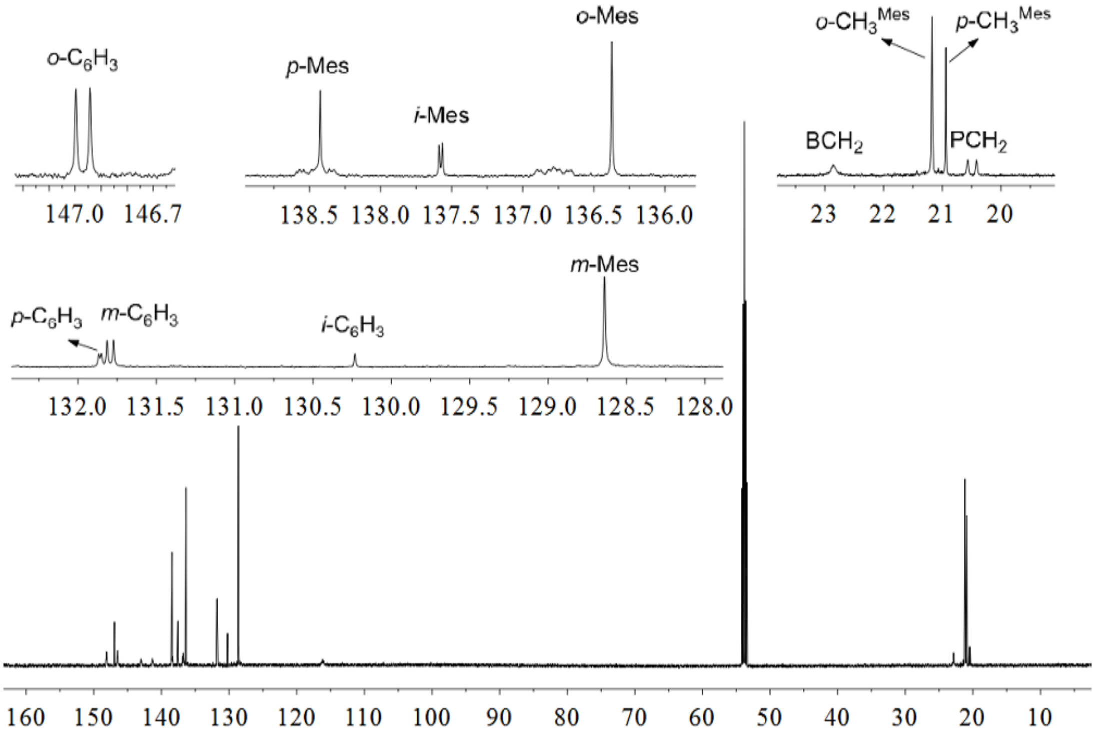

Figure S7. $^{13}C\{^{1}H\}$ **NMR** (151 MHz, 299 K, dichloromethane-d_2) spectrum of compound **11a**

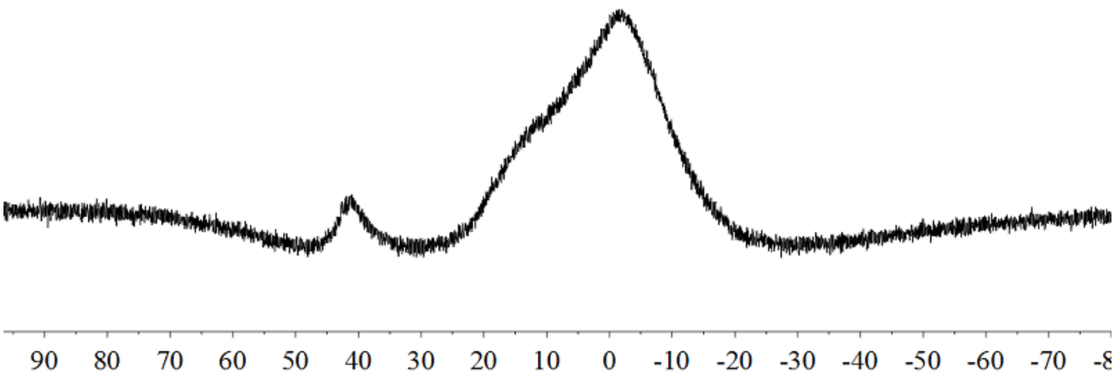

Figure S8. $^{11}B\{^{1}H\}$ **NMR** (192 MHz, 299 K, dichloromethane-d_2) spectrum of compound **11a**

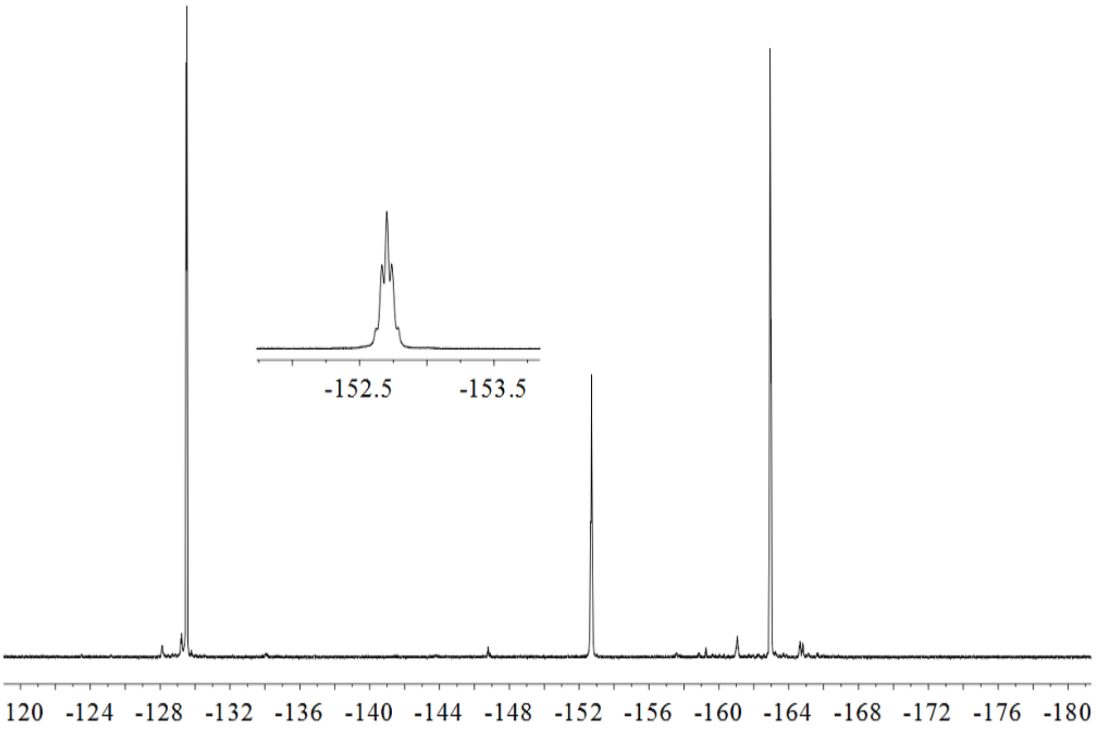

Figure S9. ^{19}**F NMR** (564 MHz, 299 K, dichloromethane-d_2) spectrum of compound **11a**

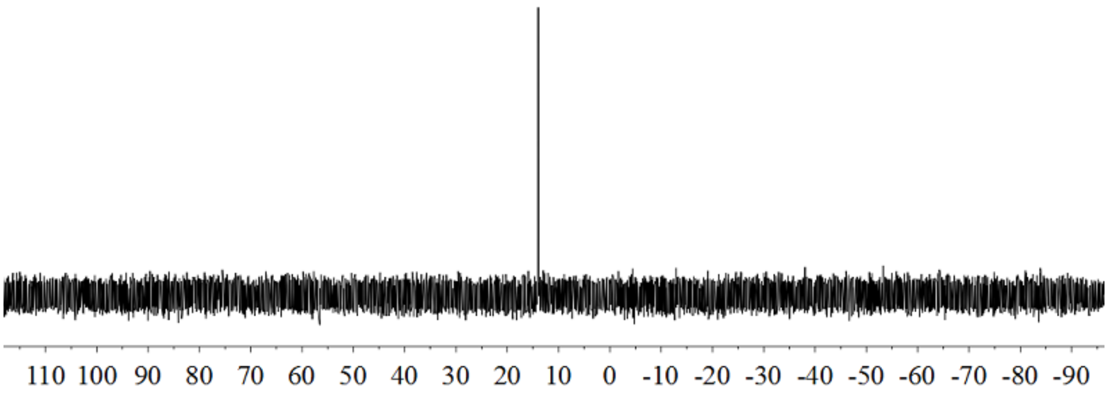

Figure S10. ^{31}**P{^{1}H} NMR** (243 MHz, 299 K, dichloromethane-d_2) spectrum of compound **11a**

Synthesis of compound 14a

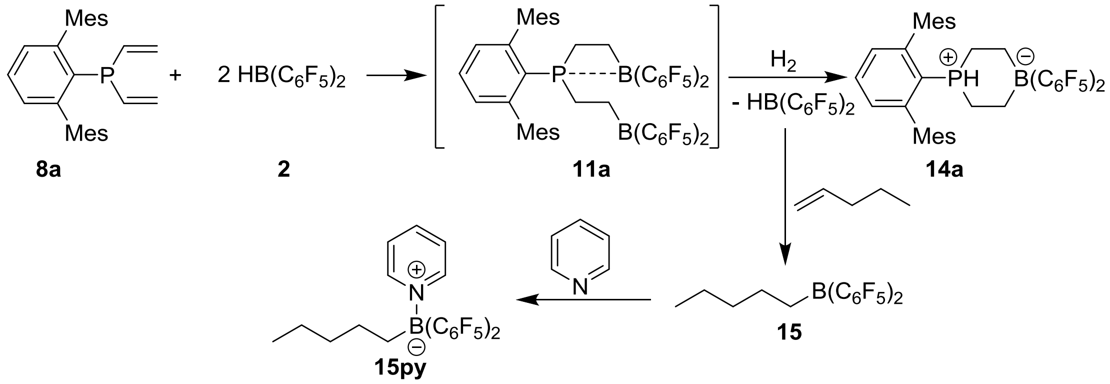

Scheme S5.

In a Schlenk flask, a solution of compound **8a** (119.6 mg, 0.30 mmol, 1.0 eq.) and compound **2** (207.6 mg, 0.6 mmol, 2.0 eq.) in dichloromethane (6 mL) was stirred for 15 min at room temperature to give a yellow solution. The solution was cooled to -78 °C (dry ice / isopropanol bath) and the flask was evacuated carefully. Then the dry ice bath was removed and the reaction mixture was exposed to H_2 gas (1.5 bar) at -78 °C. Subsequently the obtained suspension was stirred at room temperature for 30 min, then the H_2 was released under argon atmosphere followed by the addition of pentene (42.1 mg, 0.6 mmol, 2 eq.). The reaction mixture was stirred at room temperature for 2 days until the precipitate had dissolved again. Then all volatiles were removed in vacuo to give a white solid, which was washed with pentane (2 mL × 3) and dried in vacuo to give compound **14a** as a white solid (174.7 mg, 0.234 mmol, yield 78 %).
The combined washing solutions were collected and pyridine (23.7 mg, 0.3 mmol, 1.0 eq.) was added. The mixture was stored at -36 °C to give compound **15py** as a white solid (108.4 mg, 0.219 mmol, yield 73 %).

HRMS of compound 14a: m/z calc. for $C_{40}H_{34}PBF_{10}$+[Na^+] 769.2224; found 769.2231.
Melting point of compound 14a: 243 °C
NMR data of compound 14a [NMR data from a solution of the white solid in dichloromethane-d_2]
^{1}H NMR (500 MHz, 299 K, dichloromethane-d_2) δ = 7.80 (td, $^3J_{HH}$ = 7.7 Hz, $^5J_{PH}$ = 2.2 Hz, 1H, *p*-C_6H_3), 7.26 (dd, $^3J_{HH}$ = 7.7 Hz, $^4J_{PH}$ = 3.9 Hz, 2H, *m*-C_6H_3), 6.95 (m, 4H, *m*-Mes), 5.56 (dt, $^1J_{PH}$ = 463.0 Hz, $^3J_{HH}$ = 13.6 Hz, 1H, PH), 2.27 (s, 6H, *p*-CH_3^{Mes}), 1.97 (s, 12H, *o*-CH_3^{Mes}), 1.99/1.51 (each m, each 2H, PCH_2), 1.82/0.54 (each m, each 2H, BCH_2).
^{13}C{^{1}H} NMR (126 MHz, 299 K, dichloromethane-d_2) δ = 147.8 (d, $^2J_{PC}$ = 10.1 Hz, *o*-C_6H_3), 139.8 (*p*-Mes), 135.9 (*o*-Mes), 135.3 (d, $^4J_{PC}$ = 2.7 Hz, *p*-C_6H_3), 135.2 (d, $^3J_{PC}$ = 5.0 Hz, *i*-Mes), 131.2 (d, $^3J_{PC}$ = 9.1 Hz, *m*-C_6H_3), 129.2 (*m*-Mes), 118.4 (d, $^1J_{PC}$ = 77.0 Hz, *i*-C_6H_3), 21.01 (*p*-CH_3^{Mes}), 20.96 (*o*-CH_3^{Mes}), 20.8 (br, BCH_2), 19.0 (d, $^1J_{PC}$ = 43.1 Hz, PCH_2), [C_6F_5 not listed].
^{11}B{^{1}H} NMR (160 MHz, 299 K, dichloromethane-d_2) δ = -14.6 ($\nu_{1/2}$ ~ 40 Hz).
^{19}F NMR (470 MHz, 299 K, dichloromethane-d_2) δ = -133.8 (m, 2F, *o*), -163.0 (t, $^3J_{FF}$ = 20.4 Hz, 1F, *p*), -166.0 (m, 2F, *m*)(C_6F_5)[$\Delta\delta^{19}F_{m,p}$= 3.0], -134.2 (m, 2F, *o*), -163.9 (t, $^3J_{FF}$ = 20.3 Hz, 1F, *p*), -166.8 (m, 2F, *m*)(C_6F_5)[$\Delta\delta^{19}F_{m,p}$ = 2.9].
^{31}P{^{1}H} NMR (202 MHz, 299 K, dichloromethane-d_2) δ = 9.0 ($\nu_{1/2}$ ~ 10 Hz).
^{31}P NMR (202 MHz, 299 K, dichloromethane-d_2) δ = 9.0 (dm, $^1J_{PH}$ ~ 462 Hz).

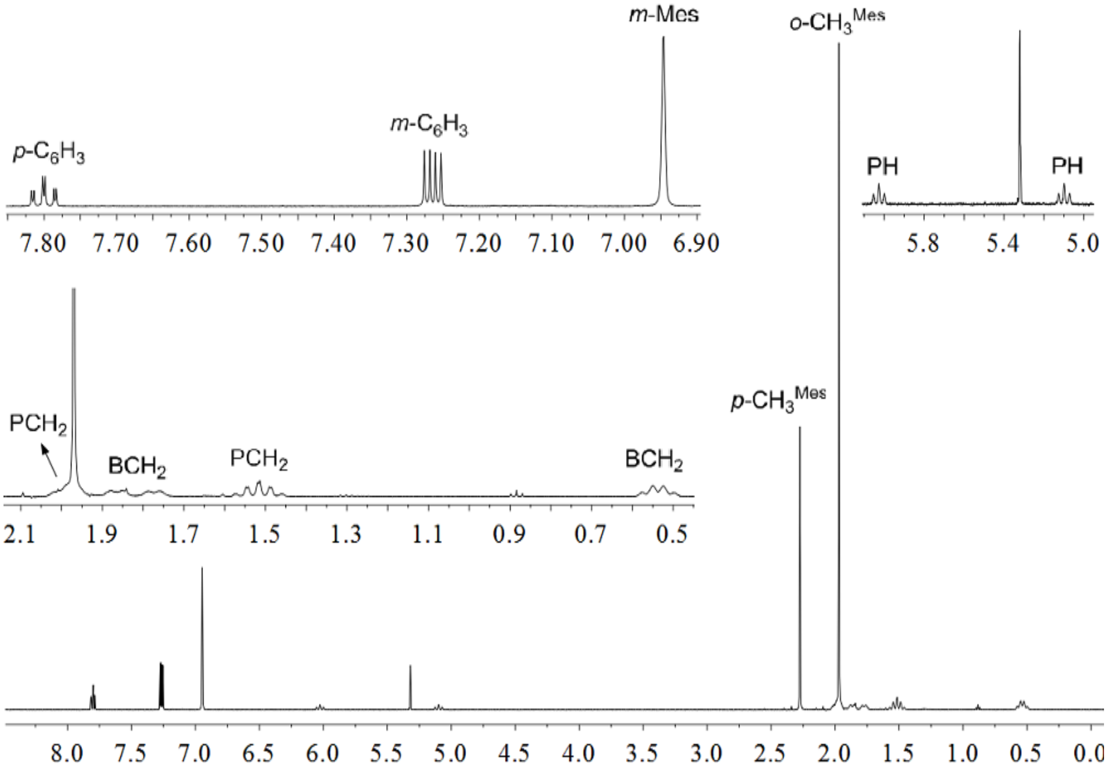

Figure S11. **^{1}H NMR** (500 MHz, 299 K, dichloromethane-d$_2$) spectrum of compound **14a**

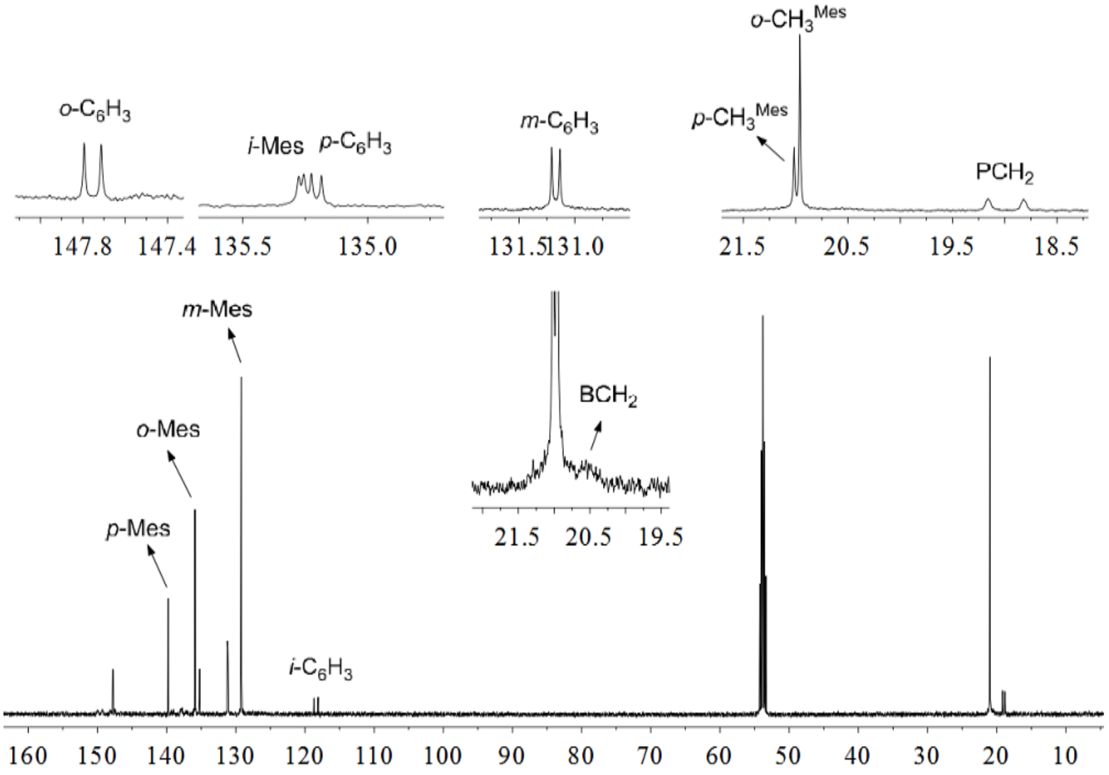

Figure S12. **^{13}C{^{1}H} NMR** (126 MHz, 299 K, dichloromethane-d$_2$) spectrum of compound **14a**

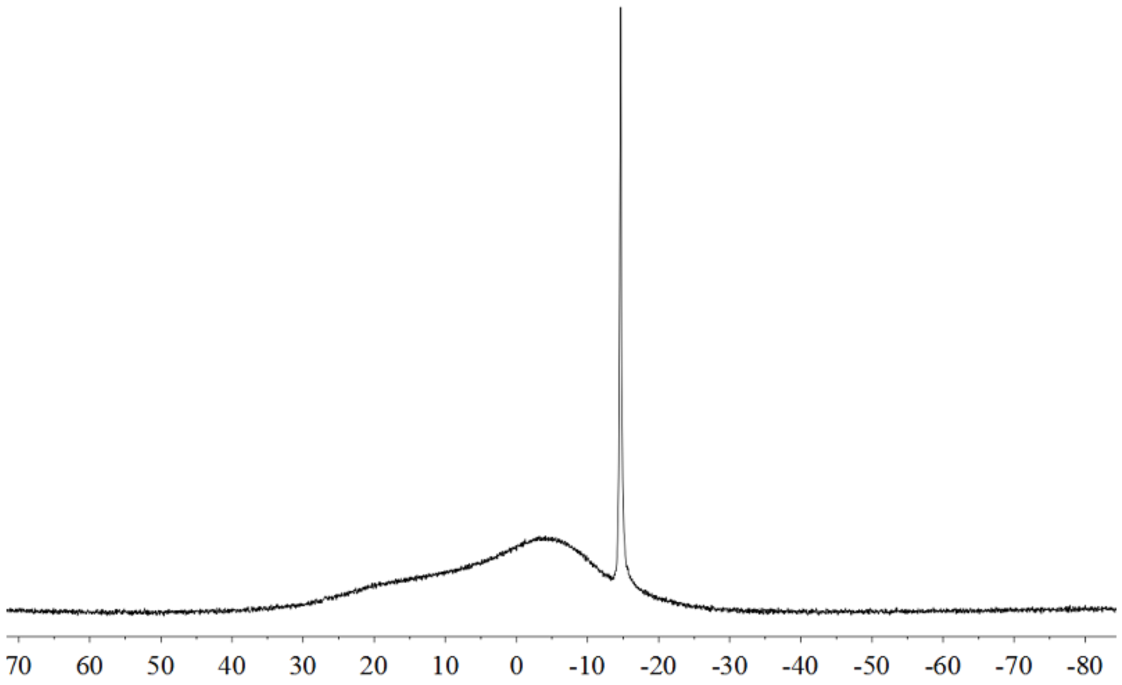

Figure S13. $^{11}B\{^{1}H\}$ NMR (160 MHz, 299 K, dichloromethane-d$_2$) spectrum of compound **14a**

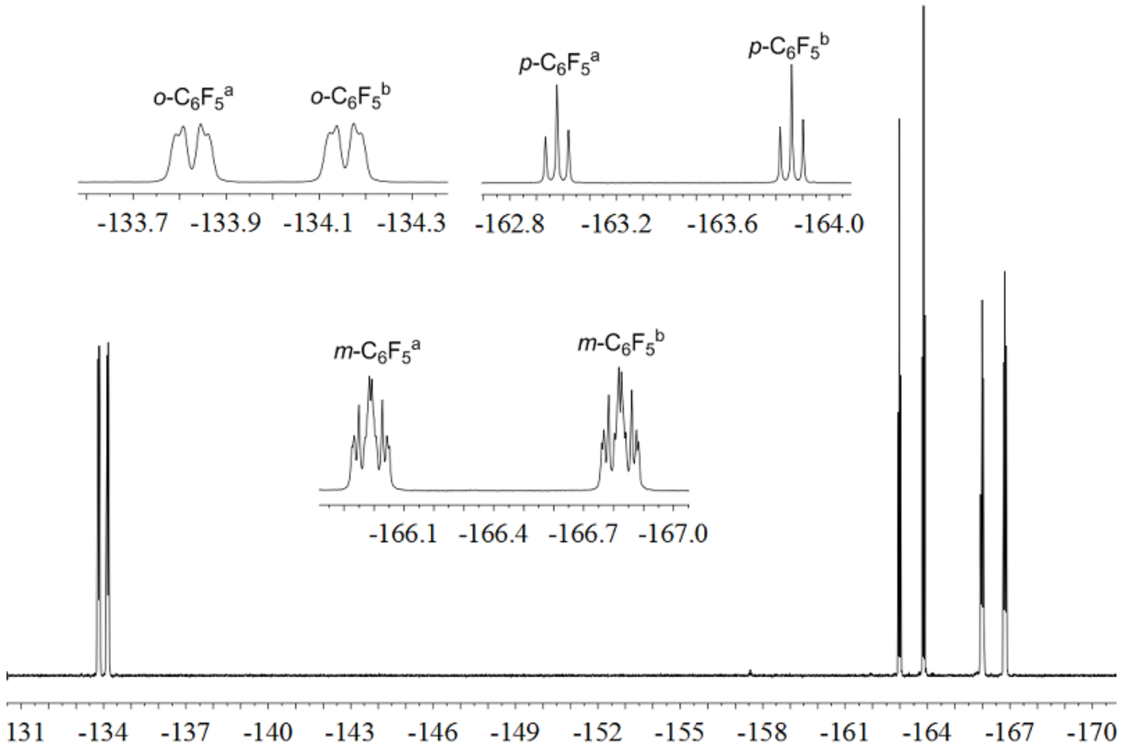

Figure S14. ^{19}F NMR (470 MHz, 299 K, dichloromethane-d$_2$) spectrum of compound **14a**

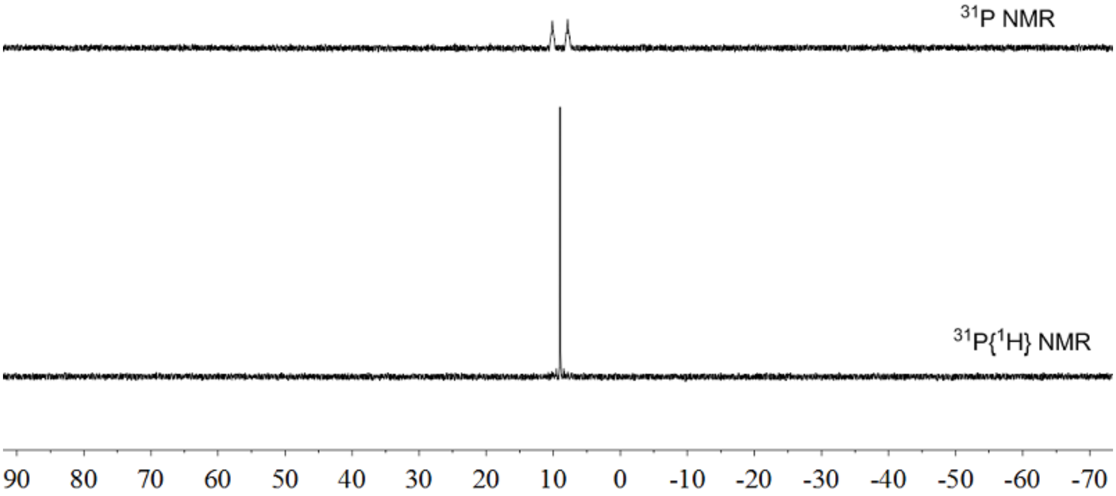

Figure S15. ^{31}**P{**^{1}**H} NMR** and ^{31}**P NMR** (202 MHz, 299 K, dichloromethane-d_2) spectrum of compound **14a**

Crystals suitable for the X-ray crystal structure analysis were obtained by slow diffusion of pentane to a solution of compound **14a** in dichloromethane at -36 ^{o}C.

X-ray crystal structure analysis of compound 14a: A colorless plate-like specimen of $C_{40}H_{34}BF_{10}P$, approximate dimensions 0.044 mm x 0.206 mm x 0.388 mm, was used for the X-ray crystallographic analysis. The X-ray intensity data were measured. A total of 342 frames were collected. The total exposure time was 6.65 hours. The frames were integrated with the Bruker SAINT software package using a narrow-frame algorithm. The integration of the data using a monoclinic unit cell yielded a total of 46282 reflections to a maximum θ angle of 25.03° (0.84 Å resolution), of which 7340 were independent (average redundancy 6.305, completeness = 99.9%, R_{int} = 15.27%, R_{sig} = 8.51%) and 4607 (62.77%) were greater than 2σ(F^2). The final cell constants of a = 13.2212(8) Å, b = 16.1944(12) Å, c = 19.5088(13) Å, β = 94.223(2)°, volume = 4165.7(5) Å^3, are based upon the refinement of the XYZ-centroids of 5505 reflections above 20 σ(I) with 4.605° < 2θ < 50.76°. Data were corrected for absorption effects using the multi-scan method (SADABS). The ratio of minimum to maximum apparent transmission was 0.915. The calculated minimum and maximum transmission coefficients (based on crystal size) are 0.9490 and 0.9940. The final anisotropic full-matrix least-squares refinement on F^2 with 479 variables converged at R1 = 6.07%, for the observed data and wR2 = 14.36% for all data. The goodness-of-fit was 1.020. The largest peak in the final difference electron density synthesis was 0.306 e^-/Å^3 and the largest hole was -0.285 e^-/Å^3 with an RMS deviation of 0.065 e^-/Å^3. On the basis of the final model, the calculated density was 1.190 g/cm^3 and F(000), 1536 e^-. The position of the hydrogen atom at P1 was refined freely; others hydrogen atoms were calculated and refined as riding atoms.

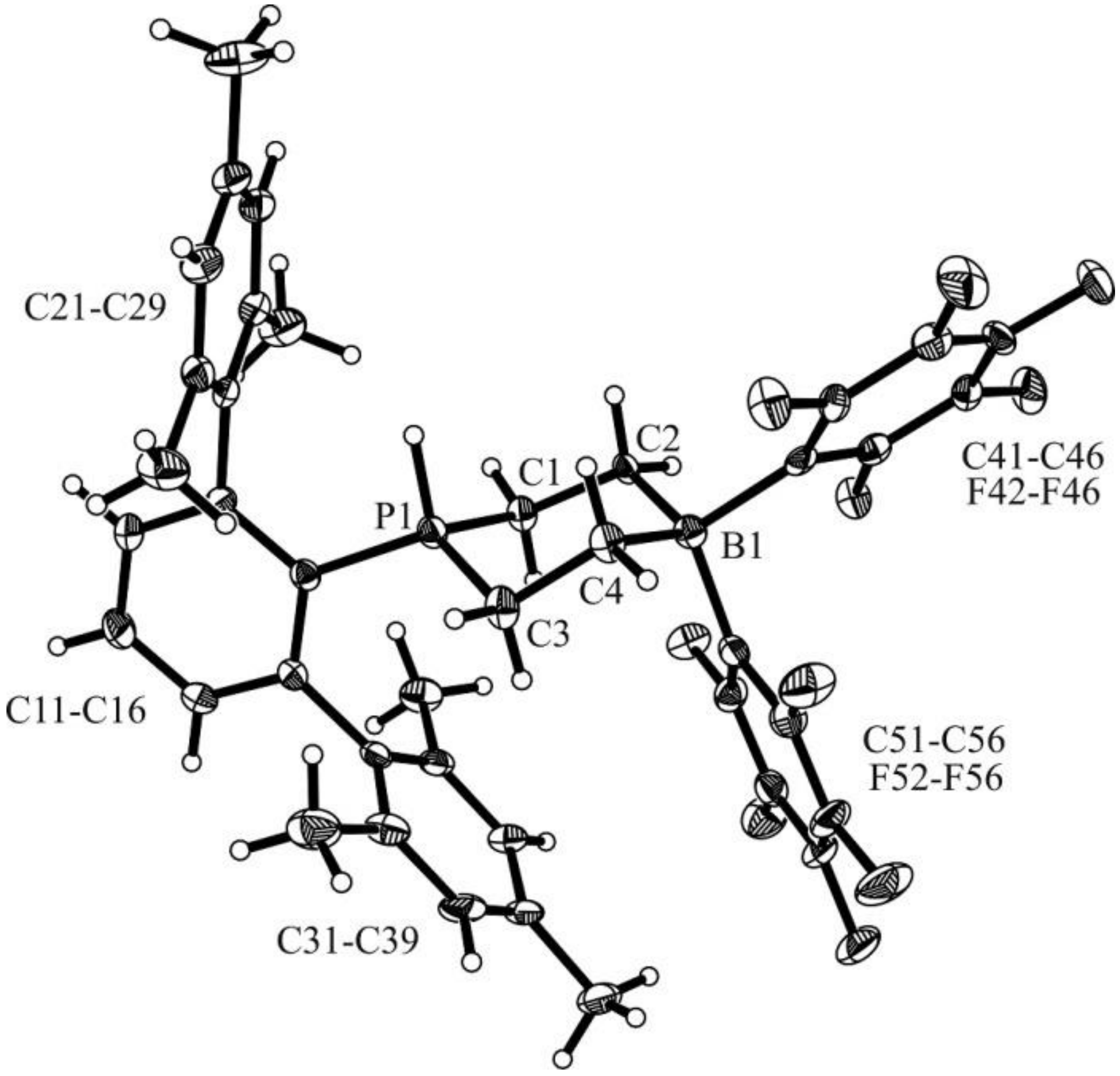

Figure S16. X-ray crystal structure of compound **14a**

Characterization of compound 15py

Elemental analysis (%) of compound 15py calcd. for $C_{22}H_{16}BF_{10}N$: C 53.43, H 3.21, N 2.83; Found: C 53.43, H 3.21, N 2.82.
Melting point of compound 15py: 80 °C
NMR data of compound 15py [NMR data from a solution of the white solid compound **10** in dichloromethane-d_2.]
^{1}H NMR (500 MHz, 299 K, dichloromethane-d_2) δ = 8.69 (m, 2H, *o*-py), 8.12 (t, $^3J_{HH}$ = 7.7 Hz, 1H, *p*-py), 7.66 (m, 2H, *m*-py), 1.33, 1.30, 1.25, 0.94 (each m, each 2H, CH_2), 0.83 (m, 3H, CH_3).
^{13}C{^{1}H} NMR (126 MHz, 299 K, dichloromethane-d_2) δ = 148.5 (dm, $^1J_{FC}$ ~ 240 Hz, C_6F_5), 146.3 (*o*-py), 142.0 (*p*-py), 139.7 (dm, $^1J_{FC}$ ~ 248 Hz, C_6F_5), 137.5 (dm, $^1J_{FC}$ ~ 250 Hz, C_6F_5), 126.4 (*m*-py), 121.2 (br, *i*-C_6F_5), 36.1, 26.5, 24.4 (br), 22.9 (CH_2), 14.3 (CH_3). **^{11}B{^{1}H} NMR** (160 MHz, 299 K, dichloromethane-d_2) δ = -0.5 ($\nu_{1/2}$ ~ 150 Hz).
^{19}F NMR (470 MHz, 299 K, dichloromethane-d_2) δ = -132.4 (m, 2F, *o*-C_6F_5), -159.5 (t, $^3J_{FF}$ = 20.2 Hz, *p*-C_6F_5), -164.8 (m, 2F, *m*-C_6F_5) [$\Delta\delta^{19}F_{m,p}$ = 5.3]

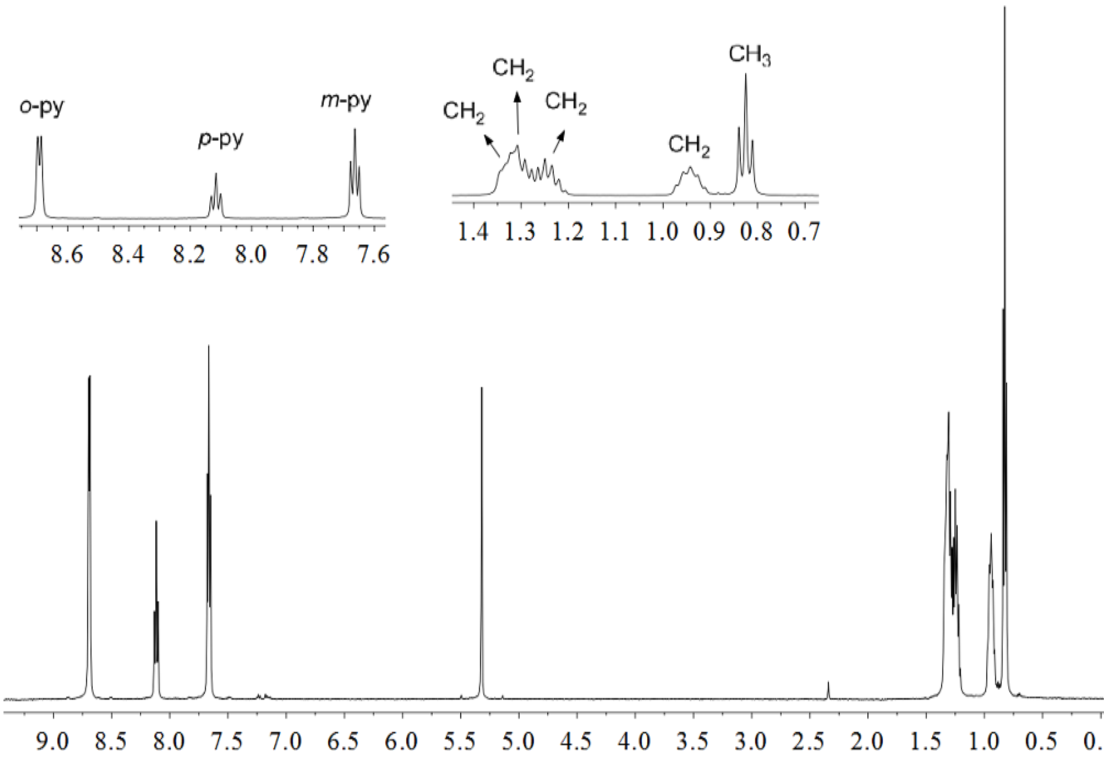

Figure S17. ^{1}**H NMR** (500 MHz, 299 K, dichloromethane-d$_2$) spectrum of compound **15py**

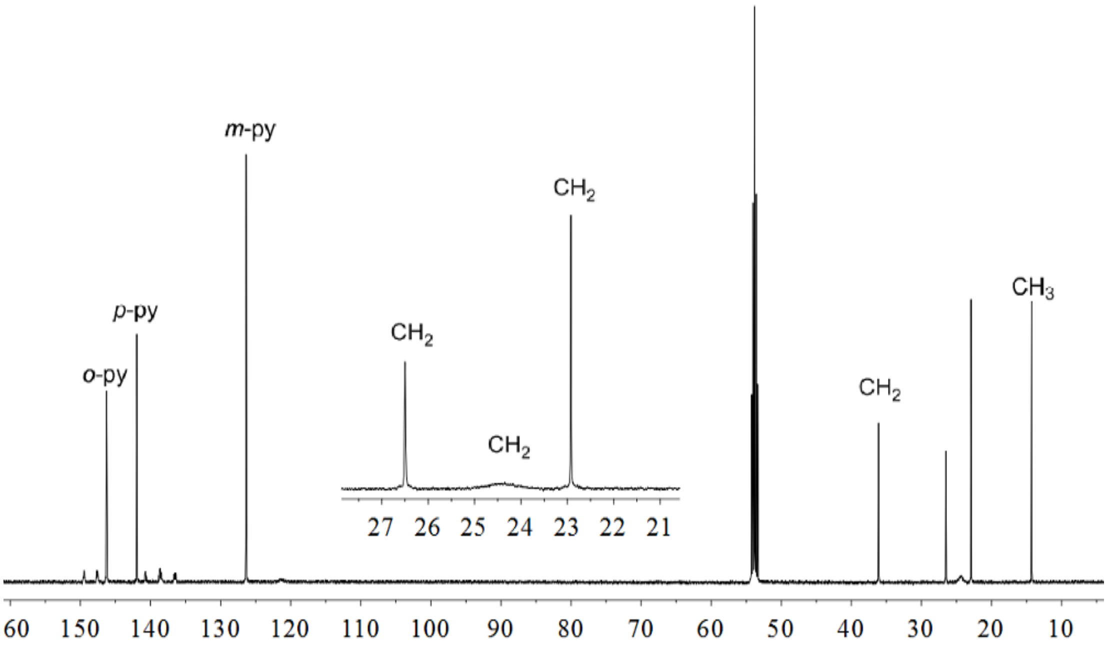

Figure S18. ^{13}**C{**^{1}**H} NMR** (126 MHz, 299 K, dichloromethane-d$_2$) spectrum of compound **15py**

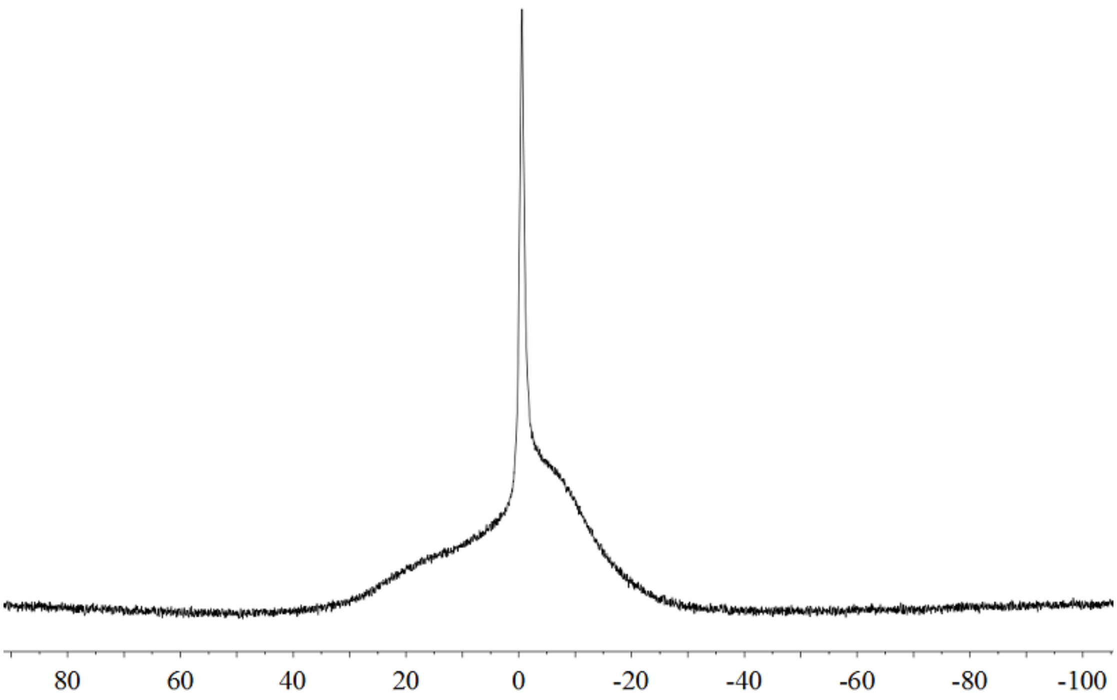

Figure S19. **$^{11}B\{^1H\}$ NMR** (160 MHz, 299 K, dichloromethane-d$_2$) spectrum of compound **15py**

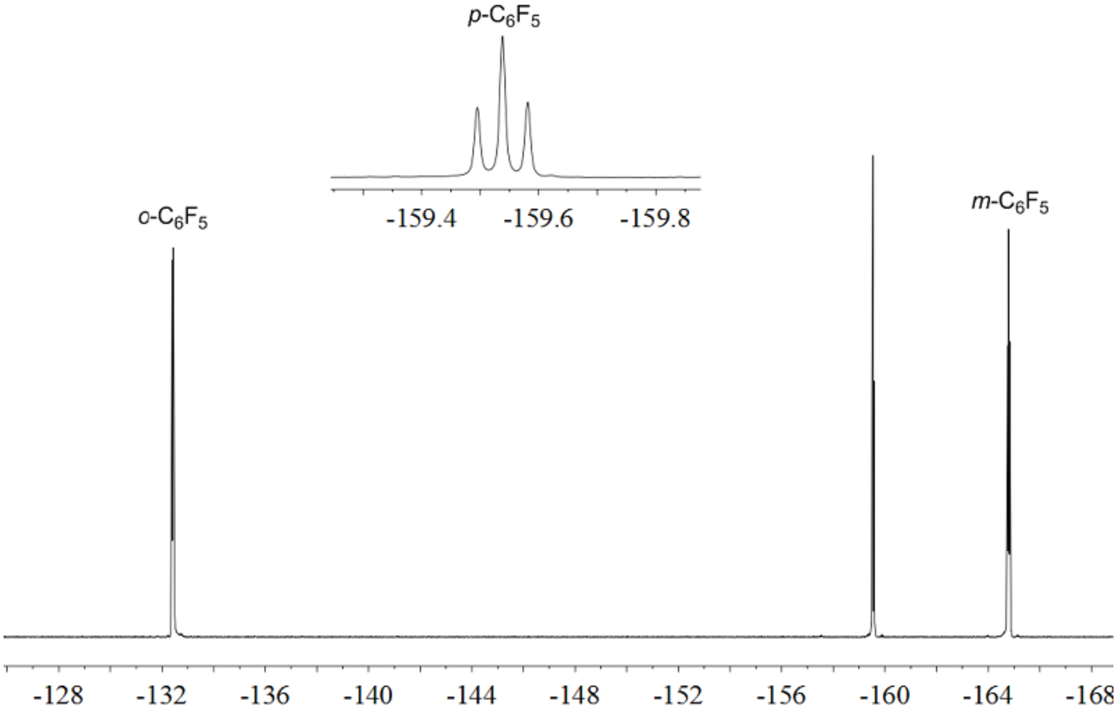

Figure S20. **^{19}F NMR** (470 MHz, 299 K, dichloromethane-d$_2$) spectrum of compound **15py**

Synthesis of compound 14b

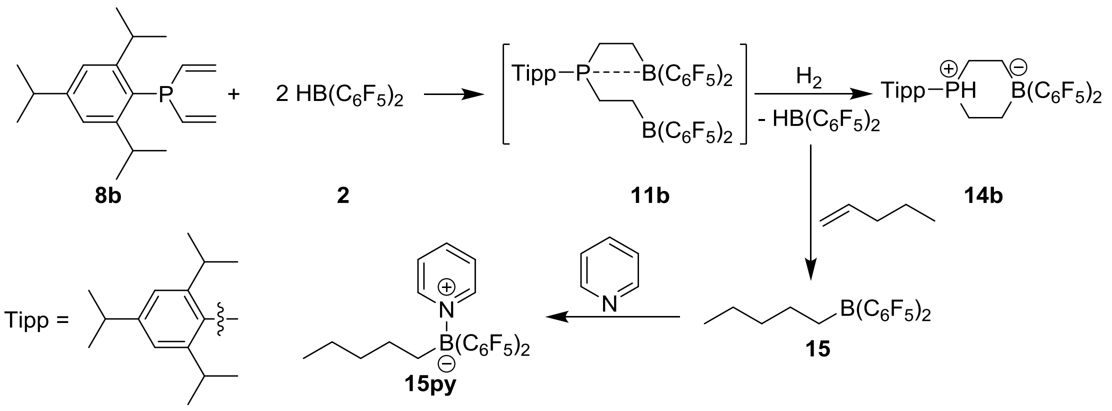

Scheme S6.

In a Schlenk flask, a solution of compound **8b** (86.5 mg, 0.30 mmol, 1.0 eq.) and compound **2** (217.9 mg, 0.63 mmol, 2.1 eq.) in pentane (10 mL) and stirred at room temperature for 24 h. Then the mixture was filtered via cannula. The obtained solution was cooled to -78 °C (dry ice / isopropanol bath) and the flask was evacuated carefully. Then the dry ice bath was removed and the reaction mixture was exposed to H_2 gas (1.5 bar). Subsequently the formed suspension was stirred at room temperature for 2 h. Then all volatiles were removed in vacuo and dichloromethane (6 mL) was added to give a suspension. After addition of pentene (42.1 mg, 0.6 mmol, 2 eq.), the reaction mixture was stirred at room temperature for 1 day until the solid was dissolved. Then the volatiles were removed in vacuo to give a white solid, which was washed with pentane (2 mL × 3) and dried in vacuo to give compound **14b** as a white solid (133.6 mg, 0.210 mmol, 70 % yield).

The combined washing solutions were collected and pyridine (23.7 mg, 0.3 mmol, 1.0 eq.) was added. The mixture was stored at -36 °C to give compound **15py** as a white solid (81.7 mg, 0.165 mmol, 55 % yield). The obtained NMR data of compound **15py** were consistant with those listed above (page 49).

HRMS of compound 14b: m/z calc. for $C_{31}H_{32}PBF_{10}$+[Na^+] 659.2067; found 659.2071.

Melting point of compound 14b: 189 °C

NMR data of compound 14b [NMR data from a solution of the white solid in dichloromethane-d$_2$.]

^{1}H NMR (500 MHz, 299 K, dichloromethane-d$_2$) δ = 7.18 (d, $^4J_{PH}$ = 4.2 Hz, 2H, *m*-Tipp), 6.67 (dm, $^1J_{PH}$ = 449.1 Hz 1H, PH), 3.00 (sept, $^3J_{HH}$ = 6.6 Hz, 2H, *o*-*i*Pr^{Tipp}), 2.92 (sept, $^3J_{HH}$ = 6.8 Hz, 1H, *p*-*i*Pr^{Tipp}), 2.50/2.49 (each m, each 2H, PCH$_2$), 2.14 (dm, $^3J_{PH}$ = 44.2 Hz)/1.11 (m, 2H, BCH$_2$), 1.242 (d, $^3J_{HH}$ = 6.6 Hz, 12H, *o*-*i*Pr^{Tipp}), 1.237 (d, $^3J_{HH}$ = 6.8 Hz, 6H, *o*-*i*Pr^{Tipp}).

^{13}C{^{1}H} NMR (126 MHz, 299 K, dichloromethane-d$_2$) δ = 156.7 (d, $^4J_{PC}$ = 2.6 Hz, *p*-Tipp), 154.0 (d, $^2J_{PC}$ = 9.4 Hz, *o*-Tipp), 124.0 (d, $^3J_{PC}$ = 10.3 Hz, *m*-Tipp), 111.7 (d, $^1J_{PC}$ = 76.3 Hz, *i*-Tipp), 35.0 (*p*-*i*Pr^{Tipp}), 33.4 (d, $^3J_{PC}$ = 7.9 Hz, *o*-*i*Pr^{Tipp}), 24.4 (*o*-*i*Pr^{Tipp}), 23.6 (*p*-*i*Pr^{Tipp}), 20.7 (d, $^1J_{PC}$ = 45.4 Hz, PCH$_2$), 20.2 (br, BCH$_2$), [C_6F_5 not listed].

^{11}B{^{1}H} NMR (160 MHz, 299 K, dichloromethane-d$_2$) δ = -14.5 ($\nu_{1/2}$ ~ 40 Hz).

^{19}F NMR (470 MHz, 299 K, dichloromethane-d$_2$) δ = -133.1 (m, 2F, *o*), -162.3 (t, $^3J_{FF}$ = 20.2 Hz, 1F, *p*), -165.6 (m, 2F, *m*)(C_6F_5)[$\Delta\delta^{19}F_{m,p}$ = 3.3], -134.1 (m, 2F, *o*), -163.7 (t, $^3J_{FF}$ = 20.2 Hz, 1F, *p*), -166.7 (m, 2F, *m*)(C_6F_5)[$\Delta\delta^{19}F_{m,p}$ = 3.0].

^{31}P{^{1}H} NMR (202 MHz, 299 K, dichloromethane-d$_2$) δ = -6.2 ($\nu_{1/2}$ ~ 15 Hz).

^{31}P NMR (202 MHz, 299 K, dichloromethane-d$_2$) δ = -6.2 (dm, $^1J_{PH}$ ~ 449 Hz).

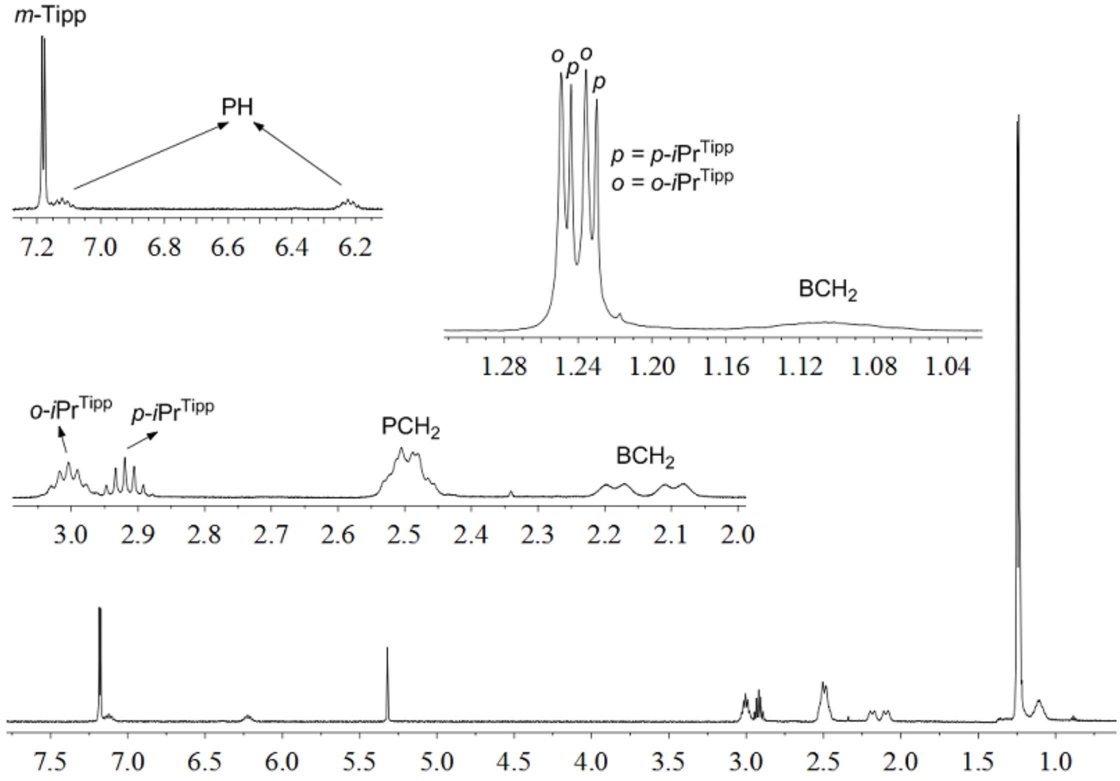

Figure S21. **^{1}H NMR** (500 MHz, 299 K, dichloromethane-d_2) spectrum of compound **14b**

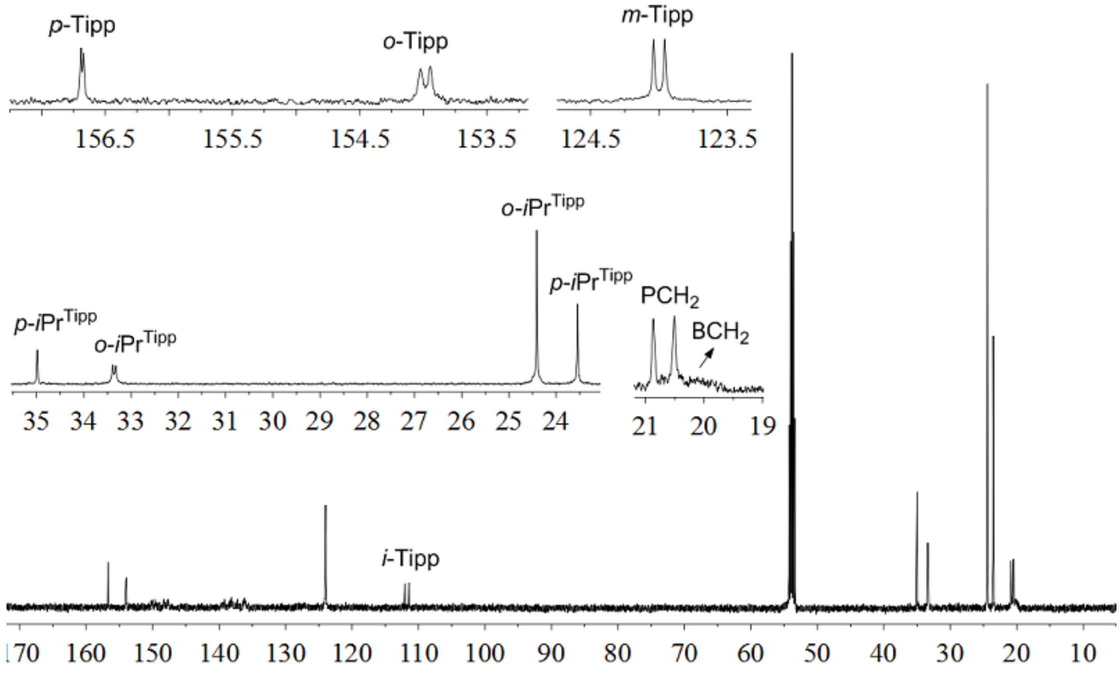

Figure S22. **^{13}C{^{1}H} NMR** (126 MHz, 299 K, dichloromethane-d_2) spectrum of compound **14b**

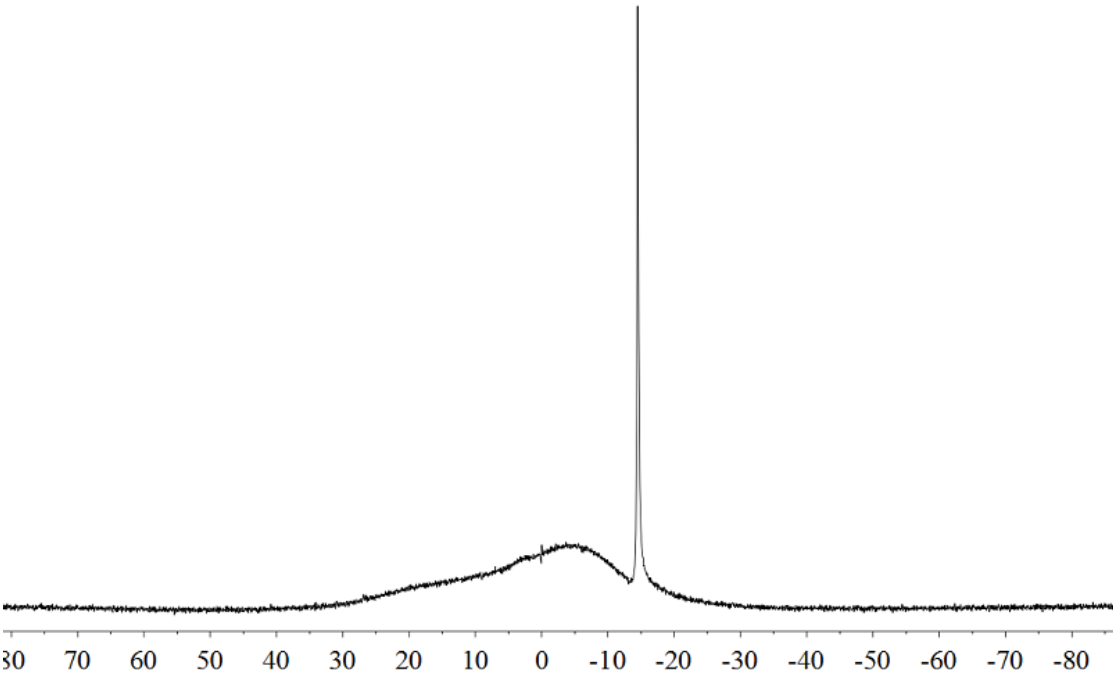

Figure S23. $^{11}B\{^{1}H\}$ NMR (160 MHz, 299 K, dichloromethane-d_2) spectrum of compound **14b**

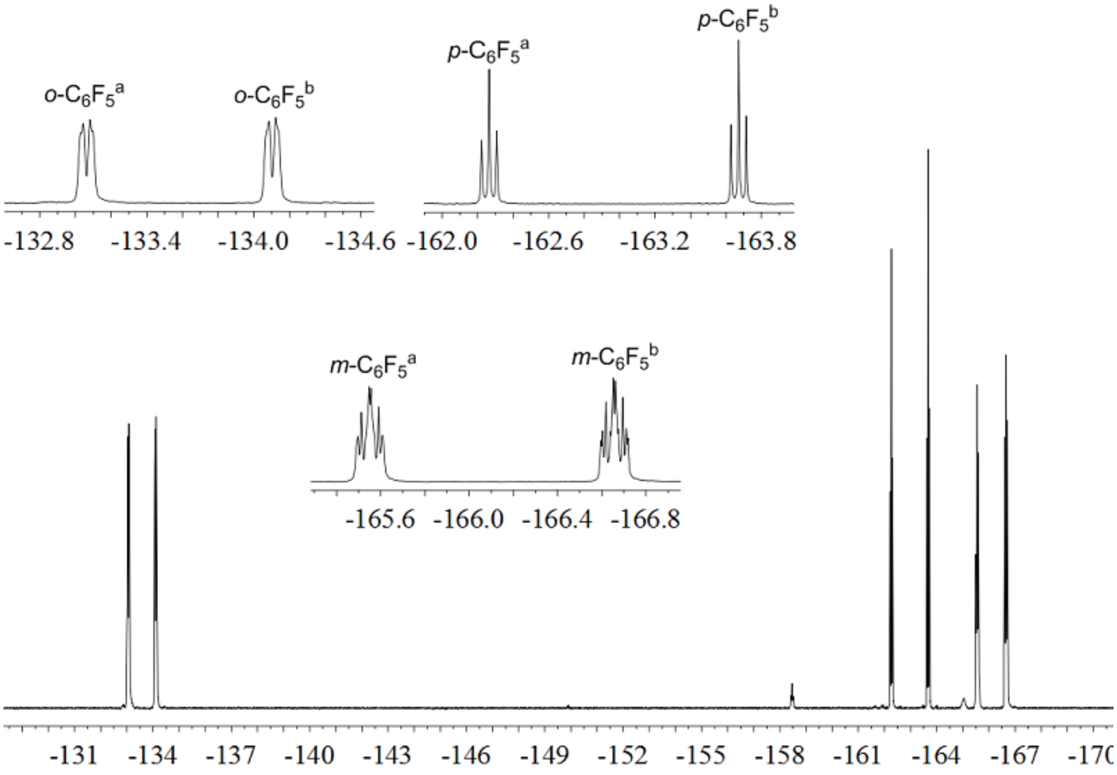

Figure S24. ^{19}F NMR (470 MHz, 299 K, dichloromethane-d_2) spectrum of compound **14b**

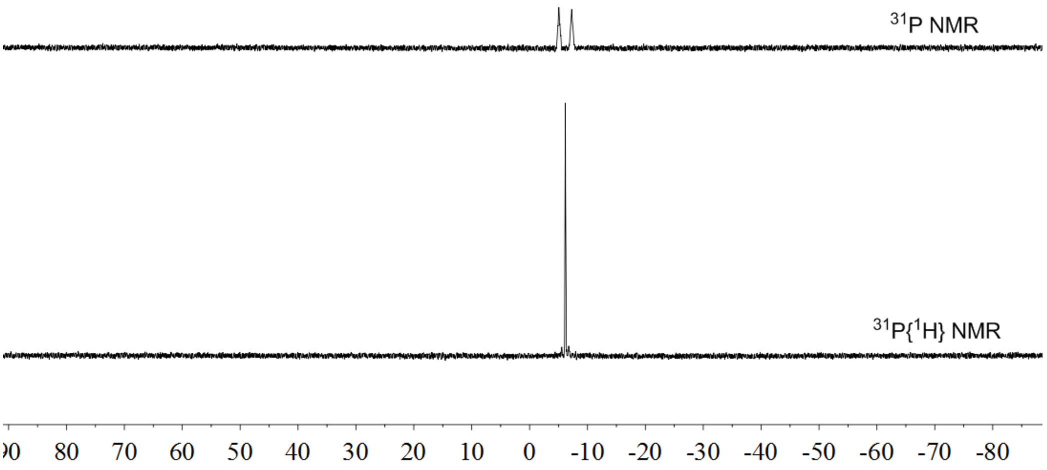

Figure S25. **^{31}P{^{1}H}** (bottom) and **^{31}P NMR** (top) (202 MHz, 299 K, dichloromethane-d_2) spectrum of compound **14b**

Crystals suitable for the X-ray crystal structure analysis were obtained from slow diffusion of pentane to a solution of compound **14b** in dichloromethane at -36 °C.

X-ray crystal structure analysis of compound 14b: A colorless plate-like specimen of $C_{31}H_{32}BF_{10}P$, approximate dimensions 0.030 mm x 0.180 mm x 0.300 mm, was used for the X-ray crystallographic analysis. The X-ray intensity data were measured. A total of 1809 frames were collected. The total exposure time was 26.63 hours. The frames were integrated with the Bruker SAINT software package using a wide-frame algorithm. The integration of the data using a monoclinic unit cell yielded a total of 43312 reflections to a maximum θ angle of 67.37° (0.84 Å resolution), of which 5066 were independent (average redundancy 8.550, completeness = 96.5%, R_{int} = 9.19%, R_{sig} = 4.59%) and 3915 (77.28%) were greater than 2σ(F^2). The final cell constants of a = 17.6792(14) Å, b = 8.5320(7) Å, c = 19.5972(15) Å, β = 99.052(4)°, volume = 2919.2(4) Å^3, are based upon the refinement of the XYZ-centroids of 9978 reflections above 20 σ(I) with 6.261° < 2θ < 133.0°. Data were corrected for absorption effects using the multi-scan method (SADABS). The ratio of minimum to maximum apparent transmission was 0.770. The calculated minimum and maximum transmission coefficients (based on crystal size) are 0.6450 and 0.9530. The final anisotropic full-matrix least-squares refinement on F^2 with 398 variables converged at R1 = 4.50%, for the observed data and wR2 = 10.02% for all data. The goodness-of-fit was 1.035. The largest peak in the final difference electron density synthesis was 0.287 e$^-$/Å^3 and the largest hole was -0.336 e$^-$/Å^3 with an RMS deviation of 0.054 e$^-$/Å^3. On the basis of the final model, the calculated density was 1.448 g/cm^3 and F(000), 1312 e$^-$. The position of the hydrogen atom at P1 was refined freely; others hydrogen atoms were calculated and refined as riding atoms.

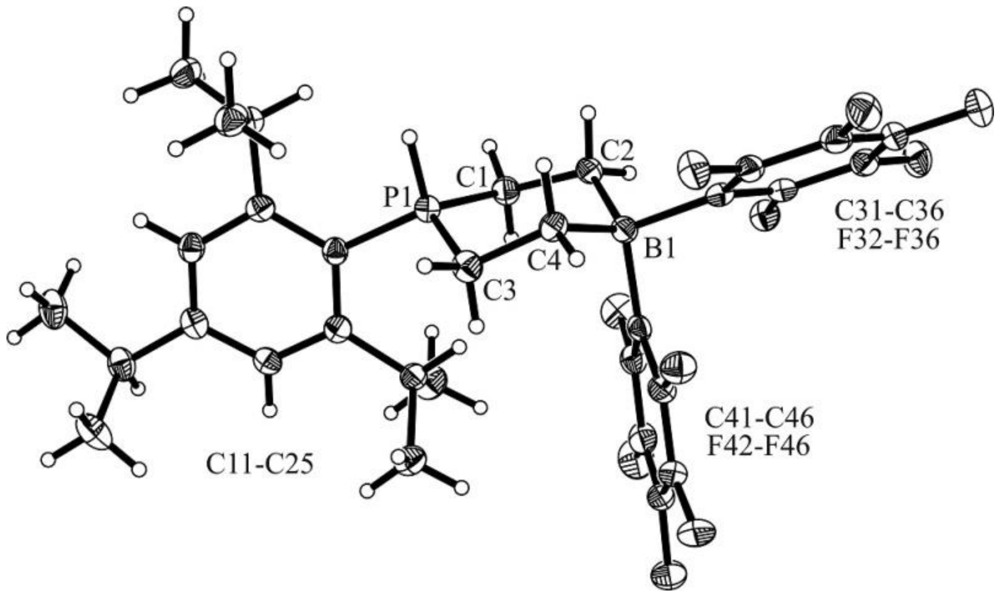

Figure S26. X-ray crystal structure of compound **14b**

In situ reaction of compound 14b (*in situ* generated) with H_2

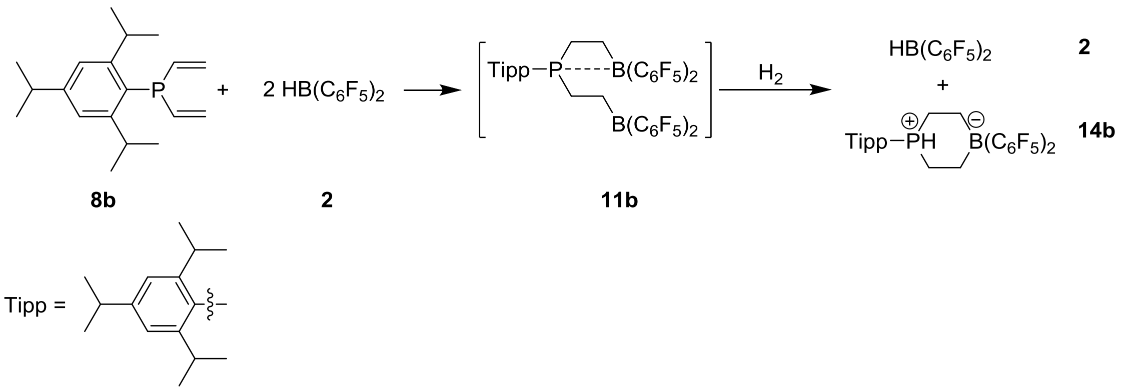

Scheme S7.

In a Schlenk flask, a solution of compound **8b** (11.5 mg, 0.04 mmol, 1.0 eq.) and compound **2** (29.0 mg, 0.084 mmol, 2.1 eq.) in pentane (2 mL) was stirred at room temperature for 12 h. Then the mixture was filtered. After the solution was filled in a J-Young tube which was cooled to -78 °C (dry ice / isopropanol bath), it was evacuated carefully. Then the dry ice bath was removed and the reaction mixture was exposed to H_2 gas (1.5 bar). The formed suspension was kept at room temperature for 16 h. Then the supernatant was removed by decantation and the collected solid was dissolved in dichloromethane-d_2 (1 mL).

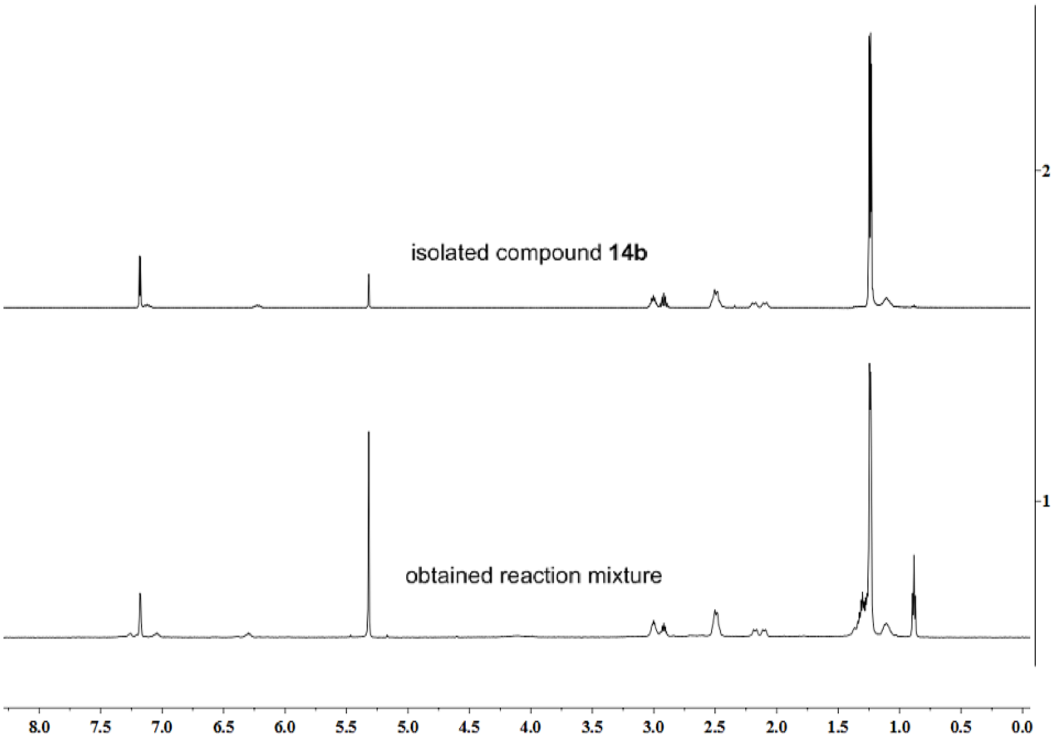

Figure S27. (1) **^{1}H NMR** (600 MHz, 299 K, dichloromethane-d_2) spectrum of the obtained reaction mixture and (2) **^{1}H NMR** (500 MHz, 299 K, dichloromethane-d_2) spectrum of the isolated compound **14b**

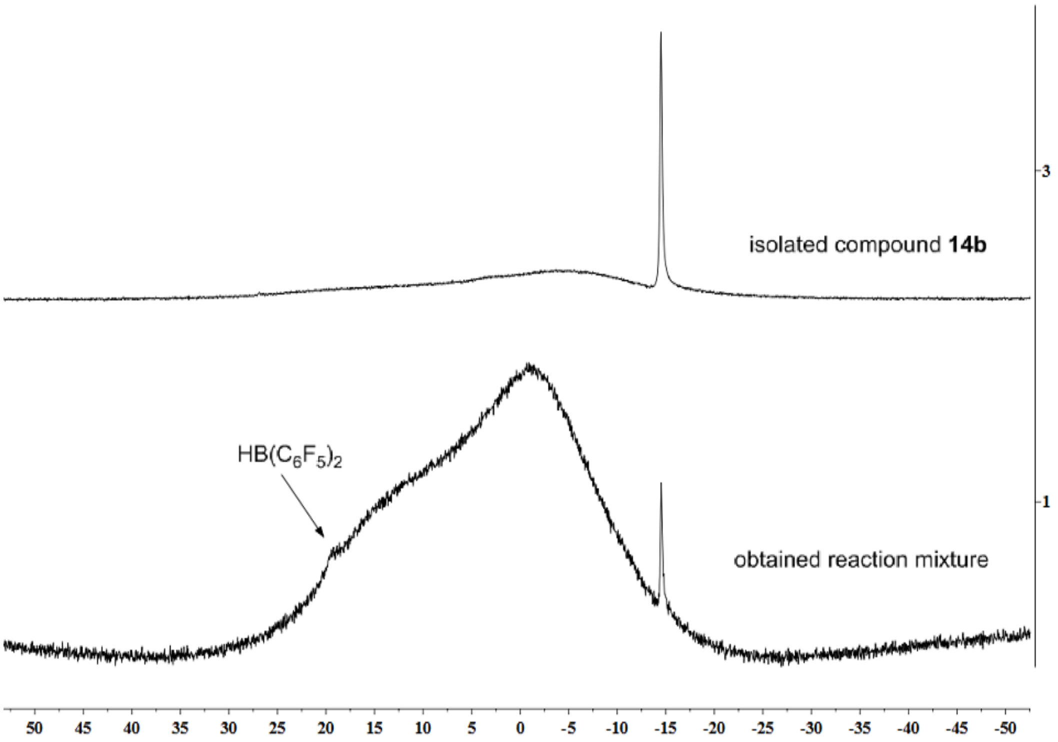

Figure S28. (1) ^{11}**B{^{1}H} NMR** (192 MHz, 299 K, dichloromethane-d$_2$) spectrum of the obtained reaction mixture and (2) ^{11}**B{^{1}H}** (160 MHz, 299 K, dichloromethane-d$_2$) spectrum of the isolated compound **14b**

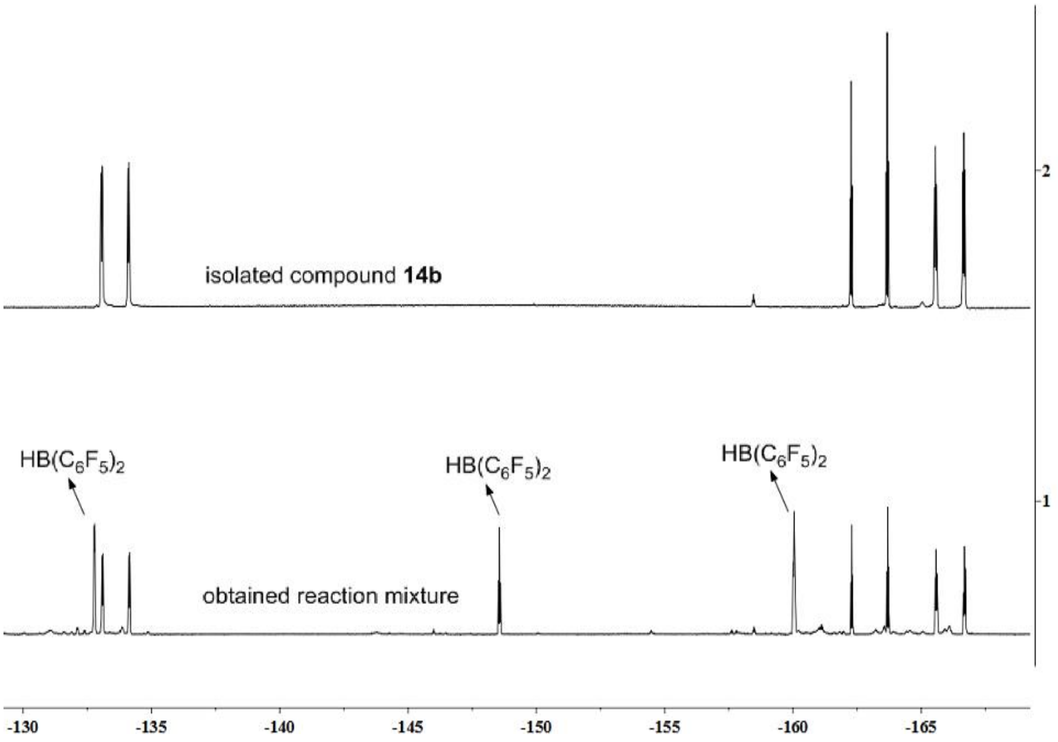

Figure S29. (1) ^{19}**F NMR** (564 MHz, 299 K, dichloromethane-d$_2$) spectrum of the obtained reaction mixture and (2) ^{19}**F NMR** (473 MHz, 299 K, dichloromethane-d$_2$) spectrum of the isolated compound **14b**

Synthesis of compound 14c

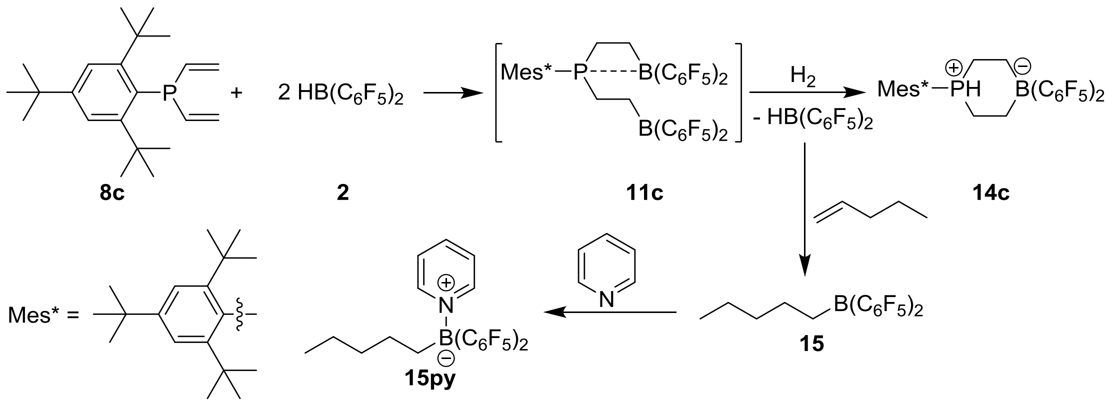

Scheme S8.

In a Schlenk flask, a solution of compound **8c** (99.1 mg, 0.30 mmol, 1.0 eq.) and compound **2** (207.6 mg, 0.6 mmol, 2.0 eq.) in dichloromethane (6 mL) was stirred at room temperature for 15 min to give a yellow solution. After the solution was cooled to -78 °C (dry ice / isopropanol bath) and the flask was evacuated carefully, the dry ice bath was removed and the reaction mixture was exposed to H_2 gas (1.5 bar). Subsequently the formed suspension was stirred at room temperature for 30 min, then the H_2 was released under argon atmosphere followed by the addition of pentene (42.1 mg, 0.6 mmol, 2 eq.). The reaction mixture was stirred at room temperature for 2 days until the precipitate had dissolved again. Then all volatiles were removed in vacuo to give a white solid which was washed with pentane (2 mL × 3) and dried in vacuo to give compound **14c** as a white solid (140.4 mg, 0.207 mmol, 69 % yield).

The combined washing solutions were collected and pyridine (23.7 mg, 0.3 mmol, 1.0 eq.) was added. The mixture was stored at -36 °C to give compound **15py** as a white solid (89.1 mg, 0.180 mmol, 60 % yield). The obtained NMR data of compound **15py** were consistant with those listed above (page 49).

HRMS of compound 14c: m/z calc. for $C_{34}H_{38}PBF_{10}$+[Na^+] 701.2537; found 701.2543.

Melting point of compound 14c: 208 °C

NMR data of compound 14c [NMR data from a solution of the white solid compound **14c** in dichloromethane-d_2.]

^{1}H NMR (500 MHz, 299 K, dichloromethane-d_2) δ = 7.56 (d, $^4J_{PH}$ = 4.3 Hz, 2H, *m*-Mes*), 6.61 (dt, $^1J_{PH}$ = 458.3 Hz, $^3J_{HH}$ = 13.2 Hz, 1H, PH), 2.77/2.44 (each m, each 2H, PCH_2), 2.16 (dm, $^3J_{PH}$ = 46.2 Hz)/1.11 (m)(each 2H, BCH_2), 1.49 (s, 18H, *o*-*t*Bu^{Mes*}), 1.31 (s, 9H, *p*-*t*Bu^{Mes*}),.

^{13}C{^{1}H} NMR (126 MHz, 299 K, dichloromethane-d_2) δ = 158.7 (d, $^2J_{PC}$ = 7.3 Hz, *o*-Mes*), 156.3 (d, $^4J_{PC}$ = 3.5 Hz, *p*-Mes*), 125.9 (d, $^3J_{PC}$ = 11.7 Hz, *m*-Mes*), 113.0 (d, $^1J_{PC}$ = 67.1 Hz, *i*-Mes*), 39.2 (d, $^3J_{PC}$ = 3.5 Hz, *o*-*t*Bu^{Mes*}), 35.5 (d, $^5J_{PC}$ = 1.3 Hz, *p*-*t*Bu^{Mes*}), 33.9 (*o*-*t*Bu^{Mes*}), 30.9 (*p*-*t*Bu^{Mes*}), 24.7 (d, $^1J_{PC}$ = 43.0 Hz, PCH_2), 22.7 (br, BCH_2), [C_6F_5 not listed]

^{11}B{^{1}H} NMR (160 MHz, 299 K, dichloromethane-d_2) δ = -14.3 ($\nu_{1/2}$ ~ 40 Hz).

^{31}P{^{1}H} NMR (202 MHz, 299 K, dichloromethane-d_2) δ = 14.9 ($\nu_{1/2}$ ~ 10 Hz).

^{31}P NMR (202 MHz, 299K, dichloromethane-d_2) δ = 14.9 (d, $^1J_{PH}$ ~ 458 Hz).

^{19}F NMR (470 MHz, 299 K, dichloromethane-d_2) δ = -133.4 (m, 2F, *o*), -162.6 (t, $^3J_{FF}$ = 20.3 Hz, 1F, *p*), -166.1 (m, 2F, *m*)(C_6F_5)[$\Delta\delta^{19}F_{m,p}$= 3.5], -133.8 (m, 2F, *o*), -163.7 (t, $^3J_{FF}$ = 20.1 Hz, 1F, *p*), -166.7 (m, 2F, *m*)(C_6F_5)[$\Delta\delta^{19}F_{m,p}$ = 3.0].

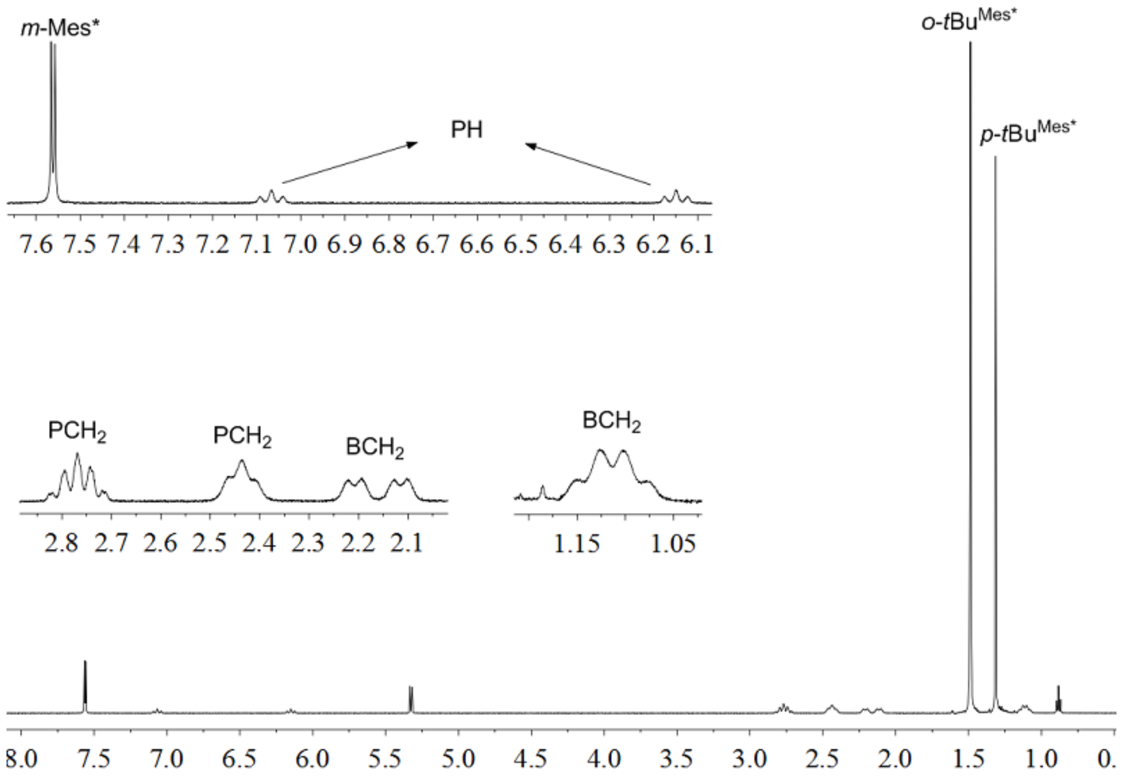

Figure S30. **^{1}H NMR** (500 MHz, 299 K, dichloromethane-d$_2$) spectrum of compound **14c**

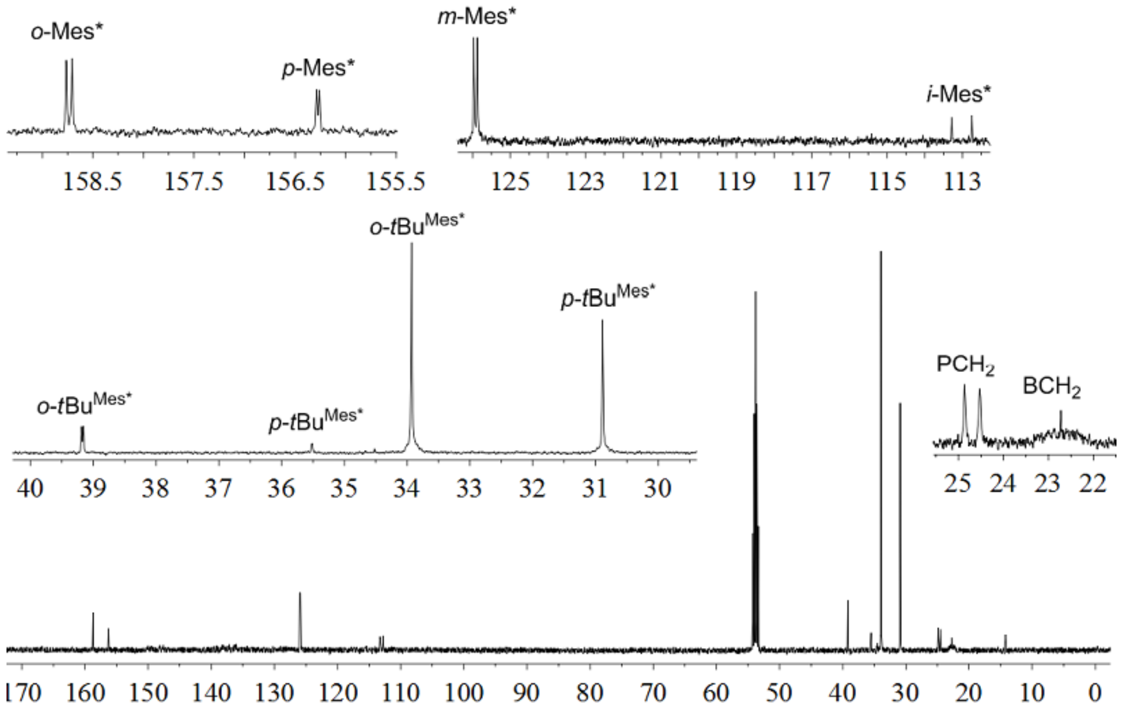

Figure S31. **^{13}C{^{1}H} NMR** (126 MHz, 299 K, dichloromethane-d$_2$) spectrum of compound **14c**

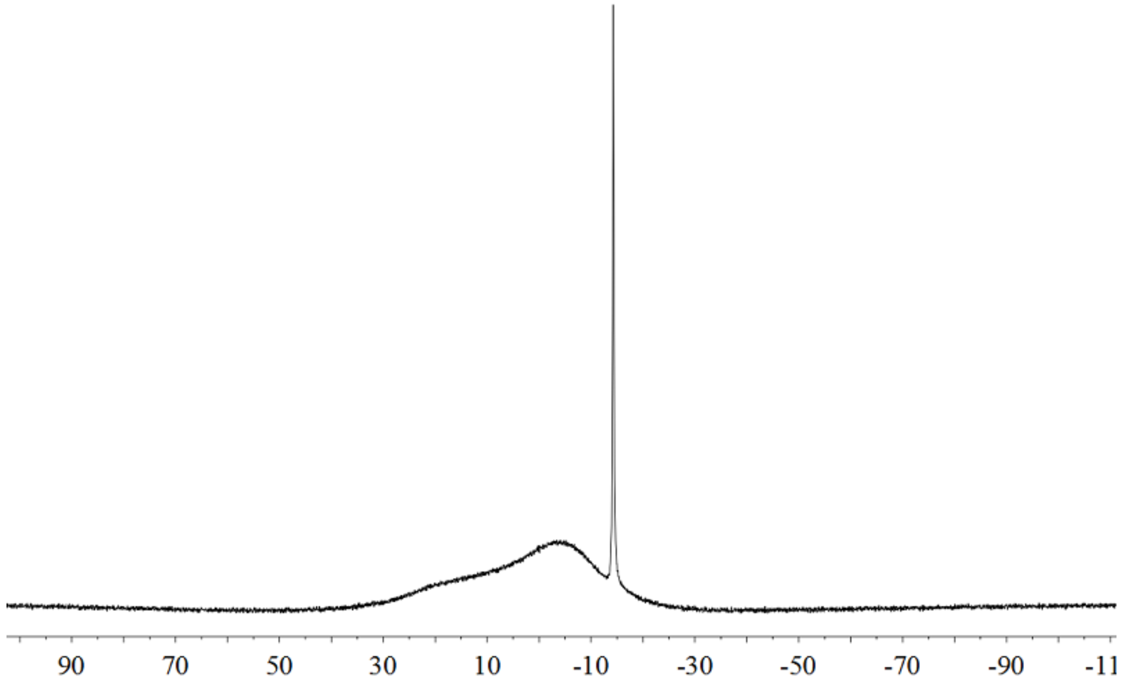

Figure S32. ^{11}**B{**^{1}**H} NMR** (160 MHz, 299 K, dichloromethane-d$_2$) spectrum of compound **14c**

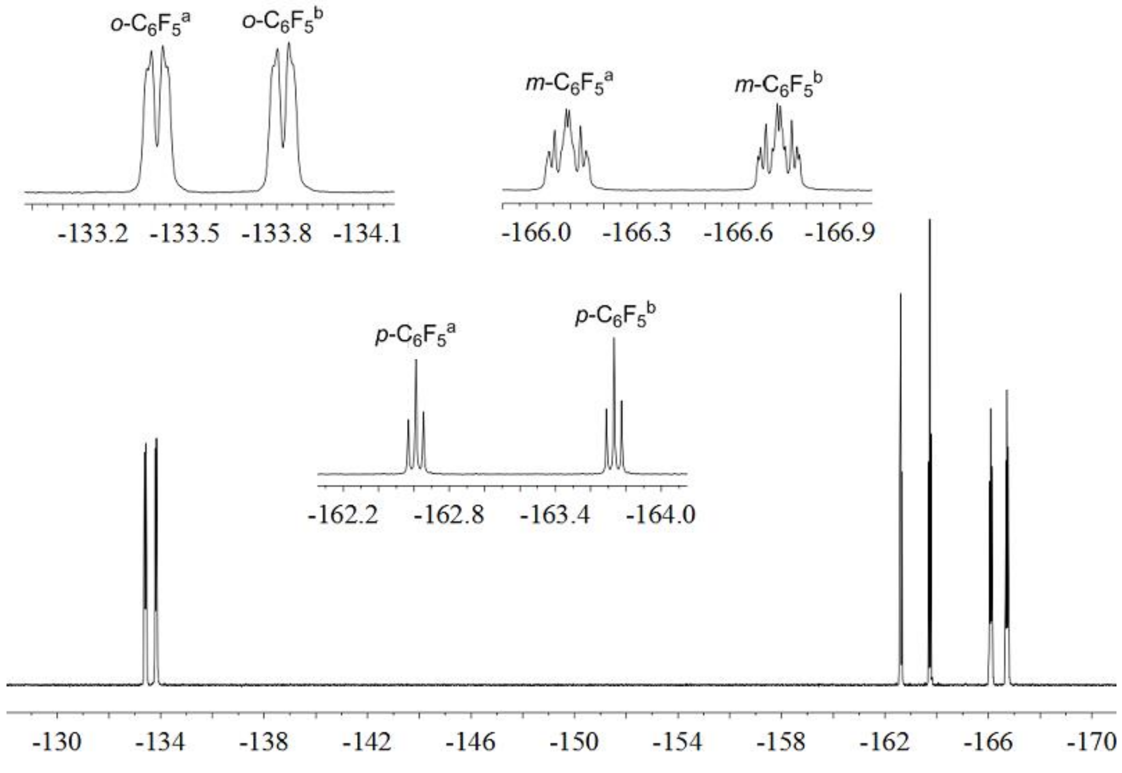

Figure S33. ^{19}**F NMR** (470 MHz, 299 K, dichloromethane-d$_2$) spectrum of compound **14c**

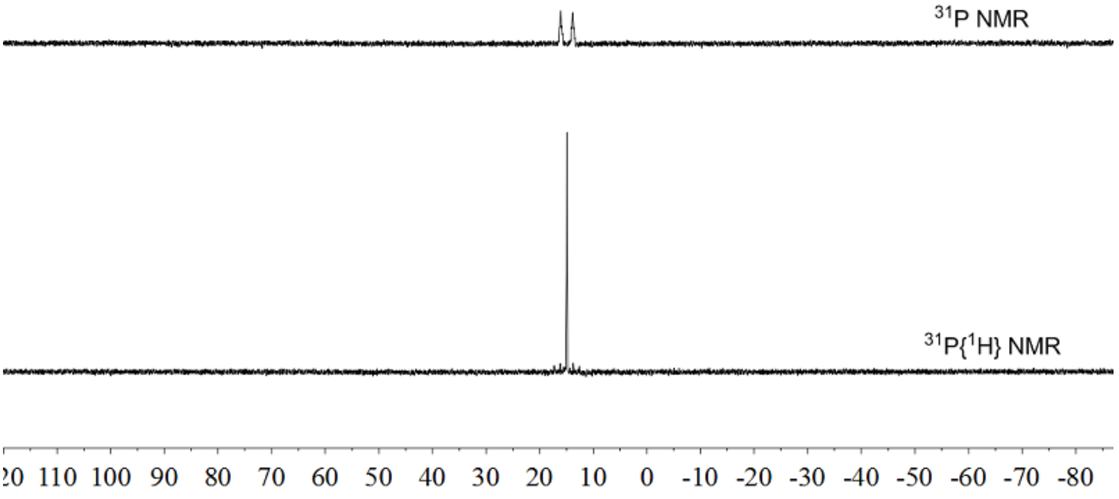

Figure S34. ^{31}P{^{1}H} NMR and **^{31}P NMR** (202 MHz, 299 K, dichloromethane-d_2) spectrum of compound **14c**

Crystals suitable for the X-ray crystal structure analysis of compound **14c** were obtained from slowly diffusion of pentane to a solution of the white solid in dichloromethane at -36 °C.

X-ray crystal structure analysis of compound 14c: formula $C_{34}H_{38}BF_{10}P$, *M* = 678.42, colourless crystal, 0.30 x 0.03 x 0.02 mm, *a* = 13.2091(5), *b* = 14.4202(6), *c* = 17.4143(7) Å, α = 102.491(2), *β* = 96.491(2), γ = 91.561(2)°, *V* = 3213.5(2) Å^3, ρ_{calc} = 1.402 gcm^{-3}, μ = 0.168 mm^{-1}, empirical absorption correction (0.951 ≤ T ≤ 0.996), *Z* = 4, triclinic, space group $P\bar{1}$ (No. 2), λ = 0.71073 Å, *T* = 173(2) K, ω and φ scans, 22688 reflections collected (±*h*, ±*k*, ±*l*), 10899 independent (R_{int} = 0.097) and 6769 observed reflections [I>2σ(I)], 855 refined parameters, *R* = 0.125, wR^2 = 0.245, max. (min.) residual electron density 0.45 (-0.45) e.Å^{-3}, the positions of the hydrogen atoms at P1A and P1B were refined freely, but with P-H distance restraints (DFIX); others hydrogen atoms were calculated and refined as riding atoms.

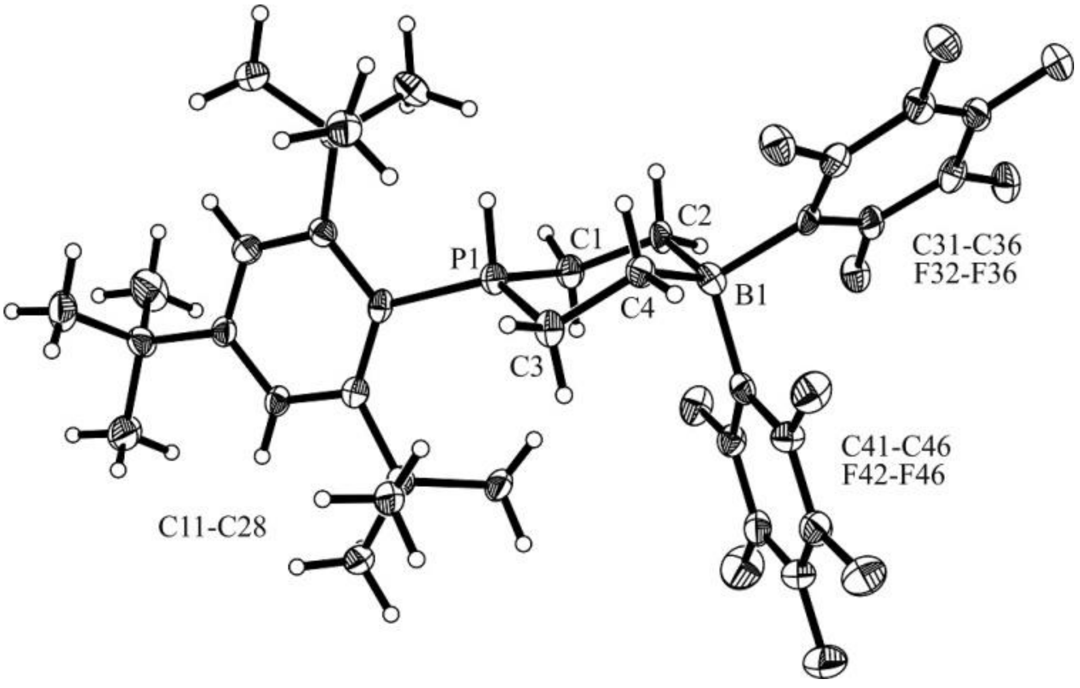

Figure S35. X-ray crystal structure of the compound **14c**

Preparation of compound 17a

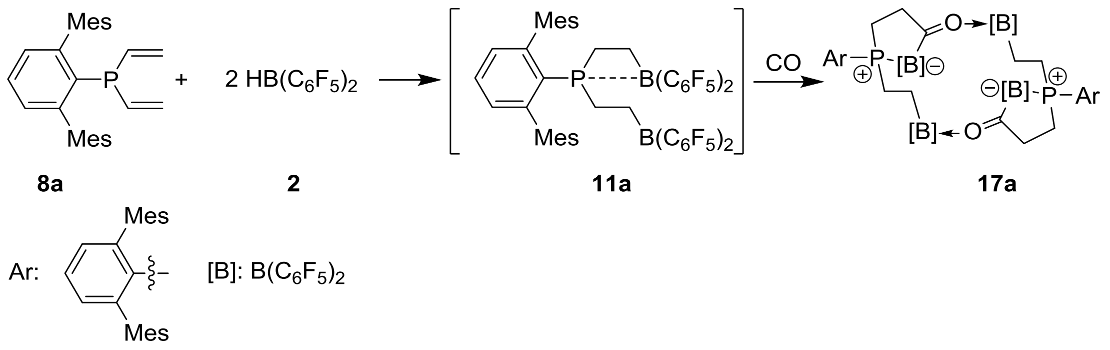

Scheme S9.

In a Schlenk flask, compound **8a** (79.7 mg, 0.20 mmol, 1.0 eq.) and compound **2** (138.4 mg, 0.40 mmol, 2.0 eq.) were mixed with dichloromethane (4 mL) and stirred at room temperature for 15 min to give a yellow solution. The solvent was removed in vacuo and the residue was dissolved in pentane (4 mL). Then the solution was cooled to -78 °C (dry ice / isopropanol bath) and subsequently the flask was evacuated carefully. Then, after the dry ice bath was removed and the reaction mixture was exposed to CO gas (1.5 bar) at room temperature, the obtained reaction mixture was stirred at room temperature for 30 min to give a white suspension. Filtration by using a filter canula gave a white solid. The white solid was washed with pentane (1 mL × 2) and dried in vacuo. Compound **17a** was obtained as a white solid (135.0 mg, 0.061 mmol, 61 %).

Elemental analysis (%) calcd. for $C_{106}H_{66}B_4F_{40}O_2P_2$: C 56.92, H 2.97; Found: C 56.95, H 2.89.
Decomp.: 197 °C
IR(KBr): $\tilde{\nu}$ [cm^{-1}] = 1647 (m, C=C), 1579 (m, C=O), 1521 (s, C=C), 1462 (s, C=C), 1288 (m), 1097 (s), 979 (s, =C-H).
[The C=O band at 1579 disappeared in the IR spectrum of the ^{13}C labeled compound **17a** (see below)]

NMR data from a solution of the white solid in dichloromethane-d$_2$.
^{1}H NMR (500 MHz, 223 K, dichloromethane-d$_2$) δ = 7.59 (t, $^3J_{HH}$ = 7.6 Hz, 1H, *p*-C$_6$H$_3$), 7.02 (s, 1H, *m*-Mes^a), 7.01 (m, 1H, *m^a*-C$_6$H$_3$), 6.91 (m, 1H, *m^b*-C$_6$H$_3$), 6.88 (s, 1H, *m'*-Mes^a), 6.19 (s, 1H, *m*-Mes^b), 6.14 (s, 1H, *m'*-Mes^b), 2.64/2.00, 2.33/1.99 (each m, each 1H, CH$_2^{CO}$), 2.33 (s, 3H, *p*-CH$_3^{Mes,a}$), 2.08 (s, 3H, *p*-CH$_3^{Mes,b}$), 1.97/1.35, 1.02/0.85 (each m, each 1H, CH$_2$), 1.77 (s, 3H, *o'*-CH$_3^{Mes,a}$), 1.68 (s, 3H, *o*-CH$_3^{Mes,a}$), 1.57 (s, 3H, *o'*-CH$_3^{Mes,b}$), 1.38 (s, 3H, *o*-CH$_3^{Mes,b}$).
^{13}C{^{1}H} NMR (126 MHz, 223 K, dichloromethane-d$_2$) δ = n.o. (C=O), 147.9, 145.1 (each m, *o^{a,b}*-C$_6$H$_3$), 138.4 (*p*-Mes^b), 138.0 (*p*-Mes^a), 137.7 (*o*-Mes^b), 137.4 (d, $^3J_{PC}$ = 2.5 Hz, *i*-Mes^a), 136.3 (*o'*-Mes^b), 136.2 (*o*-Mes^a), 136.1 (d, $^3J_{PC}$ = 4.2 Hz, *i*-Mes^b), 134.6 (*o'*-Mes^a), 132.8 (br, *p*-C$_6$H$_3$), 132.1 (br d, $^3J_{PC}$ = 8.2 Hz, *m^a*-C$_6$H$_3$), 131.8 (br d, $^3J_{PC}$ = 11.3 Hz, *m^b*-C$_6$H$_3$), 128.9 (*m'*-Mes^a), 128.8 (*m*-Mes^a), 128.2 (m, *m'*-Mes^b), 126.9 (m, *m*-Mes^b), 124.0 (dd, $^1J_{PC}$ = 52.4 Hz, J_{PC} = 8.3 Hz *i*-C$_6$H$_3$), 38.9 (br), 26.5 (br, m)(CH$_2^{CO}$), 21.6 (*o*-CH$_3^{Mes,b}$), 21.0 (m, *o'*-CH$_3^{Mes,b}$), 21.0 (m, *p*-CH$_3^{Mes,a}$), 20.9 (*o*-CH$_3^{Mes,a}$), 19.9 (*p*-CH$_3^{Mes,b}$), 19.3 (*o'*-CH$_3^{Mes,a}$), 17.6 (br d, J_{PC} = 36.9 Hz), 15.3 (br)(CH$_2$), [C$_6$F$_5$ not listed].
^{11}B{^{1}H} NMR (160 MHz, 299K, dichloromethane-d$_2$) δ = 7.8 (very broad), -10.0 ($\nu_{1/2}$ ~300 Hz).
^{10}B NMR (54 MHz, 299 K, dichloromethane-d$_2$) δ = 7.8 ($\nu_{1/2}$ ~ 1600 Hz), -10.0 ($\nu_{1/2}$ ~600 Hz).
^{19}F NMR (470 MHz, 223 K, dichloromethane-d$_2$) δ = -117.0 (m, 1F, *o*), -125.0 (dm, J_{FF} = 71.7 Hz, 1F, *o'*), -152.9 (t, $^3J_{FF}$ = 21.1 Hz, 1F, *p*), -161.5 (m, 1F, *m*), -162.5 (m, 1F, *m'*)(C$_6$F$_5$) [Δδ^{19}F$_{m,p}$ = 9.6, 8.6]; -123.2 (dm, J_{FF} = 71.7 Hz, 1F, *o*), -137.4 (m, 1F, *o'*), -154.5 (t, $^3J_{FF}$ = 20.8 Hz, 1F, *p*), -163.1 (m, 1F, *m*), -163.2 (m, 1F, *m'*)(C$_6$F$_5$) [Δδ^{19}F$_{m,p}$ = 8.6, 8.7]; -125.7 (m, 1F, *o*)^t, -135.1 (m, 1F, *o'*), -156.7 (t, $^3J_{FF}$ = 21.2 Hz, 1F, *p*), -163.4 (m, 1F, *m*), -164.3 (m, 1F, *m'*)(C$_6$F$_5$) [Δδ^{19}F$_{m,p}$ = 6.7, 7.6]; -131.1 (m, 1F, *o*)^t, -131.6 (m, 1F, *o'*), -157.7 (t, $^3J_{FF}$ = 20.8, 1F, *p*), -164.6 (m, 1F, *m*), -166.0 (m, 1F, *m'*)(C$_6$F$_5$) [Δδ^{19}F$_{m,p}$ = 8.3, 6.9], [^{t} tentative assignment].
^{31}P{^{1}H} NMR (202 MHz, 299 K, dichloromethane-d$_2$) δ = 19.1 ($\nu_{1/2}$ ~ 150 Hz).
^{31}P{^{1}H} NMR (243 MHz, 223 K, dichloromethane-d$_2$) δ = 17.6 ($\nu_{1/2}$ ~ 200 Hz).

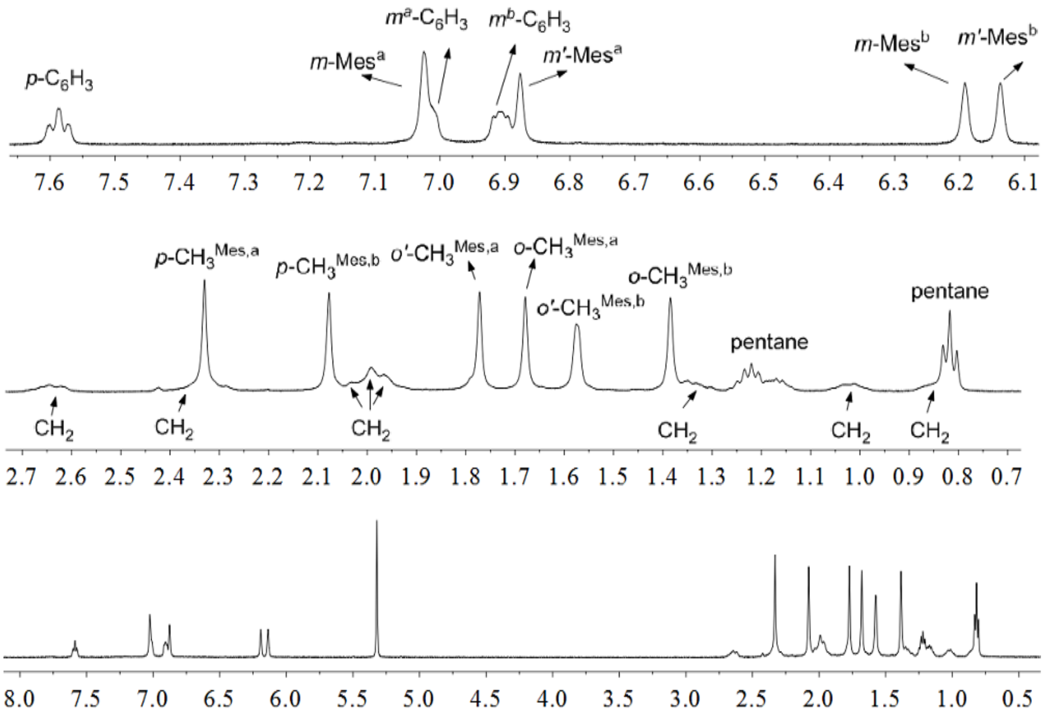

Figure S36. ^{1}**H NMR** (500 MHz, 223 K, dichloromethane-d$_2$) spectrum of compound **17a**

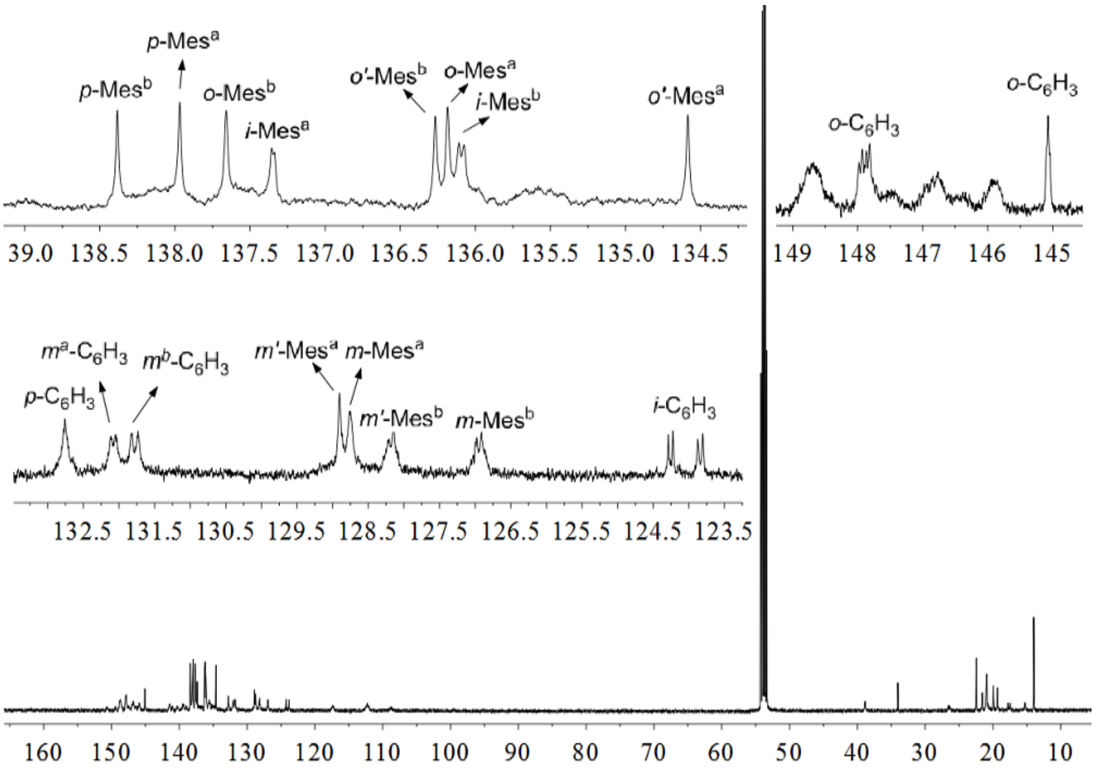

Figure S37. ^{13}**C{**^{1}**H} NMR** (126 MHz, 223 K, dichloromethane-d$_2$) spectrum of compound **17a**

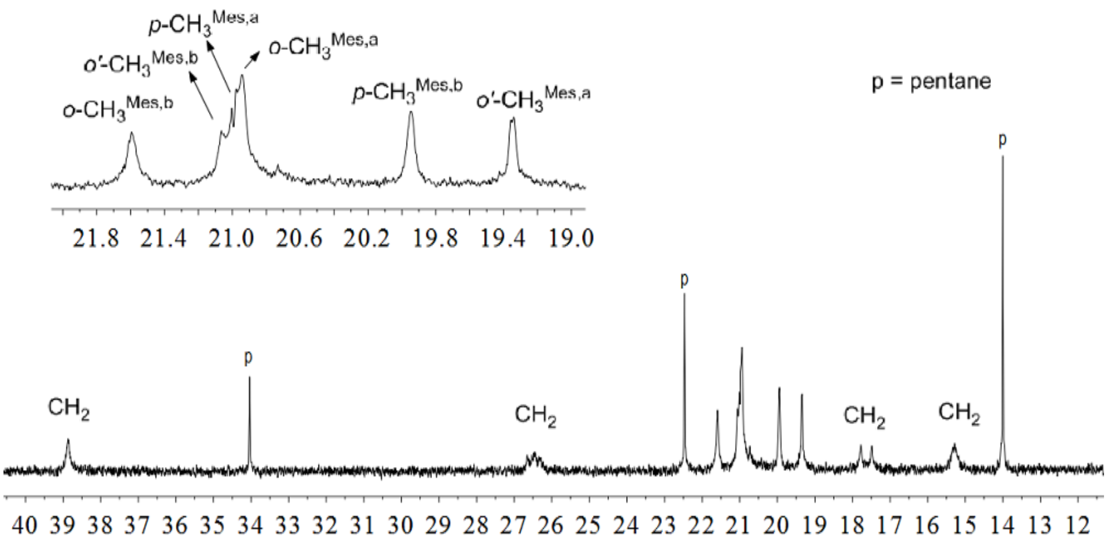

Figure S38. ^{13}**C{**^{1}**H} NMR** (126 MHz, 223 K, dichloromethane-d$_2$) spectrum of compound **17a**

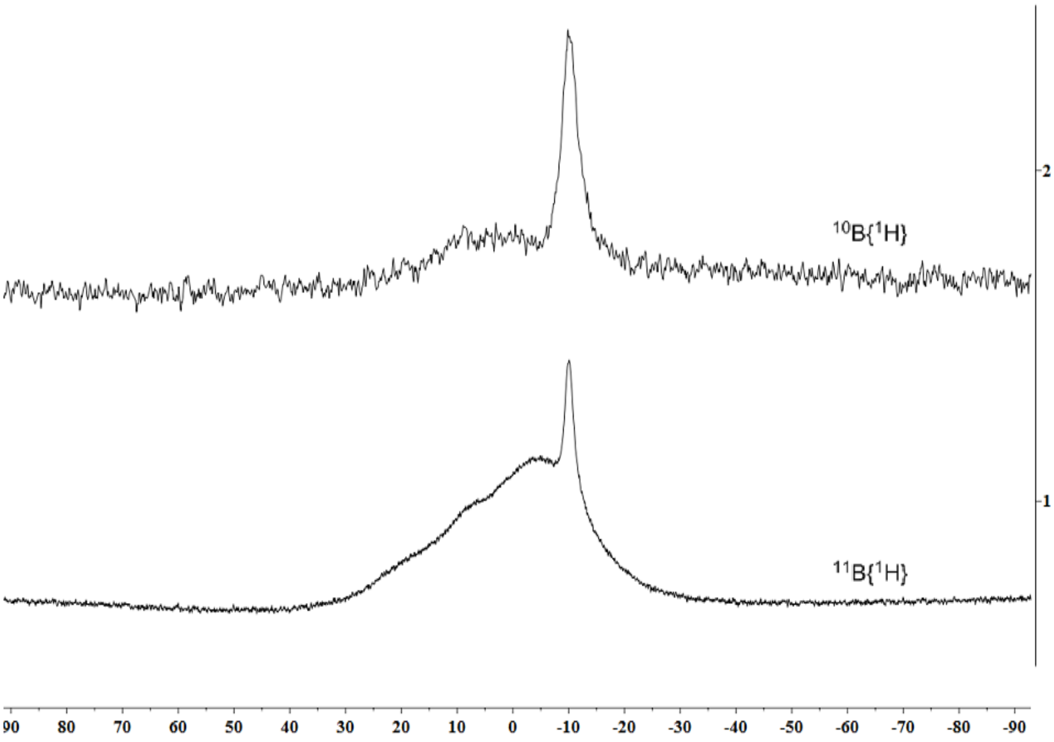

Figure S39. (1) ^{11}**B{**^{1}**H} NMR** (160 MHz, 299 K, dichloromethane-d$_2$) and (2) ^{10}**B NMR** (54 MHz, 299 K, dichloromethane-d$_2$) spectra of compound **17a**.

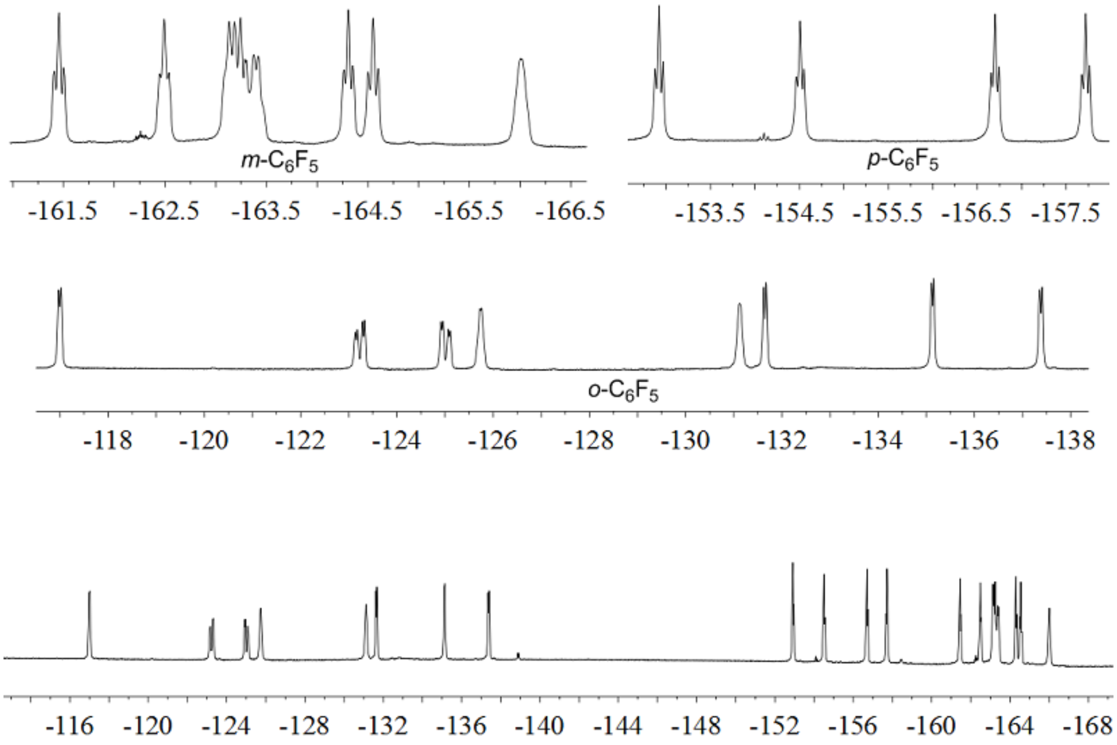

Figure S40. ^{19}**F NMR** (470 MHz, 223 K, dichloromethane-d_2) spectrum of compound **17a**

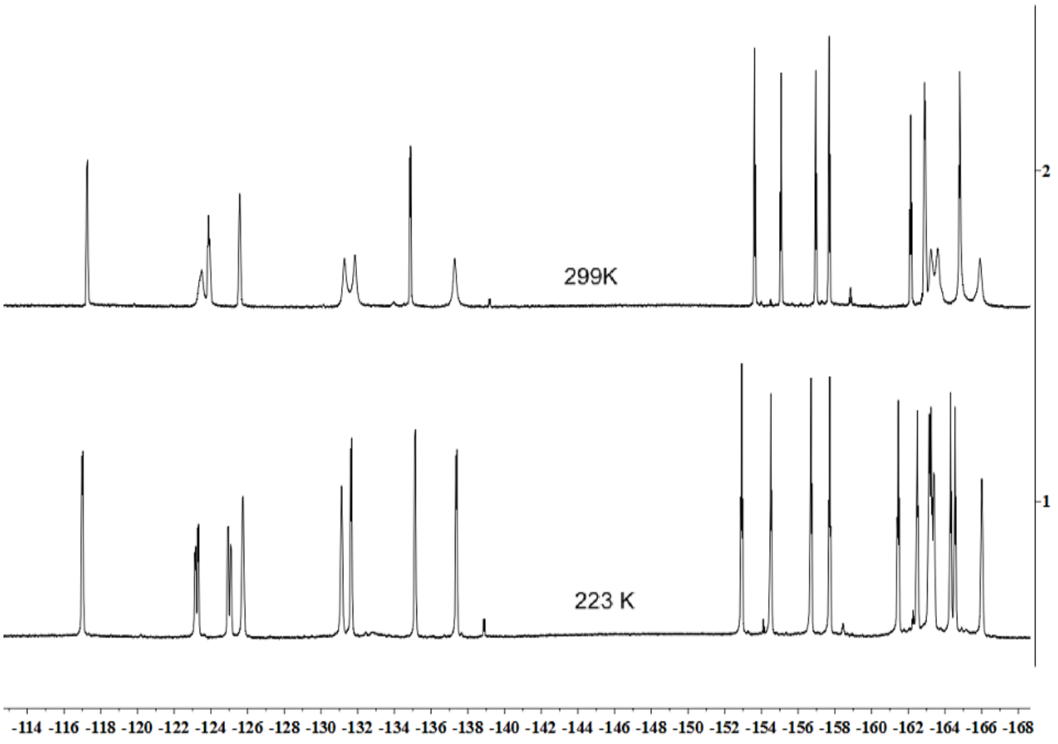

Figure S41. ^{19}**F NMR** (470 MHz, dichloromethane-d_2) spectra of compound **17a** at (1) 223 K and (2) 299 K

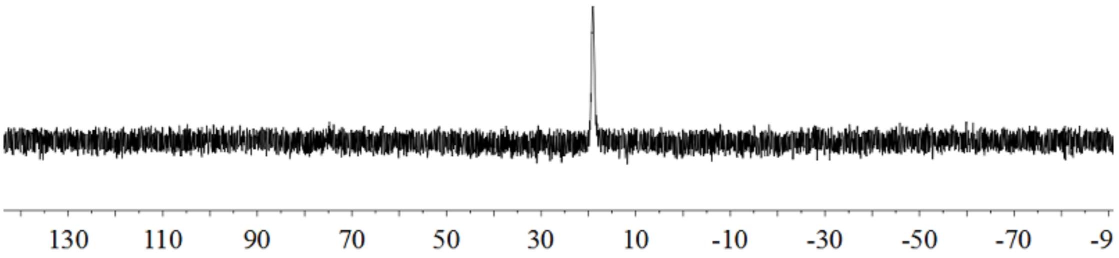

Figure S42. **$^{31}P\{^{1}H\}$ NMR** (202 MHz, 299 K, dichloromethane-d_2) of spectrum compound **17a**

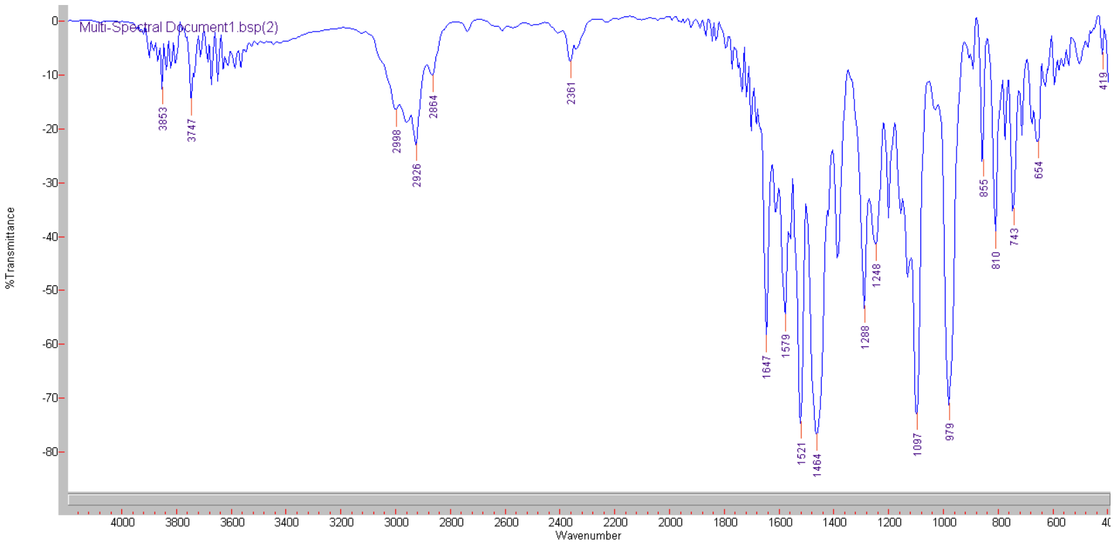

Figure S43. **IR**(KBr) spectrum of compound **17a**

Crystals suitable for the X-ray crystal structure analysis of compound **17a** were obtained from slow diffusion of pentane to a solution of the white solid in dichloromethane at -36 °C.

X-ray crystal structure analysis of compound 17a: A colorless needle-like specimen of $C_{106}H_{66}B_4F_{40}O_2P_2$, approximate dimensions 0.046 mm x 0.064 mm x 0.173 mm, was used for the X-ray crystallographic analysis. The X-ray intensity data were measured. A total of 1640 frames were collected. The total exposure time was 39.38 hours. The frames were integrated with the Bruker SAINT software package using a wide-frame algorithm. The integration of the data using an orthorhombic unit cell yielded a total of 130938 reflections to a maximum θ angle of 60.00° (0.89 Å resolution), of which 12974 were independent (average redundancy 10.092, completeness = 95.5%, R_{int} = 11.46%, R_{sig} = 10.86%) and 10263 (79.10%) were greater than $2\sigma(F^2)$. The final cell constants of a = 23.2043(10) Å, b = 30.9754(12) Å, c = 14.8030(6) Å, volume = 10639.8(8) $Å^3$, are based upon the refinement of the XYZ-centroids of 287 reflections above 20 σ(I) with 11.88° < 2θ < 73.31°. Data were corrected for absorption effects using the multi-scan method (SADABS). The ratio of minimum to maximum apparent transmission was 0.881. The calculated minimum and maximum transmission coefficients (based on crystal size) are 0.7900 and 0.9370. The final anisotropic full-matrix least-squares refinement on F^2 with 1399 variables converged at R1 = 10.81%, for the observed data and wR2 = 20.58% for all data. The goodness-of-fit was 1.161. The largest peak in the final difference electron density synthesis was 0.578 $e^-/Å^3$ and the largest hole was -0.465 $e^-/Å^3$ with an RMS deviation of 0.103 $e^-/Å^3$. On the basis of the final model, the calculated density was 1.396 g/cm^3 and F(000), 4512 e^-.

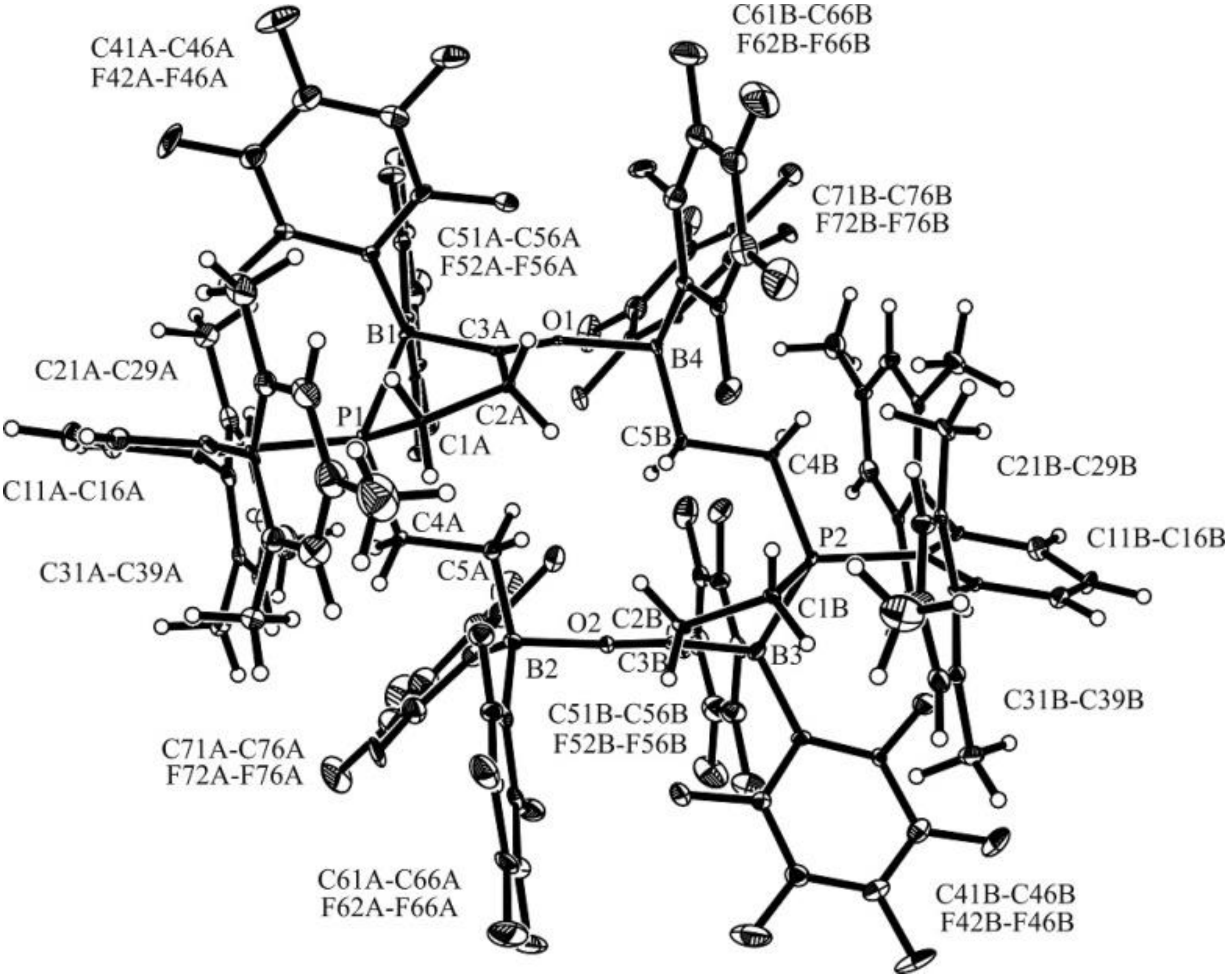

Figure S44. X-ray crystal structure analysis of compound **17a**

Preparation of ^{13}C labeled compound 17a

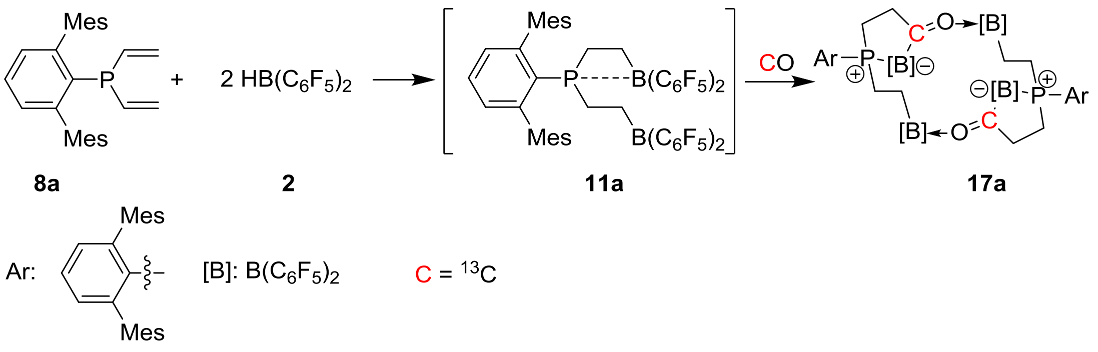

Scheme S10.

In the glove box, compound **8a** (19.9 mg, 0.05 mmol, 1.0 eq.) and compound **2** (34.6 mg, 0.10 mmol, 2.0 eq.) were mixed with pentane (1 mL) and stirred at room temperature for 1 hour to give a yellow solution. After the obtained solution was filled in a J-Young tube, it was carefully evacuated at -78 °C (dry ice / isopropanol bath) and then the solution was exposed to ^{13}CO (1.5 bar) for 10 min at room temperature to give a white suspension. Decantation of the suspension gave a white solid, which was washed with pentane (0.5 mL) and dried in vacuo to give a white solid. The white solid product was characterized by NMR and IR spectroscopy.
^{13}C{^{1}H} NMR (126 MHz, 223 K, dichloromethane-d$_2$)[key resonance] δ = 267.2 (d, $^{2}J_{PC}$ = 36.0 Hz, C=O).
IR(KBr): $\tilde{\nu}$ [cm^{-1}] = 1646 (m, C=C), 1521 (s), 1463 (s), 1289 (m), 1095 (s), 979 (s, =C-H).

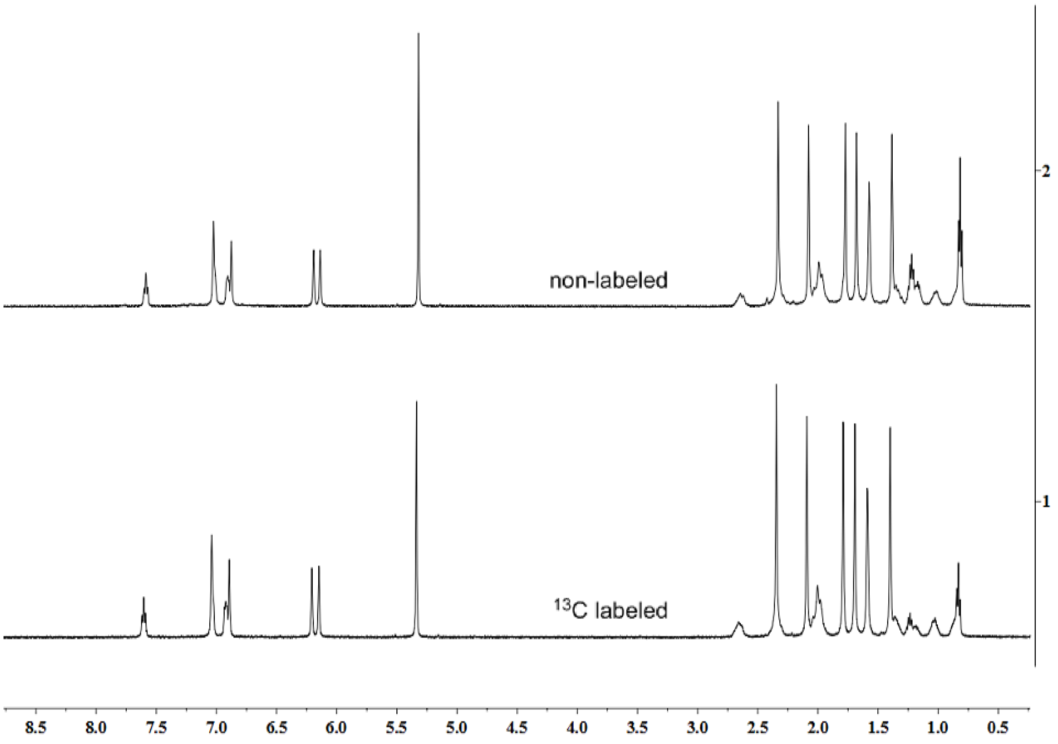

Figure S45. **^{1}H NMR** (500 MHz, 223 K, dichloromethane-d$_2$) spectra of (1) ^{13}C labeled and (2) non-labeled compound **17a**

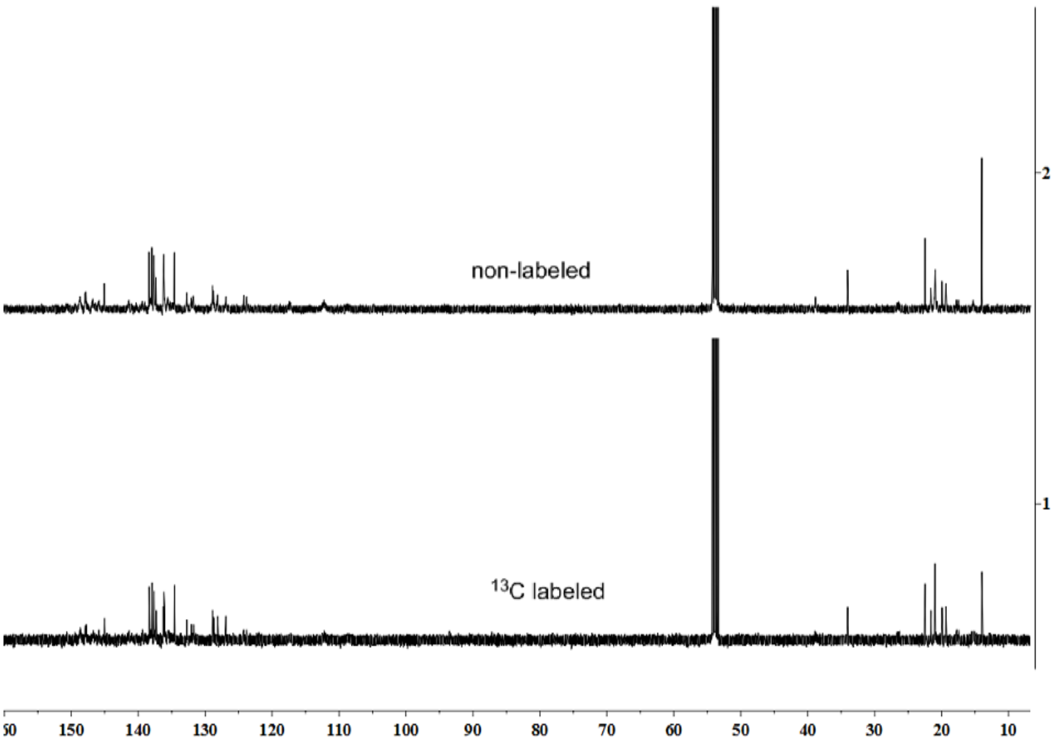

Figure S46. $^{13}C\{^1H\}$ **NMR** (126 MHz, 223 K, dichloromethane-d$_2$) spectra of (1) ^{13}C labeled and (2) non-labeled compound **17a**

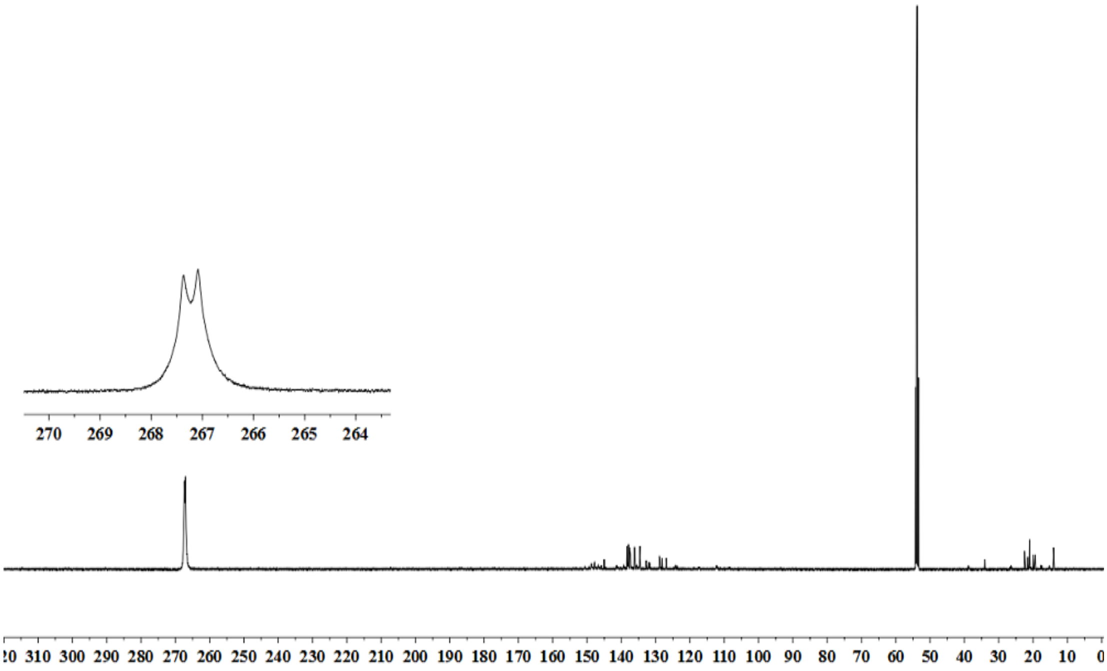

Figure S47. $^{13}C\{^1H\}$ **NMR** (126 MHz, 223 K, dichloromethane-d$_2$) spectrum of ^{13}C labeled compound **17a**

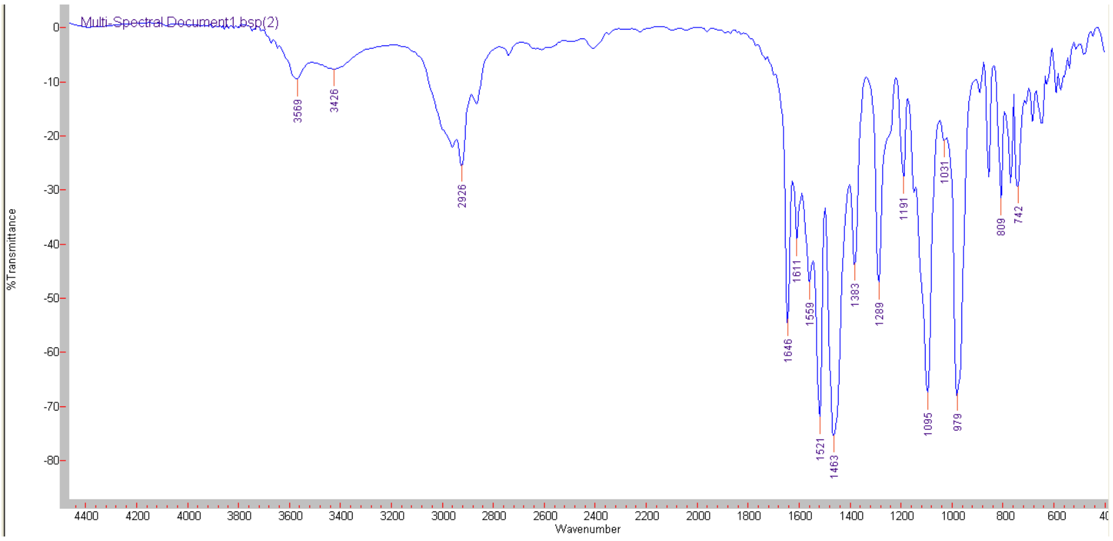

Figure S48. IR(KBr) spectrum of ^{13}C labeled compound **17a**

Preparation of compound 17b

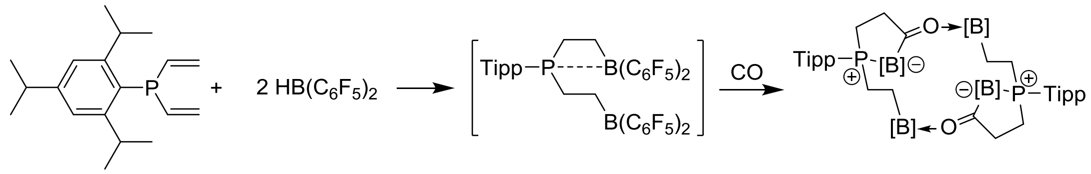

8b **2** **11b** **17b**

Tipp: 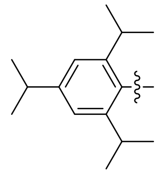 [B]: $B(C_6F_5)_2$

Scheme S11.

In a Schlenk flask, compound **8b** (57.7 mg, 0.20 mmol, 1.0 eq.) and compound **2** (145.3 mg, 0.42 mmol, 2.1 eq.) were mixed with pentane (6 mL) and stirred at room temperature for 24 h. After the reaction mixture was filtered via cannula, the obtained solution was cooled to -78 °C (dry ice / isopropanol bath) and then the flask was evacuated carefully. Then the dry ice bath was removed and the solution was exposed to CO gas (1.5 bar) and stirred at room temperature for 30 min to give a white suspension. Filtration by using a filter cannula gave a white solid. The white solid was washed with pentane (1 mL × 2) and dried in vacuo to finally give compound **17b** as a white solid (107.6 mg, 0.054 mmol, 54 %).

Elemental analysis (%) calcd. for $C_{88}H_{62}B_4F_{40}O_2P_2$: C 52.41, H 3.10; Found: C 52.11, H 2.99.

Melting point: 172 ^{o}C

IR(KBr): $\tilde{\nu}$ [cm^{-1}] = 1648 (m, C=C), 1588 (m, C=O), 1521 (s), 1465 (s), 1290 (s), 981 (s).

NMR data from a solution of the white solid in dichloromethane-d$_2$.

^{1}H NMR (500 MHz, 299 K, dichloromethane-d$_2$) δ = 7.09 (dd, $^4J_{PH}$ = 4.0 Hz, $^4J_{HH}$ = 1.8 Hz, 1H, *m'*-Tipp), 6.91 (dd, $^4J_{PH}$ = 4.8 Hz, $^4J_{HH}$ = 1.8 Hz, 1H, *m*-Tipp), 3.27, 3.22, 2.83, 2.68 (each m, each 1H, CH$_2^{CO}$), 2.87 (sept, $^3J_{HH}$ = 6.9 Hz, 1H, *p*-^{i}Pr), 2.74 (sept, $^3J_{HH}$ = 6.7 Hz, 1H, *o*-^{i}Pr), 2.58 (dsept, $^3J_{HH}$ = 6.5 Hz, $^4J_{PH}$ = 4.4 Hz, 1H, *o'*-^{i}Pr), 2.23/2.09, 1.90/1.37 (each m, each 1H, CH$_2$), 1.22/1.21 (each d, $^3J_{HH}$ = 6.9 Hz, each 3H, *p*-^{i}Pr), 1.29/0.80 (each d, $^3J_{HH}$ = 6.7 Hz, each 3H, *o*-^{i}Pr), 0.69/0.65 (each d, $^3J_{HH}$ = 6.5 Hz, each 3H, *o'*-^{i}Pr).

^{13}C{^{1}H} NMR (126 MHz, 299 K, dichloromethane-d$_2$) δ^{13}C = 155.3 (d, $^4J_{PC}$ = 3.1 Hz, *p*-Tipp), 154.9 (d, $^2J_{PC}$ = 4.1 Hz, *o*-Tipp), 154.5 (d, $^2J_{PC}$ = 16.6 Hz, *o'*-Tipp), 124.2 (d, $^3J_{PC}$ = 16.3 Hz, *m'*-Tipp), 124.1 (d, $^3J_{PC}$ = 14.4 Hz, *m*-Tipp), 114.0 (br d, $^1J_{PC}$ = 53.0 Hz, *i*-Tipp), 40.1 (br), n.o. (CH$_2^{CO}$), 34.7 (d, $^3J_{PC}$ = 10.1 Hz, *o'*-^{i}Pr), 34.3 (d, $^5J_{PC}$ = 1.1 Hz, *p*-^{i}Pr), 31.3 (d, $^3J_{PC}$ = 2.5 Hz, *o*-^{i}Pr), 25.2/24.3 (*o*-^{i}Pr), 24.4/23.7 (m)(*o'*-^{i}Pr), 23.3/23.2 (*p*-^{i}Pr), 23.0/16.7(br)(CH$_2$), [C_6F_5 not listed].

^{11}B{^{1}H} NMR (160 MHz, 299 K, dichloromethane-d$_2$) δ = -8.5 (ν$_{1/2}$ ~300 Hz).

^{10}B NMR (54 MHz, 299 K, dichloromethane-d$_2$) δ = -8.5 (ν$_{1/2}$ ~300 Hz).

^{19}F NMR (470 MHz, 223 K, dichloromethane-d$_2$) δ = -121.3(1F), -124.7(1F), -126.1(1F), -128.4(1F), -134.6(1F), -134.9(2F), -137.9(1F)(each m, *o*-C_6F_5), -152.3 (t, $^3J_{FF}$ = 20.8 Hz), -154.6 (br m), -155.8 (t, $^3J_{FF}$ = 20.8 Hz), -158.9 (t, $^3J_{FF}$ = 20.6 Hz)(each 1F, *p*-C_6F_5), -160.9, -161.8, -162.0, -163.8, -164.0, -164.1, -164.6, 164.9 (each m, each 1F, *m'*-C_6F_5).

^{31}P{^{1}H} NMR (202 MHz, 299 K, dichloromethane-d$_2$) δ = 21.0 (ν$_{1/2}$ ~ 100 Hz).

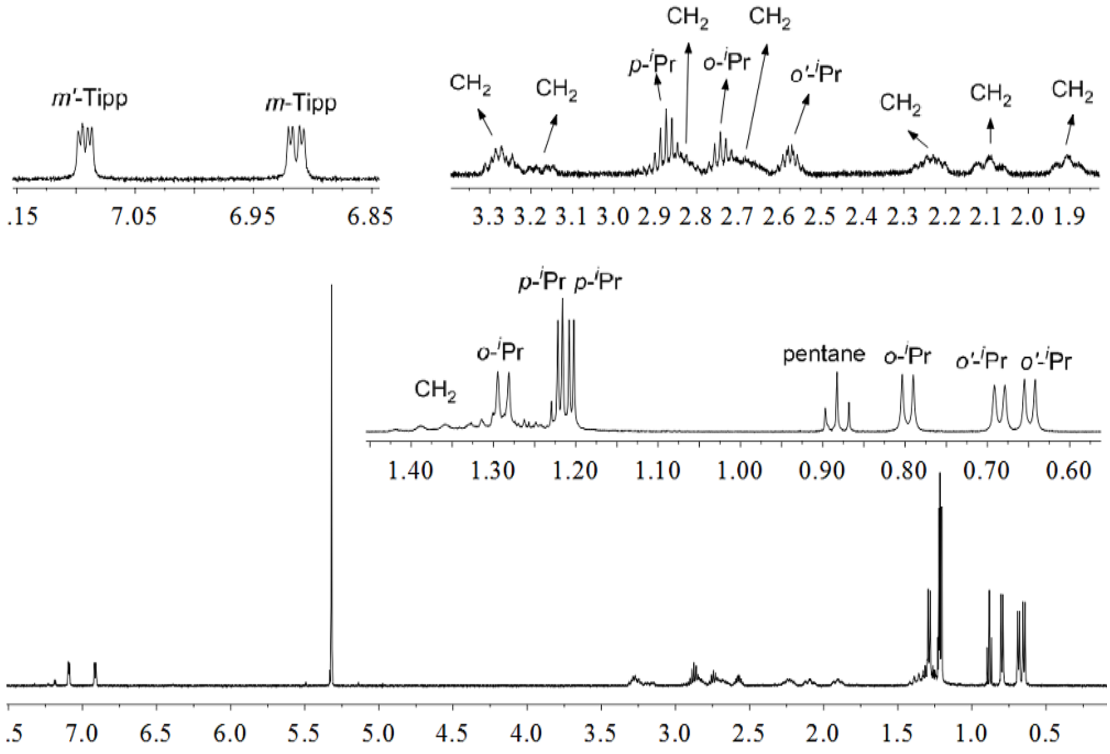

Figure S49. **^{1}H NMR** (500 MHz, 299 K, dichloromethane-d$_2$) spectrum of compound **17b**

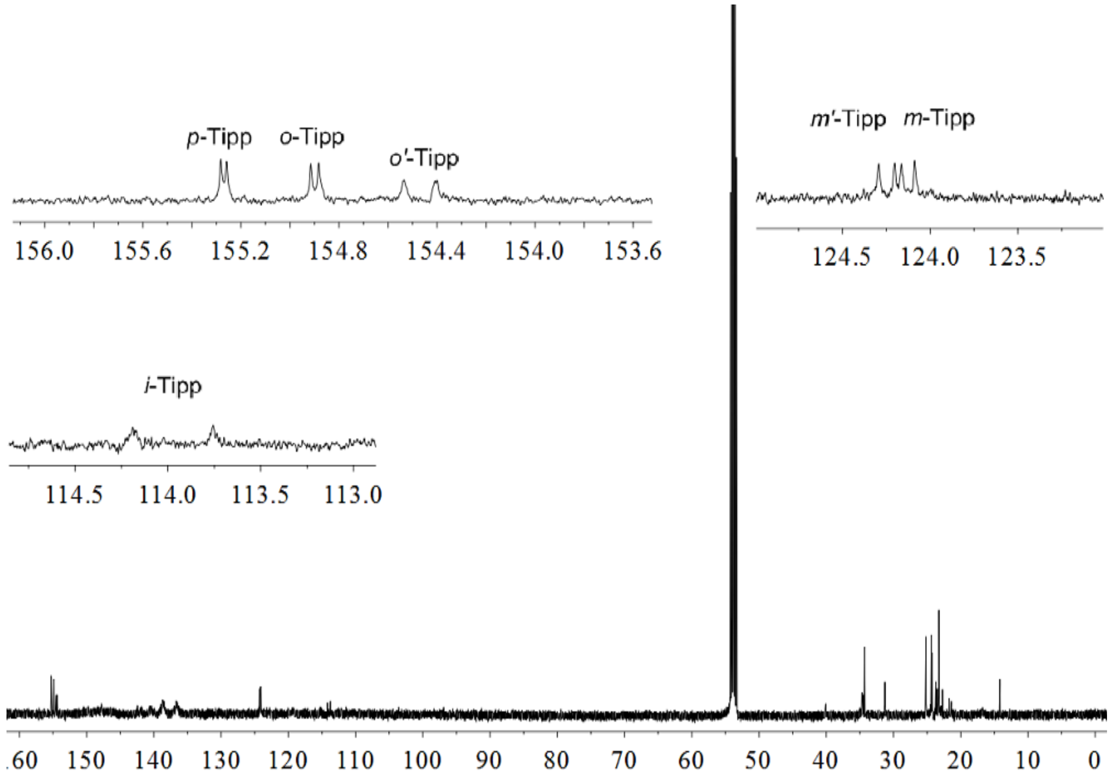

Figure S50. **^{13}C{^{1}H}** (126 MHz, 299 K, dichloromethane-d$_2$) spectrum of compound **17b**

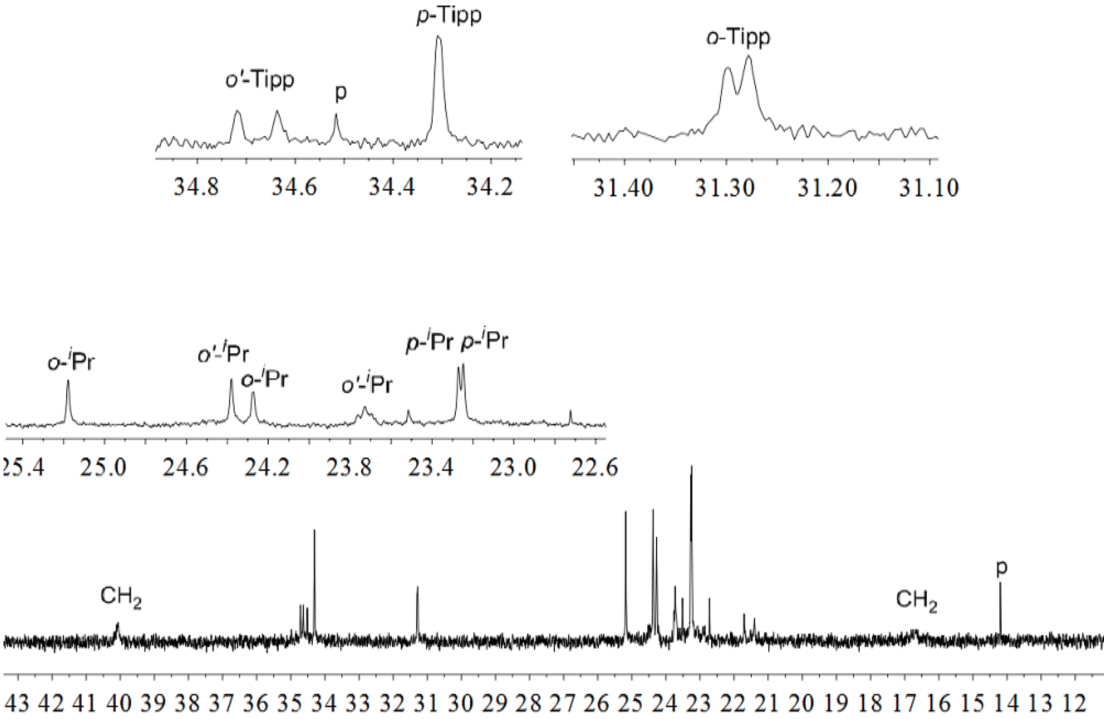

Figure S51. ^{13}**C{**^{1}**H}** (126 MHz, 299 K, dichloromethane-d$_2$) spectrum of compound **17b**

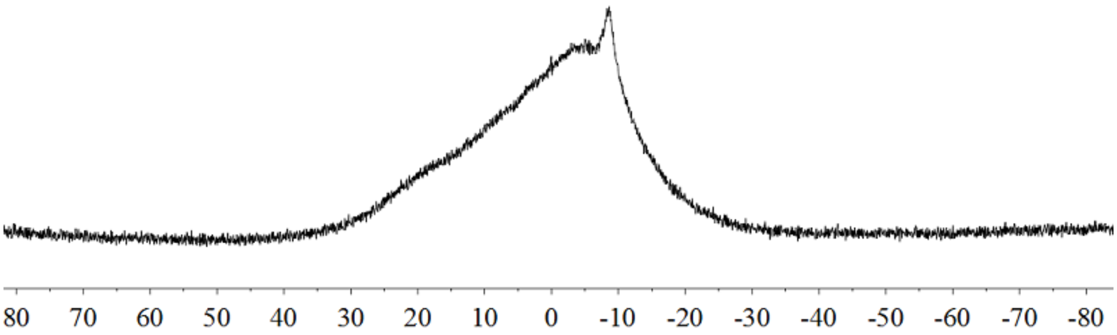

Figure S52. ^{11}**B{**^{1}**H} NMR** (160 MHz, 299 K, dichloromethane-d$_2$) spectrum of compound **17b**

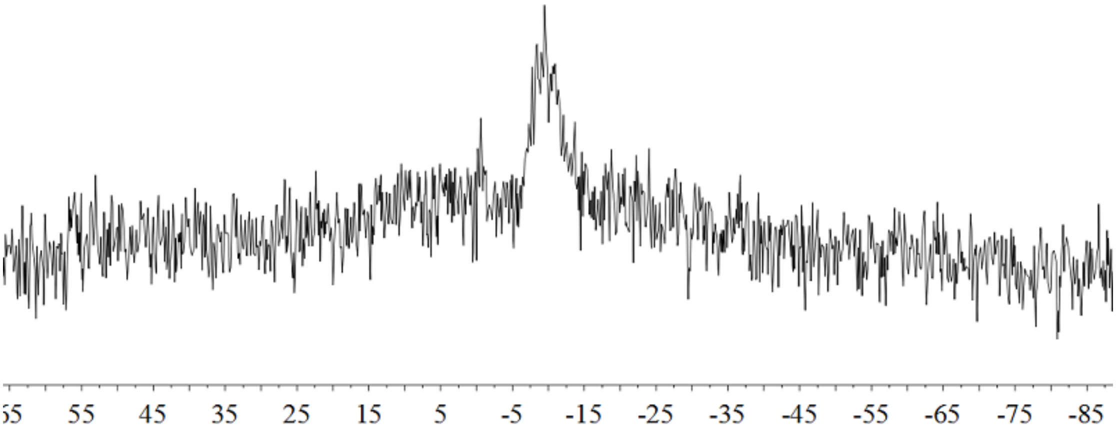

Figure S53. ^{10}**B NMR** (54 MHz, 299 K, dichloromethane-d$_2$) spectrum of compound **17b**

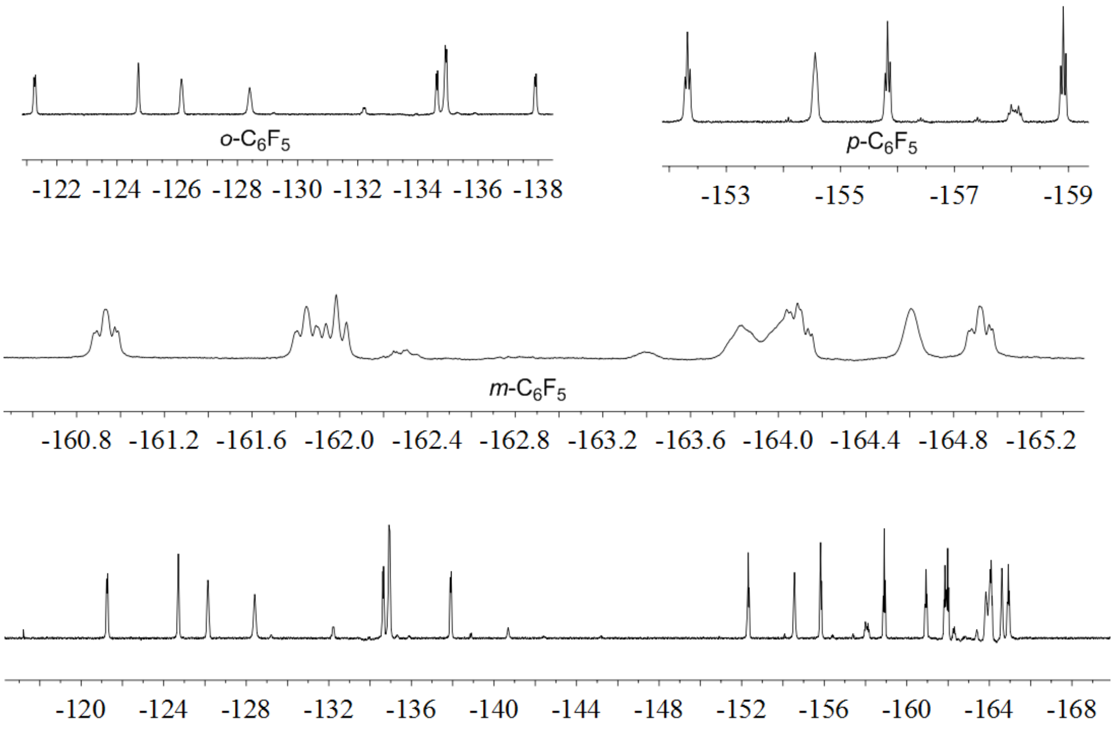

Figure S54. ^{19}**F NMR** (470 MHz, 223 K, dichloromethane-d$_2$) spectrum of compound **17b**

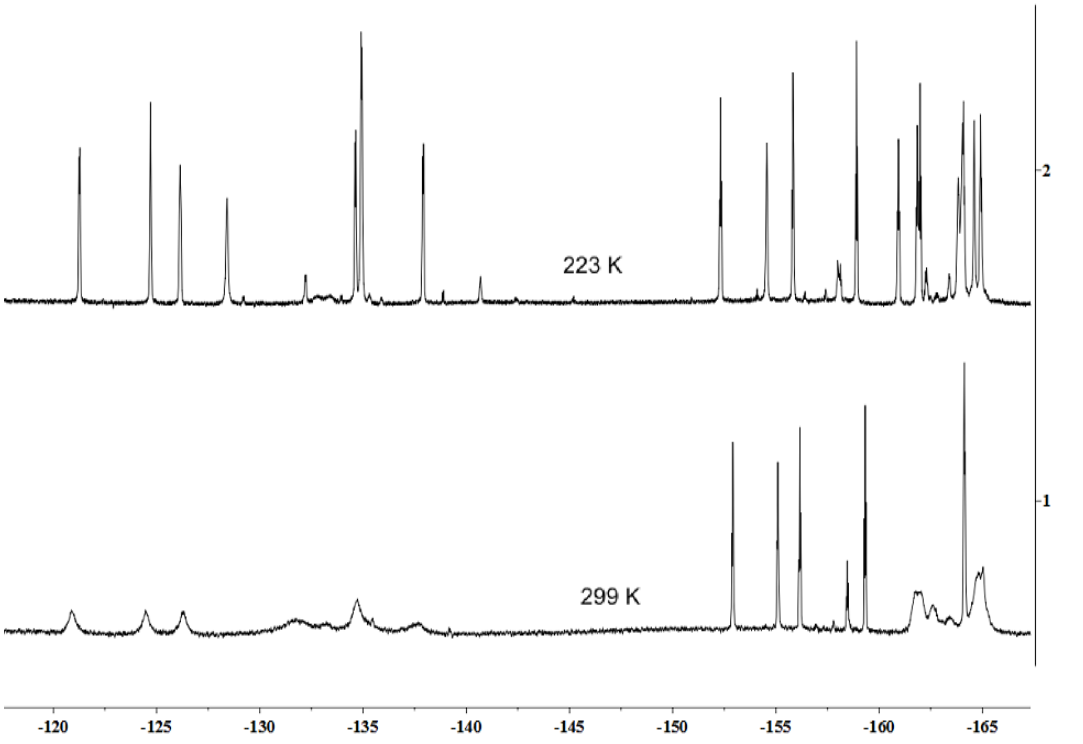

Figure S55. ^{19}**F NMR** (470 MHz, dichloromethane-d$_2$) spectra of compound **17b** at (1) 299K and (2) 223K.

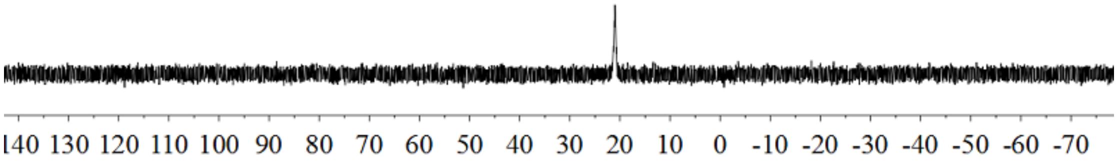

Figure S56. ^{31}**P{**^{1}**H} NMR** (202 MHz, 299 K, dichloromethane-d_2) spectrum of compound **17b**

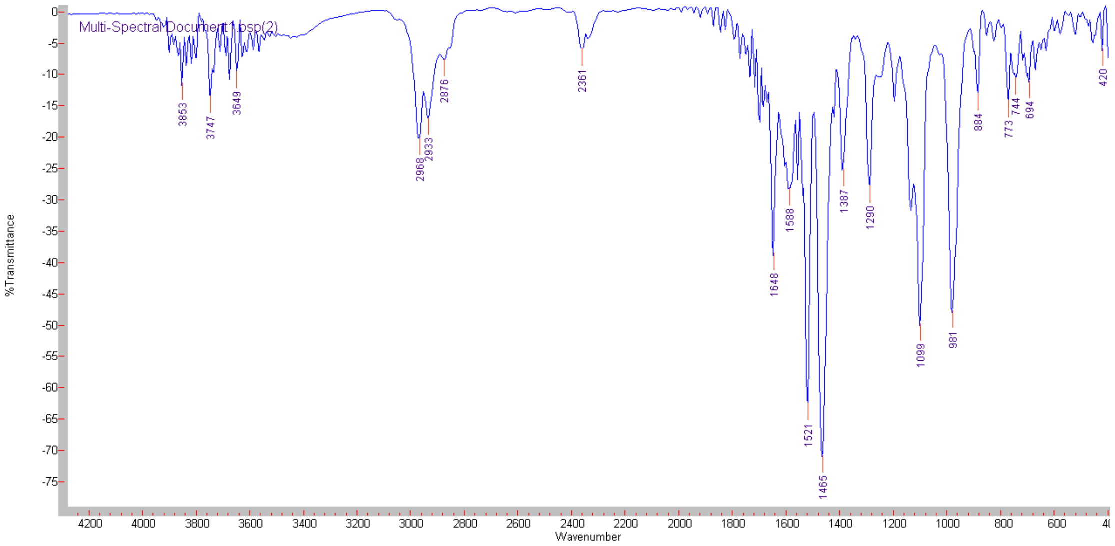

Figure S57. IR(KBr) spectrum of compound **17b**

Crystals suitable for the X-ray crystal structure analysis of compound **17b** were obtained from slow diffusion of pentane to a solution of the white solid in dichloromethane at -36 ^{o}C.

X-ray crystal structure analysis of compound 17b: A prism-like specimen of $C_{88}H_{62}B_4F_{40}O_2P_2$, approximate dimensions 0.093 mm x 0.161 mm x 0.223 mm, was used for the X-ray crystallographic analysis. The X-ray intensity data were measured. A total of 1327 frames were collected. The total exposure time was 14.74 hours. The frames were integrated with the Bruker SAINT software package using a narrow-frame algorithm. The integration of the data using a triclinic unit cell yielded a total of 80163 reflections to a maximum θ angle of 26.37° (0.80 Å resolution), of which 17300 were independent (average redundancy 4.634, completeness = 99.8%, R_{int} = 4.21%, R_{sig} = 3.78%) and 13572 (78.45%) were greater than 2σ(F^2). The final cell constants of a = 11.1630(5) Å, b = 16.2067(8) Å, c = 24.8378(11) Å, α = 76.4320(10)°, β = 87.505(2)°, γ = 76.034(2)°, volume = 4238.6(3) Å^3, are based upon the refinement of the XYZ-centroids of 9782 reflections above 20 σ(I) with 4.756° < 2θ < 54.95°. Data were corrected for absorption effects using the multi-scan method (SADABS). The ratio of minimum to maximum apparent transmission was 0.958. The calculated minimum and maximum transmission coefficients (based on crystal size) are 0.9590 and 0.9830. The structure was solved and refined using the Bruker SHELXTL Software Package, using the space group *P*-1, with Z = 2 for the formula unit, $C_{88}H_{62}B_4F_{40}O_2P_2$. The final anisotropic full-matrix least-squares refinement on F^2 with 1237 variables converged at R1 = 3.90%, for the observed data and wR2 = 8.60% for all data. The goodness-of-fit was 1.024. The largest peak in the final difference electron density synthesis was 0.452 $e^-/Å^3$ and the largest hole was -0.361 $e^-/Å^3$ with an RMS deviation of 0.056 $e^-/Å^3$. On the basis of the final model, the calculated density was 1.580 g/cm^3 and F(000), 2032 e^-.

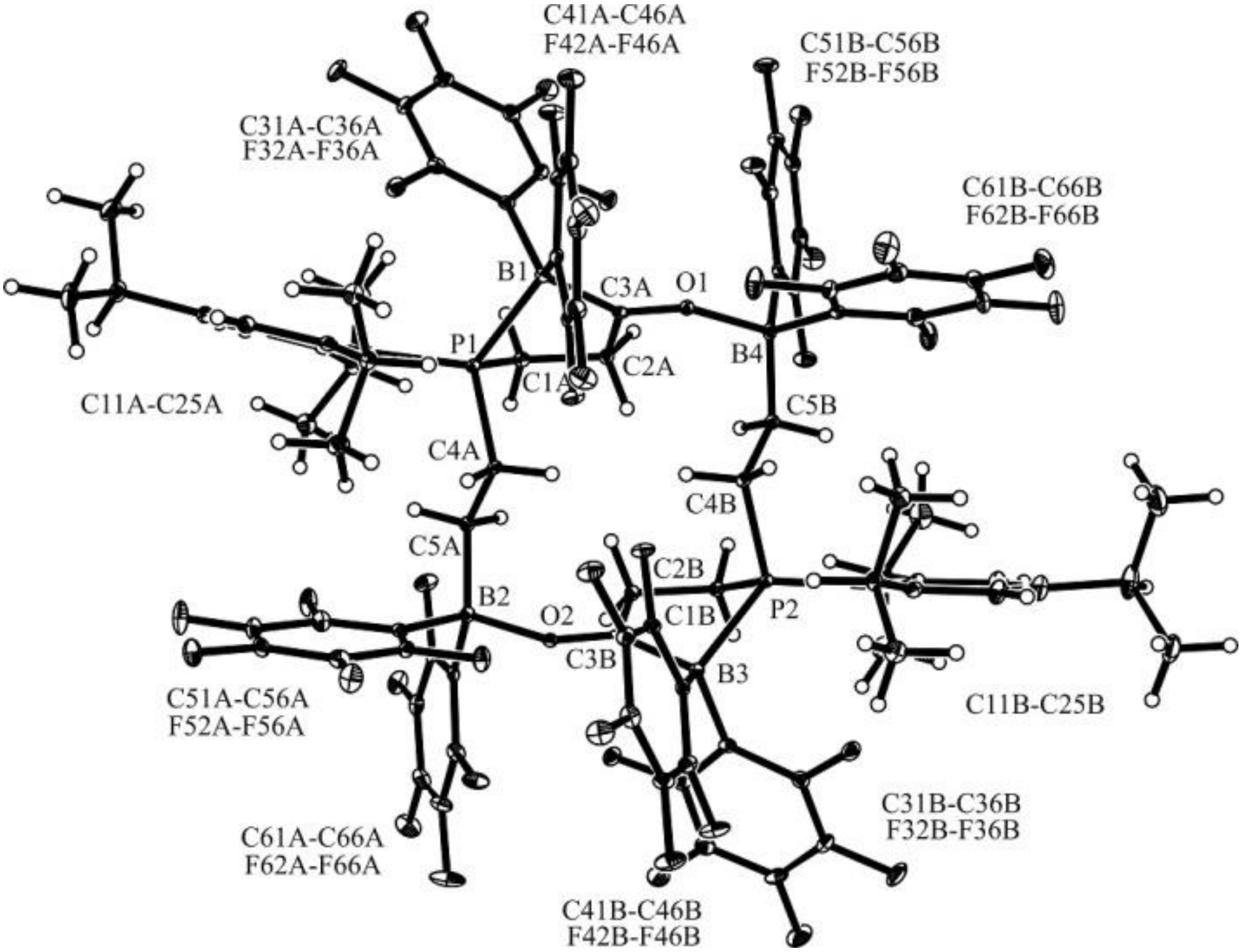

Figure S58. X-ray crystal structure analysis of compound **17b**

Preparation of ^{13}C labeled compound 17b

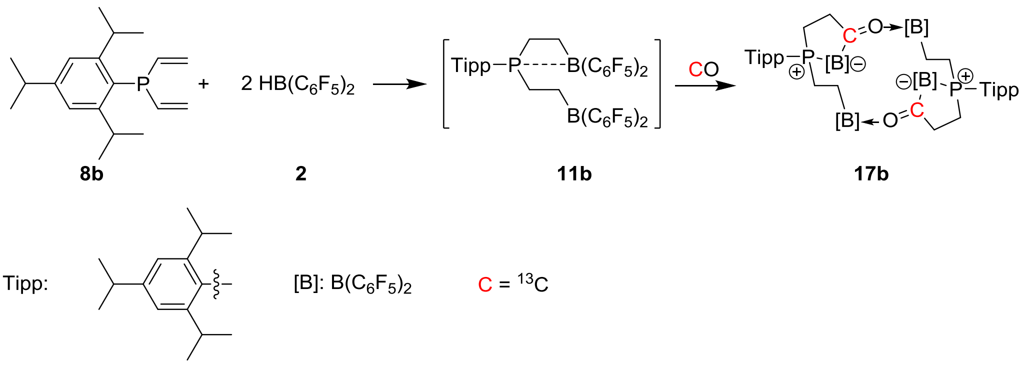

Scheme S12.

In the glove box, a solution of compound **8b** (14.4 mg, 0.05 mmol, 1.0 eq.) and compound **2** (36.3 mg, 0.105 mmol, 2.1 eq.) in pentane (1 mL) was stirred for 24 h at room temperature and then filtered. Subsequently the yellow solution was filled in a J-Young tube which was carefully evacuated at -78 °C (dry ice / isopropanol bath) and then the solution was exposed to ^{13}CO (1.5 bar) at room temperature for 10 min to give a white suspension. After decanting, the collected solid was washed with pentane (0.5 mL) and dried under vacuum to give compound f**17b** as a white solid. The white solid was characterized by NMR and IR spectroscopy.

^{13}C{^{1}H} NMR (126 MHz, 299 K, dichloromethane-d$_2$)[key resonance] δ = 269.0 (C=O).

IR(KBr): $\tilde{\nu}$ [cm^{-1}] = 1646 (m, C=C), 1547 (C=O), 1521 (s), 1463 (s), 1289 (m), 1095 (s), 979 (s).

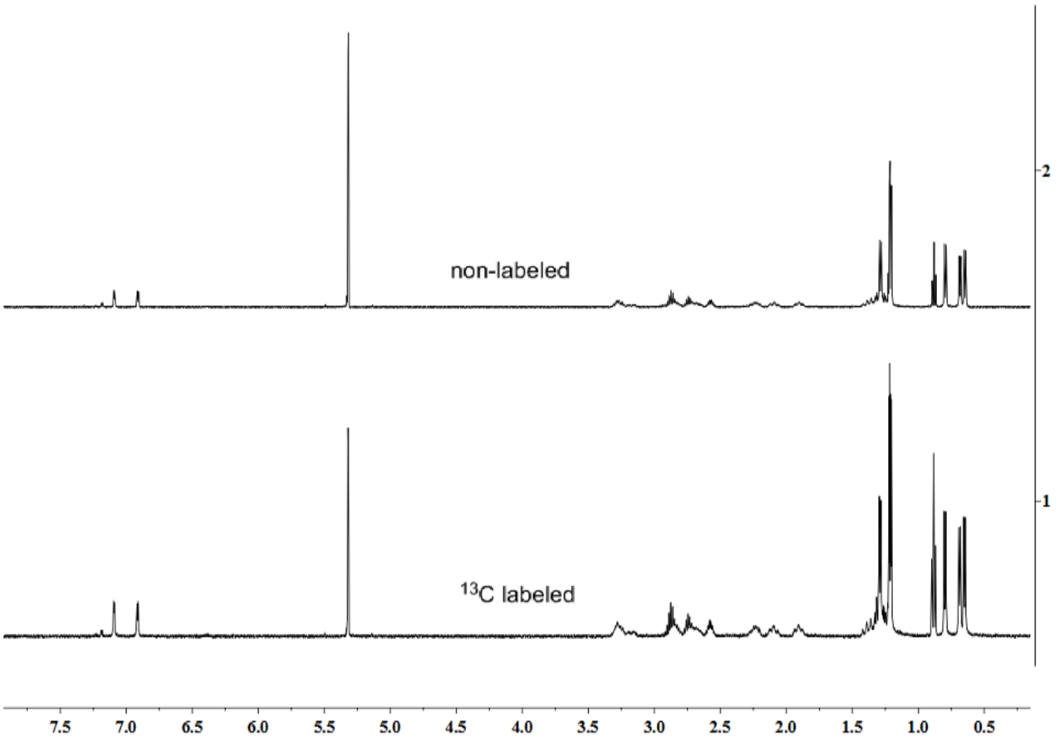

Figure S59. **^{1}H NMR** (500 MHz, 299 K, dichloromethane-d$_2$) of (1) ^{13}C labeled and (2) non-labeled compound **17b**

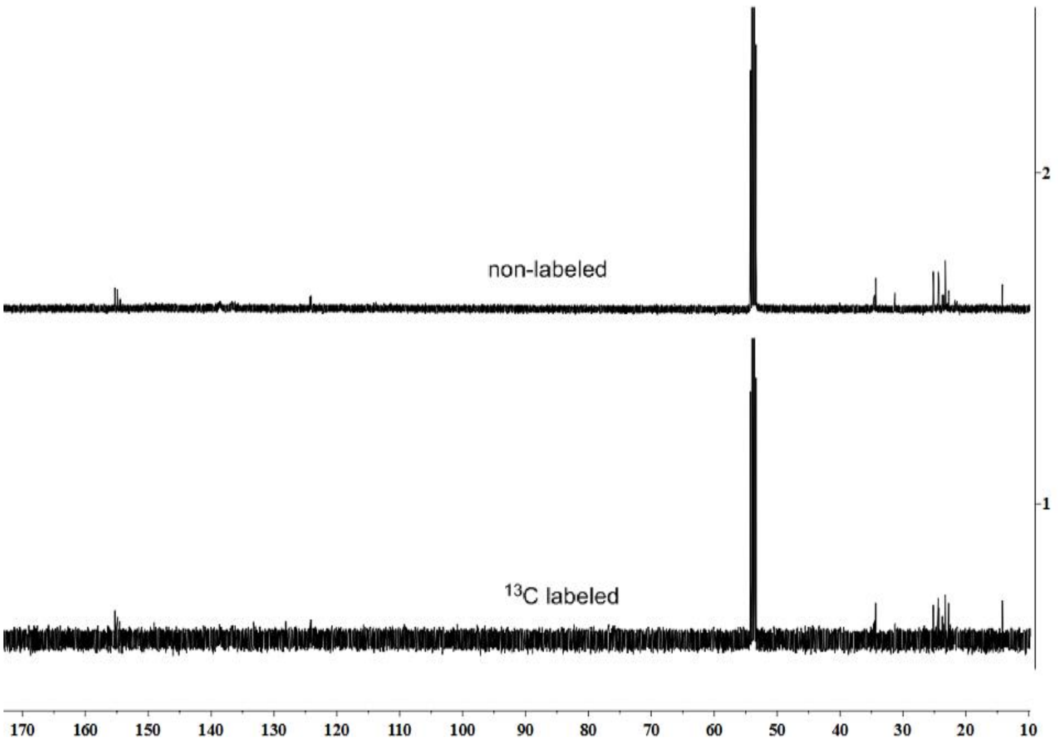

Figure S60. **$^{13}C\{^1H\}$** (126 MHz, 299 K, dichloromethane-d_2) of (1) ^{13}C labeled and (2) non-labeled compound **17b**

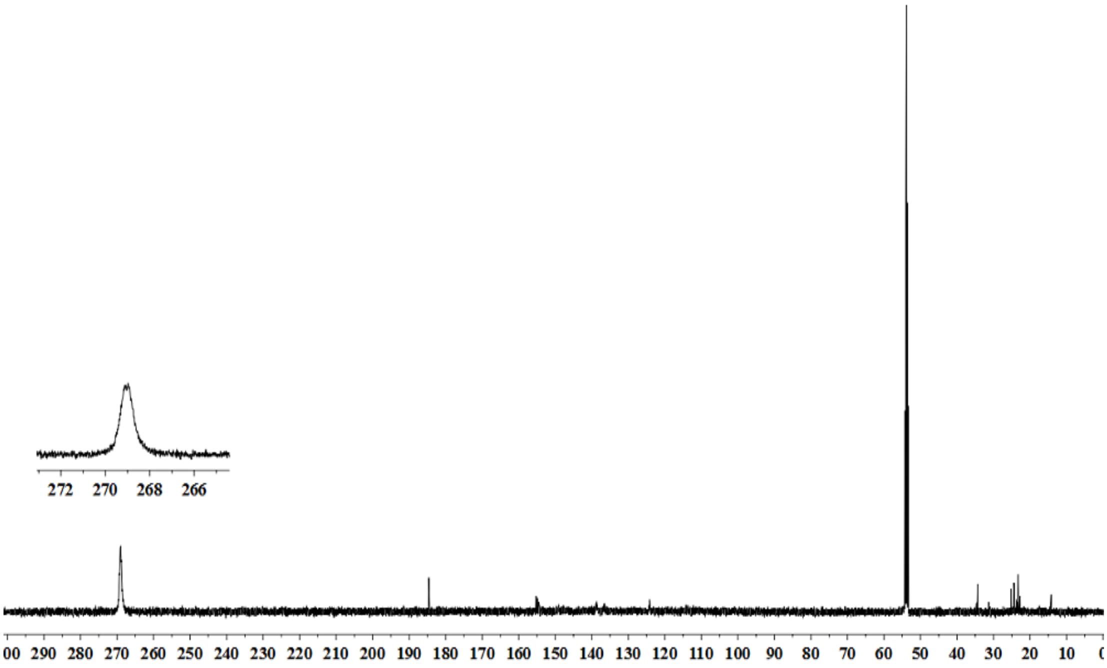

Figure S61. **$^{13}C\{^1H\}$** (126 MHz, 299 K, dichloromethane-d_2) spectrum of ^{13}C labeled compound **17b**

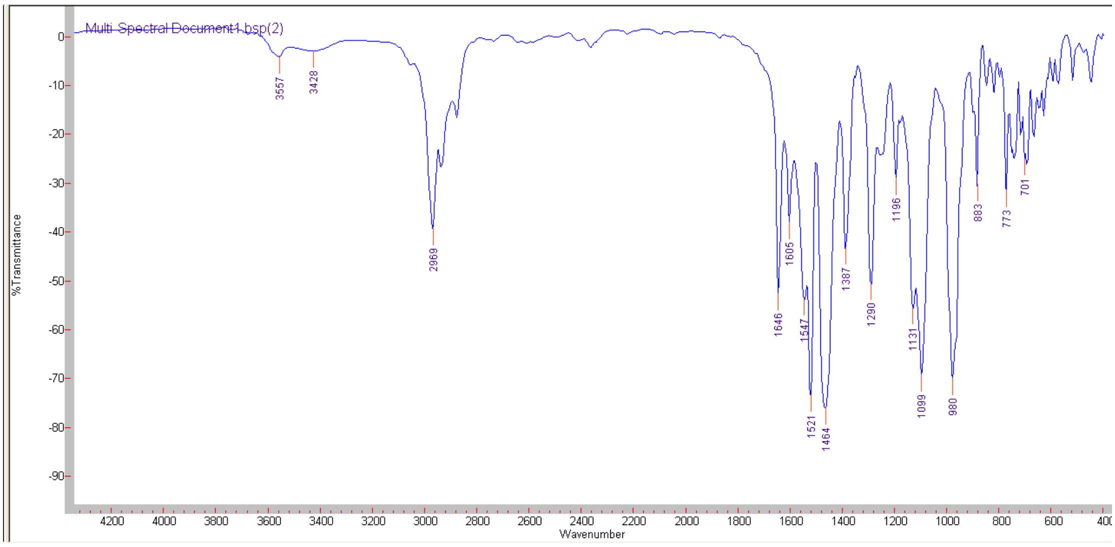

Figure S62. IR(KBr) spectrum of ^{13}C labeled compound **17b**

In situ generation of compound 18c

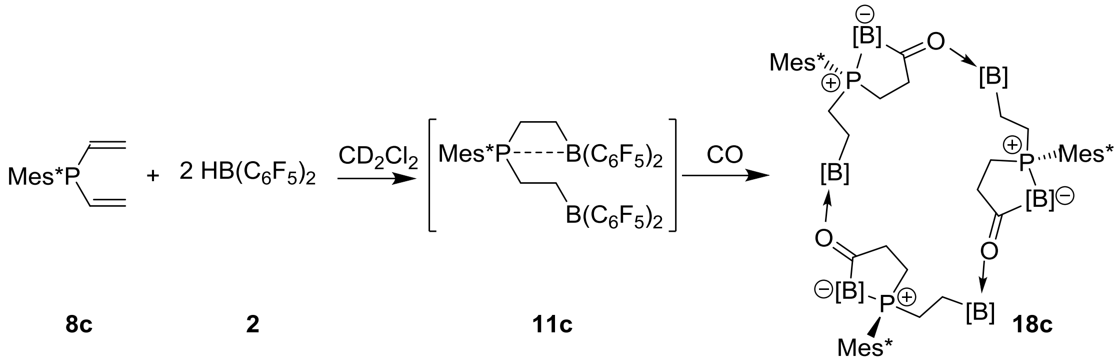

8c **2** **11c** **18c**

[B]: $B(C_6F_5)_2$

Scheme S13.

In a Schlenk flask, compound **8c** (16.5 mg, 0.05 mmol, 1.0 eq.) and compound **2** (34.6 mg, 0.10 mmol, 2.0 eq.) were mixed with dichloromethane-d_2 (2 mL) and stirred at room temperature for 15 min to give a yellow solution. After the solution (1 mL) was transferred to a J-Young NMR tube, it was cooled to -78 °C (dry ice / isopropanol bath). Then the NMR tube was evacuated carefully and subsequntly the reaction mixture was exposed to CO gas (1.5 bar) to give a colorless solution at -78 °C. The *in situ* generated solution was characterized by NMR experiments.

^{1}H NMR (500 MHz, 243 K, dichloromethane-d_2) δ = 7.50, 6.95 (each m, each 1H, *m*-Mes*^{a}), 7.34, 6.86 (each m, each 1H, *m*-Mes*^{b}), 7.33, 7.12 (each m, each 1H, *m*-Mes*^{c}), 3.38(2H), 3.35/2.73(each 1H), 3.21/2.13(each 1H), 3.17/2.83(each 1H), 3.13/2.19(each 1H), 2.87/1.43(each 1H), 2.69/2.22(each 1H), 2.63/1.95(each 1H), 2.53/2.09(each 1H), 2.03/0.52(each 1H), 1.04(2H), 0.95/0.06(each 1H)(CH_2)[1], 1.43, 0.78 (each s, each 9H, *o*-^{t}Bu^a), 1.23 (s, 9H, *p*-^{t}Bu^a), 1.20 (s, 9H, *p*-^{t}Bu^c), 1.18 (s, 9H, *p*-^{t}Bu^b), 0.99, 0.88 (each s, each 9H, *o*-^{t}Bu^c), 0.93, 0.87 (each s, each 9H, *o*-^{t}Bu^b). [[1] assignments were extracted fom the ghsqc NMR experiment, see below].

^{13}C{^{1}H} (126 MHz, 243 K, dichloromethane-d_2) δ = 161.3, 158.6 (*o*-Mes*^{a}), 161.2, 158.0 (*o*-Mes*^{b}), 160.7, 159.6 (*o*-Mes*^{c}), 154.4 (d, $^4J_{PC}$ = 3.9 Hz, *p*-Mes*^{c}), 153.8 (d, $^4J_{PC}$ = 3.5 Hz, *p*-Mes*^{a}), 153.6 (d, $^4J_{PC}$ = 3.5 Hz, *p*-Mes*^{b}), 128.8 (d, $^3J_{PC}$ = 11.6 Hz), 122.9 (d, $^3J_{PC}$ = 12.9 Hz)(*m*-Mes*^{a}), 128.6 (d, $^3J_{PC}$ = 11.4 Hz), 122.2 (d, $^3J_{PC}$ = 13.2 Hz)(*m*-Mes*^{b}), 127.2 (d, $^3J_{PC}$ = 10.6 Hz), 124.0 (d, $^3J_{PC}$ = 13.0 Hz)(*m*-Mes*^{c}), 114.4 (d, $^1J_{PC}$ = 57.3 Hz, *i*-Mes*^{b}), 113.2 (d, $^1J_{PC}$ = 59.0 Hz, *i*-Mes*^{a}), 108.5 (d, $^1J_{PC}$ = 53.0 Hz, *i*-Mes*^{c}), 41.5 (d, $^3J_{PC}$ = 3.5 Hz), 40.3 (d, $^1J_{PC}$ = 2.1 Hz)(*o*-^{t}Bu^c), 41.3 (br), 39.7 (d, $^3J_{PC}$ = 3.1 Hz)(*o*-^{t}Bu^a), 41.1 (d, $^1J_{PC}$ = 3.1 Hz), 39.6 (d, $^1J_{PC}$ = 3.5 Hz)(*o*-^{t}Bu^b), 34.37 (d, $^5J_{PC}$ = 1.6 Hz, *p*-^{t}Bu^a), 34.36 (d, $^5J_{PC}$ = 1.8 Hz, *p*-^{t}Bu^c), 34.3 (*p*-^{t}Bu^b), 33.6, 32.5 (each br m, *o*-^{t}Bu^a), 32.9, 32.6 (each br m, *o*-^{t}Bu^b), 32.8(br m), 32.4(br)(*o*-^{t}Bu^c), 30.1 (*p*-^{t}Bu^a), 30.0 (*p*-^{t}Bu^{b,c}), 40.9, 40.4, 38.0, 27.6, 26.8, 25.1, 24.3, 23.6, 21.3, 17.5, 16.0, 15.4 (CH_2)[1]. [C_6F_5 not listed; [1] chemical shifts extracted fom the ghsqc NMR experiment, see below]

^{11}B{^{1}H} NMR (160 MHz, 193 K, dichloromethane-d_2) δ = -10.9 ($\nu_{1/2}$ ~ 200 Hz).

^{11}B{^{1}H} NMR (160 MHz, 299 K, dichloromethane-d_2) δ = -8.7 ($\nu_{1/2}$ ~ 600 Hz).

^{31}P{^{1}H} NMR (202 MHz, 243 K, dichloromethane-d_2) δ = 17.2 ($\nu_{1/2}$ ~ 70 Hz, 1P, P^b), 15.8 ($\nu_{1/2}$ ~ 110 Hz, 1P, P^a), 14.2 ($\nu_{1/2}$ ~ 100 Hz, 1P, P^c).

^{19}F NMR (470 MHz, 243 K, dichloromethane-d_2) δ = -115.6(1F), -116.5(1F), -118.3(1F), -118.5(1F), -121.7(1F), -123.7(1F), -124.2(1F), -129.2(3F), -129.9(1F), -130.7(1F), -132.2(1F), -132.3(1F), -132.9(1F), -133.1(1F), -133.2(1F), -133.8(1F), -135.6(1F), -137.8(3F), -138.6(1F), -139.3(1F)(each m, *o*-C_6F_5), -150.7(1F), -153.2(1F), -153.7(1F), -153.8(1F), -154.6(1F), -154.9 (2F), -155.1(1F), -155.4 (1F), -157.7(1F), -158.0(1F), -158.3(1F)(each m, *p*-C_6F_5), -157.2(1F), -160.1(1F), -161.4(1F), -161.5(1F), -161.9(5F), -162.1(1F), -162.5(5F), -163.2(1F), -163.5(1F), -163.6(1F), -164.0(2F), -164.3(1F), -164.4(1F), -164.5(1F), -164.9(1F)(each m, *m*-C_6F_5).

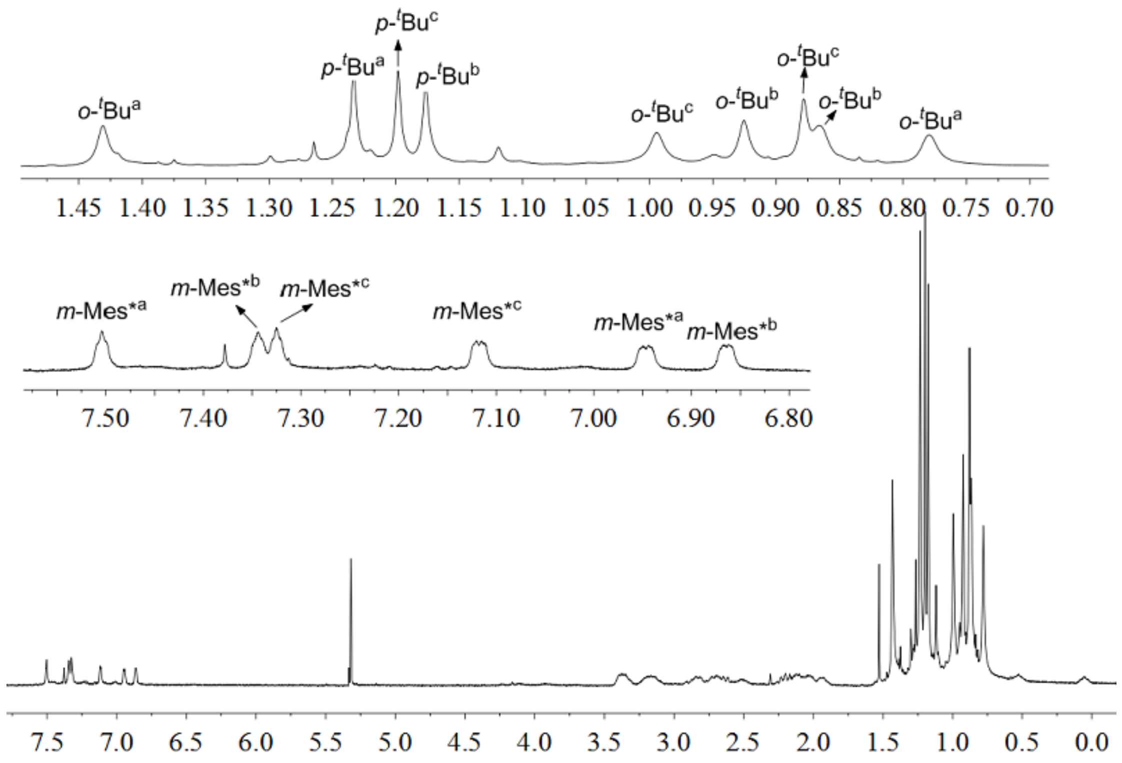

Figure S63. ^{1}**H NMR** (500 MHz, 243 K, dichloromethane-d_2) spectrum of *in situ* generated compound **18c**.

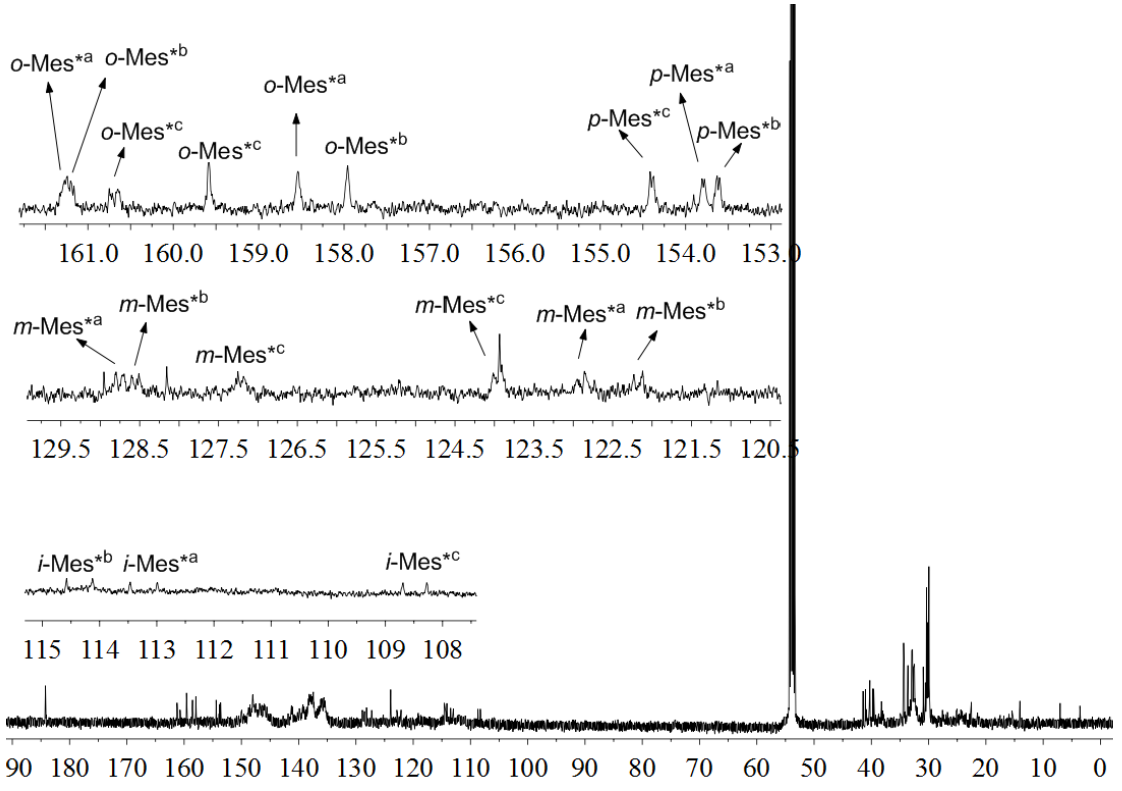

Figure S64. ^{13}**C{^{1}H}** (126 MHz, 243 K, dichloromethane-d_2) spectrum of *in situ* generated compound **18c**

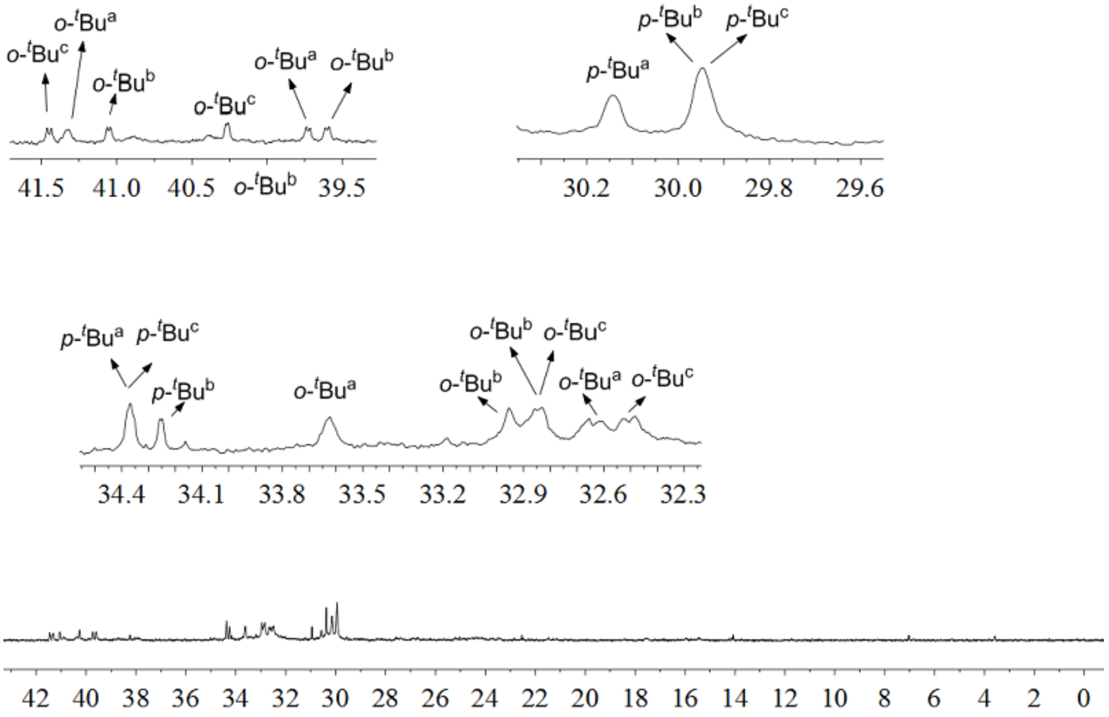

Figure S65. ^{13}**C{**^{1}**H}** (126 MHz, 243 K, dichloromethane-d$_2$) spectrum of *in situ* generated compound **18c**

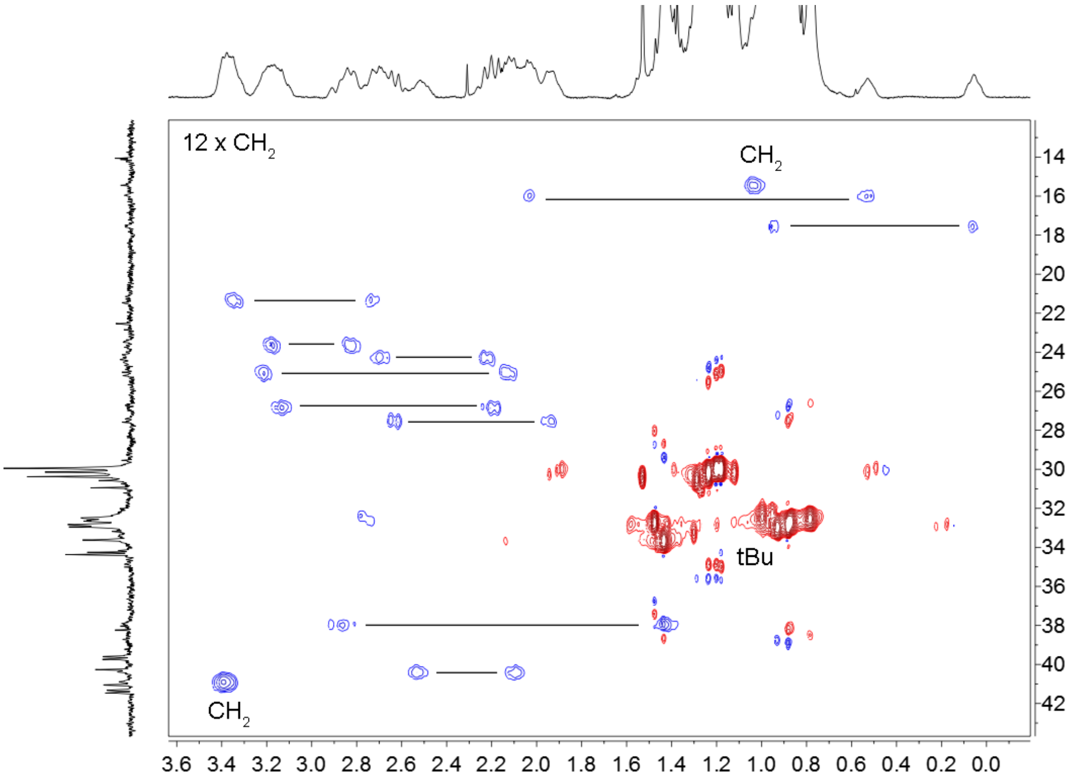

Figure S66. ^{1}**H,**^{13}**C ghsqc** (500/126 MHz, 243 K, dichloromethane-d$_2$) spectrum of *in situ* generated compound **18c**

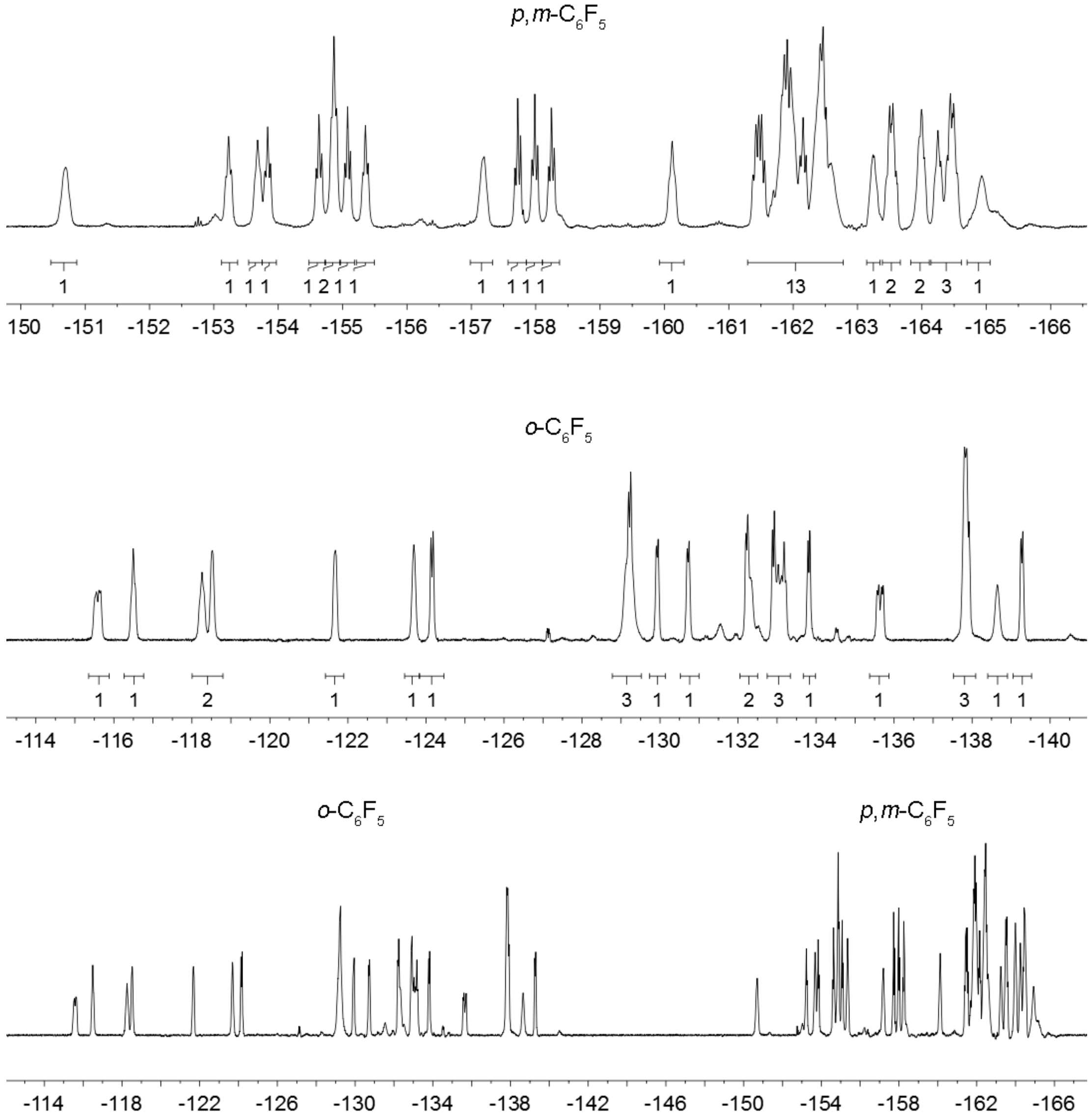

Figure S67. ^{19}**F** (470 MHz, 243 K, dichloromethane-d_2) spectrum of *in situ* generated compound **18c**

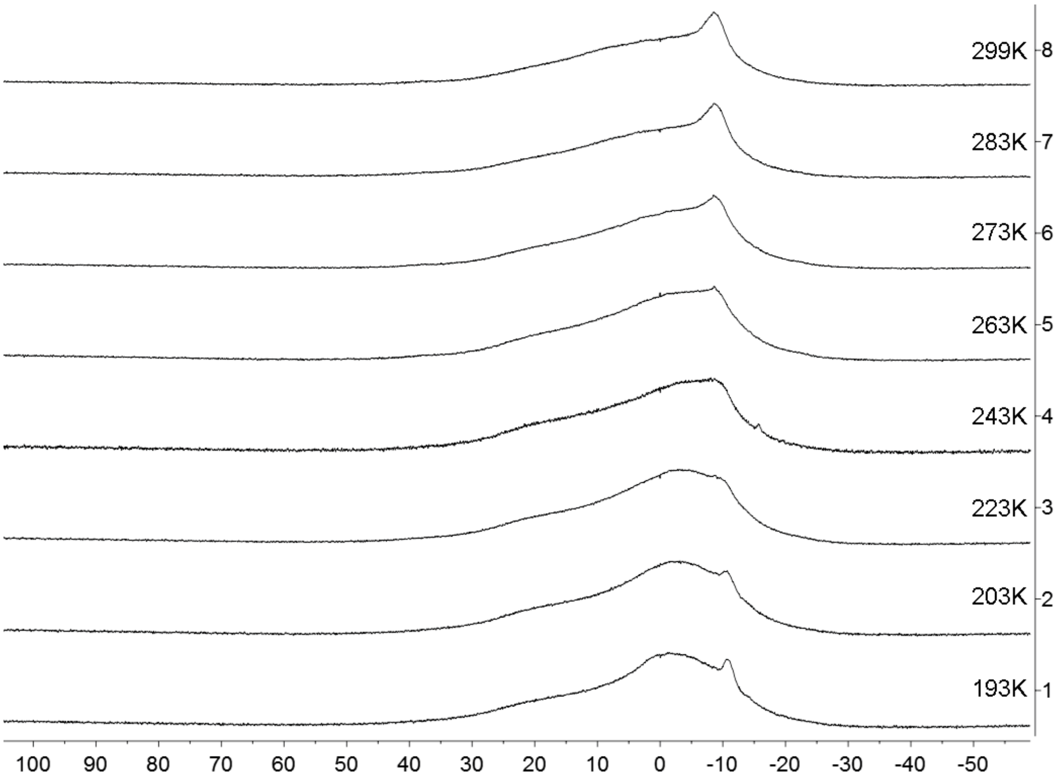

Figure S68. $^{11}B\{^1H\}$ **NMR** (160 MHz, from 193 K to 299 K, dichloromethane-d_2) spectra of *in situ* generated compound **18c**

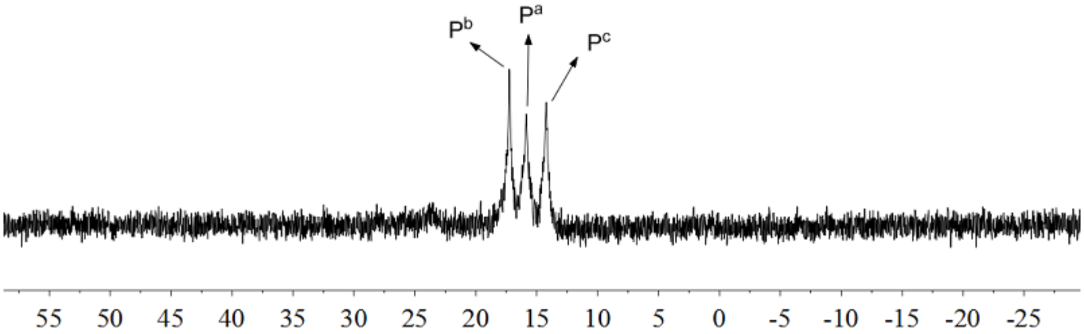

Figure S69. $^{31}P\{^1H\}$ **NMR** (202 MHz, 243K, dichloromethane-d_2) spectrum of *in situ* generated compound **18c**

Crystals suitable for the X-ray crystal structure analysis were obtained by slow diffusion of pentane to a solution of *in situ* generated solution of compound **18c** in toluene under a CO (1.5 bar) atmosphere at -5 °C.

X-ray crystal structure analysis of compound 18c: formula $C_{141}H_{111}B_6F_{60}O_3P_3$, M = 3151.07, colourless crystal, 0.12 x 0.10 x 0.10 mm, a = 12.2916(1), b = 38.2278(4), c = 34.9960(4) Å, β = 90.268(3)°, V = 16443.8(3) Å^3, ρ_{calc} = 1.273 gcm^{-3}, μ = 0.150 mm^{-1}, empirical absorption correction (0.882 ≤ T ≤ 0.985), Z = 4, monoclinic, space group $P2_1/n$ (No. 14), λ = 0.71073 Å, T = 223(2) K, ω and φ scans, 82529 reflections collected (±h, ±k, ±l), 28002 independent (R_{int} = 0.089) and 16175 observed reflections [I>2σ(I)], 1945 refined parameters, R = 0.083, wR^2 = 0.201, max. (min.) residual electron density 0.35 (-0.28) e.Å^{-3}, the hydrogen atoms were calculated and refined as riding atoms.

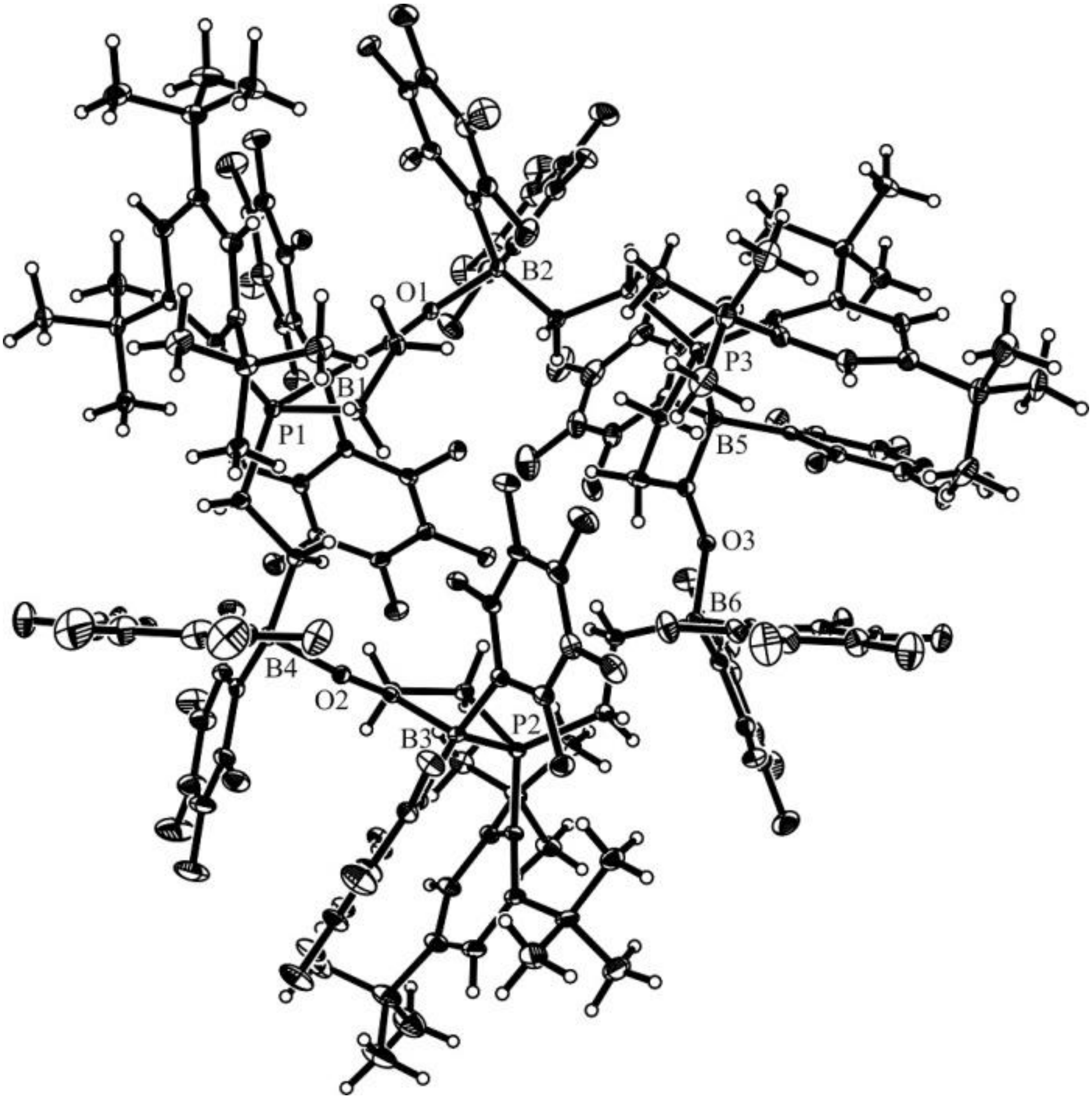

Figure S70. X-ray crystal structure analysis of compound **18c**

In situ generation of ^{13}C labeled compound 18c

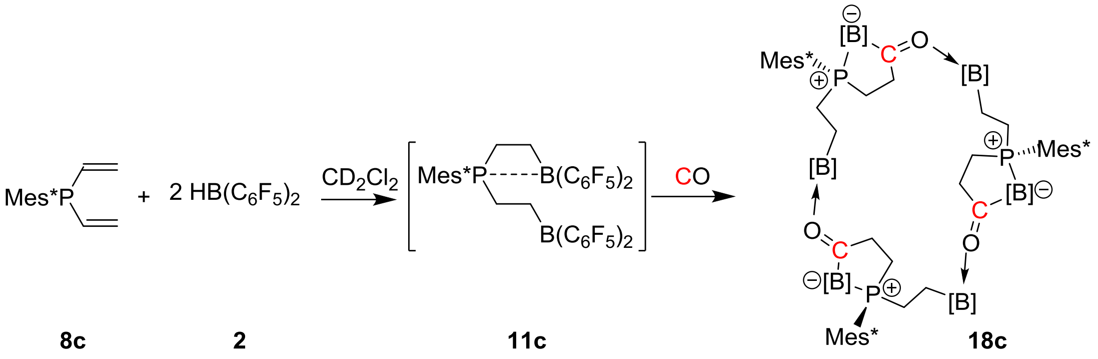

[B]: B(C_6F_5)$_2$ C = ^{13}C

Scheme S14.

In the glove box, compound **8a** (8.3 mg, 0.025 mmol, 1.0 eq.) and compound **2** (17.3 mg, 0.05 mmol, 2.0 eq.) were mixed with dichloromethane-d$_2$ (1 mL) at room temperature to give a yellow solution. The solution was filled in a J-Young tube, which was carefully evacuated at -78 ^{o}C (dry ice / isopropanol bath). Then the solution was exposed to ^{13}CO (1.5 bar) at -78 ^{o}C and characterized by NMR experiments.
^{13}C{^{1}H} NMR (126 MHz, 243 K, dichloromethane-d$_2$)[key resonances] δ = 273.3 (d, $^{2}J_{PC}$ = 23.6 Hz, C=O), 272.0 (d, $^{2}J_{PC}$ = 29.9 Hz, C=O), 271.9 (d, $^{2}J_{PC}$ = 29.9 Hz, C=O).

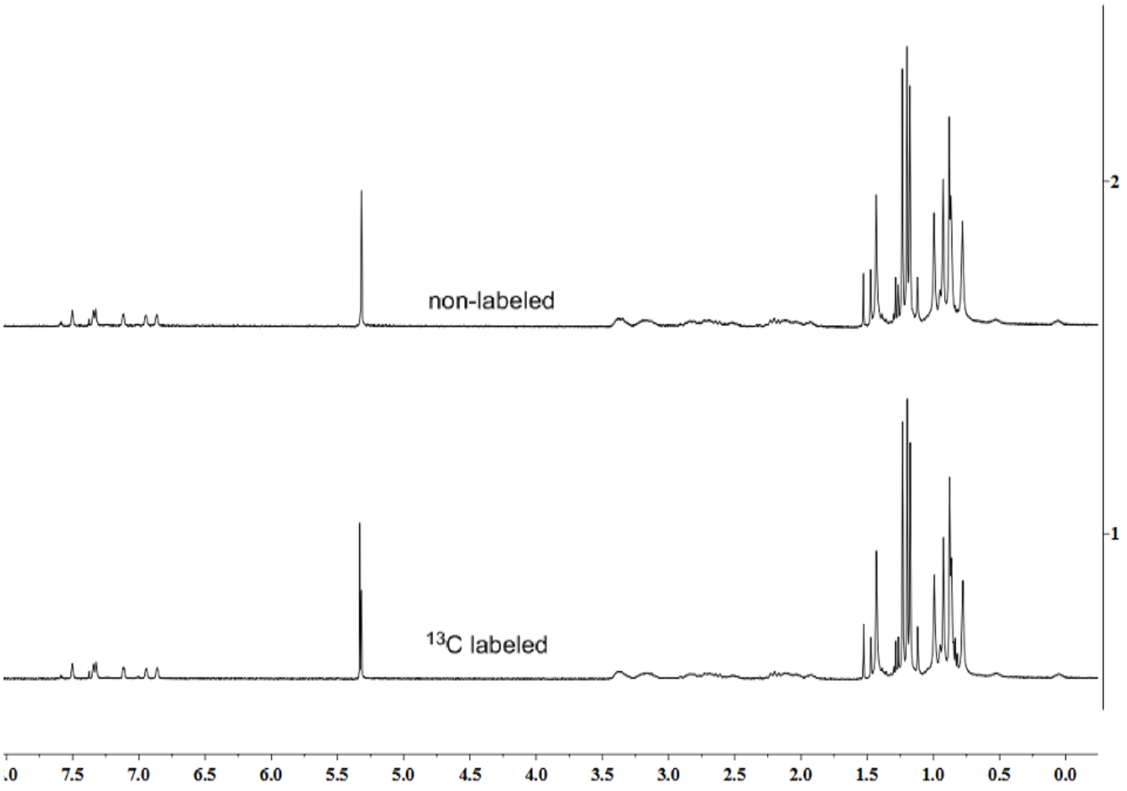

Figure S71. **^{1}H NMR** (500 MHz, 243 K, dichloromethane-d$_2$) spectra of (1) *in situ* generated ^{13}C labeled and (2) non-labeled compound **18c**

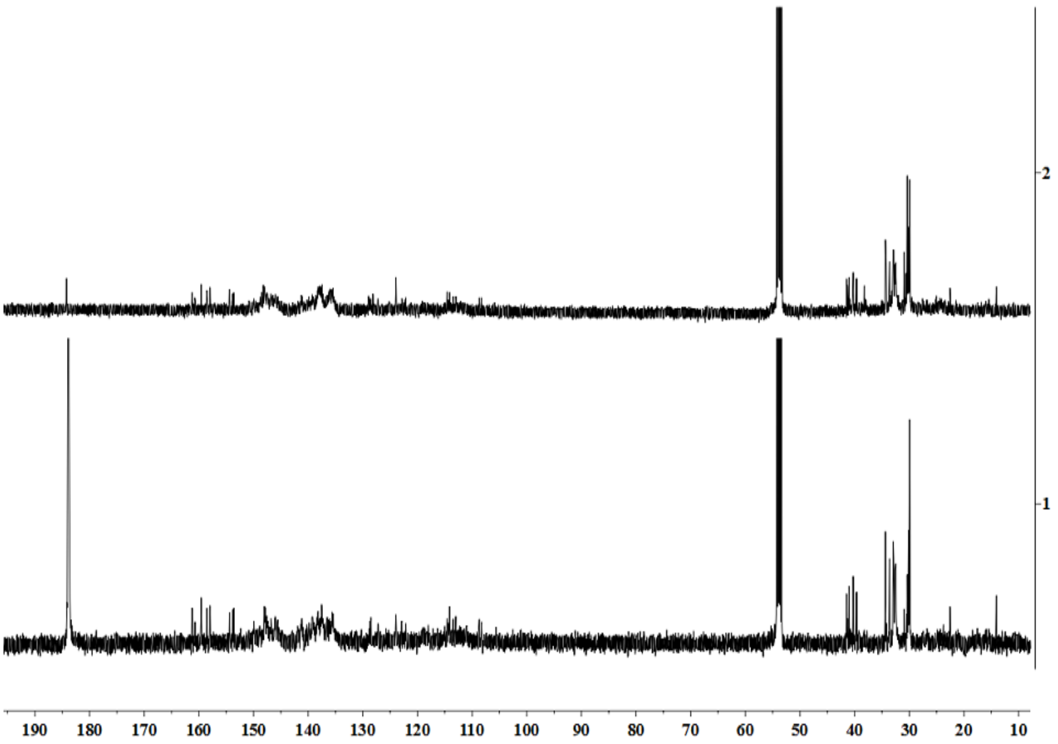

Figure S72. ^{13}**C{**^{1}**H}** (126 MHz, 243 K, dichloromethane-d_2) spectra of *in situ* generated (1) ^{13}C labeled and (2) non-labeled compound **18c**

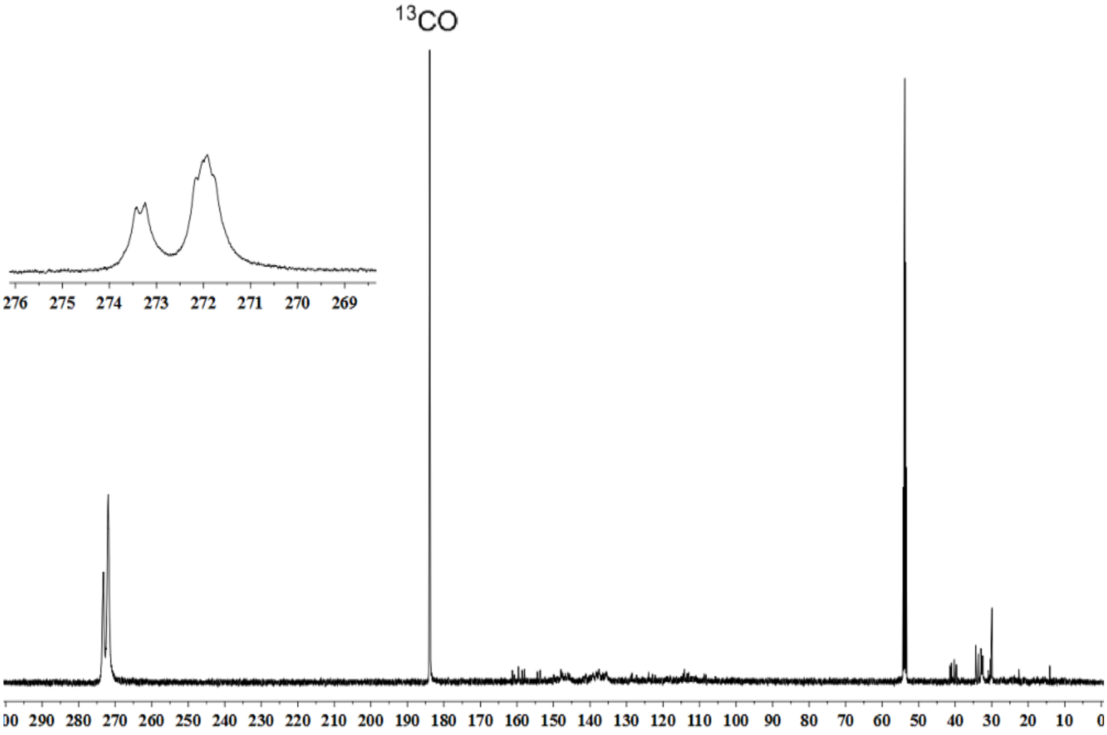

Figure S73. ^{13}**C{**^{1}**H}** (126 MHz, 243 K, dichloromethane-d_2) spectrum of *in situ* generated ^{13}C labeled compound **18c**

Preparation of compound 18c

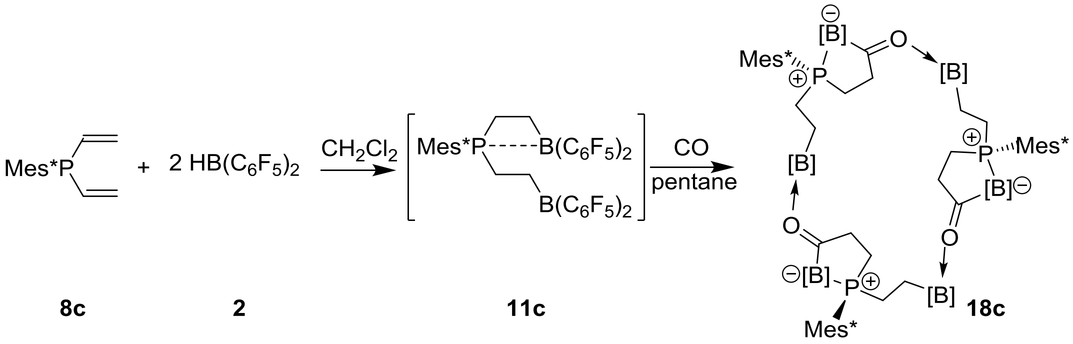

8c **2** **11c** **18c**

[B]: $B(C_6F_5)_2$

Scheme S15.

In a Schlenk flask, compound **8c** (66.1 mg, 0.20 mmol, 1.0 eq.) and compound **2** (138.4 mg, 0.40 mmol, 2.0 eq.) were mixed with dichloromethane (4 mL) at room temperature. Then all volatiles were removed in vacuo and the obtained residue was dissolved in pentane (4 mL). After the solution was cooled to -78 °C (dry ice / isopropanol bath), the flask was evacuated carefully. Then the dry ice bath was removed and the reaction mixture was exposed to CO gas (1.5 bar). The reaction mixture was stirred at room temperature for 30 min to give a white suspension. After filtration by using a filter cannula, the collected white solid was washed with pentane (1 mL × 2) and dried in vacuo to give compound **18c** as a white solid (170.2 mg, 0.054 mmol, yield 81 %).

IR(KBr): $\tilde{\upsilon}$ [cm^{-1}] = 1646 (m, C=C), 1592 (m, C=O), 1521 (s, C=C), 1468 (s, C=C), 1290 (m), 1097 (s), 979 (s, =C-H).

Elemental analysis (%) calcd. for $C_{141}H_{111}B_6F_{60}O_3P_3$: C 53.74, H 3.55; Found: C 52.53, H 3.17.

Melting point: 140 °C

The NMR data from a solution of the white solid in dichloromethane-d_2 were consistent to those obtained for compound **18c**

^{31}P NMR (202 MHz, 203 K, dichloromethane-d_2) δ = 17.9 (m, 1P), 15.8 (m, 1P), 14.0 (m, 1P).

^{31}P{^{1}H} NMR (202 MHz, 203 K, dichloromethane-d_2) δ = 17.9 (m, 1P), 15.8 (m, 1P), 14.0 (m, 1P)

^{31}P{^{1}H,^{11}B} NMR (243 MHz, 203 K, dichloromethane-d_2) δ = 17.9 (s, 1P), 15.8 (s, 1P), 14.0 (s, 1P).

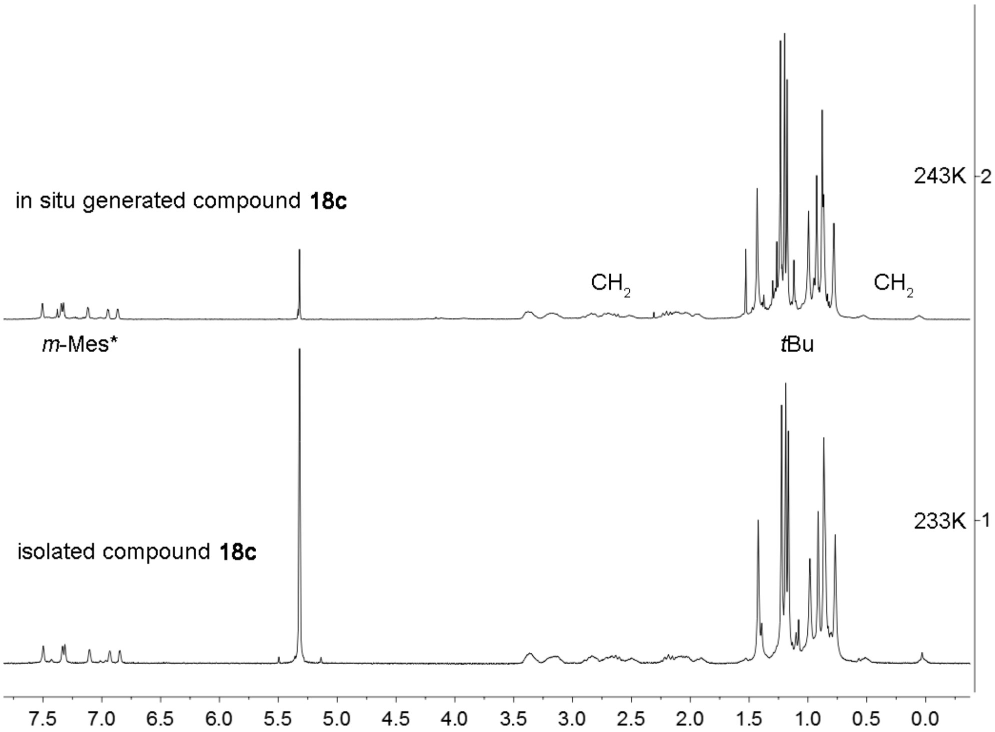

Figure S74. **^{1}H NMR** (500 MHz, dichloromethane-d_2) spectra of (1) isolated (233K) and (2) *in situ* generated (243K) compound **18c**

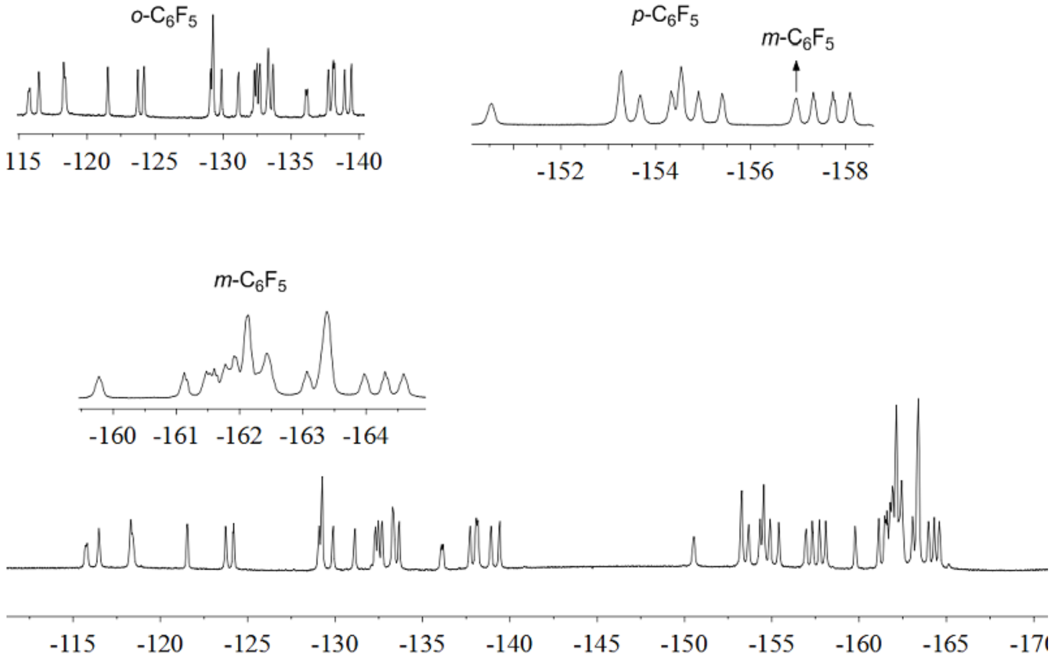

Figure S75. ^{19}**F NMR** (470 MHz, 203 K, dichloromethane-d_2) spectrum of compound **18c**

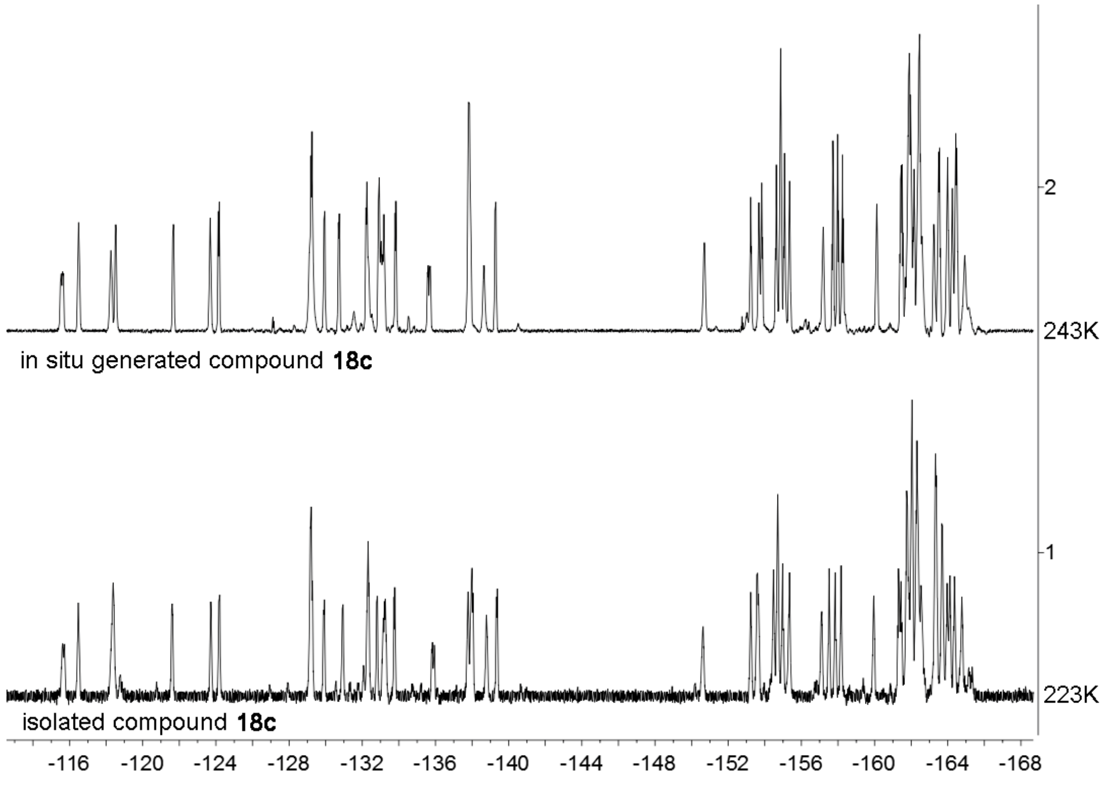

Figure S76. ^{19}**F NMR** (470 MHz, dichloromethane-d_2) spectra of (1) isolated (223K) and (2) *in situ* generated (243K) compound **18c**

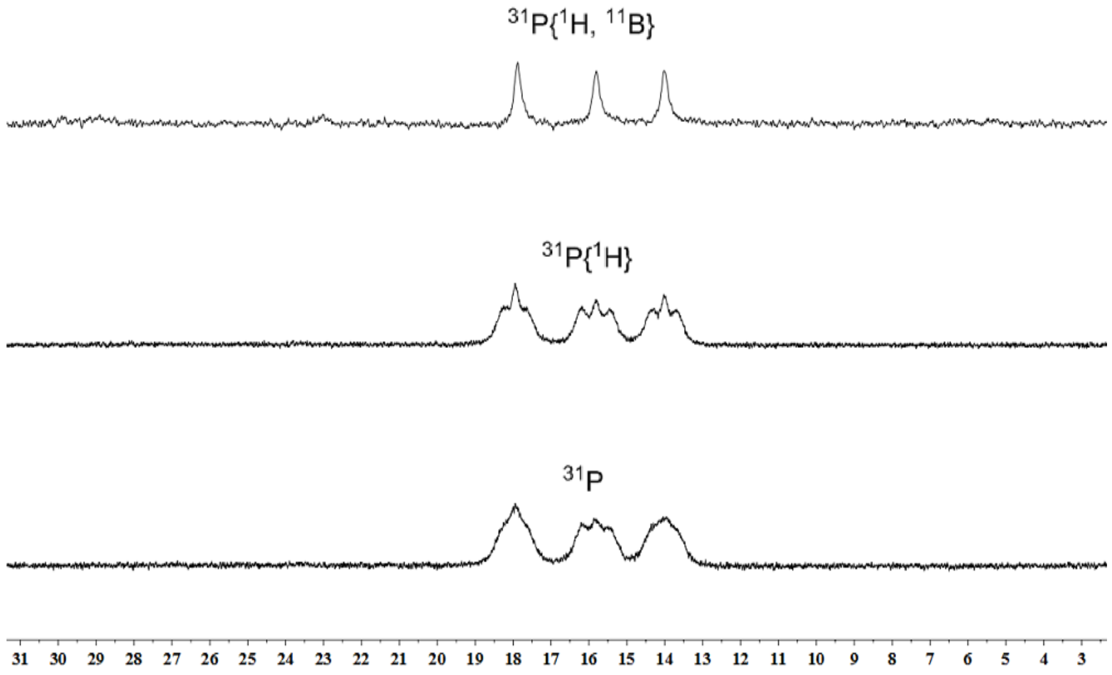

Figure S77. ^{31}**P** (bottom), ^{31}**P{^{1}H}** (middle) **NMR** (202 MHz, 203K, dichloromethane-d_2), and ^{31}**P{^{1}H,^{11}B}** (top) **NMR** (243 MHz, 203K, dichloromethane-d_2) spectra of compound **18c**

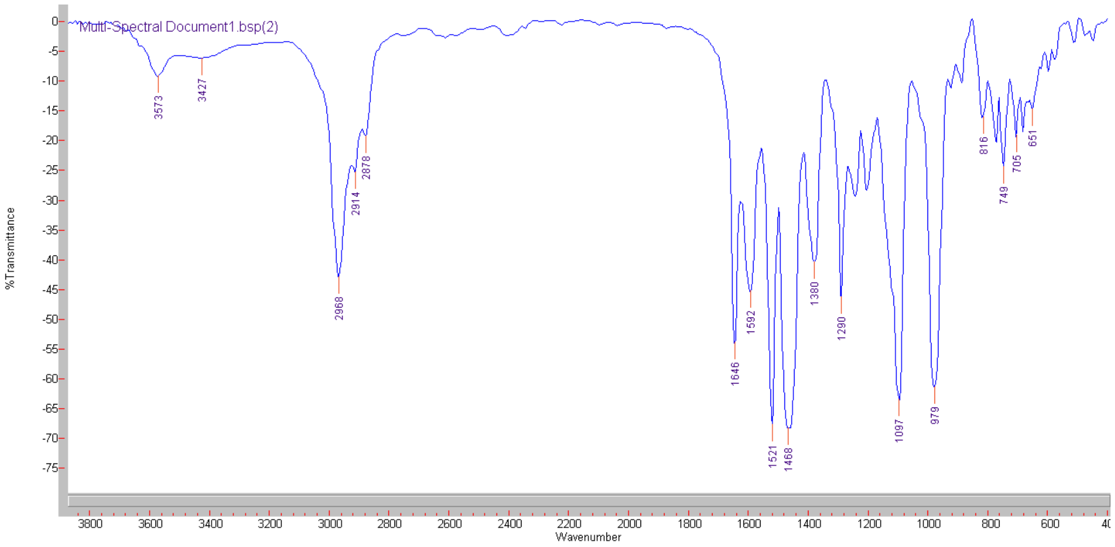

Figure S78. IR(KBr) of compound **18c**

Stability of compound 18c in dichloromethane-d_2 solution

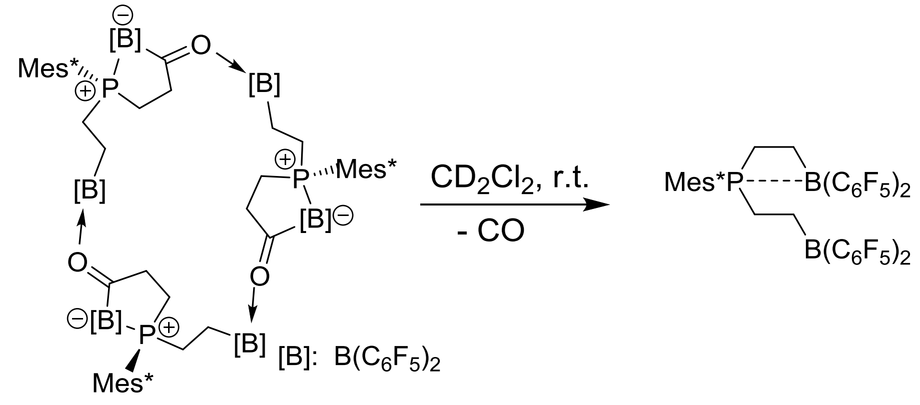

18c **11c**

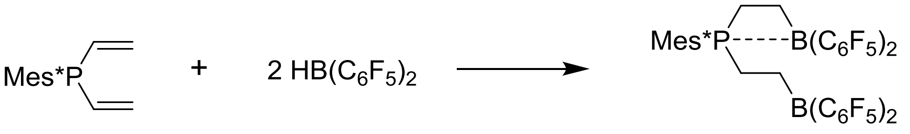

8c **2** **11c**

Scheme S16.

Compound **18c** (white solid (see page 35), 15.8 mg, 0.005 mmol) was dissolved in dichloromethane-d_2 (1 mL) at room temperature and the obtained solution was characterized by NMR experiments, immediately (after ca. about 10 minutes).

Control experiment: In a Schlenk flask, a solution of compound **8c** (16.5 mg, 0.05 mmol, 1.0 eq.) and compound **2** (34.6 mg, 0.10 mmol, 2.0 eq.) in dichloromethane-d_2 (1 mL) was stirred for 15 min at room temperature to give a yellow solution of compound **11c**.

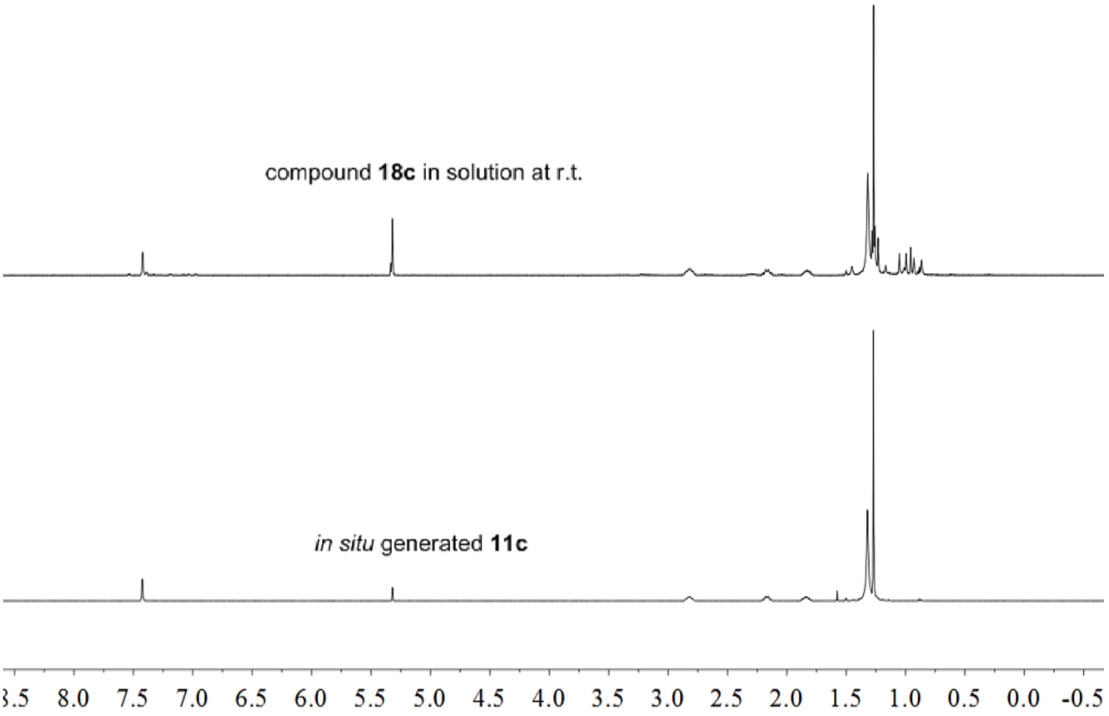

Figure S79. **^{1}H NMR** (500 MHz, 299 K, dichloromethane-d_2) spectra of compound **18c** in solution at room temperture (top) and *in situ* generated compound **11c** (bottom)

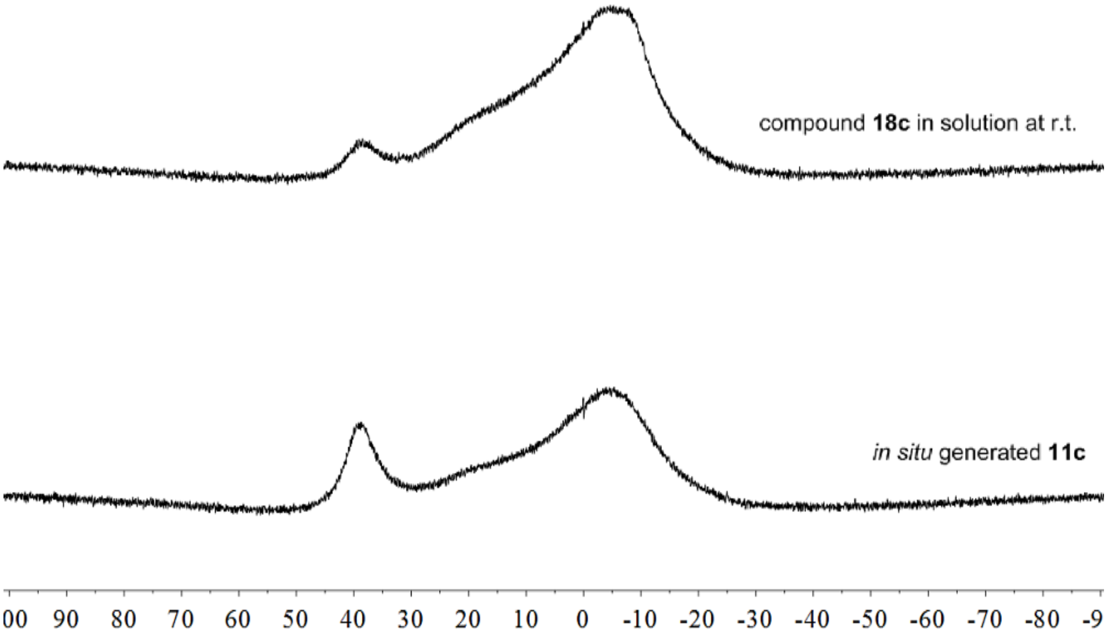

Figure S80. **$^{11}B\{^1H\}$ NMR** (160 MHz, 299K, dichloromethane-d$_2$) spectra of compound **18c** in solution at room temperture (top) and *in situ* generated compound **11c** (bottom)

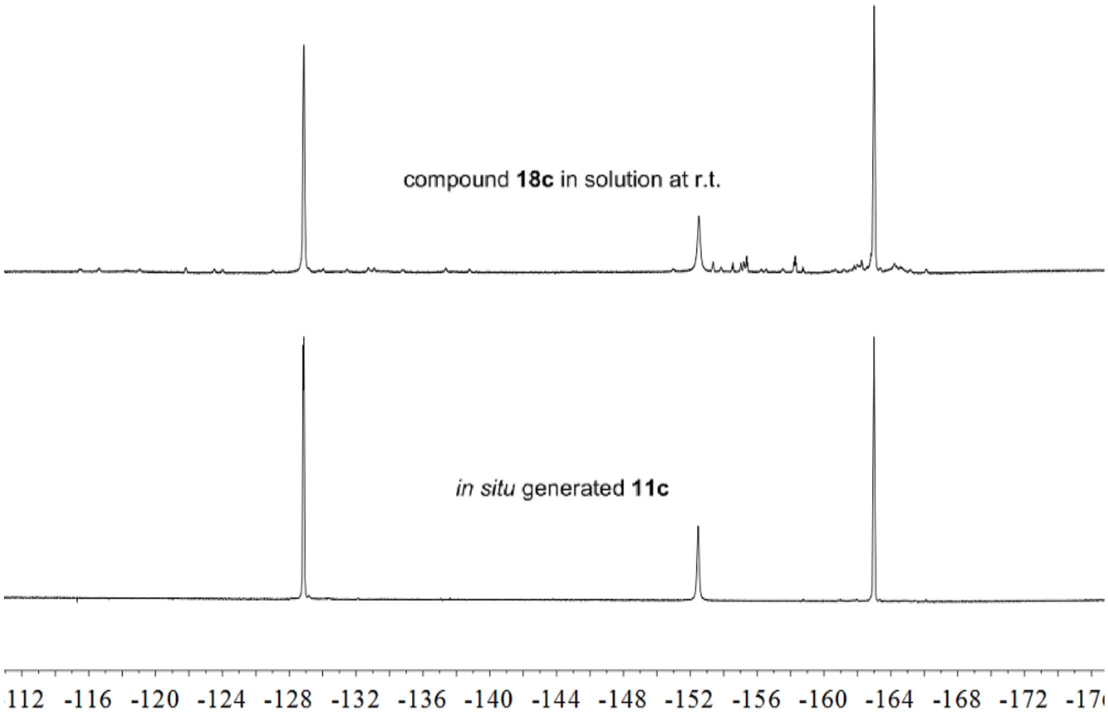

Figure S81. **^{19}F NMR** (470 MHz, 299 K, dichloromethane-d$_2$) spectra of compound **18c** in solution at room temperture (top) and *in situ* generated compound **11c** (bottom)

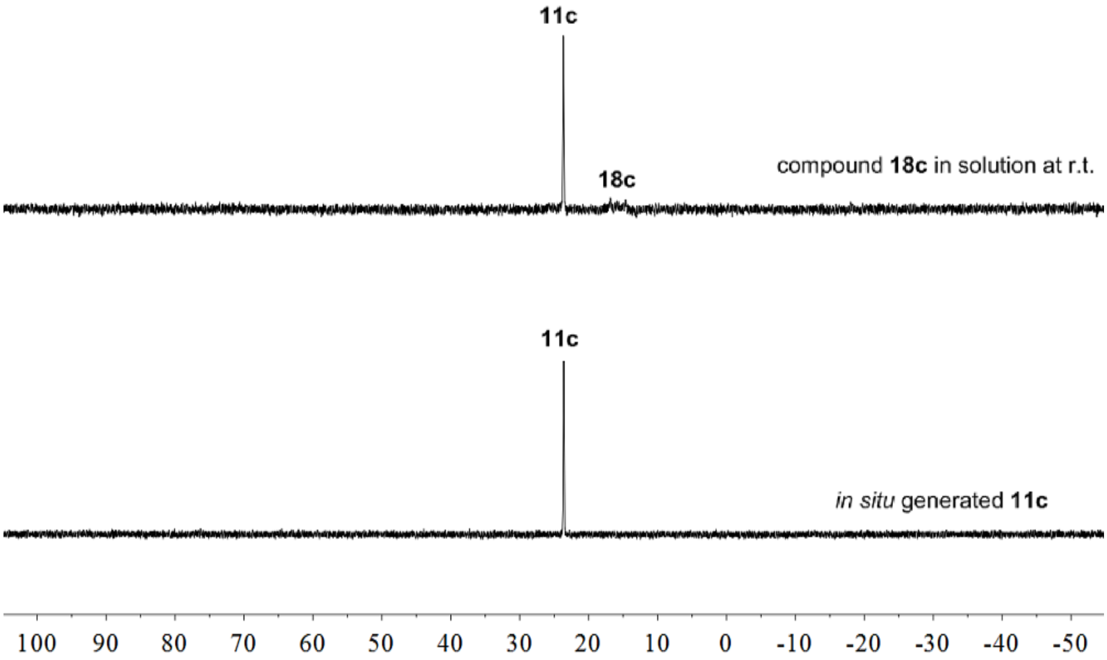

Figure S82. $^{31}P\{^1H\}$ **NMR** (299 MHz, 243K, dichloromethane-d_2) spectra of compound **18c** in solution at room temperture (top) and *in situ* generated compound **11c** (bottom)

Reaction of compound 18c with pyridine

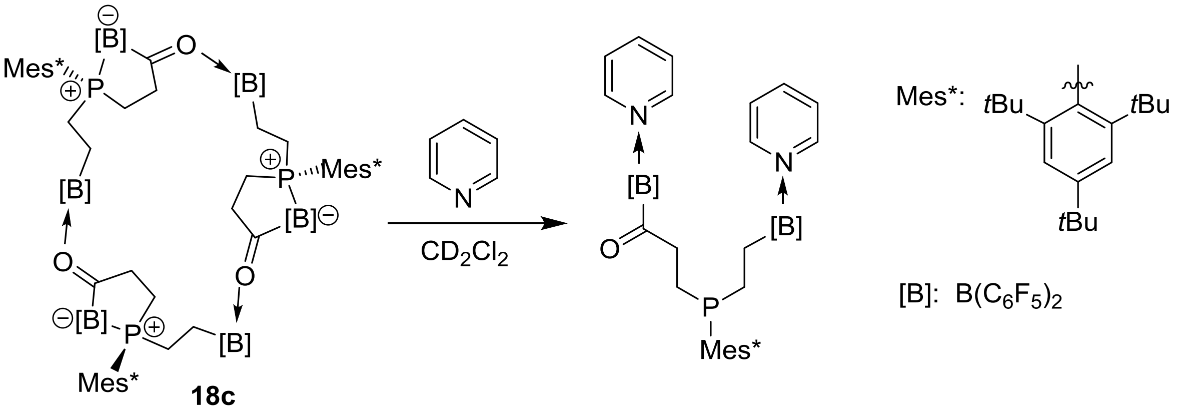

Scheme S17.

Compound **18c** (white solid, 15.8 mg, 0.005 mmol) was mixed with a solution of pyridine (2.5 mg, 0.032) in dichloromethane-d_2 (1 mL) at -30 °C. Then the *in situ* generated solution was characterized by NMR experiments.

^{1}H NMR (500 MHz, 299 K, dichloromethane-d_2) δ = 8.60, 8.55 (each m, each 2H, *o*-py), 8.11 (m, 2H, *p*-py), 7.64 (m, 4H, *m*-py), 7.24 (d, $^4J_{PH}$ = 1.5 Hz, 2H, *m*-Mes*), 2.19/1.92 (each m, each 1H, CH_2^{CO}), 1.94 (m, 2H, PCH_2^{CO}), 1.66/1.42 (each m, each 1H, PCH_2), 1.35 (s, 18H, *o*-^{t}Bu^{Mes*}), 1.29/1.11 (each m, each 1H, BCH_2), 1.17 (s, 9H, *p*-^{t}Bu^{Mes*}).

^{13}C{^{1}H} NMR (126 MHz, 299 K, dichloromethane-d_2) δ = 238.6 (br, C=O), 158.7 (d, $^2J_{PC}$ = 11.6 Hz, *o*-Mes*), 149.4 (d, $^4J_{PC}$ = 2.1 Hz, *p*-Mes*), 147.2, 146.3 (*o*-Py), 142.3, 142.1 (*p*-Py), 135.6 (d, $^1J_{PC}$ = 47.5 Hz, *i*-Mes*), 126.4, 125.8 (*m*-Py), 122.2 (d, $^3J_{PC}$ = 6.5 Hz, *m*-Mes*), 43.1 (d, $^2J_{PC}$ = 25.8 Hz, CH_2^{CO}), 39.0 (d, $^3J_{PC}$ = 4.2 Hz, *o*-^{t}Bu^{Mes*}), 34.8 (*p*-^{t}Bu^{Mes*}), 34.0 (d, $^4J_{PC}$ = 8.0 Hz, *o*-^{t}Bu^{Mes*}), 31.0 (*p*-^{t}Bu^{Mes*}), 26.0 (d, $^1J_{PC}$ = 23.5 Hz, PCH_2), 22.8 (d, $^1J_{PC}$ = 22.2 Hz, PCH_2^{CO}), 21.8 (br, BCH_2), [C_6F_5 not listed].

^{11}B{^{1}H} NMR (160 MHz, 299 K, dichloromethane-d_2) δ = -0.7 ($\nu_{1/2}$ ~ 400 Hz), -5.8 ($\nu_{1/2}$ ~ 200 Hz).

^{19}F NMR (470 MHz, 299 K, dichloromethane-d_2) δ = -130.6 (2F), -130.9 (2F), -132.2 (4F)(each m, *o*-C_6F_5), -156.6 (t, $^3J_{FF}$ = 20.1 Hz), -156.7 (t, $^3J_{FF}$ = 20.3 Hz), -159.3 (t, $^3J_{FF}$ = 20.2 Hz), -159.4 (t, $^3J_{FF}$ = 20.2 Hz)(each 1F, *p*-C_6F_5), -163.3, -163.4, -164.67, -164.73 (each m, each 2F, *m*-C_6F_5).

^{31}P{^{1}H} NMR (202 MHz, 299 K, dichloromethane-d_2) δ = -16.7 ($\nu_{1/2}$ ~ 6 Hz)

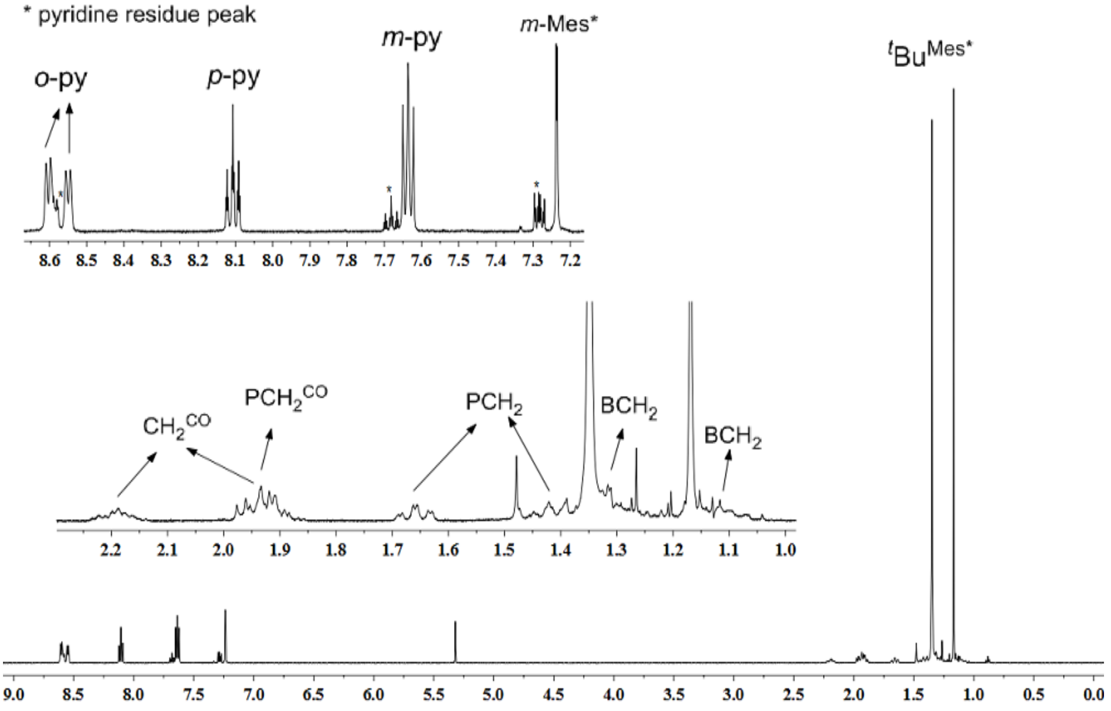

Figure S83. **^{1}H NMR** (500 MHz, 299 K, dichloromethane-d_2) spectrum of the solution

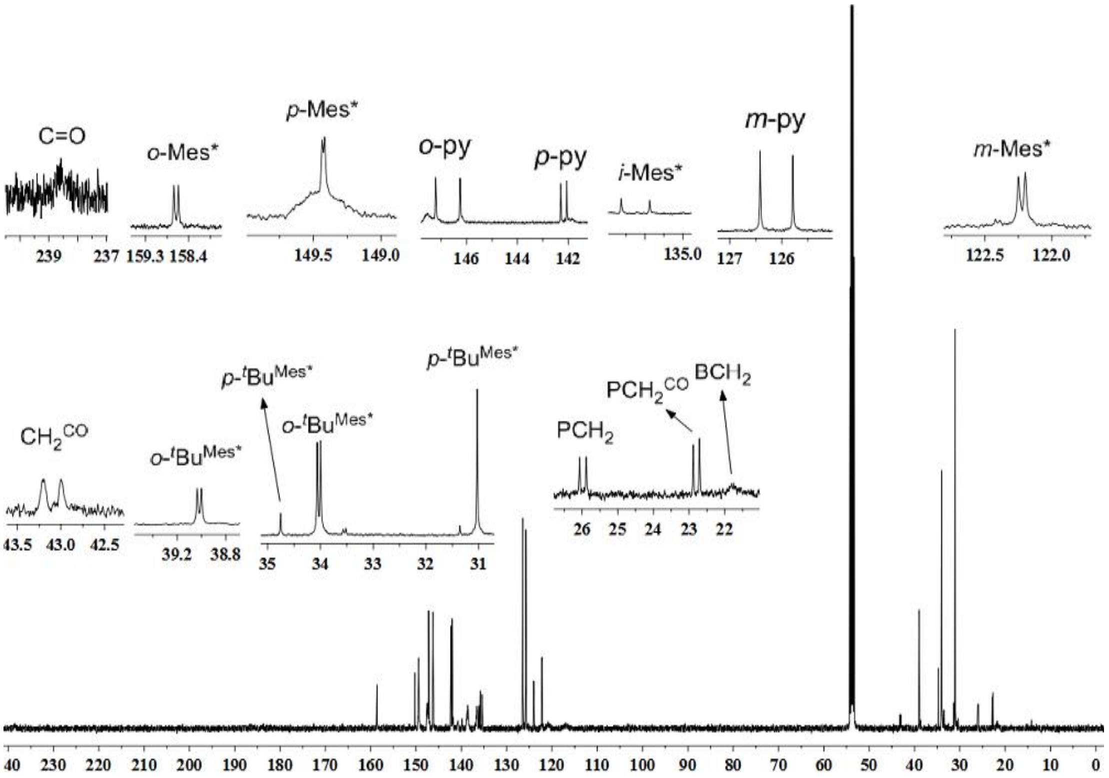

Figure S84. ^{13}**C NMR** (500 MHz, 299 K, dichloromethane-d$_2$) spectrum of the solution

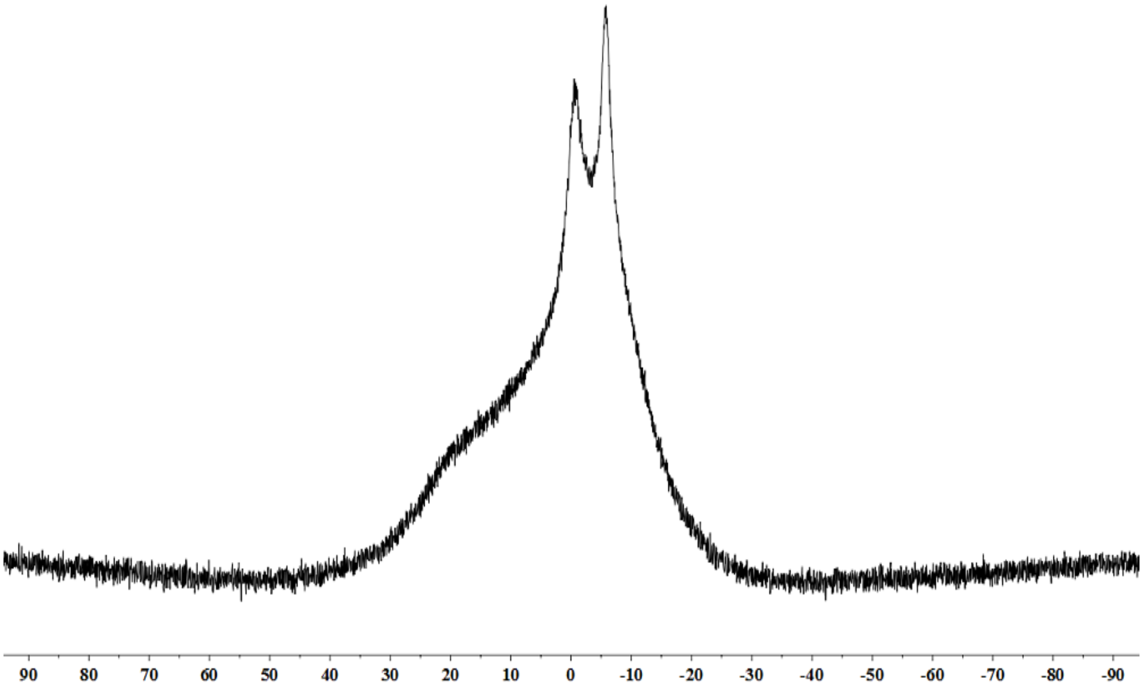

Figure S85. ^{11}**B NMR{^{1}H}** (500 MHz, 299 K, dichloromethane-d$_2$) spectrum of the solution

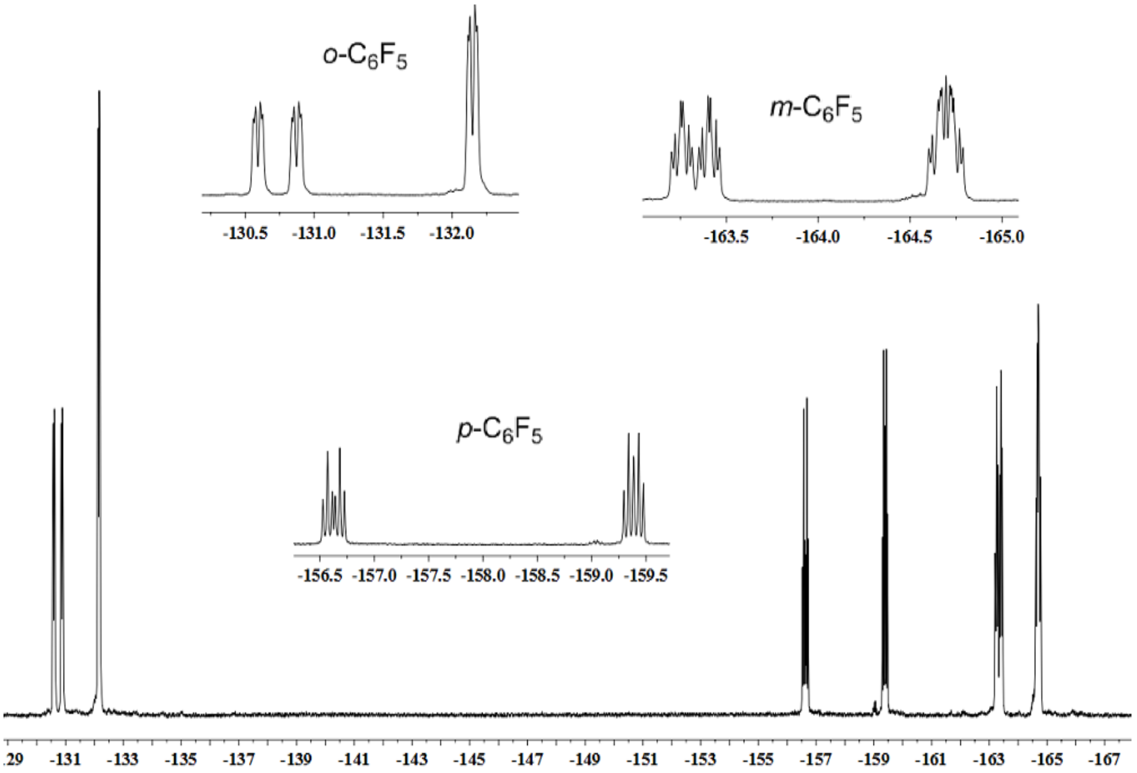

Figure S86. **^{19}F NMR** (500 MHz, 299 K, dichloromethane-d$_2$) spectrum of the solution

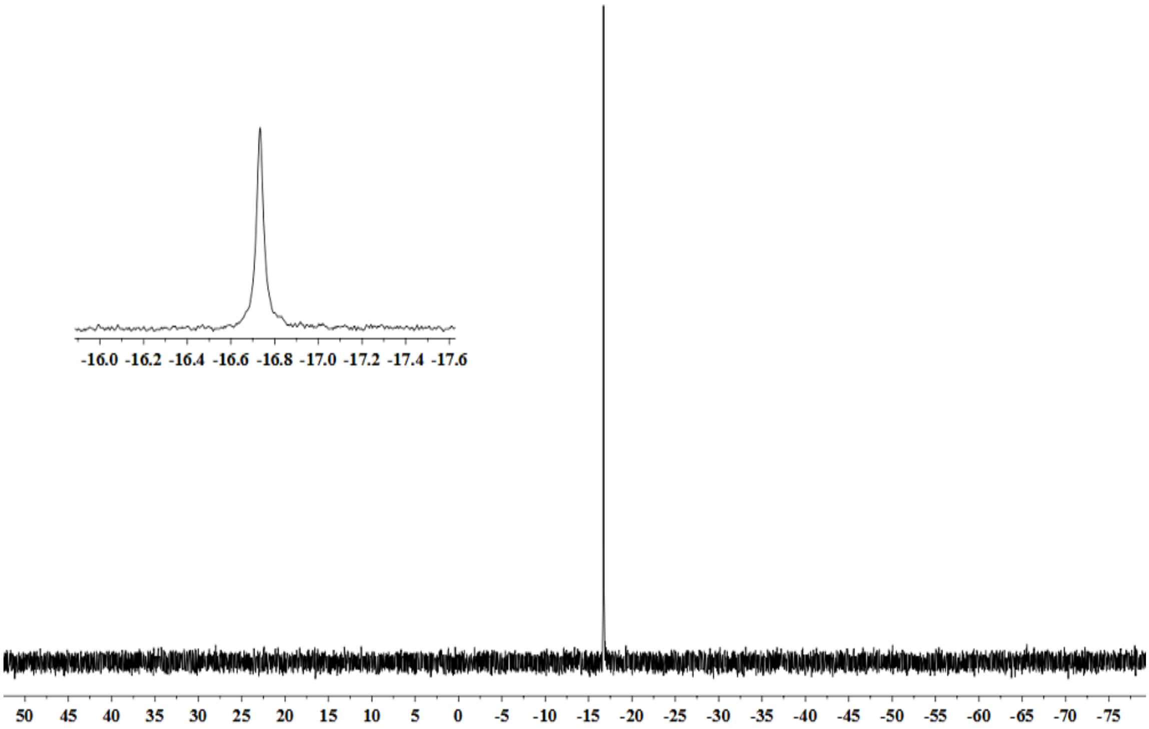

Figure S87. **^{19}F NMR** (500 MHz, 299 K, dichloromethane-d$_2$) spectrum of the solution

Stability of compound 17a in dichloromethane-d$_2$ solution at 50 °C

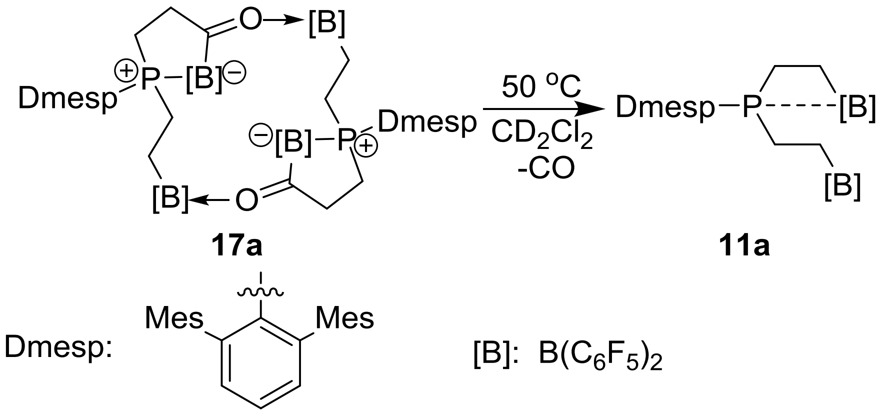

Scheme S18.

Compound **17a** (white solid, 15 mg) was dissolved in dichloromethane-d$_2$ (1 mL) at room temperature and the obtained solution was flame-sealed in a NMR tube under vacuum. The solution was heated at 50 °C for 12h to give a pale yellow solution which were characterized by NMR experiments.
Control experiment: (1) compound **17a** (white solid, 15 mg) in dichloromethane-d$_2$; (2) see Scheme S4 (compound **11a**) in dichloromethane-d$_2$.

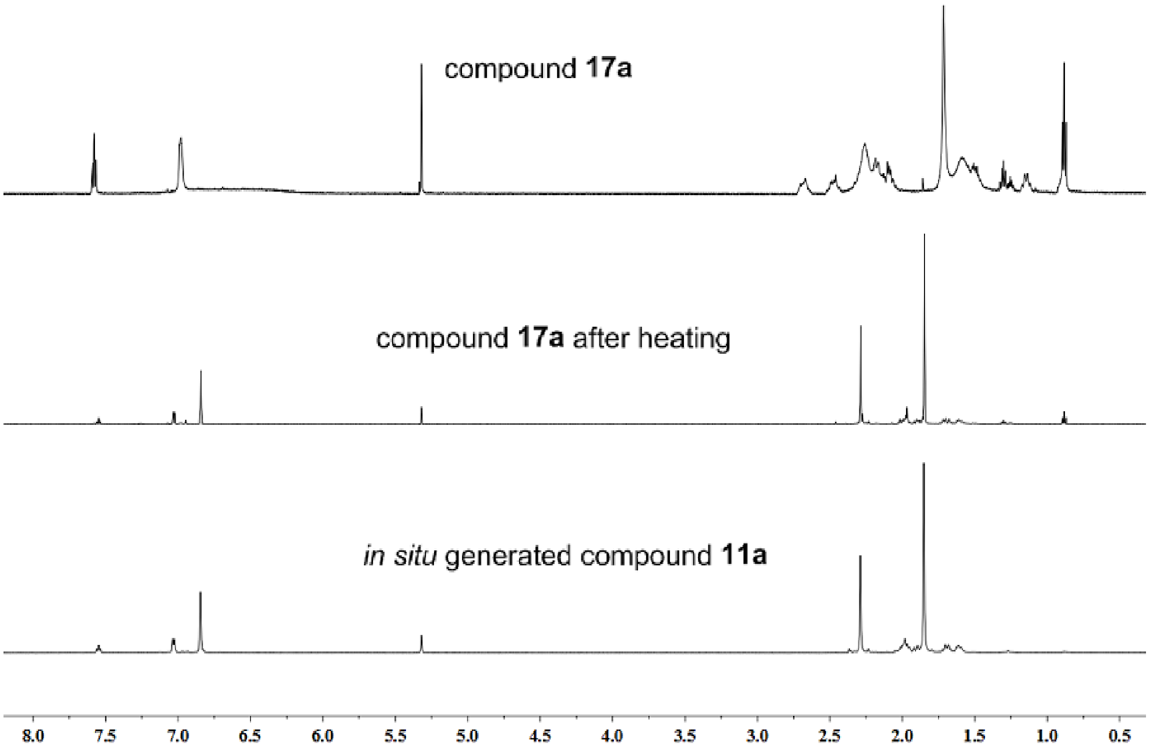

Figure S88. **^{1}H NMR** (600 MHz, 299 K, dichloromethane-d$_2$) spectra of compound **17a** (top) compound **17a** after heating (middle) and *in situ* generated compound **11a** (bottom)

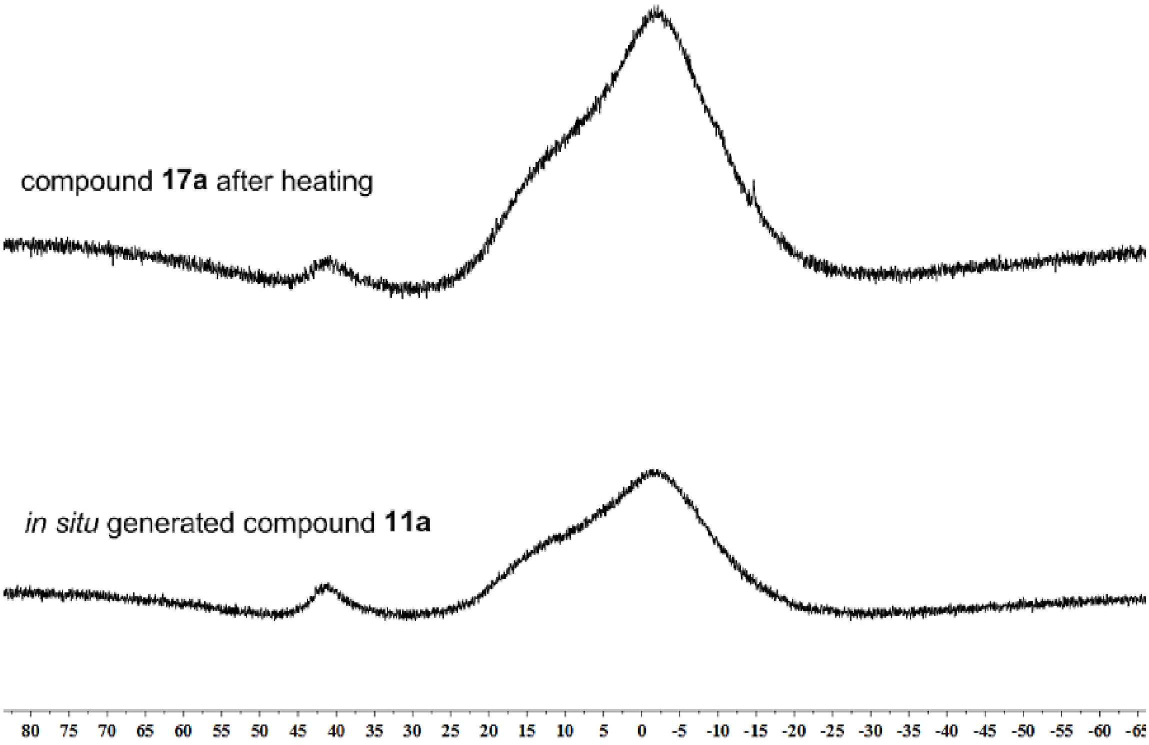

Figure S89. $^{11}B\{^{1}H\}$ **NMR** (192 MHz, 299 K, dichloromethane-d_2) spectra of compound **17a** after heating (top) and *in situ* generated compound **11a** (bottom)

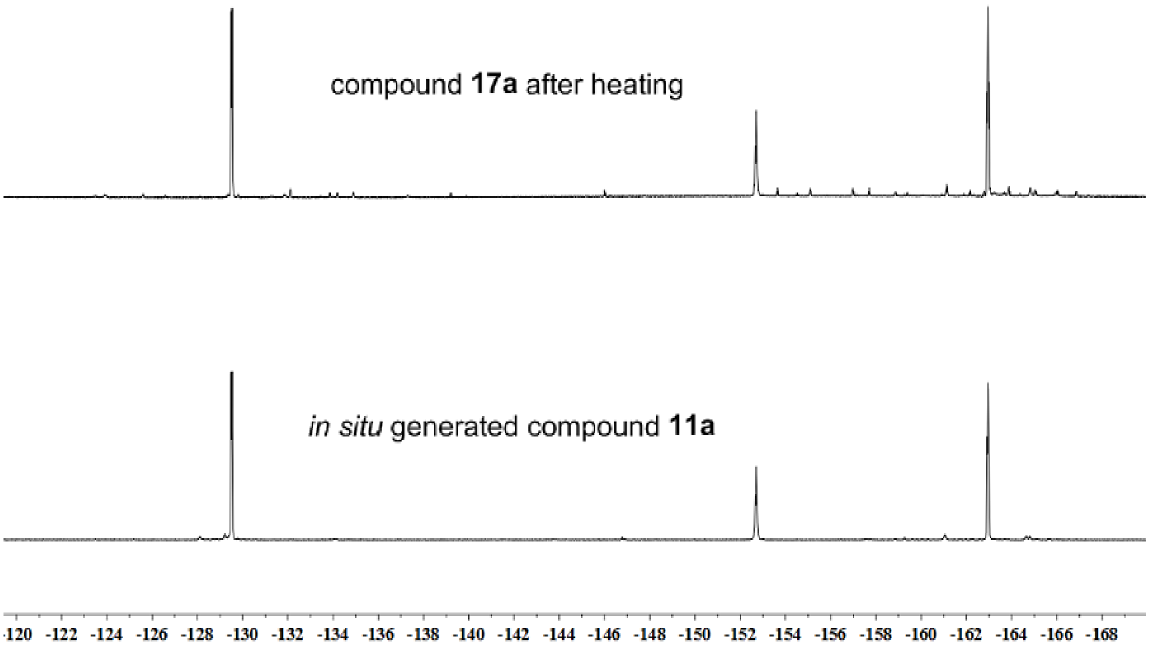

Figure S90. ^{19}F **NMR** (564 MHz, 299 K, dichloromethane-d_2) spectra of compound **17a** after heating (top) and *in situ* generated compound **11a** (bottom)

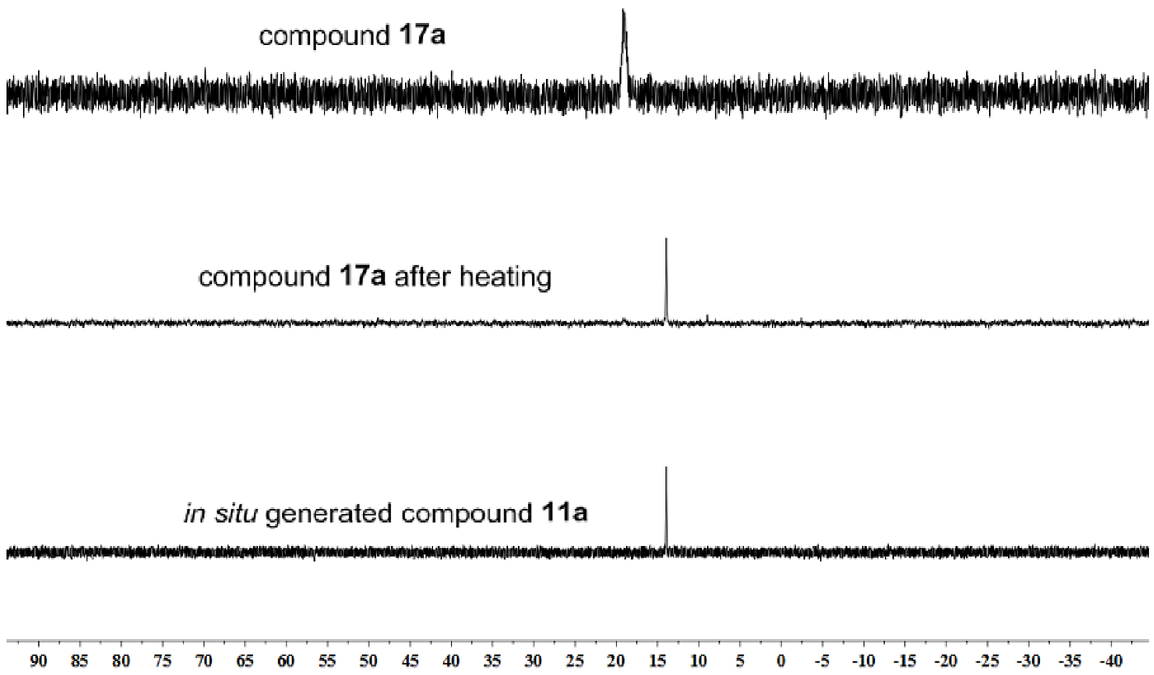

Figure S91. **$^{31}P\{^{1}H\}$ NMR** (243 MHz, 299 K, dichloromethane-d_2) spectra of compound **17a** (top), compound **17a** after heating (middle) and *in situ* generated compound **11a** (bottom)

Stability of compound 17b in benzene-d$_6$ at 80 °C

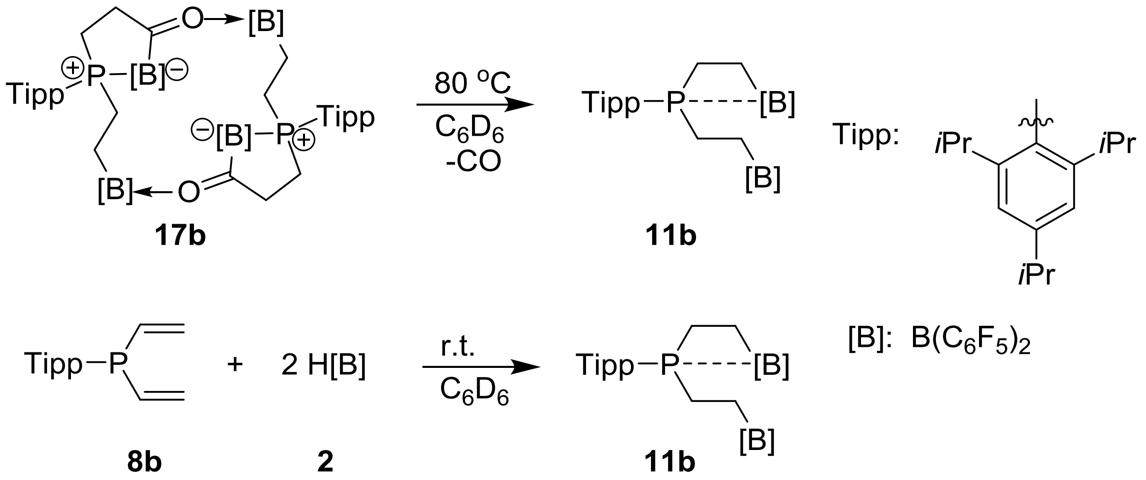

Scheme S19.

Compound **17b** (white solid, 15 mg) was mixed with benzene-d$_6$ (1 mL) in a J-Young tube at room temperature (suspension). The suspension in J-Young tube was heated at 80 °C for 6h to give a colorless solution which were characterized by NMR experiments.
Control experiment: (1) Compound **17b** (white solid, 15 mg) in dichloromethane-d$_2$ (the solubility of compound **17b** in benzene-d$_6$ is too low for the measurements). (2) In a Schlenk flask, a solution of compound **8b** (14.4 mg, 0.05 mmol, 1.0 eq.) and compound **2** (35.5 mg, 0.1026 mmol, 2.05 eq.) in benzene-d$_6$ (1 mL) were stirred for 12 h at room temperature. After filtration, a colorless solution of compound **11b** was obtained and analyzed by NMR experiments.

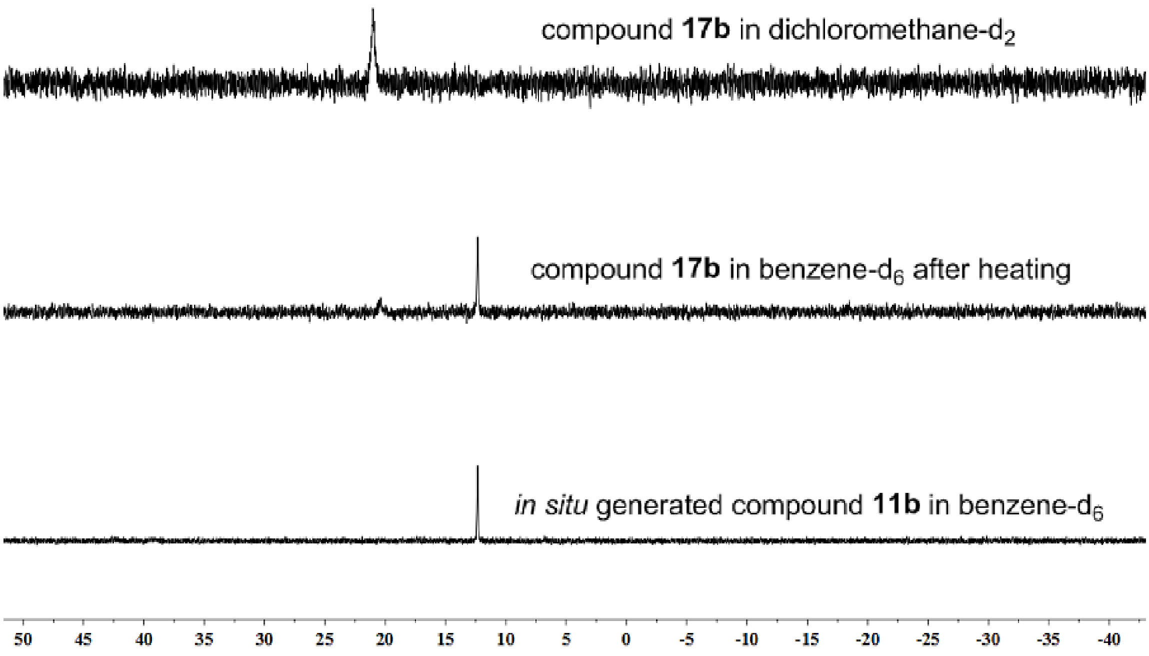

Figure S92. $^{31}P\{^1H\}$ **NMR** (243 MHz, 299 K) spectra of compound **17b** in dichloromethane-d$_2$ (top), compound **17b** in benzene-d$_6$ after heating (middle) and *in situ* generated compound **11b** in benzene-d$_6$ (bottom)

Part 2. Computational Details

Formation of Macrocyclic Ring Systems by Carbonylation of Trifunctional P/B/B Frustrated Lewis Pairs

Lei Liu,[b] Stefan Grimme,[b]

[b] Mulliken Center for Theoretical Chemistry, Institut für Physikalische und Theoretische Chemie, Universität Bonn, Germany

All density functional theory (DFT) calculations were performed by employing Turbomole 7.0[12] software. The structures were optimized at the composite PBEh-3c DFT level of theory.[13] Harmonic vibrational frequency calculations were conducted at the same level to characterize the nature of the stationary points along the reaction coordinates: no imaginary frequencies were found for the local minima, and one and only one imaginary frequency for the transition states. The thermostatistical contributions to the free energy in the gas phase were obtained from a modified rigid-rotor-harmonic oscillator approximation[14] at temperatures of 298.15 and 373.15 K, respectively, and for 1 atm pressure. The density-fitting RI-J[15,16] approach for the Coulomb integrals was used to accelerate the geometry optimization and frequencies calculations. Accurate electronic energies were obtained from single point calculations at the PW6B95 level[17] upon the optimized structures, with the BJ-damped variant of the D3 dispersion correction[18,19] in conjunction with the def2-TZVP basis set.[20,21] The COSMO-RS (Conductor-like Screening Model for Real Solvents) solvation model[22,23] was used to compute the solvation Gibbs free energies by employing the gas–phase optimized structures, and with toluene as the solvent. These calculations were done with the COSMOtherm program.[24] The final Gibbs free energies in solution were calculated from the gas-phase single point electronic energies plus the gas-phase thermal contributions, and the COSMO-RS solvation Gibbs free energies.

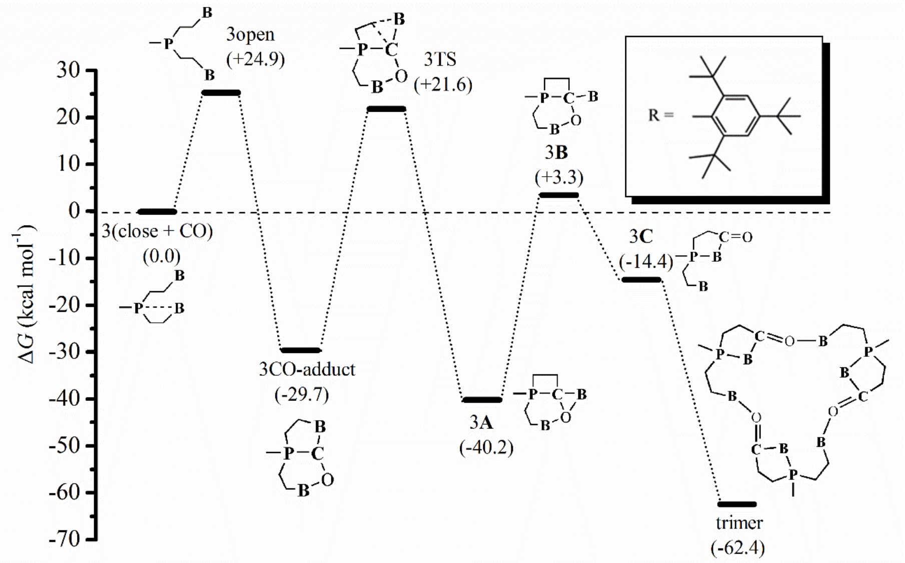

Figure S93. Computed reaction Gibbs free energy path at PW6B95-D3/def2-TZVP (COSMO-RS, toluene)//HF-3c level of the theory. The energy of 3 mol (close + CO) is selected as the reference..

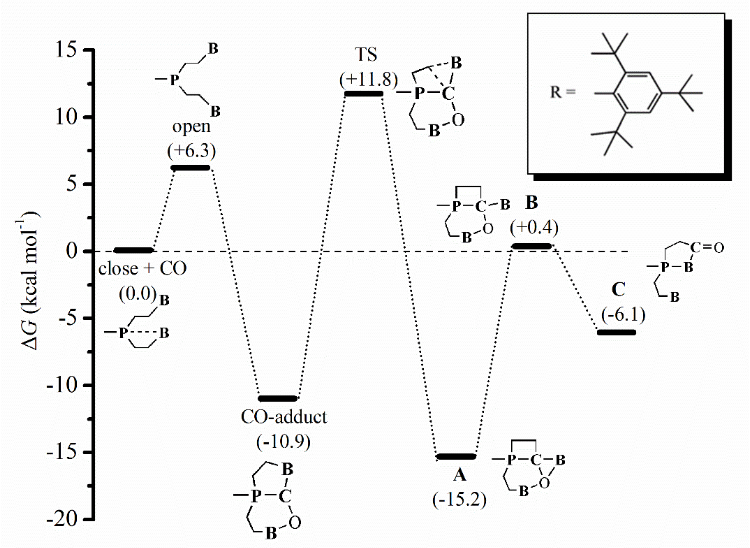

Figure S94. Computed reaction Gibbs free energy path at PW6B95-D3/def2-TZVP (COSMO-RS, toluene)//PBEh-3c level of the theory. The energy of 1 mol (close + CO) is selected as the reference.

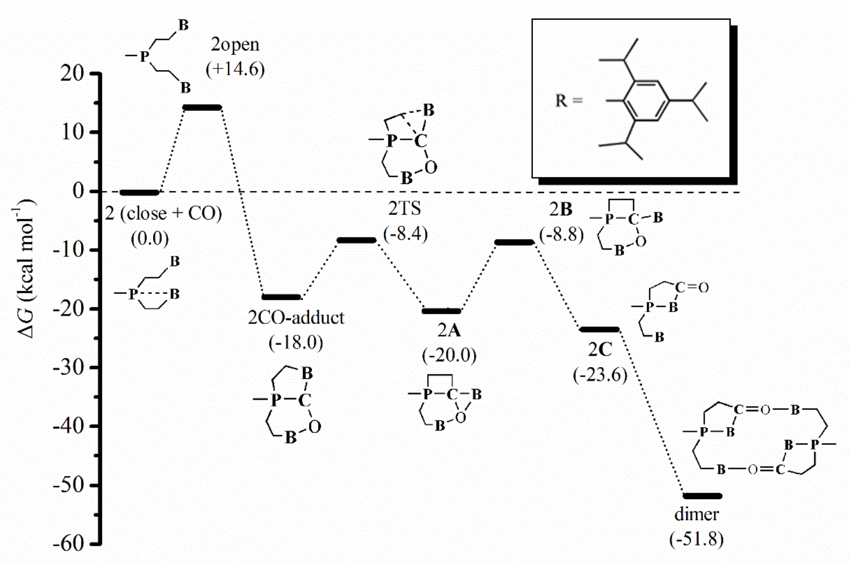

Figure S95. Computed reaction Gibbs free energy path at PW6B95-D3/def2-TZVP (COSMO-RS, toluene)//PBEh-3c level of the theory. The energy of 2 mol (close + CO) is selected as the reference.

To understand the importance of the 'second' borane, we computed Gibbs free energy for the CO insertion step with and without the ancillary borane, respectively (Fig S96). The results show that the addition of the second borane changes the thermodynamics of the CO insertion step. For example, a typical FLP could capture the CO, but CO can not be inserted into the C-B bond because the computed Δ*G* is 11.4 kcal mol $^{-1}$. After introducing the second borane, the CO inserting becomes possible with a computed Δ*G* of -4.3 kcal $^{-1}$.

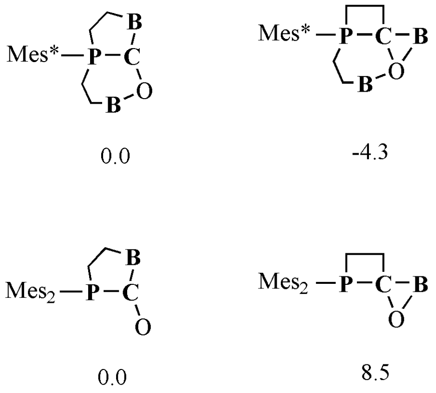

Figure S96. Computed Gibbs free energies at PW6B95-D3/def2-TZVP (COSMO-RS, toluene)//PBEh-3c level of the theory (in kcal mol1^{-1}).

To evaluate whether the observed cyclodimers/trimers might be kinetic or thermodynamic products, we computed the Gibbs free energies for alternative dimer or trimer formation in the cases of Mes* and Tipp (see Fig S97). In the Mes*-case the trimer is energetically more stable than the dimer (-16.0 *v.s* -6.2 kcal mol^{-1} per one monomer molecule). In the case of Tipp, the dimer is energetically more stable than the trimer (-12.9 *v.s* -7.6 kcal mol^{-1} per one monomer molecule).

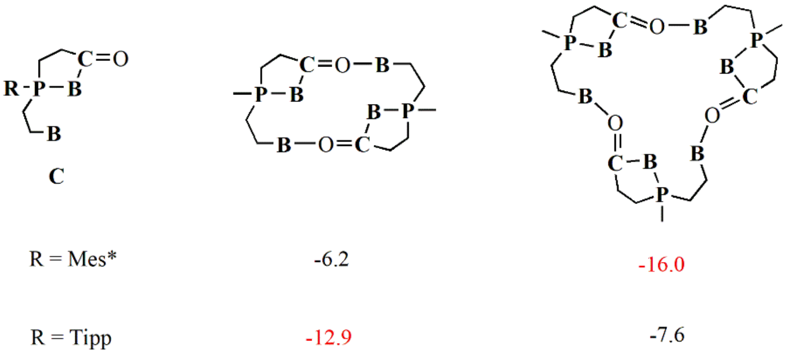

Figure S97. Computed Gibbs free energies at PW6B95-D3/def2-TZVP (COSMO-RS, toluene)//HF-3c level of the theory (in kcal mol1^{-1}).

Cartesian Coordinates

Ar = Mes*, at PBEh-3c level (Fig. S89)

CO

C	0.0000000	0.0000000	-0.5625953
O	0.0000000	0.0000000	0.5625953

CLOSE

C	-0.8667971	5.7356655	-0.6884874
C	-1.5270973	4.9994459	-1.6666723
C	-2.2195204	5.7337529	-2.6251177
C	-2.2442313	7.1148141	-2.6320624
C	-1.5785838	7.8046838	-1.6334536
C	-0.8901064	7.1152852	-0.6494021
B	-1.4874950	3.4421266	-1.7057119
C	-2.7674274	2.6502524	-2.1685189
C	-3.9169807	2.6755108	-1.4023996
C	-5.0384181	1.9325046	-1.7211333
C	-5.0166069	1.1450556	-2.8593716
C	-3.8864209	1.1055232	-3.6591769
C	-2.7840381	1.8556525	-3.2995073
F	-3.9352238	3.4198301	-0.2949336
F	-6.1194091	1.9611108	-0.9557126
F	-6.0779570	0.4268276	-3.1839894
F	-3.8729597	0.3505336	-4.7475701
F	-1.7067432	1.8174537	-4.0824835
F	-2.8571648	5.1069148	-3.6057467
F	-2.8924131	7.7801502	-3.5753694
F	-1.6014932	9.1232294	-1.6177156
F	-0.2696247	7.7807047	0.3124965
F	-0.2157388	5.1099799	0.2869228
C	-0.2380457	2.5950347	-1.2725815

C	-0.5329379	1.8481477	0.0355307
P	0.5878175	0.4228071	0.5021963
C	-0.3963647	-1.0464040	1.0546562
C	-0.5858757	-2.1461185	0.1843569
C	-0.5818467	-3.4335029	0.7198570
C	-0.3664654	-3.6889944	2.0580325
C	-0.4242526	-2.5904674	2.9045203
C	-0.5366415	-1.2840300	2.4566361
C	-0.9261979	-2.0964373	-1.3139424
C	-0.8430757	-0.7183612	-1.9513247
C	-0.1383562	-5.0835154	2.6213039
C	-1.2549378	-5.4389345	3.6115731
C	-1.0147496	-0.2857053	3.5388622
C	0.0230365	-0.0774544	4.6472971
C	1.6457018	1.1111693	1.8357283
C	2.5405876	1.8516531	0.8380987
B	2.5743251	0.7412005	-0.3571591
C	3.6521591	-0.4129262	0.0059176
C	3.4439609	-1.7116522	0.4275736
C	4.4710654	-2.5988110	0.7027009
C	5.7814638	-2.1904766	0.5572817
C	6.0442781	-0.8995964	0.1369384
C	4.9869357	-0.0486612	-0.1239910
F	2.2048196	-2.1749034	0.5875828
F	4.2043069	-3.8391009	1.1038368
F	6.7772977	-3.0259458	0.8172062
F	7.2996113	-0.4916074	-0.0068785
F	5.2922623	1.1876295	-0.5263817
C	2.6725249	1.1332472	-1.9123429
C	2.6198773	0.1536200	-2.8933603
C	2.7160235	0.4106924	-4.2484656
C	2.9156664	1.7109161	-4.6709718
C	3.0251511	2.7194735	-3.7320884
C	2.9153877	2.4155885	-2.3867485
F	2.4667939	-1.1223953	-2.5364746
F	3.0594011	3.4404717	-1.5446278
F	3.2389525	3.9689546	-4.1290863
F	3.0113579	1.9872982	-5.9625566
F	2.6253772	-0.5709500	-5.1376245
C	-0.0569401	-3.0683826	-2.1265910
C	-0.1068894	-6.1503637	1.5273870
C	1.2167701	-5.0915799	3.3425350
C	-1.4967411	1.0634568	3.0074484
C	-2.2780484	-0.9091927	4.1782777
H	3.5213828	2.0953836	1.2498917
H	2.0762689	2.8011046	0.5711134
H	1.1811359	1.7260409	2.6021329
H	2.1767696	0.2904401	2.3200374
H	-0.7017856	-4.2664033	0.0438890
H	-0.4236401	-2.7668782	3.9723543
C	-2.4002054	-2.5295979	-1.4328843
H	-0.5078788	2.5573388	0.8614485
H	-1.5393748	1.4285166	0.0341466
H	-0.0365287	1.8799402	-2.0731147
H	0.6564353	3.1997302	-1.1613022
H	-1.0595018	-6.2287264	1.0001398
H	0.6760451	-5.9572821	0.7924997
H	0.0953794	-7.1266630	1.9697458
H	1.2388461	-4.3901147	4.1776729
H	1.4300011	-6.0842804	3.7437097
H	2.0256049	-4.8203054	2.6635125
H	-1.2961816	-4.7478525	4.4543311
H	-2.2316258	-5.4270122	3.1258288
H	-1.0962440	-6.4385784	4.0205719
H	-0.3808620	0.5728217	5.4257519
H	0.2976147	-1.0192217	5.1233557
H	0.9399592	0.3780497	4.2804694
H	-2.7034141	-0.2123145	4.9031997
H	-3.0387690	-1.1149283	3.4244133
H	-2.0803076	-1.8388003	4.7085224
H	-2.1968287	0.9393235	2.1805278
H	-2.0278054	1.5918525	3.8003646
H	-0.7008511	1.7261154	2.6908169
H	-1.1393251	-0.7859463	-2.9991532
H	-1.5276525	-0.0174302	-1.4747620
H	0.1613738	-0.3025819	-1.9437052
H	-0.3026215	-4.1106516	-1.9259420
H	-0.2136371	-2.9098293	-3.1945979
H	1.0005817	-2.9349768	-1.9184724

H	-2.7109087	-2.5164624	-2.4801678
H	-2.5680042	-3.5367707	-1.0523245
H	-3.0555408	-1.8549476	-0.8790431

OPEN

C	-2.9435206	3.8094295	-2.8204754
C	-2.4115729	3.7803895	-1.5264757
C	-1.4550966	4.7639410	-1.2681460
C	-1.0205027	5.6726614	-2.2117053
C	-1.5654844	5.6414045	-3.4814087
C	-2.5387284	4.7087940	-3.7871503
B	-2.8666580	2.7305041	-0.4568960
C	-3.7656648	1.5142169	-0.9105421
C	-3.3089150	0.5398942	-1.7776080
C	-4.0591091	-0.5677893	-2.1141123
C	-5.3275044	-0.7111386	-1.5798730
C	-5.8305674	0.2532232	-0.7249601
C	-5.0413889	1.3422489	-0.4047755
F	-2.0938178	0.6598687	-2.3076241
F	-3.5769014	-1.4986574	-2.9280942
F	-6.0597611	-1.7689607	-1.8904728
F	-7.0527446	0.1249228	-0.2261984
F	-5.5540872	2.2742689	0.4011582
F	-0.8947735	4.8646893	-0.0685595
F	-0.0946328	6.5734375	-1.9126275
F	-1.1716026	6.5102949	-4.3898360
F	-3.0777312	4.6921348	-4.9970842
F	-3.9063918	2.9722625	-3.1807613
C	-2.5756074	2.8338734	1.0958221
C	-1.1894122	2.4043758	1.6236411
P	-0.4297262	0.9187464	0.7614756
C	0.2840929	-0.3152575	1.9696588
C	-0.3248207	-1.5978365	1.8888116
C	0.4466095	-2.7351120	2.1135316
C	1.7816333	-2.6787016	2.4668368
C	2.2857590	-1.4208305	2.7471983
C	1.5674574	-0.2397773	2.5832007
C	-1.8217917	-1.8627481	1.6072003
C	-2.0287261	-2.2559127	0.1417397
C	2.6524412	-3.9149513	2.6565207
C	1.9348417	-5.1982126	2.2383557
C	2.2585184	0.9784506	3.2419506
C	3.5775320	1.3302812	2.5415925
C	1.0649865	1.6883732	-0.0320263
C	1.9772508	0.6747662	-0.7531053
B	1.4597002	0.2034503	-2.1622022
C	0.8079944	-1.1790854	-2.4834959
C	1.0848797	-2.3331639	-1.7524624
C	0.5192369	-3.5571268	-2.0468524
C	-0.3724188	-3.6598616	-3.0985741
C	-0.6689471	-2.5469522	-3.8637156
C	-0.0691050	-1.3416531	-3.5564144
F	1.9614344	-2.3077280	-0.7586752
F	0.8144776	-4.6349128	-1.3301248
F	-0.9310151	-4.8225026	-3.3769889
F	-1.5045811	-2.6515971	-4.8853425
F	-0.3723082	-0.3136847	-4.3372483
C	1.7664496	1.1660458	-3.3870696
C	2.8747213	0.8551417	-4.1511131
C	3.2785256	1.6112516	-5.2349792
C	2.5429338	2.7324909	-5.5759649
C	1.4291436	3.0780153	-4.8317700
C	1.0656378	2.2949939	-3.7530769
F	3.5895354	-0.2248660	-3.8204475
F	-0.0230918	2.6484525	-3.0659441
F	0.7200316	4.1510121	-5.1608549
F	2.9037421	3.4735038	-6.6110843
F	4.3488628	1.2783656	-5.9415384
C	-2.3153682	-3.0293694	2.4876208
C	3.9265710	-3.7826535	1.8122848
C	3.0321570	-4.0363553	4.1387510
C	1.4046446	2.2344044	3.3694707
C	2.5753058	0.5855378	4.7034258
H	2.1888221	-0.1447224	-0.0704229
H	2.9343923	1.1795319	-0.9276156
H	0.6604289	2.4271077	-0.7270518
H	1.6643078	2.2586500	0.6749866
H	-0.0116612	-3.7027063	1.9864154

H	3.2930127	-1.3562300	3.1370349
C	-2.7332951	-0.6836750	1.9686835
H	-0.4939195	3.2371168	1.5625952
H	-1.2907437	2.1740438	2.6825593
H	-2.7502862	3.8595492	1.4368506
H	-3.3191425	2.2259911	1.6123313
H	4.5691721	-4.6530579	1.9567361
H	3.6905923	-3.7134229	0.7507526
H	4.5096784	-2.9007573	2.0800680
H	3.6479278	-4.9226509	4.3047807
H	3.5999343	-3.1733558	4.4886387
H	2.1437950	-4.1224720	4.7658798
H	2.6065331	-6.0505052	2.3506475
H	1.0573632	-5.3995387	2.8552904
H	1.6183228	-5.1662437	1.1955658
H	4.0753332	2.1505720	3.0624408
H	4.2691982	0.4876474	2.5260495
H	3.4270967	1.6408245	1.5082772
H	3.0014596	1.4418236	5.2298519
H	1.6690850	0.2851740	5.2311032
H	3.2910326	-0.2295536	4.7914416
H	1.9233731	2.9578393	4.0006295
H	1.2164393	2.7346641	2.4274319
H	0.4485288	2.0190094	3.8451017
H	-3.0852733	-2.4461962	-0.0600907
H	-1.6817980	-1.4715744	-0.5300665
H	-1.4840901	-3.1719223	-0.0920518
H	-3.3957058	-3.1316055	2.3750356
H	-1.8889599	-3.9940191	2.2171872
H	-2.1055346	-2.8527752	3.5434883
H	-3.7710437	-1.0206154	2.0073655
H	-2.4867751	-0.2668688	2.9468658
H	-2.7006320	0.1122031	1.2314240

ADDUCT

C	-2.1821020	3.3037634	-2.8686224
C	-2.0510925	3.4029261	-1.4872791
C	-1.5567372	4.6177861	-1.0437046
C	-1.2389123	5.6715221	-1.8830749
C	-1.4013627	5.5276574	-3.2450354
C	-1.8745590	4.3289355	-3.7431153
B	-2.4760435	2.1379289	-0.5317379
C	-3.8694510	1.5056678	-1.0856510
C	-4.0685858	0.2385903	-1.5977181
C	-5.2952261	-0.2265518	-2.0372237
C	-6.3928815	0.6094369	-1.9824038
C	-6.2449011	1.8911840	-1.4854141
C	-5.0004501	2.3076595	-1.0492420
F	-3.0418503	-0.6151880	-1.6903789
F	-5.4233982	-1.4608741	-2.5111506
F	-7.5779956	0.1863108	-2.3996863
F	-7.2942522	2.7029160	-1.4297171
F	-4.9089577	3.5549539	-0.5803277
F	-1.3351972	4.8364282	0.2611960
F	-0.7726003	6.8133612	-1.3876036
F	-1.1050817	6.5249027	-4.0634935
F	-2.0224658	4.1711929	-5.0528493
F	-2.6103495	2.1701195	-3.4218217
C	-2.6288051	2.4282125	1.0737764
C	-1.3245786	2.2875930	1.8750846
P	-0.1985784	1.1092801	1.0423776
C	0.4123484	-0.3715310	1.9350792
C	-0.1248593	-1.6606380	1.7120751
C	0.7148468	-2.7645570	1.8576877
C	2.0252766	-2.6689549	2.2764373
C	2.4519711	-1.4088777	2.6769278
C	1.6800006	-0.2625126	2.5924810
C	-1.6034165	-2.0170975	1.5015470
C	-1.8049191	-2.8085267	0.2066628
C	2.9409133	-3.8735880	2.4511590
C	2.2608080	-5.1820103	2.0498777
C	2.2296925	0.9096878	3.4495727
C	3.6244364	1.3571891	2.9916101
C	1.2824715	1.8532652	0.2793574
C	1.8818584	0.8285059	-0.6932876
B	0.9572790	0.3552049	-1.9608953
C	0.6992984	-1.2291124	-2.2094335
C	1.4492437	-2.2666310	-1.6837867
C	1.1978168	-3.5988431	-1.9627464

C	0.1572342	-3.9393121	-2.8022939
C	-0.6010119	-2.9381420	-3.3796677
C	-0.3075692	-1.6193125	-3.0878534
F	2.5070403	-2.0251923	-0.9045052
F	1.9606669	-4.5571058	-1.4379081
F	-0.0988217	-5.2119509	-3.0663126
F	-1.5817455	-3.2508466	-4.2153880
F	-1.0143599	-0.6969034	-3.7348594
C	1.3876893	1.1173923	-3.3373497
C	1.9586071	0.4822179	-4.4314205
C	2.3195243	1.1455336	-5.5933960
C	2.1192596	2.5077241	-5.6947566
C	1.5864818	3.1917194	-4.6191503
C	1.2569435	2.4902850	-3.4764450
F	2.2205997	-0.8255300	-4.4072988
F	0.8067735	3.2215202	-2.4482056
F	1.4172654	4.5071957	-4.6825642
F	2.4549323	3.1554433	-6.7993905
F	2.8620776	0.4824128	-6.6071636
C	-2.0246871	-2.8930714	2.7003028
C	4.1981811	-3.6889546	1.5922379
C	3.3417317	-3.9847716	3.9300699
C	1.3303996	2.1368158	3.6021379
C	2.3479515	0.3548410	4.8891689
H	2.2363337	-0.0070931	-0.0970308
H	2.7861691	1.2862785	-1.1016427
H	0.9649287	2.7790288	-0.2017576
H	2.0105935	2.1339308	1.0317911
H	0.3045943	-3.7407895	1.6491153
H	3.4287479	-1.3301386	3.1333356
C	-2.5394517	-0.8211320	1.5320543
H	-0.7943033	3.2361551	1.9404051
H	-1.4938160	1.9481157	2.8960820
H	-3.0455194	3.4112831	1.2825672
H	-3.3518913	1.7208807	1.4829220
H	4.8854587	-4.5215489	1.7504892
H	3.9523468	-3.6546860	0.5325583
H	4.7345171	-2.7717356	1.8401152
H	3.9686418	-4.8648080	4.0823655
H	3.9114389	-3.1216100	4.2757295
H	2.4654279	-4.0840126	4.5722498
H	2.9594675	-6.0101904	2.1729831
H	1.3922975	-5.4017415	2.6738539
H	1.9448845	-5.1803965	1.0077563
H	4.0032995	2.1337679	3.6577981
H	4.3459795	0.5411858	3.0070120
H	3.6296731	1.7637239	1.9807330
H	2.6859002	1.1449057	5.5620871
H	1.3835986	-0.0036707	5.2509499
H	3.0566062	-0.4665162	4.9752250
H	1.7590544	2.7883755	4.3646581
H	1.2377906	2.7575400	2.7153124
H	0.3358486	1.8650151	3.9532759
H	-2.8626282	-3.0233711	0.0505259
H	-1.4461249	-2.2572917	-0.6584430
H	-1.2803568	-3.7632677	0.2292844
H	-3.0859144	-3.1346928	2.6187865
H	-1.4830267	-3.8358704	2.7500650
H	-1.8717720	-2.3712793	3.6462644
H	-3.5756663	-1.1612475	1.5091337
H	-2.4174077	-0.2311541	2.4421266
H	-2.4449740	-0.1735804	0.6671678
C	-1.1399000	1.1565579	-0.6440180
O	-0.5346616	0.8863847	-1.6673844

TS

C	-3.8450177	-0.4366052	0.8212733
C	-2.6733612	-0.7438178	1.5041792
C	-2.4522131	0.0265553	2.6323433
C	-3.2761576	1.0688458	3.0245393
C	-4.4026430	1.3626476	2.2848097
C	-4.6980649	0.5927635	1.1756994
B	-1.7344433	-1.9558915	0.9020756
C	-0.3395906	-2.2715026	1.6666587
C	0.6915586	-1.1543755	1.6078526
P	1.0715970	-0.5259269	-0.0622222
C	1.6006042	1.1755167	0.4082151
C	0.6147127	2.1955990	0.5085046
C	0.6023345	2.9614132	1.6634082

C	1.5518158	2.8416305	2.6684565
C	2.6635522	2.0714322	2.3691849
C	2.7457594	1.2531007	1.2469365
F	-1.4107414	-0.2090183	3.4450956
F	-2.9974700	1.7816854	4.1148956
F	-5.2000483	2.3602487	2.6444482
F	-5.7946211	0.8470600	0.4711710
F	-4.2105862	-1.1685040	-0.2283994
C	-2.6179375	-3.3375908	0.8724984
C	-2.6470892	-4.2558152	-0.1623298
C	-3.4220571	-5.4026130	-0.1551368
C	-4.1960252	-5.6907026	0.9504111
C	-4.1687574	-4.8313081	2.0318266
C	-3.3862521	-3.6919459	1.9727322
F	-1.8852936	-4.0856369	-1.2510704
F	-3.3836025	-2.9208865	3.0651006
F	-4.8865357	-5.1098579	3.1148169
F	-4.9421158	-6.7868644	0.9813801
F	-3.4165580	-6.2286382	-1.1969939
O	-1.4531139	-1.4962330	-0.5388500
C	-0.3592644	-1.1737991	-1.1461101
B	-0.1819384	-1.4533913	-2.6433162
C	-1.3796903	-2.2285934	-3.3496099
C	-2.6914608	-1.7829402	-3.2885381
C	-3.7325167	-2.4556856	-3.9032579
C	-3.4705374	-3.6068139	-4.6227205
C	-2.1710412	-4.0639675	-4.7383139
C	-1.1602698	-3.3610538	-4.1146047
F	-2.9905396	-0.6533378	-2.6632820
F	-4.9744695	-1.9980905	-3.8250682
F	-4.4571078	-4.2557350	-5.2197966
F	-1.9071877	-5.1555982	-5.4452001
F	0.0883419	-3.8227925	-4.2663382
C	2.0579697	-1.8088334	-0.9115055
C	0.8536709	-2.5545345	-1.4562395
C	0.7723754	-0.7393196	-3.7224446
C	0.1807057	0.3283911	-4.3906488
C	0.7694988	1.0099080	-5.4359683
C	2.0123660	0.6041399	-5.8865431
C	2.6228825	-0.4810126	-5.2921466
C	1.9935324	-1.1386516	-4.2472235
F	-1.0332717	0.7246138	-4.0172113
F	0.1574956	2.0369737	-6.0075893
F	2.6034332	1.2401554	-6.8836657
F	3.8021096	-0.8998687	-5.7313281
F	2.6422144	-2.2089867	-3.7750422
C	-0.2976765	2.6297711	-0.6492541
C	1.3938132	3.6134315	3.9687644
C	4.1155806	0.6135139	0.9413332
H	-0.5277787	-2.4865221	2.7201528
H	0.0957487	-3.1978220	1.2778896
H	1.6527642	-1.4323090	2.0501924
H	0.3552735	-0.2820949	2.1642452
H	-0.1798819	3.6978063	1.7779967
H	3.5082328	2.1048881	3.0388410
H	2.6650381	-2.4050071	-0.2347141
H	2.7069386	-1.4206252	-1.6854013
H	0.4263559	-3.2758387	-0.7689995
H	1.0617329	-3.1181755	-2.3657752
C	5.2487844	1.4644669	1.5462822
C	4.2821238	-0.7856858	1.5440379
C	4.3741452	0.6371478	-0.5712810
C	-0.5943398	4.1367210	-0.5540361
C	0.4788729	2.4387073	-1.9530544
C	-1.6496137	1.9197143	-0.6666725
C	2.5047393	3.2926615	4.9685477
C	1.4205143	5.1200502	3.6776887
C	0.0474066	3.2326173	4.5983458
H	1.3013082	5.6857491	4.6031659
H	2.3655297	5.4194384	3.2221005
H	0.6171351	5.4239308	3.0058980
H	2.3228104	3.8251368	5.9026032
H	2.5465155	2.2280483	5.2050286
H	3.4880512	3.6040861	4.6116303
H	-0.0814624	3.7470243	5.5520909
H	-0.7962874	3.5073133	3.9663625
H	-0.0193827	2.1602693	4.7834619
H	6.2014760	1.1148336	1.1459487
H	5.1499599	2.5193404	1.2895521

H	5.3201074	1.3796385	2.6306457
H	5.3045019	0.1178473	-0.8064858
H	3.5882729	0.1871717	-1.1668205
H	4.4706202	1.6661883	-0.9196032
H	5.2995705	-1.1454294	1.3815136
H	4.1078985	-0.7636682	2.6208111
H	3.6166400	-1.5320913	1.1230971
H	-1.0682270	4.4566346	-1.4829028
H	-1.2910215	4.3852492	0.2467457
H	0.3108137	4.7311393	-0.4222487
H	-0.1161584	2.7741009	-2.8016378
H	1.4012914	3.0209137	-1.9376896
H	0.7581266	1.4024908	-2.1268832
H	-2.2804539	2.3336435	-1.4546866
H	-1.5866211	0.8553770	-0.8486127
H	-2.1687278	2.0622662	0.2819878

A

C	-2.7435050	3.3043073	-2.6989124
C	-2.5881201	2.7493565	-1.4401294
C	-3.3213413	3.3551018	-0.4360050
C	-4.1313492	4.4572107	-0.6324455
C	-4.2367488	4.9910114	-1.9025723
C	-3.5405834	4.4085306	-2.9441758
B	-1.6459502	1.4917843	-1.1069413
C	-2.3003601	0.0302767	-1.1301958
C	-3.6194764	-0.1944477	-1.5065133
C	-4.1825481	-1.4577735	-1.5861592
C	-3.4143269	-2.5671952	-1.2967530
C	-2.0961796	-2.3939149	-0.9219505
C	-1.5789945	-1.1178075	-0.8402635
F	-4.4267784	0.8152202	-1.8159793
F	-5.4493006	-1.6097025	-1.9462901
F	-3.9302417	-3.7825590	-1.3787445
F	-1.3380451	-3.4556477	-0.6646152
F	-0.2962173	-1.0277817	-0.4716910
F	-3.2727082	2.8547046	0.8061145
F	-4.8006856	5.0023278	0.3782651
F	-5.0052250	6.0480369	-2.1209805
F	-3.6507849	4.9054704	-4.1692179
F	-2.1362391	2.7660224	-3.7507770
C	0.0621408	3.3089232	0.2018380
C	1.1229629	2.9619049	1.2661613
P	0.9601046	1.1857243	0.8622627
C	0.8236927	-0.1485769	2.0955584
C	-0.4268091	-0.6963474	2.4646211
C	-0.5167061	-2.0807342	2.5954644
C	0.5770588	-2.9200248	2.4748076
C	1.8262766	-2.3050541	2.4298337
C	1.9918629	-0.9380744	2.3099447
C	-1.6177292	0.1158155	2.9925817
C	-2.9385647	-0.1967555	2.2898843
C	0.4835221	-4.4369247	2.5482491
C	1.2081279	-5.0408263	1.3379845
C	3.3739252	-0.3824452	2.7210352
C	3.5829015	-0.8822831	4.1702179
C	2.2896287	0.7202823	-0.2946124
C	2.2622577	1.5069031	-1.6091033
B	1.0020703	1.2259879	-2.5794633
C	0.7685645	-0.3252794	-3.0502959
C	-0.4272494	-0.6876214	-3.6501740
C	-0.7509358	-1.9793512	-4.0237144
C	0.1745350	-2.9857939	-3.8351650
C	1.4072977	-2.6693087	-3.2994914
C	1.6809690	-1.3626208	-2.9365830
F	-1.3542963	0.2415285	-3.8839290
F	-1.9421287	-2.2587928	-4.5421884
F	-0.1099812	-4.2351860	-4.1736893
F	2.3213957	-3.6210618	-3.1379017
F	2.9189440	-1.1499579	-2.4687880
C	1.0521434	2.2197256	-3.8751647
C	0.6179566	3.5358887	-3.8865379
C	0.6638195	4.3584300	-4.9970706
C	1.1969436	3.8768767	-6.1752599
C	1.6858208	2.5864558	-6.2107067
C	1.6132180	1.7987384	-5.0748795
F	0.1154020	4.1009008	-2.7817000
F	2.1483993	0.5800070	-5.1831960
F	2.2253355	2.1139205	-7.3288584

F	1.2524026	4.6471394	-7.2523662
F	0.2089360	5.6053144	-4.9342807
C	-1.7458040	-0.2847649	4.4787432
C	1.1556627	-4.9197691	3.8419639
C	-0.9629906	-4.9306318	2.5486177
C	4.5253723	-0.9172458	1.8636272
C	3.4537832	1.1444483	2.8315412
H	3.1893745	1.2813596	-2.1380090
H	2.3339066	2.5804774	-1.3982167
H	3.2383454	0.8685266	0.2139687
H	2.1931963	-0.3510649	-0.4525456
H	-1.4834532	-2.5074269	2.8227340
H	2.7086714	-2.9210867	2.5466753
C	-1.3740216	1.6186898	2.9866352
H	2.1188789	3.3512626	1.0596003
H	0.8546457	3.2181232	2.2869313
H	0.4706464	3.9075133	-0.6046674
H	-0.7837690	3.8534696	0.6195197
H	2.2125734	-4.6536372	3.8844364
H	0.6709742	-4.4955526	4.7225117
H	1.0922248	-6.0067925	3.9163255
H	-0.9784136	-6.0210898	2.5502912
H	-1.5107794	-4.6049200	3.4347845
H	-1.5089894	-4.6001578	1.6655246
H	2.2641458	-4.7701218	1.3120560
H	1.1536634	-6.1302163	1.3711537
H	0.7607161	-4.7112701	0.4012980
H	2.7754507	-0.5472894	4.8228933
H	3.6323996	-1.9675538	4.2379271
H	4.5207581	-0.4872125	4.5649429
H	3.5136511	1.6709710	1.8828169
H	2.6172753	1.5509367	3.4029820
H	4.3642604	1.4128018	3.3687710
H	4.4607252	-0.6058331	0.8228154
H	5.4797506	-0.5623420	2.2556638
H	4.5618827	-2.0065542	1.8686254
H	-3.7624094	0.2927765	2.8113778
H	-2.9433149	0.1601211	1.2652628
H	-3.1547469	-1.2651952	2.2728632
H	-2.0237750	-1.3298015	4.6085417
H	-0.8118153	-0.1197049	5.0180330
H	-2.5198993	0.3209080	4.9538548
H	-0.4661216	1.8754853	3.5358343
H	-1.3269637	2.0324136	1.9847278
H	-2.2041259	2.1270791	3.4778453
C	-0.3442600	1.8902555	-0.2661521
O	-0.2457876	1.5996370	-1.6535195

B

C	-4.5199800	-1.4902841	0.1134547
C	-3.5057397	-1.3757690	1.0560192
C	-3.8298795	-0.5845087	2.1448153
C	-5.0512460	0.0516357	2.2985826
C	-6.0225235	-0.0964703	1.3305333
C	-5.7535148	-0.8761489	0.2222793
B	-2.0872862	-2.1746951	0.7794792
C	-0.9531334	-2.0739490	1.9684008
C	-0.1002924	-0.8058469	1.9630344
P	0.8019573	-0.5199858	0.3990303
C	1.5302165	1.1618531	0.7003173
C	0.6214853	2.2491551	0.8865280
C	0.9854062	3.2436311	1.7784275
C	2.2094676	3.2826071	2.4330319
C	3.1485185	2.3558788	2.0299260
C	2.8661516	1.3139330	1.1476499
F	-2.9480489	-0.3634668	3.1312598
F	-5.2940029	0.8065566	3.3677770
F	-7.1990202	0.5037336	1.4612885
F	-6.6803990	-1.0326011	-0.7167496
F	-4.3378577	-2.2409647	-0.9772700
C	-2.5000714	-3.7662813	0.5807549
C	-2.3769532	-4.5254938	-0.5713680
C	-2.7817372	-5.8468505	-0.6690774
C	-3.3518131	-6.4667034	0.4242105
C	-3.5071293	-5.7525569	1.5969746
C	-3.0819097	-4.4378704	1.6471523
F	-1.8429549	-4.0099534	-1.6887127
F	-3.2745464	-3.7984836	2.8064719
F	-4.0603064	-6.3335642	2.6568798

F	-3.7455760	-7.7320708	0.3513328
F	-2.6285761	-6.5198931	-1.8063857
O	-1.5931355	-1.5497926	-0.5023138
C	-0.3191637	-1.4794039	-0.9102828
B	-0.2454594	-1.1490866	-2.4768781
C	-1.5247594	-1.4825615	-3.3109865
C	-2.7606038	-0.8812720	-3.1462175
C	-3.8593107	-1.2516073	-3.8962312
C	-3.7309535	-2.2578667	-4.8377116
C	-2.5092869	-2.8776679	-5.0346791
C	-1.4310122	-2.4694845	-4.2775917
F	-2.9021063	0.1324358	-2.3017222
F	-5.0247337	-0.6434605	-3.7381069
F	-4.7766647	-2.6205106	-5.5600237
F	-2.3861518	-3.8393824	-5.9385142
F	-0.2574207	-3.0810851	-4.4626609
C	1.7546238	-2.0867054	0.2009208
C	0.6070330	-2.7153731	-0.5934834
C	0.9801963	-0.6253884	-3.3378971
C	0.7395240	0.4522391	-4.1895544
C	1.6946201	0.9865810	-5.0321345
C	2.9503154	0.4102407	-5.0806818
C	3.2243138	-0.6889601	-4.2901084
C	2.2456252	-1.1839453	-3.4485859
F	-0.4578679	1.0281095	-4.2063064
F	1.4144378	2.0319779	-5.7942961
F	3.8768731	0.9002234	-5.8830385
F	4.4181688	-1.2621685	-4.3416989
F	2.5709354	-2.2742031	-2.7558668
C	-0.6555411	2.4884585	0.0553219
C	2.4924735	4.3680982	3.4612341
C	4.0664584	0.4712675	0.6887203
H	-1.4103452	-2.1344690	2.9538341
H	-0.2885823	-2.9440804	1.9430142
H	0.6818184	-0.8205115	2.7317879
H	-0.6976180	0.0802947	2.1633630
H	0.2862929	4.0437293	1.9649184
H	4.1528108	2.4383928	2.4117501
H	1.9436167	-2.5755219	1.1554533
H	2.6875757	-2.0240041	-0.3422028
H	0.0580441	-3.4316902	0.0084696
H	0.9383875	-3.2530625	-1.4787236
C	5.3866298	1.2621074	0.7780148
C	4.2545998	-0.7499041	1.6011249
C	3.9318344	0.1503921	-0.8079835
C	-1.0997059	3.9581758	0.1189838
C	-0.3329132	2.2128261	-1.4125734
C	-1.8558041	1.6554342	0.5032538
C	3.8750768	4.2180155	4.0958630
C	2.4175266	5.7428157	2.7825183
C	1.4390218	4.2845501	4.5739589
H	2.6217863	6.5342834	3.5056240
H	3.1504203	5.8267491	1.9787676
H	1.4336527	5.9391802	2.3552506
H	4.0174279	4.9884732	4.8543503
H	3.9967632	3.2516486	4.5883323
H	4.6797483	4.3350581	3.3678360
H	1.6197259	5.0556349	5.3247146
H	0.4264505	4.4296677	4.1964036
H	1.4682905	3.3162705	5.0751601
H	6.1683784	0.6915738	0.2736042
H	5.3173066	2.2321859	0.2849210
H	5.7320412	1.4188854	1.7996182
H	4.6399049	-0.6232061	-1.1101566
H	2.9404644	-0.1615267	-1.1125849
H	4.1508001	1.0446658	-1.3935946
H	5.0098768	-1.4281718	1.1994890
H	4.5962771	-0.4224854	2.5837692
H	3.3496375	-1.3216741	1.7707992
H	-1.8988742	4.1085081	-0.6073691
H	-1.5100635	4.2370028	1.0902879
H	-0.2952658	4.6511990	-0.1314156
H	-1.2126167	2.3836949	-2.0305042
H	0.4704908	2.8588410	-1.7707784
H	-0.0121428	1.1864957	-1.5680870
H	-2.7474422	1.9731188	-0.0391299
H	-1.7553986	0.5949563	0.2984315
H	-2.0560391	1.7912023	1.5667630

C

C	-2.2014216	3.3111488	-0.1323803
H	-1.8884070	4.2385589	0.3467566
H	-2.6909299	2.6982164	0.6184029
H	-2.9496961	3.5724631	-0.8821481
C	2.4803502	4.6578473	2.1011196
C	2.8348570	4.2928648	3.5482517
H	1.9542661	3.9612073	4.0973168
H	3.2506841	5.1585299	4.0670823
H	3.5770930	3.4951940	3.6007586
C	1.5001817	5.8306165	2.1224232
H	1.2679021	6.1927273	1.1193965
H	1.9384700	6.6669108	2.6685374
H	0.2688012	-1.0504436	-1.5532409
C	-0.2005594	-3.0280926	-0.7794463
H	-1.0036279	-3.5131781	-0.2326099
H	0.5647548	5.5714187	2.6184082
C	3.7474780	5.1029753	1.3573934
H	4.5176230	4.3301652	1.3534349
H	4.1764397	5.9865017	1.8336076
H	3.5275346	5.3557190	0.3190900
C	3.2730543	0.0869821	0.3737089
C	2.8306069	-1.1272765	-0.4369891
H	3.7191743	-1.6730155	-0.7584793
H	2.2956513	-0.8275863	-1.3379959
H	2.2244980	-1.8392761	0.1126487
C	4.1271499	-0.3798325	1.5602614
H	3.5808774	-1.0135716	2.2584195
H	4.5315565	0.4534986	2.1333596
H	4.9758199	-0.9626228	1.1981211
C	4.1924260	0.8738883	-0.5901352
H	4.6928142	1.7113784	-0.1074306
H	3.6257553	1.2715452	-1.4331246
H	4.9670707	0.2139996	-0.9863269
B	-1.9781018	-0.4317244	1.8400566
C	-2.3902988	0.9842113	2.5015352
C	-3.6946301	1.3318340	2.8343378
F	-4.7129619	0.5177590	2.5737535
C	-4.0224004	2.5113688	3.4828605
F	-5.2898509	2.8086583	3.7399906
C	-3.0223782	3.3720064	3.8889979
F	-3.3187611	4.5019188	4.5153448
C	-1.7051891	3.0182085	3.6733723
F	-0.7284181	3.8014183	4.1260773
C	-1.4242778	1.8353457	3.0201784
F	-0.1345219	1.4830253	2.9513973
C	-3.1023759	-1.2563077	1.0190178
C	-4.1090444	-0.6999825	0.2395699
F	-4.1573558	0.6172827	0.0338218
C	-5.1184605	-1.4384111	-0.3510217
F	-6.0626611	-0.8408406	-1.0677171
C	-5.1421896	-2.8098247	-0.1909858
F	-6.0986125	-3.5338540	-0.7516729
C	-4.1443470	-3.4190140	0.5425601
F	-4.1249800	-4.7421262	0.6681709
C	-3.1586349	-2.6406963	1.1186525
F	-2.1999940	-3.3048477	1.7775657
H	0.7256914	-3.2678508	-0.2526489
C	0.7067249	-1.4957478	1.9617734
H	0.7628369	-2.5078698	1.5625361
C	0.7808272	1.0488796	0.3753552
H	1.7204981	-1.1323602	2.0614261
C	0.0038194	-1.5190656	3.3125200
H	0.1704221	-2.4683113	3.8238813
H	0.4081874	-0.7424896	3.9648692
C	-1.4869833	-1.2608774	3.1731611
H	-2.3378850	1.6304925	-2.2206501
O	-2.2596958	-1.5497533	4.0511748
C	-0.4525002	3.4861611	-1.9033362
H	0.4129062	3.0241149	-2.3808347
H	-0.1425719	4.4610954	-1.5297924
H	-1.2089202	3.6570172	-2.6721049
C	-0.4104576	-1.5081259	-0.8350620
H	-1.4147246	-1.3067466	-1.1974089
C	0.1340060	2.2951505	0.1659554
C	0.7010278	3.4401968	0.7236135
B	-0.0366437	-3.5902675	-2.2413572
C	-1.1379280	-4.4069105	-2.9849002
F	0.4462073	-5.9144816	-3.8504792

C	-0.8182985	-5.5174624	-3.7580823
F	-1.4397783	-7.3312520	-5.1246298
C	-1.7760487	-6.2657548	-4.4147046
F	-4.0298313	-6.5822751	-4.9586552
C	-3.1051567	-5.8872316	-4.3257931
F	-4.7345771	-4.4068902	-3.5168348
C	-3.4669093	-4.7782363	-3.5793360
F	-2.8529162	-2.9877555	-2.2412979
H	0.1720053	4.3747279	0.6077387
C	1.8978438	3.4330859	1.4112903
C	-2.4830733	-4.0697402	-2.9201839
C	2.6342402	2.2588173	1.3316795
H	3.6576139	2.2748189	1.6834482
P	-0.2276793	-0.4771197	0.7200327
C	2.1551002	1.0882294	0.7664429
C	-1.0332998	2.5775157	-0.7984222
C	-1.5540212	1.3449619	-1.5184996
H	-2.0019589	0.6259487	-0.8432295
H	-0.7659929	0.8583995	-2.0938428
C	1.3085342	-3.2914502	-3.0016032
F	2.5948413	-4.5143447	-1.4713087
C	2.5363450	-3.7499109	-2.5613304
F	4.8757877	-3.9132945	-2.7741220
C	3.7137609	-3.4612734	-3.2227076
F	4.7893062	-2.3799191	-5.0011684
C	3.6708314	-2.6755969	-4.3621834
F	2.4254258	-1.4400882	-5.9193269
C	2.4603220	-2.1968059	-4.8331376
F	0.1419171	-2.0463950	-4.6019709
C	1.3030190	-2.5208283	-4.1500081

TRIMER (HF-3c)

P	-0.8865570	-0.8285714	4.6760905
C	-1.5248185	-0.8677071	2.9430402
H	-2.5189807	-0.4872829	2.8089748
H	-0.8602620	-0.2379496	2.3653025
C	-1.3996835	-2.2968794	2.4104921
H	-2.1079541	-2.9611916	2.9046472
H	-1.5825042	-2.3417943	1.3424614
C	0.0118396	-2.7747648	2.7693728
O	0.5526443	-3.7198523	2.1832691
C	-0.5786461	1.0041744	4.9850782
H	-1.3867821	1.3440916	5.6213297
H	0.3405638	1.1201059	5.5462172
C	-0.5130191	1.8723327	3.7009433
H	-1.3088165	1.5846000	3.0352480
H	0.4133581	1.6659163	3.1905917
C	-2.1094216	-1.6340615	5.8062894
C	-3.4242663	-1.9335621	5.3355142
C	-4.0194670	-3.1042448	5.7601726
H	-4.9710106	-3.3755781	5.3477780
C	-3.4741854	-3.9120040	6.7509609
C	-2.3690721	-3.4255046	7.4129696
H	-2.0172756	-3.9305111	8.2899488
C	-1.6771409	-2.2953021	6.9811210
C	-4.3913513	-0.9663934	4.5874037
C	-4.8088178	-1.4721515	3.1776101
H	-5.5313414	-0.7845545	2.7527793
H	-5.2813941	-2.4425208	3.2697827
H	-3.9876845	-1.5699356	2.4857165
C	-3.9291638	0.5144118	4.5965976
H	-3.7756172	0.8331280	5.6196784
H	-4.7234832	1.1141311	4.1727906
H	-3.0374538	0.7270786	4.0421275
C	-5.7087388	-0.9225715	5.4340875
H	-5.4858774	-0.6901375	6.4680317
H	-6.2462954	-1.8579040	5.3956782
H	-6.3547269	-0.1493447	5.0363811
C	-4.1873780	-5.2118297	7.1429498
C	-4.2280240	-6.1416786	5.9007627
H	-3.2204760	-6.3867326	5.5859057
H	-4.7469339	-5.6624639	5.0809987
H	-4.7462200	-7.0610860	6.1467962
C	-3.4603056	-5.9437670	8.2939133
H	-3.9775684	-6.8708472	8.5082700
H	-3.4548767	-5.3419549	9.1935320
H	-2.4399141	-6.1785534	8.0169367
C	-5.6374726	-4.8879836	7.5959685
H	-6.1357252	-5.8029492	7.8940100

H	-6.2048700	-4.4359063	6.7934146
H	-5.6269429	-4.2084359	8.4400048
C	-0.6353858	-1.7834040	8.0142948
C	0.0845622	-0.4840468	7.6181212
H	0.7258102	-0.6295387	6.7651079
H	0.7168177	-0.1725225	8.4405015
H	-0.6263400	0.3051585	7.4300135
C	0.4364257	-2.8376419	8.3909433
H	1.1337941	-2.9817951	7.5806926
H	-0.0081543	-3.7884323	8.6493990
H	0.9901154	-2.4774486	9.2498805
C	-1.4619561	-1.4486699	9.2960027
H	-0.8050475	-1.0081769	10.0366248
H	-1.9103315	-2.3370488	9.7189450
H	-2.2475058	-0.7393715	9.0642770
B	0.7355017	-2.0287565	4.0845205
C	1.2003513	-3.2725339	5.0121212
C	2.5033345	-3.5720733	5.4266993
F	3.5404183	-2.8155940	5.1124847
C	2.8038498	-4.7312728	6.1852719
F	4.0498131	-4.9550504	6.5749593
C	1.7855581	-5.6395263	6.5227545
F	2.0505569	-6.7138410	7.2478054
C	0.4766001	-5.3971796	6.0548166
F	-0.4995041	-6.2509375	6.3284539
C	0.2282097	-4.2420433	5.2957294
F	-0.9891884	-4.0914083	4.7856383
C	1.8609071	-0.9616109	3.6203194
C	2.7504052	-0.3756744	4.5302066
F	2.6579751	-0.6487074	5.8212814
C	3.7611409	0.5260113	4.1337442
F	4.6074097	1.0204708	5.0222502
C	3.8574913	0.9038605	2.7780695
F	4.7770110	1.7709987	2.3821450
C	2.9464550	0.3653330	1.8510141
F	2.9997272	0.7392207	0.5802863
C	1.9744986	-0.5584925	2.2899097
F	1.1220056	-1.0290700	1.3839493
B	0.1274860	-4.6510097	0.9959931
C	1.3290445	-5.7576672	0.8465435
C	2.6304964	-5.5367794	1.2994971
F	2.9712591	-4.3987195	1.8903115
C	3.6597831	-6.4979460	1.1519489
F	4.8736394	-6.2489233	1.6221338
C	3.3926571	-7.7159973	0.5024304
F	4.3443613	-8.6241185	0.3469990
C	2.0917434	-7.9515499	0.0112945
F	1.8116594	-9.0834615	-0.6212095
C	1.0945774	-6.9758897	0.1970031
F	-0.1127865	-7.2589190	-0.2885250
C	-1.2631683	-5.3876981	1.5010200
C	-2.5573566	-5.0264598	1.1174282
F	-2.7933073	-3.9575631	0.3651338
C	-3.7121760	-5.7350647	1.5219378
F	-4.9076762	-5.3353892	1.1054716
C	-3.5880812	-6.8536800	2.3613828
F	-4.6557795	-7.5406706	2.7361246
C	-2.3019347	-7.2174357	2.8112583
F	-2.1526897	-8.2548974	3.6221953
C	-1.1801350	-6.4732568	2.3913672
F	-0.0119006	-6.8498079	2.8938244
P	1.0654206	4.6966533	-1.0178776
C	1.9827602	3.7597057	0.2972981
H	1.9093967	2.7048228	0.0554404
H	3.0252195	4.0185251	0.3205015
C	1.3719580	4.0069375	1.6917044
H	1.5994141	3.1914204	2.3703474
H	1.7813080	4.9200841	2.1265170
C	-0.1385667	4.2261060	1.5493763
O	-0.9284856	4.0466914	2.4830759
C	0.8644800	3.5591691	-2.5058243
H	-0.1528756	3.6853338	-2.8509261
H	1.5029070	3.9874855	-3.2639509
C	1.1498983	2.0330644	-2.4009017
H	0.3408914	1.5551810	-1.8719584
H	2.0512436	1.8482419	-1.8361229
C	2.0432560	6.2146178	-1.4426516
C	1.3879808	7.4158642	-1.8057904
C	1.9680209	8.6331029	-1.4544436

H	1.4436969	9.5334031	-1.7026223
C	3.1797854	8.7158962	-0.8066149
C	3.9141434	7.5427579	-0.6986431
H	4.9277151	7.6073177	-0.3557274
C	3.4191796	6.3067060	-1.0657465
C	0.1666639	7.5787709	-2.7532969
C	-0.4303476	6.2610008	-3.2765491
H	-0.8471940	5.6675709	-2.4838172
H	0.3127058	5.6968813	-3.8193946
H	-1.2376834	6.4936389	-3.9600538
C	0.7133336	8.3232986	-4.0125438
H	1.5450206	7.7777855	-4.4425730
H	1.0430268	9.3235316	-3.7689707
H	-0.0749327	8.3918782	-4.7527201
C	-0.9706191	8.4376460	-2.1424066
H	-0.5950595	9.3711958	-1.7472266
H	-1.4806022	7.9030052	-1.3566620
H	-1.6923895	8.6629755	-2.9183736
C	3.7995807	10.0409910	-0.3458426
C	4.2023781	9.9179664	1.1481736
H	3.3292752	9.7157293	1.7580639
H	4.6505195	10.8463085	1.4816434
H	4.9191058	9.1199718	1.2902720
C	2.8134348	11.2213741	-0.5021026
H	2.5675111	11.3870025	-1.5429100
H	3.2724332	12.1227397	-0.1150489
H	1.9021066	11.0357704	0.0529795
C	5.0653959	10.3286994	-1.1999362
H	5.8023348	9.5462210	-1.0741213
H	5.5061816	11.2693376	-0.8914115
H	4.8056131	10.3951635	-2.2499335
C	4.5392550	5.2349962	-1.2511470
C	4.1116117	3.9407521	-1.9808049
H	5.0069090	3.3809117	-2.2140977
H	3.6236704	4.1930005	-2.9092627
H	3.4755620	3.2894439	-1.4151522
C	5.3114037	4.9175782	0.0603110
H	4.6982172	4.4552902	0.8208308
H	5.7312417	5.8239405	0.4772528
H	6.1268326	4.2402401	-0.1672882
C	5.5702871	5.8814219	-2.2371211
H	6.0532395	6.7454696	-1.8056321
H	5.0758632	6.1822203	-3.1528764
H	6.3341421	5.1523807	-2.4790513
B	-0.6625992	4.9011372	0.1215450
C	-1.0658958	6.3991021	0.6098484
C	-2.3818335	6.8347055	0.8269927
F	-3.4226725	6.0389813	0.6527090
C	-2.6802156	8.1408614	1.2801522
F	-3.9423985	8.5127363	1.4327473
C	-1.6390330	9.0426279	1.5718900
F	-1.9049020	10.2744241	1.9738794
C	-0.3084126	8.5950204	1.4607770
F	0.6981381	9.3951498	1.7820948
C	-0.0585036	7.2830499	1.0152040
F	1.2042374	6.8748583	1.0349693
C	-1.8092108	3.9498247	-0.5135057
C	-2.8797619	4.3718577	-1.3113244
F	-2.9802235	5.6292692	-1.7116233
C	-3.9073273	3.4923738	-1.7264954
F	-4.9196609	3.9494985	-2.4472125
C	-3.8562250	2.1316396	-1.3612018
F	-4.8153200	1.2949615	-1.7246469
C	-2.7574580	1.6701303	-0.6092151
F	-2.6624999	0.3876615	-0.2734238
C	-1.7640278	2.5851855	-0.2140346
F	-0.7338914	2.1038589	0.4804101
B	-0.7683829	3.4801476	3.9346131
C	0.3615384	4.4274385	4.6424992
C	1.5716154	3.9941225	5.1781749
F	1.9323493	2.7197144	5.0918405
C	2.4785458	4.8698522	5.8204739
F	3.6163140	4.4094656	6.3197066
C	2.1671469	6.2380029	5.9227985
F	3.0024527	7.0745834	6.5184408
C	0.9556784	6.7085261	5.3725006
F	0.6460235	7.9947135	5.4512755
C	0.0858383	5.7969707	4.7430155
F	-1.0350494	6.2836002	4.2224176

C	-2.2198575	3.7091936	4.6539783
C	-2.3304410	3.8726914	6.0367058
F	-1.2503133	3.8270063	6.8105486
C	-3.5688395	4.0625410	6.6863493
F	-3.6186959	4.2067539	8.0038234
C	-4.7536238	4.0871286	5.9255036
F	-5.9281682	4.2613303	6.5134109
C	-4.6723559	3.8960989	4.5326605
F	-5.7784869	3.8719282	3.8008359
C	-3.4126430	3.6969969	3.9263562
F	-3.4124027	3.4512091	2.6222878
P	0.0456932	-3.7598588	-3.3137781
C	-1.0574799	-2.2935116	-3.0414708
H	-1.5877708	-2.1096017	-3.9689499
H	-1.7926215	-2.4617482	-2.2689689
C	-0.2050378	-1.0573459	-2.6786980
H	-0.7369652	-0.1384360	-2.9038509
H	0.0267753	-1.0554877	-1.6132737
C	1.1359831	-1.1287584	-3.4307964
O	1.7171311	-0.1160599	-3.8337144
C	0.3049581	-4.5848434	-1.6515861
H	-0.3241878	-5.4646928	-1.6288267
H	1.3291550	-4.9312380	-1.6341011
C	0.0536689	-3.7239815	-0.3796194
H	-0.8868714	-3.2108215	-0.4654851
H	0.8237860	-2.9681162	-0.3178221
C	-0.8394821	-4.7776713	-4.5978231
C	-0.1215813	-5.4620797	-5.5983653
C	-0.6583581	-5.5539814	-6.8838208
H	-0.0763718	-6.0401012	-7.6405559
C	-1.8976342	-5.0497577	-7.2004077
C	-2.6938760	-4.6355236	-6.1363818
H	-3.7258796	-4.4168690	-6.3275530
C	-2.2355595	-4.5634959	-4.8394944
C	-2.4911733	-5.1018352	-8.6137110
C	-3.6065908	-6.1843556	-8.6388017
H	-4.3930587	-5.9413568	-7.9357956
H	-3.1977385	-7.1541117	-8.3806120
H	-4.0362185	-6.2418205	-9.6320207
C	-1.4215033	-5.4606097	-9.6712141
H	-1.8673607	-5.4206386	-10.6574113
H	-1.0388952	-6.4604816	-9.5142731
H	-0.5989094	-4.7567179	-9.6379736
C	-3.1078951	-3.7233092	-8.9700586
H	-2.3476210	-2.9511828	-8.9463560
H	-3.8947381	-3.4591443	-8.2764300
H	-3.5299359	-3.7628562	-9.9670334
C	1.1377447	-6.3352100	-5.3825028
C	2.2802830	-5.9702704	-6.3638356
H	3.1408920	-6.5978250	-6.1647210
H	2.5765291	-4.9383446	-6.2570178
H	1.9697857	-6.1372207	-7.3863953
C	0.6943968	-7.8009889	-5.6871993
H	0.3964784	-7.9157093	-6.7199598
H	-0.1355690	-8.0851730	-5.0514691
H	1.5253092	-8.4688903	-5.4921421
C	1.6321593	-6.3527292	-3.9234668
H	2.4633652	-7.0423412	-3.8397358
H	0.8425999	-6.6941938	-3.2684079
H	1.9791167	-5.3901669	-3.6015547
C	-3.3675079	-4.5265828	-3.7705725
C	-4.3959426	-5.6319042	-4.1819365
H	-5.1084379	-5.7620627	-3.3767210
H	-3.8908474	-6.5742803	-4.3538456
H	-4.9443554	-5.3624351	-5.0721913
C	-2.8834999	-4.9685896	-2.3726937
H	-2.2179942	-4.2694512	-1.9084672
H	-2.3901794	-5.9294316	-2.4438217
H	-3.7451259	-5.0762995	-1.7268294
C	-4.1565315	-3.1901835	-3.7186827
H	-3.5904552	-2.3800053	-3.2876372
H	-5.0465854	-3.3319317	-3.1165919
H	-4.4636341	-2.9026210	-4.7170353
B	1.7356544	-2.6377098	-3.8338090
C	3.0115305	-3.0019889	-2.8925148
F	3.9383559	-4.6331860	-4.3483872
C	4.0015232	-3.9382968	-3.2254898
F	6.0255719	-5.0857333	-2.7921288
C	5.1306969	-4.1838056	-2.4166281

F	6.3813300	-3.6479602	-0.4719888
C	5.3124117	-3.4552600	-1.2247749
C	4.3401174	-2.5043639	-0.8622573
F	4.4893667	-1.7938680	0.2453906
F	2.3363524	-1.3930478	-1.3133251
C	3.2191920	-2.3073978	-1.6972370
C	2.0314597	-2.5475434	-5.4300878
F	4.3962814	-2.3017428	-5.2399949
C	3.3113946	-2.4112124	-5.9880642
F	4.7578074	-2.2646895	-7.8604118
C	3.5248005	-2.3589794	-7.3851619
F	2.6183095	-2.3957803	-9.5786301
C	2.4302645	-2.4116582	-8.2692435
F	0.0725235	-2.4564295	-8.5298108
C	1.1296623	-2.4542481	-7.7308715
F	-0.2764372	-2.4276232	-5.8682114
C	0.9655891	-2.4892800	-6.3329621
B	1.4024951	1.4149505	-3.9142244
C	0.1852205	1.4728600	-5.0199330
F	1.7043936	0.7456024	-6.6868637
C	0.4823080	1.1386843	-6.3514772
F	-0.1276191	0.8974414	-8.6326699
C	-0.4705702	1.1915309	-7.3871241
F	-2.7134164	1.6168388	-8.0464670
C	-1.7997289	1.5639275	-7.0903807
F	-3.3805719	2.1942836	-5.4434429
C	-2.1362123	1.8644107	-5.7602190
F	-1.5447597	2.0807504	-3.5195252
C	-1.1398550	1.8098597	-4.7566158
C	2.7647989	2.0789989	-4.5388261
F	4.1435356	0.5080977	-3.4266718
C	4.0223650	1.5476584	-4.2414095
F	6.3907722	1.5634553	-4.4334873
C	5.2196210	2.0996209	-4.7486307
F	6.2800155	3.7860118	-6.0404182
C	5.1671821	3.2436256	-5.5679520
F	3.8347014	4.9085716	-6.6059323
C	3.9132608	3.8147424	-5.8592988
F	1.5914964	3.8237069	-5.6342500
C	2.7427222	3.2215118	-5.3422625

Ar = Tipp, at PBEh-3c level (Fig. S90)

CLOSE

C	2.7603587	3.2241552	0.5421407
C	2.4038845	2.3388453	-0.4656548
C	1.8604856	2.9348815	-1.5941411
C	1.6155228	4.2903083	-1.7111202
C	1.9543856	5.1252809	-0.6634925
C	2.5372990	4.5881231	0.4685660
B	2.6628102	0.7519331	-0.4350493
C	3.4917309	0.2746234	-1.7428891
C	3.0812697	-0.5470379	-2.7753887
C	3.8899712	-0.8912639	-3.8450710
C	5.1739198	-0.3886151	-3.9137748
C	5.6295743	0.4488321	-2.9117912
C	4.7890506	0.7567071	-1.8584153
F	1.8453203	-1.0568424	-2.7796536
F	3.4405739	-1.6957888	-4.8021953
F	5.9622232	-0.7048032	-4.9313674
F	6.8611601	0.9410972	-2.9698283
F	5.2693580	1.5705895	-0.9153674
F	1.5496385	2.1772998	-2.6466656
F	1.0689750	4.7916679	-2.8128801
F	1.7315909	6.4282241	-0.7466894
F	2.8827695	5.3846588	1.4737985
F	3.3521601	2.7913968	1.6575438
P	1.0388226	-0.5361459	-0.0159231
C	2.4999108	-1.3599258	0.7331005
C	3.2355523	-0.0227998	0.8935701
C	0.0257884	0.0085518	1.4496374
C	0.2180932	1.4512007	1.9359932
B	-0.7461091	1.7417956	3.1430490
C	-2.2829806	1.8838103	2.8431246
C	-3.1872353	0.9769610	3.3655720
C	-4.5371346	1.0220160	3.0727515
C	-5.0081618	2.0220520	2.2388824
C	-4.1351540	2.9573396	1.7093654
C	-2.7912351	2.8683900	2.0151900
F	-2.7389842	0.0029942	4.1590289

F	-5.3767170	0.1275224	3.5727267
F	-6.2957941	2.0827185	1.9464494
F	-4.5909684	3.9101127	0.9097967
F	-1.9654554	3.7792090	1.5018209
C	-0.2392543	1.8542411	4.6148055
C	0.8507313	1.1300001	5.0884938
C	1.3240571	1.2461363	6.3796295
C	0.7075932	2.1338416	7.2449168
C	-0.3768441	2.8807957	6.8189447
C	-0.8377487	2.7209303	5.5261728
F	1.4590201	0.2480454	4.3003662
F	-1.8701543	3.4669069	5.1534776
F	-0.9560001	3.7343320	7.6488410
F	1.1545833	2.2663246	8.4783347
F	2.3514936	0.5221194	6.7960928
C	-0.1915219	-1.5458814	-0.9424275
C	-1.1473979	-0.8415993	-1.7019008
C	-2.0850508	-1.5495958	-2.4382217
C	-2.1076582	-2.9350818	-2.4688515
C	-1.1756308	-3.6064895	-1.6989392
C	-0.2276034	-2.9510169	-0.9192840
C	-1.2404076	0.6709381	-1.7440559
C	-2.4953996	1.1600866	-1.0185213
C	-3.1109536	-3.6855196	-3.3101663
C	-2.8999922	-3.4051406	-4.7979542
C	0.7142863	-3.8239178	-0.1147121
C	-0.0066186	-4.9384737	0.6447850
H	-0.3743954	1.1009960	-1.2311400
C	-1.2216808	1.2019018	-3.1780174
H	-2.9406849	-4.7544024	-3.1461759
C	-4.5458131	-3.3753887	-2.8861022
C	1.8114805	-4.3965772	-1.0124350
H	1.1867355	-3.2144718	0.6492322
H	4.3198044	-0.1394943	0.9153199
H	2.9569500	0.4282600	1.8438985
H	2.3295470	-1.9291468	1.6471166
H	2.9915254	-2.0024443	0.0024630
H	-2.8172178	-0.9982091	-3.0165959
H	-1.1921984	-4.6897845	-1.7022221
H	0.2311103	-0.7144948	2.2420103
H	-1.0133480	-0.1536116	1.1617357
H	-0.0299228	2.1266086	1.1125880
H	1.2556289	1.6384189	2.1928750
H	-4.7049311	-3.5908587	-1.8295650
H	-4.7950251	-2.3259307	-3.0517678
H	-5.2544634	-3.9727181	-3.4611834
H	-1.8833642	-3.6459715	-5.1077569
H	-3.5864651	-3.9982074	-5.4037480
H	-3.0770166	-2.3546966	-5.0351692
H	2.5445504	-4.9537856	-0.4270512
H	1.3912216	-5.0782968	-1.7539013
H	2.3366527	-3.6138265	-1.5595956
H	0.6924087	-5.4404598	1.3149317
H	-0.8255797	-4.5466885	1.2481185
H	-0.4152689	-5.7029120	-0.0162771
H	-2.1380856	0.9396481	-3.7082246
H	-1.1487642	2.2892735	-3.1835426
H	-0.3842766	0.8001630	-3.7439964
H	-2.5183335	2.2501753	-0.9846097
H	-3.3996649	0.8277009	-1.5304084
H	-2.5509142	0.7871745	0.0046205

OPEN

C	1.6790135	2.5225296	-3.0062748
C	2.1926455	2.6542607	-1.7330034
C	3.2001479	3.5860548	-1.5683194
C	3.6863535	4.3597510	-2.6054816
C	3.1467925	4.1958825	-3.8693059
C	2.1388011	3.2702184	-4.0738086
B	1.7004596	1.8961073	-0.4295504
C	2.6292667	0.7882925	0.1621862
C	2.7786527	0.5725613	1.5322533
C	3.6623528	-0.3559851	2.0458390
C	4.4251831	-1.1202656	1.1820912
C	4.3049732	-0.9513624	-0.1853072
C	3.4257410	-0.0018744	-0.6672833
F	2.1136038	1.3059132	2.4127802
F	3.7855726	-0.5298832	3.3557166
F	5.2607292	-2.0206521	1.6648603

F	5.0344427	-1.6892465	-1.0078568
F	3.3574444	0.1359476	-1.9838574
F	3.7157900	3.7543236	-0.3469415
F	4.6479171	5.2488350	-2.4039472
F	3.5917814	4.9244079	-4.8801070
F	1.6240091	3.1093270	-5.2865100
F	0.7136534	1.6324586	-3.2381318
C	0.3941589	2.4431062	0.2548836
C	-0.9219845	2.0020633	-0.4088668
P	-1.2391227	0.1736127	-0.2460609
C	-0.9987056	-0.1703108	1.5533797
C	-0.1567669	-1.2490649	1.8993086
C	0.2314993	-1.4153097	3.2237234
C	-0.2101659	-0.5709244	4.2315775
C	-1.1257374	0.4116358	3.8894565
C	-1.5438492	0.6215158	2.5815533
C	0.2244668	-2.3061460	0.8783491
C	-1.0112462	-3.1595224	0.5745050
C	0.2596396	-0.6779769	5.6644869
H	-0.6215353	-0.5432525	6.3017657
C	-2.6425839	1.6465462	2.3699117
C	-3.9307532	1.1890681	3.0584908
C	-3.0717407	0.0066917	-0.5637885
C	-3.3241616	-0.8917282	-1.7920753
B	-2.1092465	-0.9756399	-2.8210486
C	-1.2704701	-2.3168387	-2.8507861
C	0.1043408	-2.3568007	-2.6857136
C	0.8153556	-3.5396156	-2.6409463
C	0.1471143	-4.7392891	-2.8136186
C	-1.2199575	-4.7413699	-3.0240460
C	-1.8993061	-3.5367242	-3.0317757
F	0.7899699	-1.2245102	-2.5630251
F	2.1276097	-3.5379617	-2.4372809
F	0.8181162	-5.8802543	-2.7897228
F	-1.8592979	-5.8875346	-3.2178030
F	-3.2128879	-3.5663208	-3.2671747
C	-1.8382437	0.1061460	-3.9297390
C	-1.0627889	-0.1573075	-5.0628486
C	-0.8235602	0.7750767	-6.0540366
C	-1.3668016	2.0404158	-5.9427653
C	-2.1546243	2.3510305	-4.8507349
C	-2.3859467	1.3881561	-3.8896860
F	-0.5325025	-1.3559955	-5.2651751
F	-3.1716676	1.7575328	-2.8829262
F	-2.6873599	3.5609009	-4.7408766
F	-1.1530130	2.9412441	-6.8811377
F	-0.0890889	0.4641607	-7.1115821
H	0.5265475	-1.7980885	-0.0398274
C	1.3670511	-3.2283527	1.2907296
C	0.8824117	-2.0223583	6.0182349
C	1.2352924	0.4613272	5.9742927
C	-2.2494042	3.0491040	2.8331172
H	-2.8734187	1.7059446	1.3089058
H	0.3417538	2.2017474	1.3129592
H	0.4336706	3.5362087	0.1840939
H	-0.9046758	2.2093926	-1.4781249
H	-1.7403292	2.5990698	-0.0040778
H	0.8881465	-2.2352477	3.4768415
H	-1.5356868	1.0324141	4.6797818
H	-3.5140193	0.9944153	-0.6864075
H	-3.5437639	-0.4277361	0.3183751
H	-4.2417275	-0.5762986	-2.2975284
H	-3.5251919	-1.8983799	-1.4241774
H	1.5459494	0.4325866	7.0198003
H	2.1285070	0.3837683	5.3544937
H	0.7879520	1.4373741	5.7856321
H	1.1068006	-2.0630725	7.0842952
H	0.2157844	-2.8543599	5.7888715
H	1.8226700	-2.1843661	5.4878050
H	-3.0467555	3.7610775	2.6147088
H	-2.0690207	3.0849329	3.9083617
H	-1.3439723	3.4013426	2.3389187
H	-4.7466674	1.8793359	2.8383159
H	-4.2297086	0.1966271	2.7205064
H	-3.8184378	1.1464337	4.1425487
H	1.6623807	-3.8468309	0.4429343
H	2.2507037	-2.6832810	1.6177218
H	1.0794959	-3.9067221	2.0957497
H	-0.7750653	-3.9545418	-0.1339470

H	-1.3833166	-3.6272085	1.4877828
H	-1.8264373	-2.5723642	0.1521209

ADDUCT

C	0.8714694	3.0353574	-3.0365957
C	0.8663840	3.2234508	-1.6641411
C	1.3108094	4.4683367	-1.2478861
C	1.7907610	5.4323556	-2.1188660
C	1.8229729	5.1721706	-3.4750391
C	1.3499882	3.9609675	-3.9428481
B	0.2982495	2.0744667	-0.6616452
O	1.0072828	0.7254331	-1.2015593
C	0.6149477	-0.4333608	-1.2485487
P	-1.1930526	-0.7628512	-0.7850101
C	-1.8431131	0.7148151	-1.6179989
C	-1.3296169	1.9361452	-0.8504846
F	1.2753458	4.8052131	0.0431594
F	2.2162878	6.6037239	-1.6623255
F	2.2825126	6.0819477	-4.3205886
F	1.3399289	3.7055271	-5.2455079
F	0.3647392	1.9063786	-3.5543327
C	-1.3546019	-2.3210556	-1.6902116
C	0.0756043	-2.8784915	-1.8236083
B	1.2101144	-1.7227677	-2.1021192
C	2.6667310	-2.2889206	-1.6533850
C	3.4848822	-1.7936434	-0.6556130
C	4.7015480	-2.3570606	-0.3123014
C	5.1566376	-3.4599651	-1.0079310
C	4.3865944	-3.9791498	-2.0321417
C	3.1719722	-3.3894725	-2.3306894
F	3.1167040	-0.7123245	0.0390757
F	5.4298652	-1.8455708	0.6741945
F	6.3196263	-4.0154982	-0.6978215
F	4.8152845	-5.0353113	-2.7132604
F	2.4745787	-3.9261735	-3.3353844
C	1.3273776	-1.1114356	-3.6224505
C	2.3532437	-0.2166688	-3.9100408
C	2.5600912	0.3412040	-5.1562966
C	1.7127688	0.0070119	-6.1953264
C	0.6831885	-0.8825227	-5.9657784
C	0.5130506	-1.4190701	-4.7001949
F	3.1951993	0.1595806	-2.9491377
F	-0.5063034	-2.2788191	-4.5724236
F	-0.1343387	-1.2200609	-6.9579292
F	1.8871879	0.5318044	-7.3979993
F	3.5522555	1.1978834	-5.3611024
C	-1.6909678	-0.8113209	0.9509084
C	-0.7656762	-1.0293304	1.9854066
C	-1.1670441	-0.7694383	3.2901655
C	-2.4551418	-0.3634630	3.6028816
C	-3.3858610	-0.3210873	2.5720264
C	-3.0432416	-0.5509334	1.2517091
C	0.5596593	-1.7173792	1.7542650
H	0.8869714	-1.5393703	0.7261641
C	-2.8960909	-0.0088477	5.0043442
H	-3.8040775	-0.5890824	5.2066057
C	-4.1660605	-0.6295283	0.2351648
C	-5.0989378	-1.7952551	0.5747322
C	1.6893315	-1.2593369	2.6667474
C	0.3448496	-3.2285536	1.8955336
C	-1.8775621	-0.3494619	6.0840119
C	-3.2687363	1.4759600	5.0701863
C	-4.9432278	0.6798378	0.1128931
H	-3.7534101	-0.8711834	-0.7479937
C	0.9267403	2.1801294	0.8374828
C	0.2335077	2.2670795	2.0351964
C	0.8597101	2.3981695	3.2646067
C	2.2355196	2.4594751	3.3340803
C	2.9738403	2.3806289	2.1678839
C	2.3132134	2.2404948	0.9625703
F	-1.1013123	2.2503237	2.0784074
F	0.1435965	2.4638424	4.3860107
F	2.8402430	2.5892456	4.5052038
F	4.2969127	2.4483195	2.2176090
F	3.0751871	2.2090048	-0.1263537
H	-1.8629680	1.9787548	0.0937666
H	-1.6661943	2.8128754	-1.4085110
H	-1.4650506	0.6685982	-2.6413341
H	-2.9278889	0.7038989	-1.6908186

H	-0.4503871	-0.9089568	4.0865144
H	-4.4211629	-0.1093082	2.8154505
H	-1.7994505	-2.1052403	-2.6612377
H	-2.0348632	-2.9876546	-1.1574699
H	0.0660400	-3.6718984	-2.5677692
H	0.3325415	-3.3724671	-0.8815668
H	-3.6280484	1.7349532	6.0666323
H	-2.4062617	2.1024278	4.8481936
H	-4.0544526	1.7323903	4.3595785
H	-2.2909689	-0.1289964	7.0678100
H	-1.6039497	-1.4052399	6.0751188
H	-0.9669859	0.2426915	5.9785022
H	-5.6878364	0.6124677	-0.6810739
H	-5.4749248	0.9115762	1.0363947
H	-4.2914991	1.5264979	-0.1029094
H	-5.8587780	-1.9080792	-0.1993645
H	-4.5509500	-2.7347332	0.6484955
H	-5.6145688	-1.6380762	1.5221783
H	2.6163398	-1.7630516	2.3993857
H	1.8609567	-0.1888356	2.5932271
H	1.4918638	-1.5023001	3.7111075
H	1.2568888	-3.7671488	1.6352251
H	0.0828709	-3.4853386	2.9230470
H	-0.4574050	-3.5923251	1.2530725

TS

C	-3.5628278	1.3953167	-0.3900247
C	-2.4634683	1.0488669	0.3793696
C	-1.7668788	2.1185828	0.9254365
C	-2.0853441	3.4416091	0.6951835
C	-3.1662670	3.7373109	-0.1128016
C	-3.9137224	2.7092139	-0.6525878
B	-1.9692339	-0.4032072	0.8412705
C	-2.1424518	-1.6208367	-0.6857241
C	-1.6917841	-0.9323138	-1.9707580
P	-0.0519085	-0.4608962	-1.3245769
C	-0.7003675	-1.0502466	0.3186302
O	-0.0392809	-1.9574214	0.9652840
B	0.8526052	-3.0606486	0.4129806
C	0.2702869	-4.5294273	0.8637626
C	-0.9734244	-4.7986602	1.4101403
C	-1.3810160	-6.0652009	1.7965287
C	-0.5274291	-7.1371677	1.6309123
C	0.7201644	-6.9249587	1.0759310
C	1.0830670	-5.6432203	0.7062242
F	-1.8772254	-3.8266828	1.5897095
F	-2.5878873	-6.2556859	2.3206371
F	-0.9025908	-8.3570304	1.9927126
F	1.5515966	-7.9467685	0.9013603
F	2.3004036	-5.5035167	0.1687455
F	-0.7486025	1.8684879	1.7417497
F	-1.3755701	4.4208071	1.2403134
F	-3.4864413	4.9974337	-0.3645839
F	-4.9537343	2.9857735	-1.4286899
F	-4.3320059	0.4592940	-0.9516446
C	2.2667192	-2.8243259	1.2225057
C	2.2213669	-2.7980635	2.6127328
C	3.3282761	-2.5804892	3.4134661
C	4.5630805	-2.3860549	2.8227381
C	4.6650741	-2.4309211	1.4475835
C	3.5300950	-2.6565523	0.6856890
F	1.0705885	-3.0152983	3.2458975
F	3.7278592	-2.6926550	-0.6405995
F	5.8467310	-2.2440431	0.8639333
F	5.6372428	-2.1630649	3.5684264
F	3.2209383	-2.5608344	4.7369763
C	0.8959421	-3.0239935	-1.2236457
C	1.1760252	-1.6651629	-1.8740422
C	0.4487486	1.2644674	-1.4229237
C	1.3062280	1.7650125	-0.4209806
C	1.4344918	3.1379716	-0.3073109
C	0.7906014	4.0201398	-1.1682987
C	0.0626203	3.4896897	-2.2233784
C	-0.1047540	2.1207356	-2.3894958
C	2.2134673	0.8596888	0.3870327
H	1.8011812	-0.1527009	0.4209192
C	1.0022936	5.5052886	-0.9962126
C	2.3334465	5.9080319	-1.6378121
C	-0.7206469	1.6303618	-3.6860813

C	-2.1235477	2.1716643	-3.9458044
C	-2.5472976	-0.9599506	2.2065453
C	-1.7532834	-1.0005345	3.3431963
C	-2.2144697	-1.4879129	4.5524085
C	-3.5134865	-1.9525358	4.6461887
C	-4.3425393	-1.9113358	3.5404070
C	-3.8478221	-1.4079080	2.3536163
F	-0.5148818	-0.5242785	3.3116300
F	-1.4323531	-1.5013339	5.6223192
F	-3.9687488	-2.4203806	5.7969891
F	-5.5934787	-2.3445458	3.6298361
F	-4.6801491	-1.3694769	1.3076936
C	3.5578552	0.7964114	-0.3511790
C	2.4426183	1.2629268	1.8396939
C	-0.1356542	6.3636423	-1.5354863
H	1.0787197	5.6926915	0.0792811
C	0.2188790	1.9723202	-4.8472985
H	-0.7805092	0.5397787	-3.6707558
H	1.6424376	-3.7242442	-1.5974095
H	-0.0519587	-3.4120958	-1.6150527
H	1.1379509	-1.6929018	-2.9682260
H	2.1601520	-1.2848020	-1.6095668
H	2.0666854	3.5443616	0.4734678
H	-0.3712797	4.1586057	-2.9560170
H	-1.6212942	-1.6001225	-2.8299483
H	-2.3112145	-0.0870532	-2.2495662
H	-1.9253769	-2.6832075	-0.6539261
H	-3.2101352	-1.5371230	-0.4836757
H	0.0276362	7.4075288	-1.2678035
H	-0.1993735	6.3241771	-2.6243926
H	-1.1012804	6.0674441	-1.1287345
H	2.5352009	6.9674198	-1.4751816
H	3.1695508	5.3425246	-1.2258107
H	2.3144726	5.7326948	-2.7148721
H	-0.1579740	1.5457498	-5.7776284
H	0.3056853	3.0498784	-4.9888105
H	1.2214259	1.5797997	-4.6771355
H	-2.5186616	1.7714046	-4.8798196
H	-2.8235086	1.9105135	-3.1529714
H	-2.1273750	3.2585052	-4.0329435
H	4.2413216	0.1066485	0.1424733
H	3.4497081	0.4806190	-1.3891947
H	4.0306140	1.7795866	-0.3616027
H	3.0606046	0.5065049	2.3238485
H	2.9761239	2.2105312	1.9241124

A

C	-2.1780368	4.5765372	-2.2306963
C	-1.9771706	3.8787452	-1.0471846
C	-2.7474246	4.3229198	0.0163573
C	-3.6910223	5.3287674	-0.0824828
C	-3.8753961	5.9683625	-1.2920919
C	-3.1036542	5.5952177	-2.3742674
B	-0.8796321	2.6778756	-0.9437576
O	-1.3913728	1.6159884	0.1250856
C	-0.7025750	1.0476207	1.2171349
P	1.1319205	0.8652057	1.1284508
C	1.6174164	2.2482465	0.0295857
C	0.4901786	3.2418273	-0.2868179
F	-2.6062618	3.7843927	1.2343129
F	-4.4103709	5.6881602	0.9751279
F	-4.7707885	6.9383998	-1.4098742
F	-3.2515896	6.2169424	-3.5386760
F	-1.4451837	4.3009997	-3.3123725
C	0.9458502	1.5010797	2.8265113
C	-0.5246990	1.8624171	2.5191573
C	2.0847188	-0.6643201	0.8617438
C	1.4988236	-1.9095302	1.1971002
C	2.0692711	-3.0673073	0.6987750
C	3.2105789	-3.0541001	-0.0930647
C	3.8438141	-1.8403941	-0.2805125
C	3.3304685	-0.6422703	0.2042572
C	0.3639472	-2.0416701	2.1877434
H	-0.2541550	-1.1472845	2.1487017
C	3.7662173	-4.3245108	-0.6854453
C	4.1815504	-5.3166373	0.4004448
C	4.2445367	0.5638650	0.0993282
H	3.7673951	1.4209302	0.5676570
C	-0.5611886	-3.2303954	1.9328811

C	0.9528134	-2.1494097	3.5997517
C	2.7713055	-4.9528305	-1.6610286
H	4.6638277	-4.0560512	-1.2514026
C	5.5161099	0.3171007	0.9182486
C	4.5907793	0.9359705	-1.3417847
C	-0.7626066	1.8716965	-2.3597152
C	-1.8475621	1.1392174	-2.8161774
C	-1.8317017	0.3608983	-3.9585243
C	-0.6905887	0.3290669	-4.7357550
C	0.3989220	1.0885028	-4.3584866
C	0.3370121	1.8419534	-3.1996799
F	-2.9883649	1.1517985	-2.1253340
F	-2.8923351	-0.3588686	-4.3064018
F	-0.6468245	-0.4101631	-5.8346572
F	1.4971635	1.0887853	-5.1074126
F	1.4316363	2.5699578	-2.9308343
B	-2.0189907	0.3474320	0.6564637
C	-1.9864987	-0.9412999	-0.2923469
C	-3.0525457	-1.8286568	-0.3804411
C	-3.0453136	-2.9458825	-1.1996111
C	-1.9409971	-3.2128974	-1.9846589
C	-0.8579668	-2.3557774	-1.9396229
C	-0.9075019	-1.2572544	-1.1050160
F	-4.1582298	-1.6459234	0.3348847
F	-4.0920345	-3.7583745	-1.2378348
F	-1.9204939	-4.2747942	-2.7729670
F	0.2047948	-2.5834067	-2.7018821
F	0.1731943	-0.4666114	-1.1258805
C	-3.3051001	0.5926563	1.5801221
C	-4.3956718	1.3632573	1.2156381
C	-5.4799255	1.5748758	2.0492277
C	-5.5010600	0.9893938	3.3012090
C	-4.4399197	0.2002372	3.7023018
C	-3.3767355	0.0227998	2.8380909
F	-4.4462633	1.9246360	0.0125067
F	-2.3711646	-0.7541427	3.2692382
F	-4.4479713	-0.3708029	4.9023190
F	-6.5316655	1.1807560	4.1112287
F	-6.5007793	2.3250715	1.6569129
H	0.9254218	3.9877959	-0.9542745
H	0.2484457	3.8089460	0.6170903
H	2.4484061	2.7709542	0.5065021
H	2.0054971	1.8035545	-0.8820417
H	1.6065055	-4.0168012	0.9337371
H	4.7855055	-1.8255462	-0.8166204
H	1.6016088	2.3280919	3.0923225
H	1.0498525	0.7156587	3.5719189
H	-0.6464423	2.9291392	2.3554580
H	-1.2149017	1.5713623	3.3081815
H	4.6416898	-6.1990761	-0.0452926
H	4.9006839	-4.8768043	1.0915914
H	3.3242259	-5.6563441	0.9834510
H	2.4782150	-4.2521420	-2.4414417
H	3.2093188	-5.8285961	-2.1409419
H	1.8626129	-5.2787338	-1.1519242
H	5.2817017	0.0503979	1.9488548
H	6.1225704	-0.4857763	0.4985195
H	6.1308705	1.2179523	0.9345587
H	3.7074838	1.0737143	-1.9644754
H	5.1631010	1.8636723	-1.3673832
H	5.2019347	0.1670412	-1.8157615
H	-1.4495689	-3.1466579	2.5559880
H	-0.8862400	-3.2849660	0.8955758
H	-0.0858671	-4.1793364	2.1856545
H	1.5196598	-3.0750473	3.7097430
H	1.6336857	-1.3300248	3.8314897
H	0.1571111	-2.1514249	4.3457197

B

C	-3.6980269	0.0597036	-0.3620050
C	-2.6465620	0.1689181	0.5290250
C	-2.4543966	1.4213604	1.0909990
C	-3.2030388	2.5287037	0.7540043
C	-4.2089343	2.3884892	-0.1841040
C	-4.4675704	1.1481784	-0.7361784
B	-1.6039640	-0.9288713	0.9928757
C	-1.7240143	-1.5424060	2.4203944
C	-0.7428394	-1.4301802	3.3924951
C	-0.8638148	-2.0418257	4.6253483

C	-1.9914701	-2.7957807	4.9004941
C	-2.9946184	-2.9249196	3.9553665
C	-2.8461570	-2.2863036	2.7415698
F	0.3137298	-0.6578347	3.1867340
F	0.0707011	-1.8938284	5.5504300
F	-2.1188310	-3.3845267	6.0764581
F	-4.0735569	-3.6457677	4.2248021
F	-3.8087789	-2.4223835	1.8264976
F	-1.4888578	1.5626853	1.9972334
F	-2.9676810	3.7086075	1.3077083
F	-4.9228993	3.4399446	-0.5527203
F	-5.4423147	1.0102819	-1.6242927
F	-4.0102218	-1.1183412	-0.9029497
C	-0.4414074	-1.4476300	0.0563762
C	-1.1682229	-2.3814072	-1.0124952
C	-1.1982358	-1.4330573	-2.2231818
P	0.0218651	-0.3421073	-1.3989861
C	-0.1454268	1.4645244	-1.3355598
C	0.5818832	2.1342664	-0.3257616
C	0.3208751	3.4788140	-0.1088215
C	-0.5909168	4.1940852	-0.8677861
C	-1.2284634	3.5332864	-1.9083018
C	-1.0280316	2.1852882	-2.1719436
C	1.7327293	1.5070225	0.4399298
H	1.6398073	0.4174505	0.4427508
C	-0.7959140	5.6648712	-0.5923105
C	0.3557021	6.4660179	-1.2066656
C	-1.6858313	1.5777249	-3.3983771
H	-2.1651375	0.6448667	-3.1110566
C	1.6582700	-0.9380551	-1.9229310
C	1.9060486	-2.3852387	-1.4758111
B	1.7448300	-2.6927571	0.1392263
O	0.6066343	-1.9308521	0.7442612
C	3.0013116	-2.2244304	1.1024248
C	2.8988621	-2.4674130	2.4677911
C	3.8248811	-2.0378644	3.4003257
C	4.9314662	-1.3260278	2.9759893
C	5.0912690	-1.0747935	1.6293073
C	4.1413125	-1.5348742	0.7309452
F	1.8613104	-3.1551462	2.9497384
F	4.3893721	-1.2428981	-0.5556971
F	6.1469518	-0.3829694	1.2040637
F	5.8280757	-0.8902241	3.8521793
F	3.6643581	-2.2970881	4.6935866
C	1.5443880	-4.3199520	0.3321141
C	0.4246959	-4.9573622	0.8425135
C	0.3290853	-6.3317144	0.9914669
C	1.3923609	-7.1331256	0.6275411
C	2.5362047	-6.5461377	0.1204960
C	2.5832698	-5.1709278	-0.0141976
F	-0.6586574	-4.2664124	1.2285175
F	-0.7772447	-6.8841931	1.4824832
F	1.3168495	-8.4515969	0.7615131
F	3.5697590	-7.3045427	-0.2308454
F	3.7196002	-4.6592058	-0.5017206
C	3.0363367	1.8955518	-0.2720539
C	1.8378440	1.9153040	1.9084428
C	-2.1387131	6.2083893	-1.0667594
H	-0.7503379	5.7929893	0.4940743
C	-2.7935785	2.4258328	-4.0165260
C	-0.6272323	1.2624004	-4.4594893
H	2.9061424	-2.6447529	-1.8181164
H	1.2553165	-3.0462537	-2.0569055
H	1.7057119	-0.8176899	-3.0106436
H	2.4088131	-0.2709556	-1.5098882
H	0.8548595	3.9940226	0.6803426
H	-1.9077902	4.0907127	-2.5352235
H	-0.8389620	-1.8499531	-3.1630451
H	-2.1765427	-1.0040063	-2.3927878
H	-0.5578988	-3.2600969	-1.1971411
H	-2.1521058	-2.7456443	-0.7182249
H	-2.2700560	7.2317892	-0.7156183
H	-2.2062747	6.2375938	-2.1557827
H	-2.9756620	5.6209963	-0.6912510
H	0.2578715	7.5261745	-0.9697039
H	1.3252392	6.1305000	-0.8384021
H	0.3619154	6.3652904	-2.2932937
H	-3.2809564	1.8588489	-4.8092939
H	-3.5626291	2.6972083	-3.2929899

H	-2.4120591	3.3421391	-4.4690979
H	-1.0797761	0.7812569	-5.3272027
H	-0.1405405	2.1780139	-4.7980167
H	0.1579993	0.6009479	-4.0907606
H	3.8912055	1.3920860	0.1784092
H	3.0271548	1.6612386	-1.3363373
H	3.2022788	2.9706462	-0.1864150
H	2.6103958	1.3148577	2.3885261
H	2.1316286	2.9593779	2.0223044
H	0.9139800	1.7630672	2.4555939

C

C	-2.6439722	-0.5736630	0.6618079
C	-1.5846638	-0.0456365	-0.0955891
C	-1.1820252	-0.7199790	-1.2706671
C	-1.7186732	-1.9651609	-1.5559242
C	-2.6658283	-2.5678055	-0.7410852
C	-3.1355134	-1.8344160	0.3325718
P	-0.4892845	1.3290745	0.4597325
C	-0.7725274	1.9508616	2.1786969
C	-1.5205831	3.2739166	2.1136623
C	-1.1151363	4.0431089	0.8539967
O	-1.4409288	5.1911651	0.6962128
C	-0.2678208	-0.1070142	-2.3102020
H	0.1167044	0.8376474	-1.9322245
C	-3.2048269	-3.9430574	-1.0474116
H	-3.8932777	-4.2115714	-0.2399476
C	-3.3626072	0.1546819	1.7782163
H	-2.9683016	1.1627258	1.8513936
B	-0.3096995	3.2042952	-0.3154321
C	-1.1113556	3.4991121	-1.6751116
C	-0.6767357	4.3270120	-2.6977653
C	-1.4622905	4.6447975	-3.7941937
C	-2.7520891	4.1586717	-3.8776701
C	-3.2504719	3.3778470	-2.8510297
C	-2.4291172	3.0852729	-1.7819152
F	0.5256442	4.8966106	-2.6519690
F	-0.9914446	5.4282646	-4.7569588
F	-3.5130214	4.4557000	-4.9213660
F	-4.5005316	2.9292349	-2.8983990
F	-2.9695091	2.3836983	-0.7727797
C	1.2911905	3.4643591	-0.2931395
C	2.1333548	3.0630070	-1.3225224
C	3.4998772	3.2617829	-1.3221174
C	4.0930900	3.8826551	-0.2380466
C	3.3063352	4.2857209	0.8218327
C	1.9388494	4.0677157	0.7753395
F	1.6308641	2.4361932	-2.3900565
F	1.2501079	4.4530130	1.8535304
F	3.8633163	4.8685208	1.8758227
F	5.4023002	4.0783902	-0.2140339
F	4.2458719	2.8595734	-2.3433619
C	1.1061050	0.4343429	0.6930430
C	0.9669247	-0.7409555	1.6769651
B	2.3294854	-1.5188422	1.7558413
C	3.3025146	-1.4304054	2.9703465
C	3.3923376	-0.3013284	3.7802464
C	4.2282211	-0.2280341	4.8760945
C	5.0069006	-1.3257892	5.2022137
C	4.9520118	-2.4722767	4.4288867
C	4.1177543	-2.5026804	3.3282911
F	2.6801161	0.7840252	3.4920769
F	4.2912525	0.8694415	5.6128183
F	5.8010455	-1.2794028	6.2528995
F	5.6923117	-3.5209117	4.7512209
F	4.0838802	-3.6284457	2.6271554
C	2.7185488	-2.3996501	0.5132240
C	1.9530968	-3.4765867	0.1037213
C	2.2721796	-4.2268809	-1.0107954
C	3.3871588	-3.8824282	-1.7564670
C	4.1751500	-2.8079072	-1.3798540
C	3.8310567	-2.0929204	-0.2484269
F	0.8927568	-3.8337562	0.8277063
F	1.5310397	-5.2649241	-1.3713670
F	3.7020968	-4.5854103	-2.8302570
F	5.2368181	-2.4813511	-2.0995638
F	4.5850515	-1.0515590	0.1037799
C	0.9275289	-0.9843968	-2.6783580
C	-1.0703588	0.2353767	-3.5682616

C	-4.8470304	0.3276024	1.4456837
C	-3.1808623	-0.5347302	3.1298710
C	-3.9986587	-3.9462508	-2.3536786
C	-2.0899473	-4.9874802	-1.0745389
H	0.6206336	-0.3853390	2.6473796
H	0.1997075	-1.4209761	1.2961611
H	1.4670772	0.0960072	-0.2768391
H	1.8270903	1.1706592	1.0494295
H	-3.9303655	-2.2572504	0.9367119
H	-1.3930331	-2.4760886	-2.4549472
H	0.2406360	2.1267204	2.5492817
H	-1.2142024	1.2199690	2.8490306
H	-1.3371999	3.8795640	3.0019341
H	-2.6028103	3.1206224	2.0642973
H	-2.5042264	-5.9851080	-1.2251341
H	-1.3848015	-4.8024889	-1.8859317
H	-1.5254323	-4.9956393	-0.1429948
H	-4.4338295	-4.9293762	-2.5371377
H	-4.8097872	-3.2185743	-2.3298250
H	-3.3631139	-3.7051936	-3.2073185
H	0.6171529	-1.8962045	-3.1904702
H	1.5960524	-0.4461125	-3.3501605
H	1.5056435	-1.2861322	-1.8050679
H	-0.4535543	0.8001608	-4.2679380
H	-1.4141692	-0.6665294	-4.0774626
H	-1.9476394	0.8352365	-3.3344627
H	-4.9795234	0.8188704	0.4828522
H	-5.3773315	-0.6241574	1.4125935
H	-5.3299396	0.9430065	2.2060403
H	-3.6528081	-1.5179248	3.1421458
H	-2.1282179	-0.6839908	3.3769432
H	-3.6337633	0.0544590	3.9284754

DIMER

P	-0.6557209	2.8824526	0.8660001
B	-0.8662330	3.2557535	-1.1597901
B	0.9671464	-0.0807536	3.5467876
O	-1.2750020	1.2076301	-2.5381639
C	-2.3221720	2.0798157	0.8575335
H	-2.5214945	1.5076708	1.7587342
H	-3.1018256	2.8315568	0.7716414
C	-2.2917562	1.1597760	-0.3595841
H	-1.7928905	0.2251682	-0.0993902
H	-3.2919888	0.8698569	-0.6874656
C	-1.5088021	1.7956364	-1.4741193
C	0.4141728	1.5136429	1.5008937
H	0.4357111	0.7362967	0.7349183
H	1.4355575	1.8832800	1.5631995
C	-0.0601081	0.9808508	2.8504963
H	-1.0635567	0.5713875	2.7507009
H	-0.1694148	1.8204156	3.5425705
C	-0.4468193	4.3342275	1.9566751
C	0.7573029	5.0594682	1.8135709
C	0.8893144	6.2841281	2.4503132
H	1.8173697	6.8269490	2.3334259
C	-0.1296576	6.8372764	3.2068032
C	-1.2671766	6.0700170	3.4025523
H	-2.0448975	6.4600154	4.0495063
C	-1.4413051	4.8176926	2.8313796
C	1.9740948	4.5336356	1.0785679
H	1.6825237	3.6847293	0.4627885
C	2.9899167	4.0260222	2.1063825
H	3.4061894	4.8542369	2.6814063
H	3.8169562	3.5147258	1.6118284
H	2.5386560	3.3356527	2.8185435
C	2.6451154	5.5280909	0.1332163
H	1.9725513	5.8847523	-0.6426578
H	3.4910459	5.0444815	-0.3564160
H	3.0417869	6.3956880	0.6608949
C	-0.0575611	8.2261954	3.7932765
H	-0.5571861	8.1914174	4.7671251
C	1.3607930	8.7349031	4.0192064
H	1.9503857	8.0440219	4.6225181
H	1.3357874	9.6912840	4.5412177
H	1.8890371	8.9009704	3.0787198
C	-0.8433668	9.1904325	2.8989683
H	-0.4003252	9.2439348	1.9034538
H	-0.8435544	10.1957578	3.3221184
H	-1.8805914	8.8773461	2.7795084

C	-2.6797559	4.0546359	3.2536244
H	-2.5975370	3.0319393	2.9030756
C	-2.7744946	3.9367311	4.7767039
H	-2.9533051	4.8970122	5.2603761
H	-1.8620918	3.5145824	5.1975083
H	-3.6011932	3.2789347	5.0472583
C	-3.9484775	4.6683746	2.6629214
H	-4.1160121	5.6759626	3.0460994
H	-4.8252895	4.0709151	2.9157224
H	-3.8959631	4.7464059	1.5755571
C	-2.0169414	4.4112450	-1.1188142
C	-1.7084406	5.7107792	-0.7392360
C	-2.6401634	6.7272181	-0.6546224
C	-3.9568668	6.4692855	-0.9881551
C	-4.3098790	5.2041358	-1.4159897
C	-3.3406729	4.2185448	-1.4778251
C	0.3943792	3.5836315	-2.0928118
C	0.3618293	4.5221635	-3.1155022
C	1.4524067	4.7973372	-3.9222494
C	2.6352964	4.1099560	-3.7322241
C	2.7034117	3.1356095	-2.7550616
C	1.5852244	2.8864410	-1.9863967
C	2.3318379	0.7027613	3.9797444
C	2.2583100	1.5726579	5.0607792
C	3.3284971	2.3098756	5.5320259
C	4.5554807	2.1890883	4.9069031
C	4.6810938	1.3387343	3.8271126
C	3.5798497	0.6238600	3.3840609
C	0.4043422	-0.9214536	4.8264972
C	-0.8359994	-0.7877907	5.4279955
C	-1.2253593	-1.5186536	6.5380253
C	-0.3501658	-2.4300737	7.0941273
C	0.9016927	-2.5983930	6.5327987
C	1.2490060	-1.8478471	5.4264842
P	0.0549214	-3.0512029	-0.5748875
B	1.4494674	-3.1640159	0.9534639
B	-1.8201958	-0.1428508	-3.1548681
O	1.3924003	-1.2165236	2.5295355
C	-1.2802718	-2.9189568	0.6970570
H	-2.2322662	-2.6016567	0.2811483
H	-1.4392928	-3.8806946	1.1770306
C	-0.7592565	-1.8782882	1.6841010
H	-0.9634137	-0.8780314	1.3007848
H	-1.2707038	-1.9272463	2.6471773
C	0.7301078	-2.0061446	1.8425194
C	-0.2028579	-1.5009964	-1.5517352
H	0.0422828	-0.6625341	-0.8980762
H	0.5421917	-1.4729034	-2.3450802
C	-1.6143651	-1.3929883	-2.1228822
H	-2.3396622	-1.3745404	-1.3102029
H	-1.8410216	-2.3063650	-2.6804512
C	-0.0353978	-4.4722179	-1.7198425
C	1.0905282	-4.6756225	-2.5538012
C	1.1811127	-5.8455658	-3.2860867
H	2.0472831	-5.9959594	-3.9189096
C	-0.9150484	-6.5811042	-2.4664542
H	-1.7044328	-7.3236242	-2.4520050
C	-1.0860684	-5.4076550	-1.7396653
C	2.1740657	-3.6379017	-2.7716785
H	2.0977113	-2.8696206	-2.0030035
C	3.6049357	-4.1669768	-2.6941589
H	3.8292825	-4.6208007	-1.7324407
H	4.3031493	-3.3427833	-2.8440373
H	3.8147443	-4.9022354	-3.4713470
C	1.9415491	-2.9577100	-4.1244228
H	2.1278743	-3.6529623	-4.9442652
H	2.6130256	-2.1072954	-4.2497281
H	0.9166139	-2.6031051	-4.2317802
C	0.2155402	-6.8389912	-3.2182552
C	0.3819232	-8.1449280	-3.9529712
H	-0.4854160	-8.7678952	-3.7129733
C	1.6314818	-8.8847075	-3.4760213
H	2.5418831	-8.3394903	-3.7300399
H	1.6975926	-9.8671249	-3.9447555
H	1.6227713	-9.0255111	-2.3951918
C	0.3987669	-7.9359896	-5.4668273
H	-0.5034795	-7.4305429	-5.8106974
H	0.4663790	-8.8928956	-5.9854609
H	1.2543404	-7.3344064	-5.7784345

C	-2.4287679	-5.2311224	-1.0613684
H	-2.5145590	-4.2063499	-0.7175929
C	-3.5865139	-5.4086556	-2.0467498
H	-3.4661530	-4.7657728	-2.9184112
H	-4.5274951	-5.1413501	-1.5645765
H	-3.6840516	-6.4357475	-2.3977277
C	-2.5774243	-6.1619934	0.1415073
H	-2.5680382	-7.2086209	-0.1661508
H	-3.5194158	-5.9810941	0.6611773
H	-1.7660257	-6.0348145	0.8600652
C	1.1184178	-4.6484116	1.5440068
C	0.4854335	-4.8862564	2.7527040
C	0.2072316	-6.1488860	3.2456495
C	0.5779361	-7.2559780	2.5072661
C	1.2382066	-7.0767263	1.3057896
C	1.5073044	-5.7954272	0.8646263
C	3.0013824	-2.8576124	0.6992467
C	3.4089481	-1.7666187	-0.0487633
C	4.7261984	-1.4432131	-0.2990255
C	5.7199986	-2.2253027	0.2574522
C	5.3718334	-3.2974454	1.0556821
C	4.0351863	-3.5885588	1.2696588
C	-3.3549760	0.2314266	-3.5656983
C	-3.5664291	1.3232394	-4.3997333
C	-4.8125068	1.7152973	-4.8495277
C	-5.9253540	0.9913182	-4.4643764
C	-5.7682325	-0.1058182	-3.6411675
C	-4.5006632	-0.4623031	-3.2118333
C	-0.9521268	-0.4127240	-4.5103191
C	-1.4214711	-1.3670733	-5.4040172
C	-0.7883479	-1.6901257	-6.5896461
C	0.3859015	-1.0420664	-6.9242982
C	0.8996304	-0.0923848	-6.0650347
C	0.2333831	0.1971951	-4.8855634
F	-0.4473398	6.0351630	-0.4787152
F	-2.2812554	7.9444334	-0.2715224
F	-4.8659548	7.4261428	-0.9080978
F	-5.5632954	4.9414698	-1.7585213
F	-3.7489762	3.0192492	-1.9141011
F	-0.7515494	5.2004354	-3.3875557
F	1.3671356	5.7136615	-4.8772170
F	3.6885932	4.3729108	-4.4880632
F	3.8241115	2.4503171	-2.5727863
F	1.6811631	1.8962725	-1.0889179
F	1.0947987	1.7342025	5.7014346
F	3.1890961	3.1295323	6.5673273
F	5.5963110	2.8896255	5.3335962
F	5.8514946	1.2225446	3.2106257
F	3.7850898	-0.1494047	2.3168588
F	-1.7434172	0.0754713	4.9546901
F	-2.4313996	-1.3499888	7.0696038
F	-0.7078451	-3.1391425	8.1533552
F	1.7496270	-3.4740370	7.0558404
F	2.4668143	-2.0528257	4.9244621
F	0.1055113	-3.8574119	3.5222342
F	-0.4081932	-6.2995186	4.4099652
F	0.3149755	-8.4736238	2.9502664
F	1.6211760	-8.1305577	0.5982124
F	2.2200099	-5.6880680	-0.2505217
F	2.4800809	-0.9522736	-0.5675047
F	5.0423932	-0.3952329	-1.0470037
F	6.9932177	-1.9409132	0.0393601
F	6.3196007	-4.0363989	1.6165961
F	3.7834248	-4.6103814	2.0858863
F	-2.5288048	2.0569185	-4.8028838
F	-4.9506323	2.7712976	-5.6406382
F	-7.1311146	1.3480159	-4.8789710
F	-6.8309978	-0.8110700	-3.2686552
F	-4.4389572	-1.5408130	-2.4211810
F	-2.5471266	-2.0351213	-5.1278309
F	-1.2913986	-2.6115482	-7.4024838
F	1.0158979	-1.3377086	-8.0519892
F	2.0359338	0.5261449	-6.3649276
F	0.8205287	1.1006745	-4.0992253

Part 3. Solid State NMR Experiments

Formation of Macrocyclic Ring Systems by Carbonylation of Trifunctional P/B/B Frustrated Lewis Pairs

Robert Knitsch,[c] Hellmut Eckert,[c,d] Michael Ryan Hansen[c]

[c] Institut für Physikalische Chemie and Graduate School of Chemistry, Westfälische Wilhelms-Universität Münster, Corrensstr. 30, 48149 Münster, Germany

[d] Institute of Physics in Sao Carlos, University of Sao Paulo, CEP 369, Sao Carlos SP 13566-590, Brazil

Experimental Procedures

Solid state MAS-NMR studies were conducted on a Bruker Avance III 300 (^{1}H 300.1 MHz, 7.05 T) and a Bruker DSX 500 (^{1}H 500 MHz, 11.74 T) spectrometer using a rotational frequency of 12.5 kHz in 4 mm NMR double and triple resonance probes.
For quantitative analysis the ^{11}B-MAS NMR spectra were measured using short pulses in the range of 0.5-0.7 µs to achieve uniform excitation and a relaxation delay between 10 and 20 s. During acquisition proton decoupling was performed (SWFTPPM15 57 kHz RF-Power).

The ^{31}P-CPMAS NMR spectra were conducted using a 5.9 µs (7.05 T) /4.8 µs (11.74 T) π/2 pulse on ^{1}H (42/53 kHz RF-Power) for excitation followed by 750 µs spinlock pulse ramped on ^{1}H from 90/70 to 100% to a maximum nutation frequency of 42/53 kHz. A recycle delay of 3 s was sufficient. During acquisition proton decoupling was performed (SWFTPPM15 60/53 kHz RF-Power). Additionally SWFTPPM15 decoupling of ^{11}B (45 kHz RF-Power) partially was applied to enhance resolution.

For direct excitation of phosphorous in ^{31}P MAS NMR experiments a 5.2 µs pulse (48 kHz RF-Power) was applied followed by a relaxation delay of 60 s (**18c**) and 800 s (**17b**) respectively. During acquisition proton decoupling was performed (SWFTPPM15 60 kHz RF-Power).

^{11}B{^{31}P} J-resolved NMR spectra were acquired using a ^{11}B excitation pulse of 5 µs and reconversion pulses of 10 µs for boron and 10.4 µs for phosphorous. After evolution a z-filter was utilized. During evolution and acquisition ^{1}H decoupling was performed (SWFTPPM15 42 kHz RF-Power). A relaxation delay of 4 s was sufficient.

For ^{11}B{^{31}P} CP-INEPT experiments the parameters from ^{31}P-CPMAS NMR experiments were used. To selectively excite the central transition soft refocusing pulses of 14 µs (35 kHz RF-Power) were applied on the boron channel. Proton decoupling was performed during evolution and acquisition (SWFTPPM15 60 kHz RF-Power) followed by a relaxation delay of 2 s.

Spectral deconvolution was done with DMFIT software (version 2011)[25]

DFT calculation of NMR parameters were conducted using crystal structures obtained by x-ray diffraction. Chemical shifts calculation were performed with TURBOMOLE (version 6.5)[26-27] using B3LYP[28-29] as functional in combination with a def2-SVP basis set for **18c** and a def2-TZVP basis set[20] for **17c** (less computational effort). Quadrupolar coupling parameters were calculated on a GGA DFT level using GAUSSIAN (version GAUSSIAN09)[30] and the B97-D functional[31]. Here for both molecules the def2-TZVP basis set obtained from EMSL database[32-33] could be used with additional functions for boron from the cc-pCVTZ basis set[34-35] to enhance the accuracy near the nucleus.

Results and Discussion

Table S1: ^{11}B isotropic shift (±0.2 ppm), quadrupolar coupling constant C_Q (±0.1 MHz) and electric field gradient asymmetry parameter η_Q (±0..05) obtained via lineshape deconvolution of $^{11}B\{^1H\}$ MAS NMR spectra of **17b** depicted in figure S98 and ^{11}B spectra extracted from $^{11}B\{^{31}P\}$ CP-INEPT NMR experiments (figure S103)

B_0 field	species	δ_{CS}^{iso}/ppm	C_Q/MHz	η_Q	δ_{CS}^{iso}/ppm calc. [a]	C_Q/MHz calc.[a]	η_Q calc.[a]	rel. Int. /%	bound ^{31}P species [b]
^{11}B MAS NMR 7.05 T (300 MHz)	1	8.2	2.3	0.30	5.8, 4.9	2.32,2.33	0.30	51.3	-
	2	-9.3	1.0	0.20	-11.5	1.03	0.26	25.9	2
	3	-9.5	1.1	0.20	-11.5	1.11	0.25	22.7	1
$^{11}B\{^{31}P\}$ CP-INEPT	2	-9.0	1.0	0.20	-11.5	1.03	0.26	---	2
	3	-9.1	1.1	0.15	-11.5	1.11	0.25	---	1

[a] Calculated shifts assigned to the species in such a way that the trend form high to low shift values is matched. Quadrupolar parameters of ^{11}B were assigned to the respective species.

[b] ^{31}P species listed in table 2 assigned to boron species using $^{11}B\{^{31}P\}$ CP-INEPT NMR data.

Table S2: ^{31}P parameters obtained via line shape deconvolution of $^{31}P\{^1H\}$ CPMAS NMR spectra of **17b** depicted in figure S99-101. Estimated errors: ±0.2 ppm and ±2 Hz for δ_{CS}^{iso} and J, respectively.

method	species	δ_{CS}^{iso}/ppm (calc.)	J(^{31}P-^{11}B) /Hz	J(^{31}P-^{10}B) /Hz	xG/(1-x)L	d(^{31}P-^{11}B) /Hz	d(^{31}P-^{10}B) /Hz	rel. Int. /%
CPMAS 7.05 T ^1H dec.	1	21.8 (28.7)	66	19	0.5	6	12	56
	2	20.8 (24.7)	70	20	0.5	6	12	44
CPMAS 7.05 T ^1H, ^{11}B dec.	1	22.2	---	19	0.0	6	12	56
	2	21.3	---	20	0.0	6	12	44
MAS 7.05 T ^1H dec.	1	21.9	72	20	0.5	6	12	50
	2	20.9	73	20	0.5	6	12	50

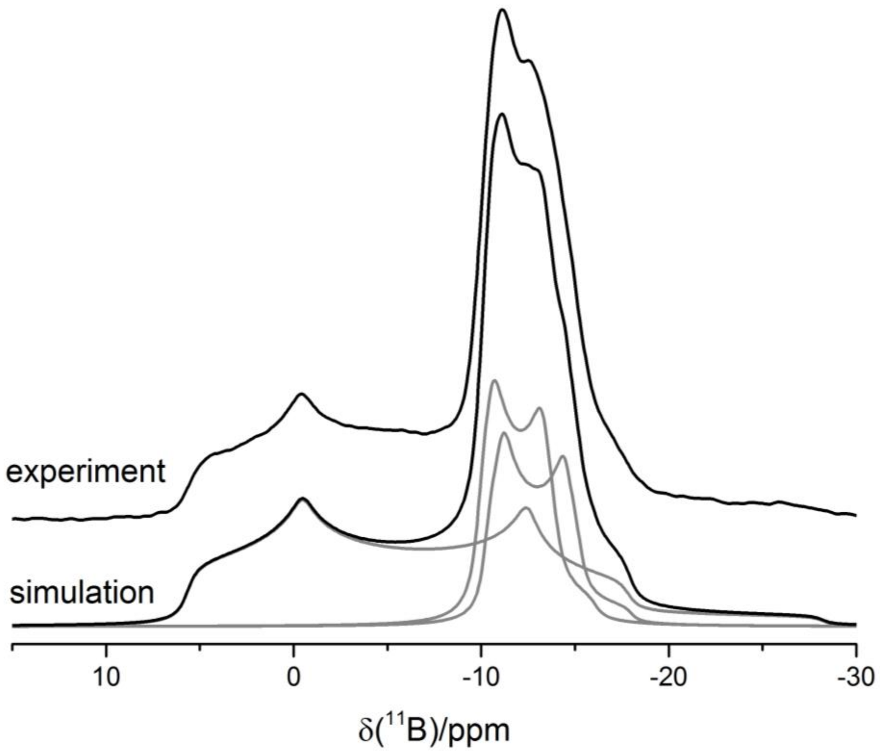

Figure S98. $^{11}B\{^{1}H\}$ MAS NMR spectrum measured of **17b** at a magnetic field strength of 7.05 T and a spinning frequency of 12.5 kHz. ^{1}H decoupling was applied during acquisition using the SWFTPPM15 scheme.

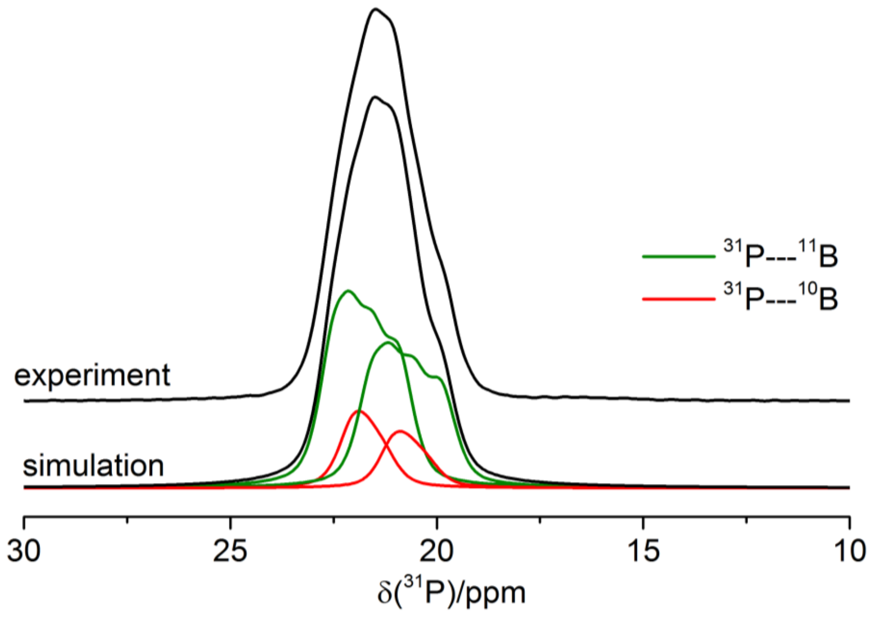

Figure S99. $^{31}P\{^{1}H\}$ CPMAS NMR spectrum of **17b** measured at a magnetic field strength of 7.05 T and a spinning frequency of 12.5 kHz. ^{1}H decoupling was applied during acquisition using the SWFTPPM15 scheme.

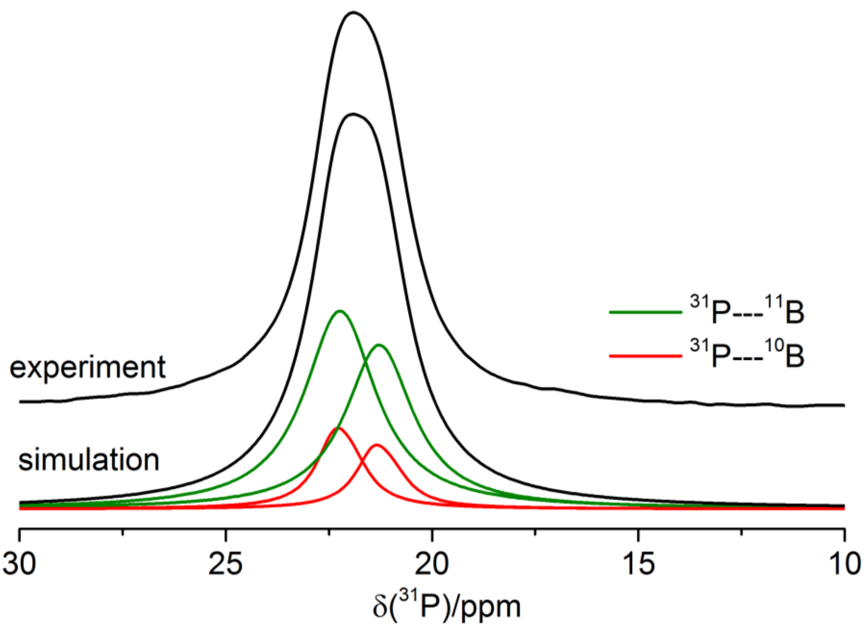

Figure S100. $^{31}P\{^{1}H\}$ CPMAS NMR spectrum of **17b** measured at a magnetic field strength of 7.05 T and a spinning frequency of 12.5 kHz. ^{11}B and ^{1}H decoupling was applied during acquisition using the SWFTPPM15 scheme.

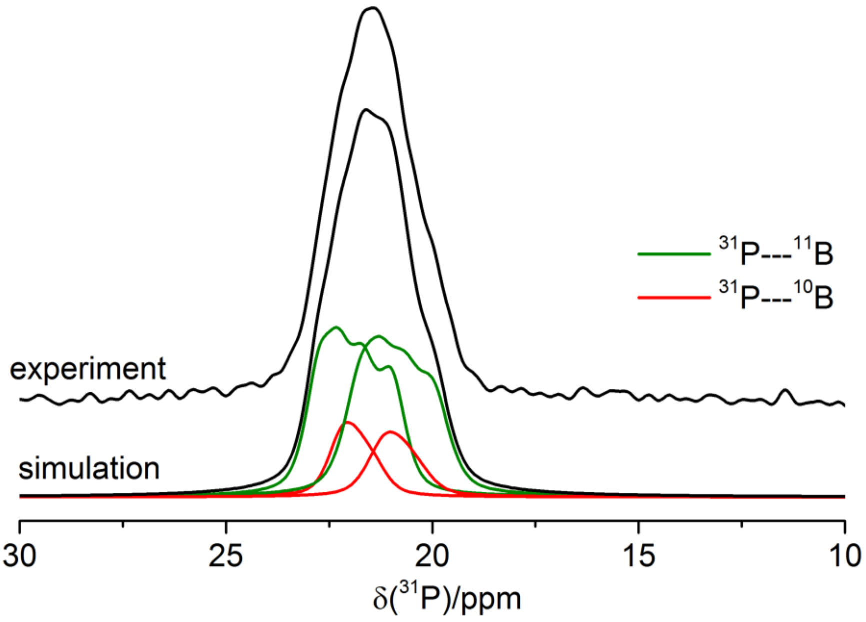

Figure S101. $^{31}P\{^{1}H\}$ MAS NMR spectrum of **17b** measured at a magnetic field strength of 7.05 T and a spinning frequency of 12.5 kHz. ^{11}B and ^{1}H decoupling was applied during acquisition using the SWFTPPM15 scheme.

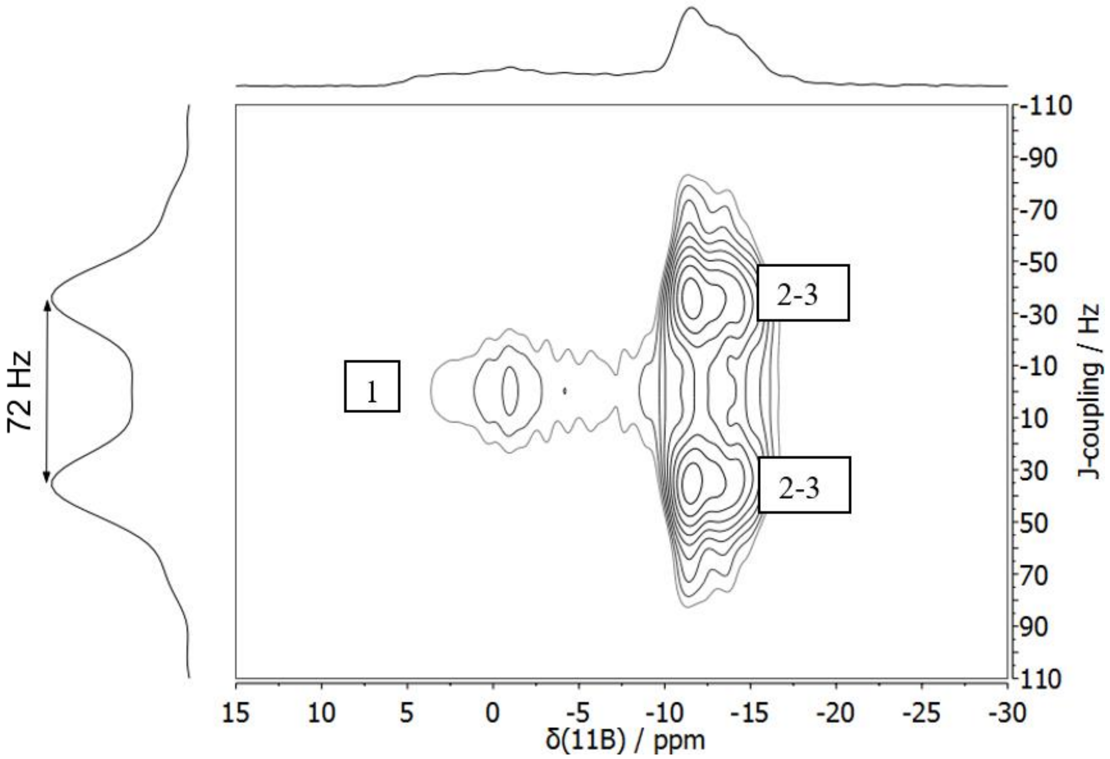

Figure S102. $^{11}B\{^{31}P\}$J-resolved NMR spectrum of **17b** measured at 7.05 T with a spinning frequency of 12.5 kHz. SWFTPPM15 ^{1}H decoupling scheme was used during evolution and acquisition.

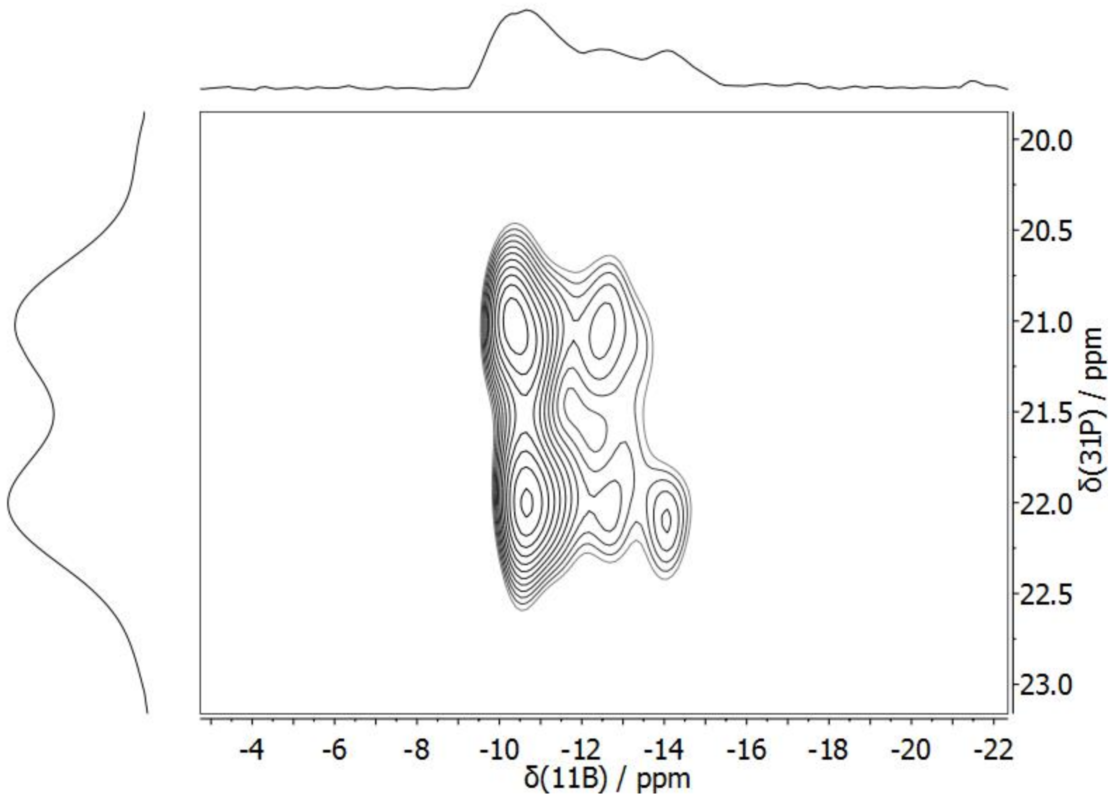

Figure S103. $^{11}B\{^{31}P\}$ CP-INEPT NMR spectrum of **17b** measured at a field strength of 7.05 T and a spinning speed of 12.5 kHz. Soft pulses on boron were applied to selectively excite the central transition and SWFTPPM15 ^{1}H decoupling scheme was used during evolution and acquisition.

Table S3: ^{11}B isotropic chemical shift (±0.2 ppm), quadrupolar coupling constant C_Q(± 0.1 MHz) and electric field gradient asymmetry parameter η_Q (± 0.05) obtained via lineshape deconvolution of ^{11}B{^{1}H} MAS NMR spectra of **18c-batch 1** depicted in figure S104, S105 and S112 and ^{11}B spectra extracted from ^{11}B{^{31}P} CP-INEPT NMR experiments.

method	species	δ_{CS} / ppm	C_Q/MHz	η_Q	δ_{CS} /ppm calc. [a]	C_Q/MHz calc. [a]	η_Q calc. [a]	rel. Int. /%	bound ^{31}P species[b]
^{11}B MAS NMR 7.05 T (300 MHz)	1	7.3	2.38	0.25	9.2	2.49	0.26	17.6	---
	2	6.9	2.36	0.28	8.5	2.27	0.33	17.2	---
	3	6.5	2.37	0.28	7.3	2.24	0.33	17.4	---
	4	-8.4	1.05	0.35	-3.3	1.17	0.11	13.3	2
	5	-8.9	1.10	0.20	-4.9	1.04	0.17	13.6	3
	6	-9.6	1.09	0.20	-5.4	1.13	0.06	13.5	1
	7	-8.8	1.08	0.20	---	---	---	7.4	4
^{11}B MAS NMR 11.74 T (500 MHz)	1	7.6	2.38	0.20	9.2	2.49	0.26	17.1	---
	2	6.7	2.34	0.25	8.5	2.27	0.33	17.0	---
	3	5.8	2.33	0.20	7.3	2.24	0.33	16.3	---
	4	-8.3	1.05	0.30	-3.3	1.17	0.11	15.0	2
	5	-9.0	1.10	0.20	-4.9	1.04	0.17	14.2	3
	6	-9.7	1.08	0.20	-5.4	1.13	0.06	14.9	1
	7	-8.9	1.08	0.20	---	---	---	5.5	4
^{11}B{^{31}P} CP-INEPT 7.05 T (300 MHz)	4	-8.4	1.03	0.26	-3.3	1.17	0.11	---	2
	5	-8.5	1.13	0.20	-4.9	1.04	0.17	---	3
	6	-9.0	1.08	0.21	-5.4	1.13	0.06	---	1
	7	-8.8	1.12	0.20	---	---	---	---	4

[a] Calculated shifts assigned to the species in such a way that the trend form high to low shift values is matched. Quadrupolar parameters of ^{11}B were assigned to the respective species.

[b] ^{31}P species listed in table 2 assigned to boron species using ^{11}B{^{31}P} CP-INEPT NMR data.

Table S4: ^{31}P parameters obtained via line shape deconvolution of ^{31}P{^{1}H} CPMAS NMR spectra of **18c-batch 1** depicted in figure S106-S109. Estimated errors: ±0.2 ppm and ±2 Hz for δ_{CS}^{iso} and J, respectively.

method	species	δ_{CS} / ppm	J(^{31}P-^{11}B) /Hz	J(^{31}P-^{10}B) /Hz	xG/(1-x)L	d(^{31}P-^{11}B) /Hz	d(^{31}P-^{10}B) /Hz	rel. Int. /%
CPMAS 7.05 T (300 MHz) ^{1}H dec.	1	15.0	80	23	0.3	5	10	34.1
	2	13.2	82	24	0.1	5	10	21.7
	3	11.7	78	23	0.3	0	0	35.2
	4	10.1	60	18	0.5	0	0	9.0
CPMAS 7.05 T (300 MHz) ^{1}H, ^{11}B dec.	1	15.4	---	23	0.0	---	10	34.1
	2	13.7	---	24	0.1	---	10	20.3
	3	12.2	---	23	0.3	---	10	33.9
	4	10.8	---	23	0.5	---	10	11.7
CPMAS 11.74 T (500 MHz) ^{1}H dec.	1	14.8	80	23	0.3	3	6	31.1
	2	13.2	82	24	0.1	5	10	24.5
	3	11.7	78	23	0.3	0	0	35.4
	4	10.1	---	---	0.5	0	0	9.0
MAS 7.05 T (300 MHz) ^{1}H dec.	1	14.9	80	23	0.3	6	12	32.4
	2	13.2	82	24	0.1	4	8	29.0
	3	11.7	76	23	0.3	0	0	31.4
	4	10.0	60	18	0.5	0	0	7.2

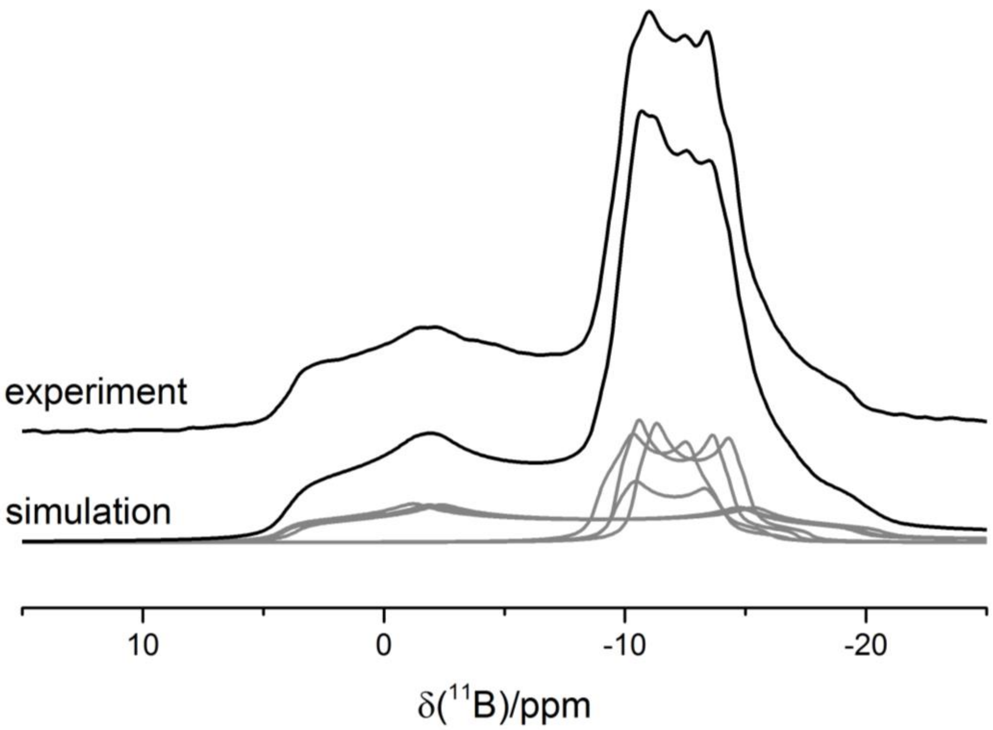

Figure S104. $^{11}B\{^1H\}$ MAS NMR spectrum of **18c-batch 1** measured at a field strength of 7.05 T and a spinning speed of 12.5 kHz. SWFTPPM15 ^1H decoupling was applied during acquisition.

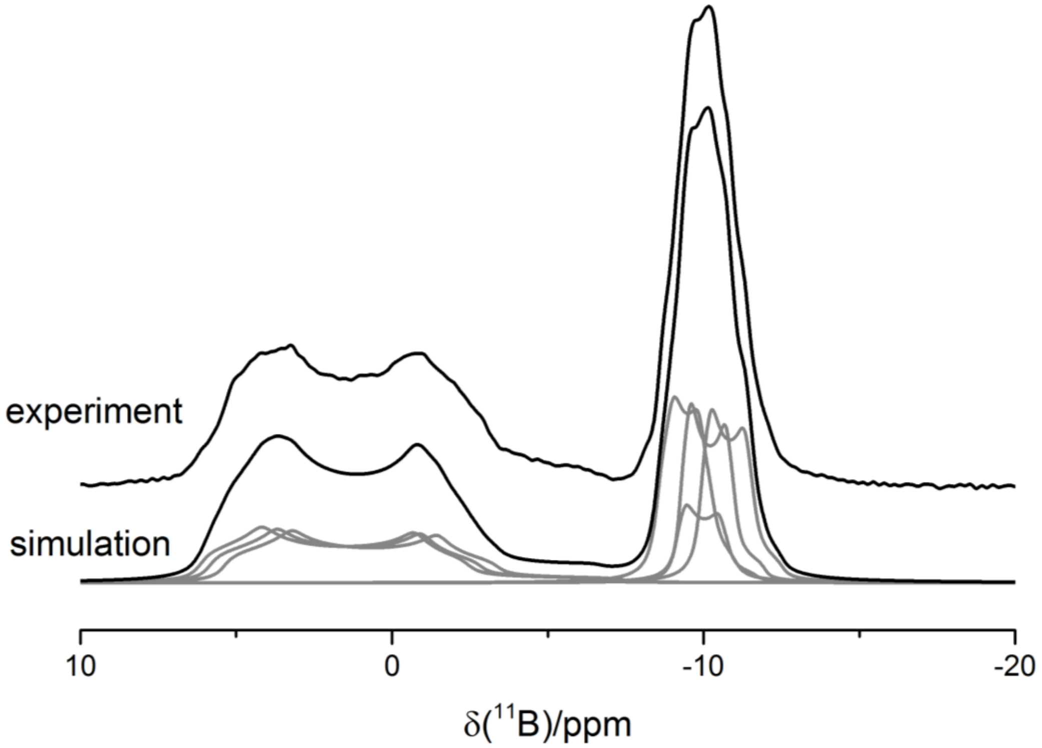

Figure S105. $^{11}B\{^1H\}$ MAS NMR spectrum of **18c-batch 1** measured at a field strength of 11.74 T and a spinning speed of 12.5 kHz. SWFTPPM15 ^1H decoupling was applied during acquisition.

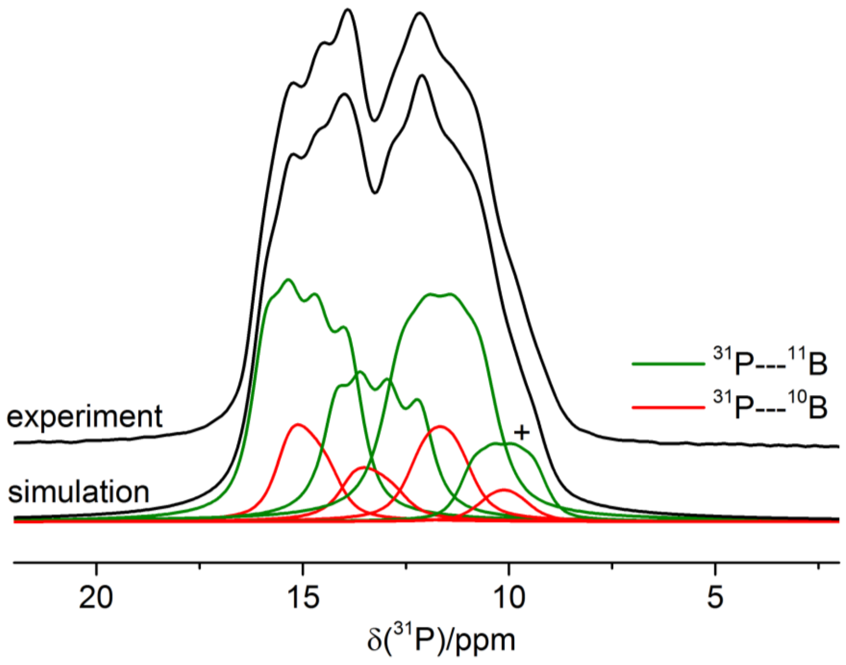

Figure S106. $^{31}P\{^1H\}$ CPMAS NMR spectrum of **18c-batch 1** measured at 7.05 T using a spinning frequency of 12.5 kHz. SWFTPPM15 ^{1}H decoupling was applied during acquisition. The symbol + denotes a spectral component attributed to an impurity.

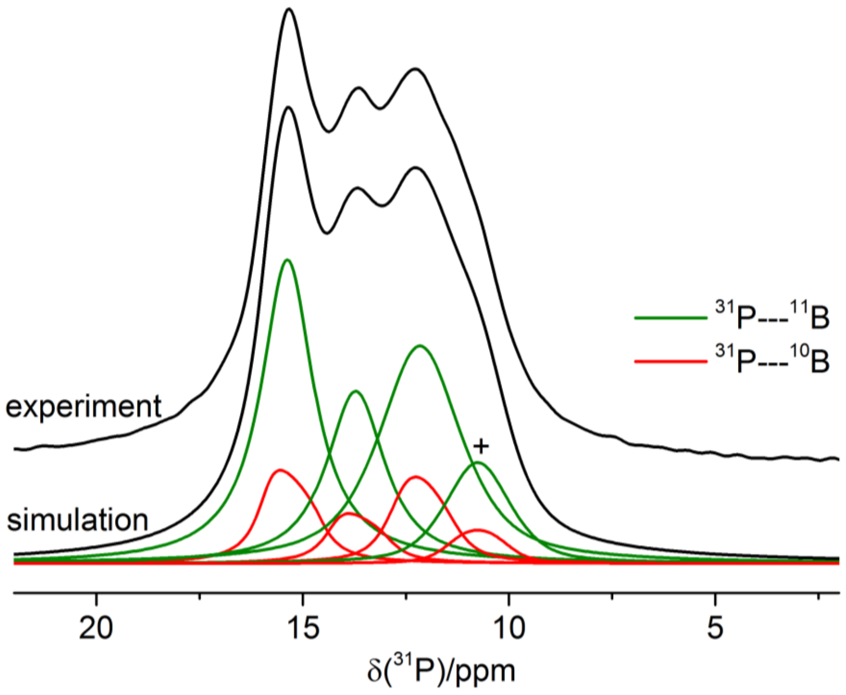

Figure S107. $^{31}P\{^1H\}$ CPMAS NMR spectrum of **18c-batch 1** measured at 7.05 T using a spinning frequency of 12.5 kHz. SWFTPPM15 ^{1}H and ^{11}B decoupling was applied during acquisition. The symbol + denotes a spectral component attributed to an impurity.

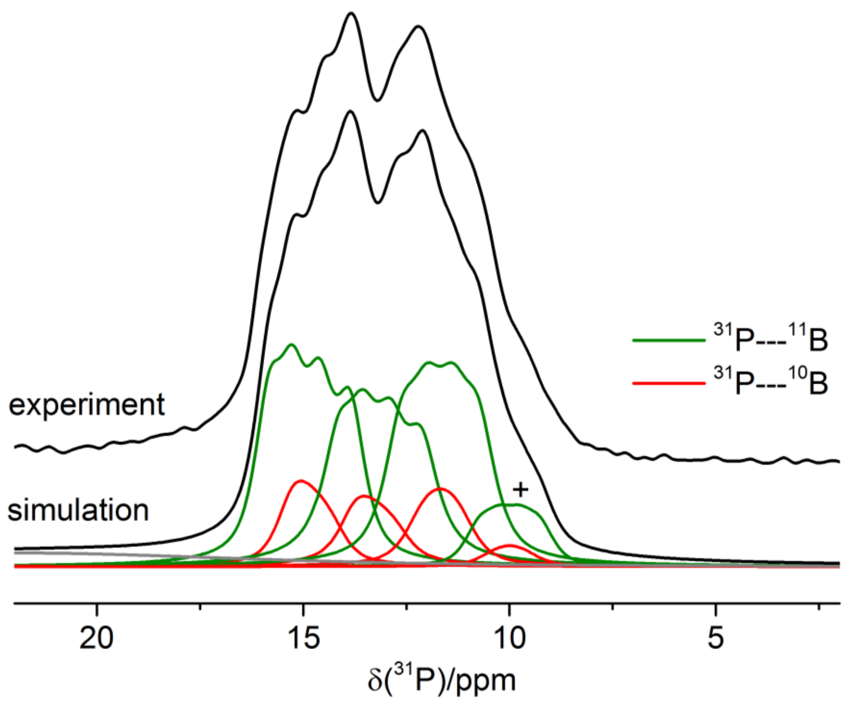

Figure S108. $^{31}P\{^{1}H\}$ MAS NMR spectrum of **18c-batch 1** measured at 7.05 T using a spinning frequency of 12.5 kHz. SWFTPPM15 ^{1}H decoupling was applied during acquisition. The symbol + denotes a spectral component attributed to an impurity.

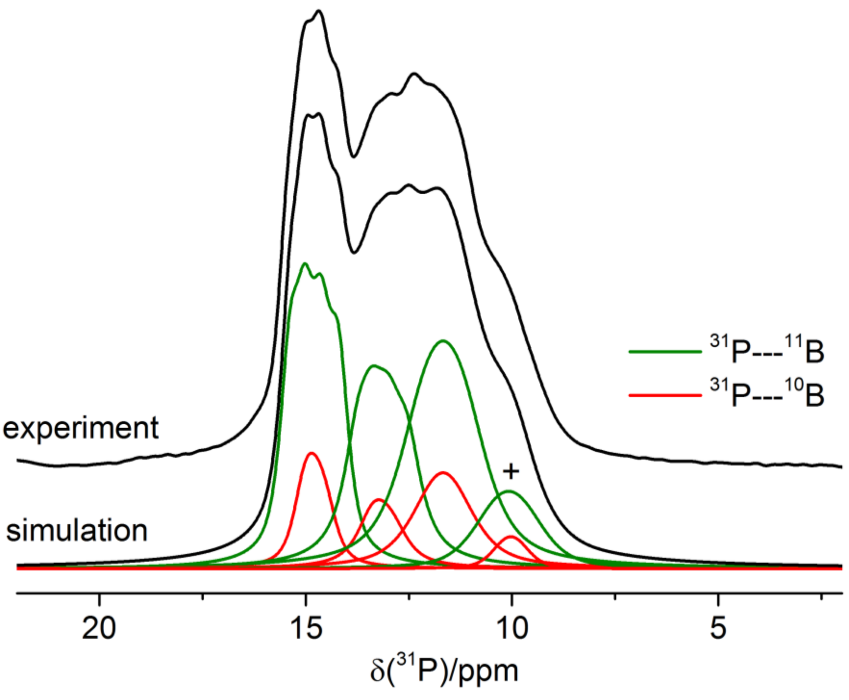

Figure S109. $^{31}P\{^{1}H\}$ CPMAS NMR spectrum of **18c-batch 1** measured at 11.74 T using a spinning frequency of 12.5 kHz. SWFTPPM15 ^{1}H decoupling was applied during acquisition. The symbol + denotes a spectral component attributed to an impurity.

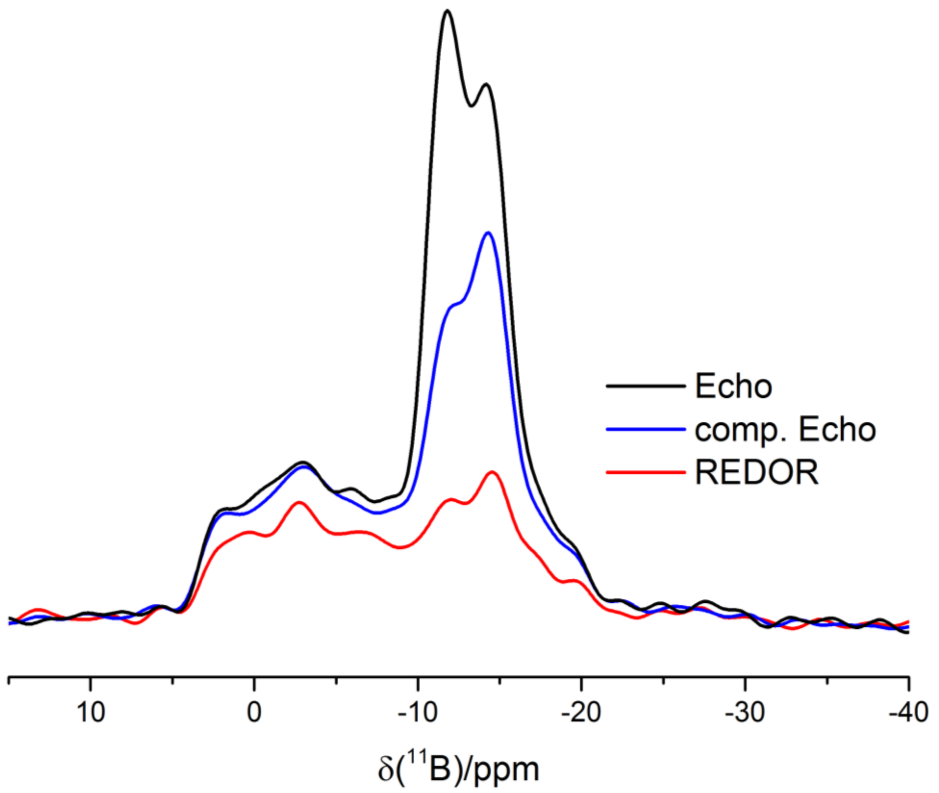

Figure S110. $^{11}B\{^{31}P\}$ REDOR NMR spectra of **18c-batch 1** measured at 7.05 T with a spinning frequency of 12.5 kHz. Depicted are 1D spectra after an evolution time of 2.9 ms. SWFTPPM15 ^{1}H decoupling was applied during acquisition.

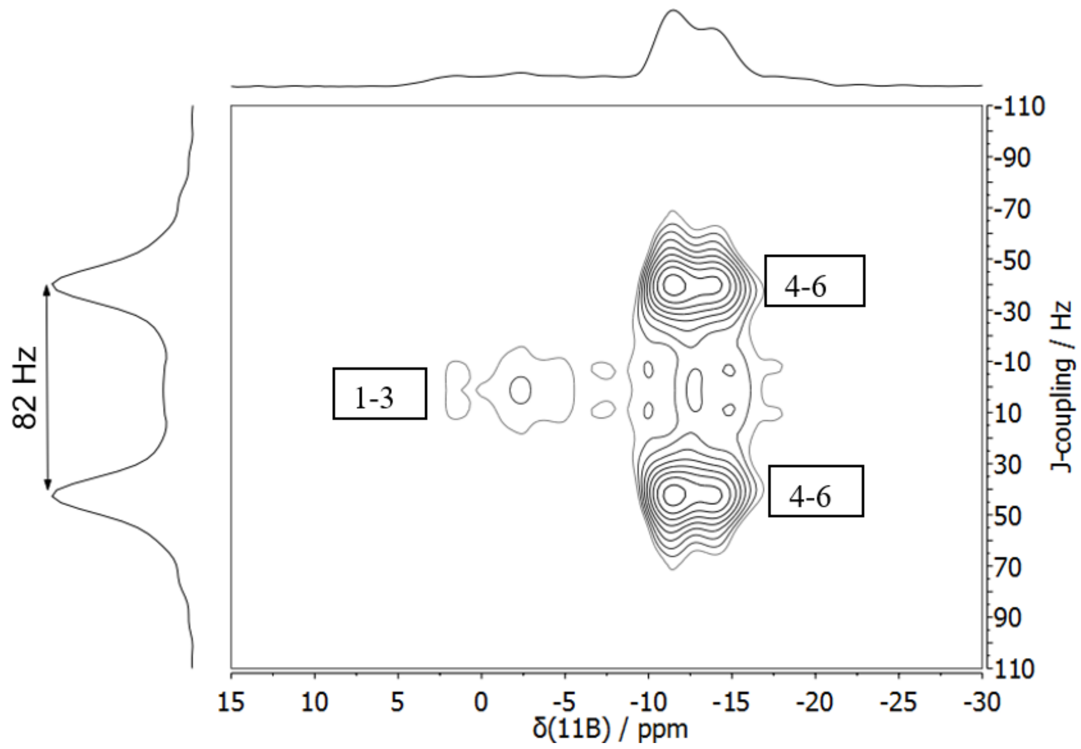

Figure S111. $^{11}B\{^{31}P\}$ J-resolved NMR spectrum of **18c-batch 1** measured at 7.05 T with a spinning frequency of 12.5 kHz. SWFTPPM15 ^{1}H decoupling scheme was used during evolution and acquisition.

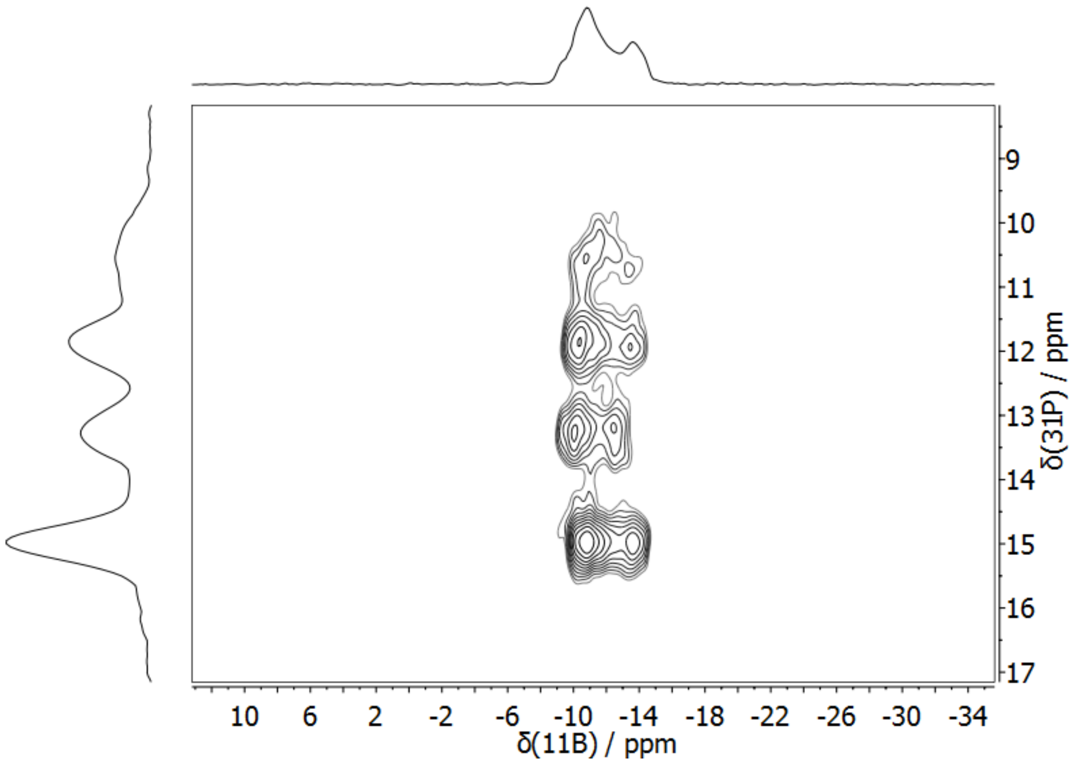

Figure S112 $^{11}B\{^{31}P\}$ CP-INEPT NMR of **18c-batch 1** measured at a field strength of 7.05 T and a spinning speed of 12.5 kHz. Soft pulses on boron were applied to selectively excite the central transition and SWFTPPM15 ^{1}H decoupling scheme was used during evolution and acquisition.

Table S5: ^{31}P parameters obtained via line shape deconvolution of $^{31}P\{^{1}H\}$ CPMAS NMR spectra of **18c-batch 2** depicted in figure S113. Estimated errors: ±0.2 ppm and ±2 Hz for δ_{CS}^{iso} and J, respectively.

method	species	δ_{CS} / ppm	J(^{31}P-^{11}B) /Hz	J(^{31}P-^{10}B) /Hz	xG/(1-x)L	d(^{31}P-^{11}B) /Hz	d(^{31}P-^{10}B) /Hz	rel. Int. /%
CPMAS 7.05 T (300 MHz) ^{1}H dec.	1	14.8	81	23	0.3	5	10	31.4
	2	13.2	84	24	0.1	5	10	21.9
	3	11.6	80	23	0.3	0	0	32.9
	4	10.2	80	22	0.5	0	0	13.8

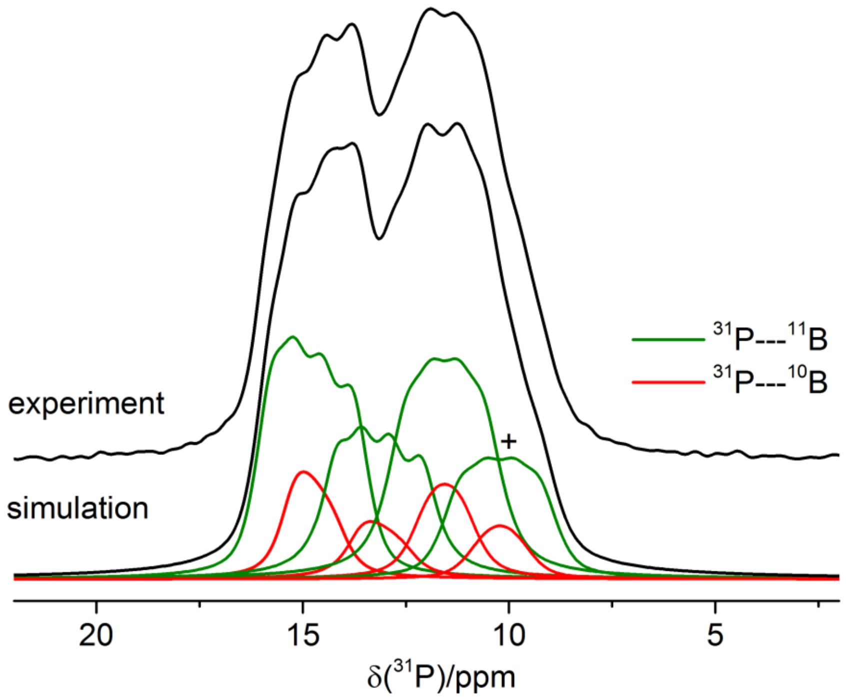

Figure S113. $^{31}P\{^{1}H\}$ CPMAS NMR spectrum of **18c-batch 2** measured at 7.05 T using a spinning frequency of 12.5 kHz. SWFTPPM15 ^{1}H decoupling was applied during acquisition. The symbol + denotes a spectral component attributed to an impurity.

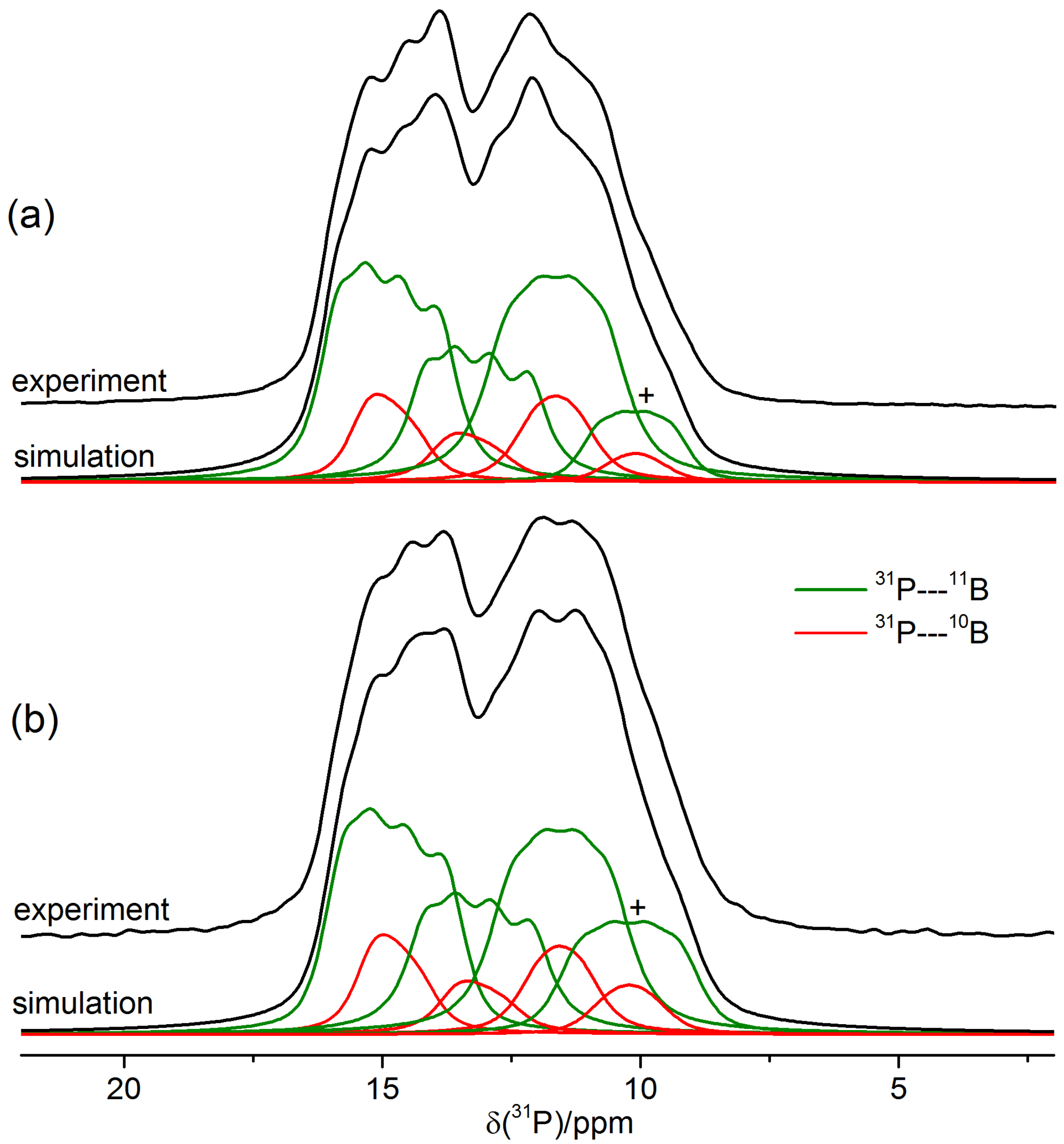

Figure S114. $^{31}P\{^1H\}$ CPMAS NMR spectrum of **18c-batch 1** (a) and **batch 2** (b) measured at 7.05 T using a spinning frequency of 12.5 kHz. SWFTPPM15 ^1H decoupling was applied during acquisition. The symbol + denotes a spectral component attributed to an impurity, based on the fact that its contribution to the spectra varies with sample batch.

References

1. D. J. Parks, R. E. von H. Spence and W. E. Piers, *Angew. Chem. Int. Ed. Engl.*, 1995, **34**, 809-811.

2. D. J. Parks, W. E. Piers and G. P. A. Yap, *Organometallics*, 1998, **17**, 5492-5503..

3. Y. Hasegawa, G. Kehr, S. Ehrlich, S. Grimme, C. G. Daniliuc and G. Erker, *Chem. Sci.*, 2014, **5**, 797-803..

4. A. Saednya and H. Hart, *Synthesis*, 1996, 1455-1458.

5. V. Chandrasekhar, P. Sasikumar, R. Boomishankar and G. Anantharaman, *Inorg. Chem.*, 2006, **45**, 3344-3351.

6. D. G. Yakhvarov, E. Hey-Hawkins, R. M. Kagirov, Y. H. Budnikova, Y. S. Ganushevich and O. G. Sinyashin, *Rus. Chem. Bull.*, 2007, **56**, 935-942.

7. C. Overländer, J. J. Tirrée, M. Nieger, E. Niecke, C. Moser, S. Spirk and R. Pietschnig, *Appl. Organomet. Chem.*, 2007, **21**, 46-48.

8. E. Rivard and P. P. Power, *Inorg. Chem.*, 2007, **46**, 10047-10064.

9. S. Ito, H. Miyake and M. Yoshifuji, *Phosphorus Sulfur Silicon Relat. Elem.*, 2009, **184**, 917-927.

10. L. Wang, S. Zhang, Y. Hasegawa, C. G. Daniliuc, G. Kehr, G. Erker, Chem. Commun. 2017, 53, 5499-5502.

11. C. Chen, T. Voss, R. Fröhlich, G. Kehr and G. Erker, *Org. Lett.*, 2011, **13**, 62-65.

12. F. Furche, R. Ahlrichs, C. Hättig, W. Klopper, M. Sierka and F. Weigend, *Comput. Mol. Sci.*, 2014, **4**, 91-100.

13. S. Grimme, J. G. Brandenburg, C. Bannwarth and A. Hansen, *J. Chem. Phys.*, 2015, **143**, 054107..

14. S. Grimme, *Chem. Eur. J.*, 2012, **18**, 9955-9964.

15. K. Eichkorn, O. Treutler, H. Öhm, M. Häser and R. Ahlrichs, *Chem.Phys. Lett.*, 1995, **240**, 283-290.

16. K. Eichkorn, F. Weigend, O. Treutler and R. Ahlrichs, *Theor. Chem. Acc.*, 1997, **97**, 119-124.

17. Y. Zhao and D. G. Truhlar, *J. Phys. Chem. A*, 2005, **109**, 5656-5667.

18. S. Grimme, J. Antony, S. Ehrlich and H. Krieg, *J. Chem. Phys.*, 2010, **132**, 154104.

19. S. Grimme, S. Ehrlich and L. Goerigk, *J. Comput. Chem.*, 2011, **32**, 1456-1465.

20. F. Weigend and R. Ahlrichs, *Phys. Chem. Chem. Phys.*, 2005, **7**, 3297-3305.

21. F. Weigend, *Phys. Chem. Chem. Phys.*, 2006, **8**, 1057-1065.

22. A. Klamt and G. Schuurmann, *J. Chem. Soc., Perkin Trans. 2.*, 1993, 799-805.

23. A. Klamt, *J. Phys. Chem.*, 1995, **99**, 2224-2235.

24. F. Eckert and A. Klamt, COSMOtherm. 2010, (COSMOlogic GmbH & Co. KG, Leverkusen, Germany)

25. D. Massiot, F. Fayon, M. Capron, I. King, S. Le Calvé, B. Alonso, J.-O. Durand, B. Bujoli, Z. Gan and G. Hoatson, *Magn. Reson. Chem.*, 2002, **40**, 70-76.

26. R. Ahlrichs, M. Bär, M. Häser, H. Horn and C. Kölmel, *Chem. Phys. Lett.*, 1989, **162**, 165-169.

27. R. Ahlrichs, F. Furche and C. Hättig, TURBOMOLE v6.5, 2013, (University of Karlsruhe).

28. A. D. Becke, *J. Chem. Phys.*, 1993, **98**, 5648-5652.

29. P. J. Stephens, F. J. Devlin, C. F. Chabalowski and M. J. Frisch, *J. Phys. Chem.*, 1994, **98**, 11623-11627.

30. Gaussian 09, Revision A.02, M. J. Frisch, G. W. Trucks, H. B. Schlegel, G. E. Scuseria, M. A. Robb, J. R. Cheeseman, G. Scalmani, V. Barone, G. A. Petersson, H. Nakatsuji, X. Li, M. Caricato, A. Marenich, J. Bloino, B. G. Janesko, R. Gomperts, B. Mennucci, H. P. Hratchian, J. V. Ortiz, A. F. Izmaylov, J. L. Sonnenberg, D. Williams-Young, F. Ding, F. Lipparini, F. Egidi, J. Goings, B. Peng, A. Petrone, T. Henderson, D. Ranasinghe, V. G. Zakrzewski, J. Gao, N. Rega, G. Zheng, W. Liang, M. Hada, M. Ehara, K. Toyota, R. Fukuda, J. Hasegawa, M. Ishida, T. Nakajima, Y. Honda, O. Kitao, H. Nakai, T. Vreven, K. Throssell, J. A. Montgomery, Jr., J. E. Peralta, F. Ogliaro, M. Bearpark, J. J. Heyd, E. Brothers, K. N. Kudin, V. N. Staroverov, T. Keith, R. Kobayashi, J. Normand, K. Raghavachari, A. Rendell, J. C. Burant, S. S. Iyengar, J. Tomasi, M. Cossi, J. M. Millam, M. Klene, C. Adamo, R. Cammi, J. W. Ochterski, R. L. Martin, K. Morokuma, O. Farkas, J. B. Foresman, and D. J. Fox, Gaussian, Inc., Wallingford CT, 2016.

31. S. Grimme, *J. Comput. Chem.*, 2006, **27**, 1787-1799.

32. D. Feller, *J. Comput. Chem.*, 1996, **17**, 1571-1586.

33. K. L. Schuchardt, B. T. Didier, T. Elsethagen, L. Sun, V. Gurumoorthi, J. Chase, J. Li and T. L. Windus, *J. Chem. Inf. Model.*, 2007, **47**, 1045-1052.

34. D. E. Woon and T. H. D. Jr., *J. Chem. Phys.*, 1995, **103**, 4572-4585.

35. K. A. Peterson and T. H. Dunning, *J. Chem. Phys.*, 2002, **117**, 10548-10560.
